# High-Throughput and Site-Specific N-Glycosylation Analysis of Human Alpha-1-Acid Glycoprotein Offers a Great Potential for New Biomarker Discovery

**DOI:** 10.1074/mcp.RA120.002433

**Published:** 2021-01-23

**Authors:** Toma Keser, Marko Tijardović, Ivan Gornik, Edita Lukić, Gordan Lauc, Olga Gornik, Mislav Novokmet

**Affiliations:** 1 Faculty of Pharmacy and Biochemistry, University of Zagreb, Zagreb, Croatia; 2 Department of Emergency Medicine, Clinical Hospital Zagreb, Zagreb, Croatia; 3 Division of Anesthesiology for Cardiovascular Surgery and Intensive Care Medicine, Medical University of Graz, Graz, Austria; 4 Genos Ltd., Genos Glycoscience Research Laboratory, Zagreb, Croatia

**Keywords:** Alpha-1-acid glycoprotein, glycosylation, high-throughput, biomarker, type 2 diabetes, AGP, Alpha-1-acid glycoprotein, AUC, Area under the curve, BMI, Body mass index, CV, Coefficient of variation, FDR, False discovery rate, HILIC-SPE, Hydrophilic interaction chromatography based solid-phase extraction, ICU, Intensive care unit, IQR, Interquartile range, QC, Quality control, UPLC, Ultra-performance liquid chromatography

## Abstract

Alpha-1-acid glycoprotein (AGP) is an acute phase glycoprotein in blood, which is primarily synthetized in the liver and whose biological role is not completely understood. It consists of 45% carbohydrates that are present in the form of five N-linked complex glycans. AGP N-glycosylation was shown to be changed in many different diseases, and some changes appear to be disease-specific; thus, it has a great diagnostic and prognostic potential. However, AGP glycosylation was mainly analyzed in small cohorts and without detailed site-specific glycan information. Here, we developed a cost-effective method for a high-throughput and site-specific N-glycosylation LC-MS analysis of AGP which can be applied on large cohorts, aid in search for novel disease biomarkers, and enable better understanding of AGP’s role and function in health and disease. The method does not require isolation of AGP with antibodies and affinity chromatography, but AGP is enriched by acid precipitation from 5 μl of bloodplasma in a 96-well format. After trypsinization, AGP glycopeptides are purified using a hydrophilic interaction chromatography-based solid-phase extraction and analyzed by reversed-phase-liquid chromatography-electrospray ionization-MS. We used our method to show for the first time that AGP N-glycan profile is stable in healthy individuals (14 individuals in three time points), which is a requirement for evaluation of its diagnostic potential. Furthermore, we tested our method on a population including individuals with registered hyperglycemia in critical illness (59 cases and 49 controls), which represents a significantly increased risk of developing type 2 diabetes. Individuals at higher risk of diabetes presented increased N-glycan branching on AGP’s second glycosylation site and lower sialylation of N-glycans on AGP’s third and AGP1’s fourth glycosylation site. Although this should be confirmed on a larger prospective cohort, it indicates that site-specific AGP N-glycan profile could help distinguish individuals who are at risk of type 2 diabetes.

## Highlights

• Cost-effective method for high-throughput detailed AGP glycoprofiling is presented.
• Site-specific AGP N-glycan profile can be obtained from 5 μl of blood plasma.
• AGP N-glycan profile is stable in a healthy individual.
• AGP glycoprofile could help identify individuals who are at risk of type 2 diabetes.

## Graphical Abstract

**Figure:**
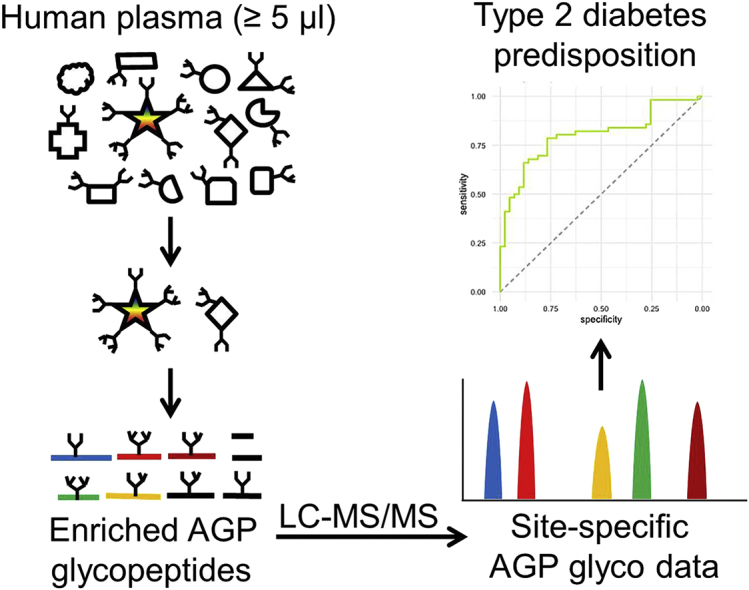

## In Brief

For the first time, a cost-effective method for a high-throughput detailed AGP N-glycosylation profiling was developed, which includes site-specific glycosylation information. Using the method, it was demonstrated that AGP N-glycan profile is stable in a healthy individual. Furthermore, using the method on a pilot cohort, it was found that site-specific AGP N-glycan profile could help distinguish individuals who are at risk of type 2 diabetes. The method presents a new valuable tool for investigation of AGP’s large biomarker potential.

Alpha-1-acid glycoprotein (AGP) or orosomucoid is a 41 to 43 kDa glycoprotein, and carbohydrates account for approximately 45% of its mass (1). AGP is formed primarily in the liver and circulates in the plasma of healthy humans at concentrations between 0.36 and 1.46 mg/ml with a mean of 0.77 mg/ml, with men having slightly higher levels than women (2, 3). AGP is one of the major acute phase proteins in humans, and in most disease states including inflammation, infection, and cancer, AGP levels increase from 2-fold to 6-fold (2). While the biological role of AGP remains unclear, it has been demonstrated to regulate immunity and play a role in both pro-inflammatory and anti-inflammatory response (4, 5, 6). Furthermore, AGP has the ability to bind and carry endogenous ligands (*e.g.*, steroid hormones, histamine, serotonin, and melatonin) and numerous basic and neutral lipophilic drugs (7).

Three genes (*AGP-A*, *AGP-B*, and *AGP-B′*) which are located on chromosome 9 encode human AGP (4). *AGP-A* encodes AGP1 and is expressed in the liver at more than 100-fold that of *AGP-B* and *AGP-B′*. *AGP-B* and *AGP-B′* are identical in structure and encode AGP2, which differs from AGP1 by 22 amino acids (7, 8). AGP1 is polymorphic with three closely related genetic variants: F1, F2, and S, differing in less than five amino acids (F1 has Gln-38/Val-174; F2 has Gln-38/Met-174 and S has Arg-38/Val-174) and are generally referred to as AGP1∗F1, AGP1∗F2, and AGP1∗S (9, 10). *AGP-B* and *AGP-B′* (AGP2) encode the genetic variant AGP2∗A (7). Most individuals possess a mixture of these variants (11). F1 + S + A is the most common phenotype (50%), followed by F1 + A (35%) and S + A (15%). The F1 and S variants are distributed worldwide, but the F2 variant is limited to Europeans, North Africans, and West Asians (12, 13, 14). In most healthy individuals, the molar ratio of F1∗S to A variant in blood is ∼2 to 3:1 (7, 11). The ratio can increase up to 8:1 in a disease setting because the AGP1 is inducible (15).

Glycosylation, the addition of oligosaccharide chains (glycans) is one of the most abundant cotranslational and posttranslational modifications (16). Most glycans present on plasma proteins are classified as N-glycans because they are attached by amide linkage to the nitrogen of the protein’s asparagine. For asparagine to be able to receive N-glycan, it has to be part of the Asn-X-Ser/Thr/Cys amino acid sequence, where X is any amino acid except proline. Glycan heterogeneity was shown to be associated with numerous diseases, and glycan analysis has a great diagnostic potential (17). Human AGP contains five N-linked glycans on the polypeptide backbone (Asn-33, -56, -72, -93, -103), each of which can be a biantennary, triantennary, or tetraantennary glycan and exhibit various degrees of fucosylation and sialylation (9, 18). Sialic acid, in the form of N-acetylneuraminic acid, accounts for 10 to 12% of the total number of monosaccharides and is responsible for giving AGP its low isoelectric point of 2.8 to 3.8 (4). AGP is one of the few serum glycoproteins that contain tetraantennary N-linked glycans (19).

Although the concentration of AGP alone is not diagnostic for a particular pathological condition, the altered glycosylation of AGP in different diseases provides a promising biomarker target. Altered glycosylation of AGP has been studied in a number of pathophysiological conditions. Changes in glycosylation of AGP are not only restricted to acute inflammatory conditions but also occur in a wide variety of other physiological and pathophysiological conditions like pregnancy, severe rheumatoid arthritis, liver cirrhosis, hepatitis, asthma, type I diabetes, and cancer (20, 21, 22, 23, 24, 25, 26, 27, 28, 29, 30). In general, decreased branching of AGP glycans has been demonstrated in acute inflammation, whereas an increase is associated with chronic inflammation (26, 31, 32, 33, 34). Furthermore, many inflammatory diseases are characterized by an increased fucosylation of the branches (27, 29, 35, 36, 37, 38, 39, 40) and an increased sialic acid content (38, 41, 42). AGP fucosylation levels also show a promising potential as a marker for prognosis in cancer (27, 43, 44). Nevertheless, AGP glycans changes appear to be specific for some inflammatory diseases, while not for others (30, 32). This is not the case with the most studied glycoprotein—immunoglobulin G, whose glycosylation pattern cannot be used as a stand-alone disease-specific biomarker and is of more value as a biomarker of general immune activation (45). Therefore, AGP glycosylation has a great potential for new, disease-specific biomarker discovery.

Glycosylation changes of AGP were studied in many diseases, but it was mainly done on small sample sizes and with lectin-based techniques, which allow only a very rough semiquantitation of a few different AGP glycoforms. Recently, AGP glycosylation is more and more analyzed with MS-based methods, but they mainly use commercial AGP (46, 47, 48, 49), expensive affinity chromatography for the AGP isolation, and/or study glycosylation on a released glycan level, where site-specific glycosylation information is lost, and contaminating glycoproteins (possibly coeluted/coprecipitated during the isolation process) are confounding the results (30, 38, 40, 50).

Here, we developed a cost-effective method for a high-throughput and site-specific N-glycosylation LC-MS analysis of AGP on a glycopeptide level. Our method allows a detailed analysis of AGP glycosylation in hundreds of plasma samples in 1 week. It does not require isolation of AGP with antibodies and affinity chromatography, but AGP is analyzed directly from the “seromucoid” fraction obtained from 5 μl of human plasma. We provide the first information about intraindividual temporal stability of AGP glycosylation in healthy individuals, which is a requirement for evaluation of its diagnostic potential.

Furthermore, we tested our method on a population including individuals with registered hyperglycemia in critical illness, which represents a significantly increased risk of developing type 2 diabetes (51, 52). Recently, we showed on the same population that the increased branching of plasma N-glycan structures is associated with higher risk of developing type 2 diabetes (53). There, we measured the whole plasma protein N-glycome, which is comprised of different glycans originating from many different glycoproteins. Thus, it was hard to distinguish the real cause and the rationale behind that change, because measured glycan levels in total plasma protein N-glycome are product of both composition of glycans attached to different proteins, as well as the relative abundance of those proteins. The most likely candidate for the origin of the detected glycan changes is AGP, considering that it is the source of the most branched glycan structures present in the whole plasma protein N-glycome (19). Because identifying the exact glycoproteins that contribute to those differences would probably help to develop stratification methods which could reliably distinguish individuals who are at risk of type 2 diabetes development, we have chosen this population for the pilot study of our method.

## Experimental Procedures

### Experimental Design and Statistical Rationale

The study contains three sets of experiments: method development, intraindividual temporal stability study, and pilot study with individuals with and without hyperglycemia during critical illness.

In the method development part, the method was developed and optimized using two sets of standards: a blood plasma standard, made from pooled plasma from the two populations described below, and AGP standard, isolated from human plasma (≥99%), which was purchased from Sigma-Aldrich. Repeatability of the method was assessed by measuring a coefficient of variation (CV) of the pooled plasma standard technical replicates. Intraplate repeatability was measured using 10 replicates which were randomized across one 96-well plate, and interplate repeatability was measured using 10 replicates which randomized across two different 96-well plates. Interplate repeatability was also assessed with batch-corrected data from the same replicates (see below for more details about batch effect correction). For a sensitivity test, AGP was enriched from 50, 20, 10, 5, 2.5, and 1 μl of the pooled plasma standard, all in triplicates.

All different glycoforms which were identified and annotated from the pooled plasma standard were included for the quantification and further data analysis in the intraindividual temporal stability study and the pilot study. Extracted signals were summed and normalized to total integrated area per glycosylation site. Normalization serves the purpose of removing the variation in signal intensity between samples and allows for their comparison. Interpreting and understanding data from many different glycoforms on each glycosylation site for each sample is challenging; so to simplify this information, glycans within each glycopeptide were grouped by their common structural and compositional characteristics to calculate the derived glycosylation traits.

The intraindividual temporal stability study was performed to provide information about intraindividual temporal stability of AGP glycosylation in healthy individuals. The samples were measured from 14 healthy and age-matched male students, at three time points (0, 6, and 10 weeks) for each student. All samples were randomized across a 96-well plate before the analysis. A total number of eight random students’ technical duplicates (across all three time points) served to calculate a baseline CV of the method itself. From longitudinal samples of each student, an intraindividual CV was calculated, whereas an interindividual CV was calculated from all students’ samples within each time point.

A total number of 108 subjects were included in the pilot study (59 who developed hyperglycemia during critical illness *versus* 49 controls who did not develop hyperglycemia during critical illness), and each subject was sampled once. All samples were randomized across two 96-well plates before the analysis.

Variation in laboratory conditions during sample preparation and analysis can lead to slight shifts in obtained results; therefore, batch effect correction was performed to obtain more accurate data. Measurements were log-transformed before batch correction which was performed by applying the ComBat method (R package “sva”). Order number of sample plate, which represented the laboratory source of variation, was modeled as a batch covariate. Estimated batch effect was then subtracted from log-transformed measurements, reducing the introduced experimental noise.

To evaluate association between each glycosylation trait and hyperglycemia, we used logistic regression. The glycan trait was considered as an independent variable and the presence of hyperglycemia as a dependent variable. To remove the effect of differences in age and sex between the case and control groups in the final results, these variables were included as additional covariates, so their effect was excluded from the coefficient calculated for derived trait effect. The calculated coefficient represents the change in log odds ratio (the change in the natural logarithm of the ratio of odds for a patient to be hyperglycemic *versus* not being hyperglycemic) for each unit of the derived trait. In the model, instead of using originally calculated glycan trait values, they were transformed to standard Normal distribution by the inverse transformation of ranks to Normality (R package “GenABEL”). Variables transformed this way always have the same standardized variance, so the estimated odds ratio can be compared between different glycan traits. This means that the calculated B coefficient represents the change in probability for a patient to be hyperglycemic (expressed in units on a log-odds scale) for each unit of standard deviation the glycan trait value increases. The analysis was repeated for each individual glycan trait. Considering multiple tests performed, Benjamini-Hochberg method was used to control the false discovery rate (FDR) with *p*-value <0.05 considered as significant.

Six glycan traits identified as statistically significant in the previous step were then assessed for their ability to predict individuals with increased type 2 diabetes risk. Four predictive logistic regression models were built and compared: one using only age and sex as predictors, one using age, sex, body mass index (BMI), and family history of diabetes, and two more corresponding to previous two, but with six glycan traits included. The models were assessed using a receiver operating characteristic curve analysis and evaluated based on area under the curve (AUC) criteria (R package “pROC”).

All data analysis and visualization were done using R programming language (version 3.5.1).

### Population for the Intraindividual Temporal Stability Study

Fourteen male physical education students (age 19 ± 0.7 years) participated in the study. All participants were screened for cardiovascular diseases, muscle injuries, or ongoing medical treatment before their inclusion into the experimental protocol. Participants were instructed to refrain from alcohol and cigarette consumption as well as antioxidant supplementation throughout the study. The blood samples were taken from each participant at three time points: 0, 6, and 10 weeks. The blood was collected in vacuum tubes containing EDTA with 20-G straight needle venipuncture from the antecubital vein. The EDTA tubes were immediately centrifuged (at 1370*g* for 10 min) to separate erythrocytes from plasma. Subsequently, plasma supernatant was aspirated into a series of 1 ml aliquots and stored at −80 °C until analysis.

### Population for the Pilot Study (Individuals With and Without Hyperglycemia During Critical Illness)

This population was used previously for the analysis of the total plasma N-glycome (53). Individuals admitted to the medical intensive care unit (ICU) at the University Hospital Centre Zagreb during a period of 6 months (February to July 2013) were included in the study. Adults (aged >18 years old) with negative history of diabetes who were admitted to the ICU and discharged from the hospital alive were eligible for inclusion. We excluded individuals diagnosed with diabetes or impaired glucose tolerance and/or impaired fasting glucose before or during hospitalization and those with documented gestational diabetes, pregnant women, and individuals taking glucocorticoids during or 3 months before the admission. Informed consent was obtained from participants by a member of the study team at the discharge from the ICU or the hospital. Consenting participants were asked to attend a follow-up appointment, 6 to 8 weeks after the hospital discharge. At this visit, inclusion/exclusion criteria were confirmed. Complete blood count and C-reactive protein levels were determined to exclude any persisting inflammatory process. Individuals with elevated markers of inflammation were retested after 2 weeks. All participants underwent an oral glucose tolerance test and measurement of glycated hemoglobin A1c to identify preexisting diabetes. The American Diabetes Association criteria for diagnosis of diabetes were employed, and any individual diagnosed with existing diabetes or with impaired glucose tolerance was excluded. Height, weight, and BMI were recorded, and family history of diabetes was documented. For all participants, fasting blood samples for N-glycan profiling were collected in tubes containing EDTA anticoagulant, plasma was separated immediately (at 1370*g* for 10 min) and stored at −20 °C until analysis. In total, 108 participants were enrolled in the study. Their demographic data are summarized in supplemental Table S1.

Both studies are designed in accordance with the Declaration of Helsinki, supported by written informed consent from all individuals and approvals from eligible local Ethics Committees—Ethical Committee of the University of Zagreb Faculty of Pharmacy and Biochemistry and Ethical Committee of the Clinical Hospital Centre Zagreb, respectively.

### Enrichment of AGP From Plasma Samples

The AGP was enriched by precipitation of the “seromucoid” fraction from human plasma samples (54). 50 μl of plasma in each well was mixed with 1.2 M perchloric acid (Merck) 1:1 (v:v) in a 96-well PCR plate (Thermo Scientific) and centrifuged for 20 min at 1200*g* at 5 °C. The supernatant was transferred to a new PCR plate using in-house 3D printed adapters (supplemental Fig. S1) in a high-throughput manner by low-speed centrifugation at 15*g* for 30 s, and 1/10th (≈70 μl) volume of 2% phosphotungstic acid (Sigma-Aldrich) in 2M HCI (VWR International) was added to the transferred supernatants and then centrifuged for 20 min at 1200*g* at 5 °C. The supernatants were discarded to an empty PCR plate using the 3D printed adapters and by low-speed centrifugation at 15*g* for 30 s. The remaining precipitate, containing the “seromucoid” fraction with enriched AGP, was solubilized by adding ≈40 μl of 0.1 M NaOH (Sigma-Aldrich)—the solution has to become clear.

For the sensitivity test, AGP was enriched from 50, 20, 10, 5, 2.5, and 1 μl of pooled human plasma standard, all in triplicates. Each sample was adjusted to the same starting volume (50 μl) by water. All the reagents for the enrichment were added in equal amounts, except for NaOH, whose concentration was reduced based on the starting amount of plasma.

### Reduction, Alkylation, and Trypsin Digestion

Five microliter of 1.5% RapiGest SF Surfactant (Waters) in 30 mM ammonium bicarbonate (Acros Organics) was added to the solubilized precipitates, and the samples were incubated for 5 min at 60 °C in an oven. After the incubation, 5 μl of 60 mM dithiothreitol (Sigma-Aldrich) was added to the samples, and the plate was incubated for 30 min at 60 °C. After cooling to room temperature, 5 μl of 160 mM iodoacetamide (Sigma-Aldrich) was added, and the samples were incubated in dark while shaking for 30 min. Afterward, 1 μl of 200 mM dithiothreitol was added to quench the iodoacetamide, and 1 μl of 2 M ammonium bicarbonate was added to set pH for trypsin digestion. Subsequently, 4 μl of 0.4 μg/μl TPCK-treated trypsin (Promega) in 50 mM acetic acid was added to each sample, and they were incubated overnight at 37 °C. After the trypsinization, 2 μl of 1 M HCl was added to the samples, and they were incubated at 37 °C for 45 min to degrade the RapiGest SF.

For the sensitivity test, all the reagents were added in equal amounts, except for RapiGest SF and trypsin, whose concentrations were corrected based on the starting amount of plasma.

### Glycopeptide Enrichment With HILIC-SPE

Glycopeptides were enriched using a HILIC-SPE on a 96-well polypropylene filter plate (OrochemA). Five milligram of Chromabond HILIC beads (Macherey-Nagel) in 0.1% TFA in water (Sigma-Aldrich) (50 mg/ml suspension) was added to each well. Solvent was removed by application of vacuum using a vacuum manifold (Millipore Corporation). All wells were prewashed using 2× 250 μl of 0.1% TFA in water, followed by equilibration using 2× 250 μl of 90% acetonitrile (VWR International) + 10% 0.1% TFA in water. The samples were diluted with 450 μl of 0.1% TFA in acetonitrile and loaded into the wells, which were subsequently washed 2× with 250 μl of 90% acetonitrile +10% 0.1% TFA in water. Enriched glycopeptides were eluted into a PCR plate with 200 μl of 0.1% TFA in water. The eluates were immediately dried down in a SpeedVac Vacuum Concentrator (Thermo Scientific) and stored at −20 °C until analysis.

### Reversed-Phase–Liquid Chromatography–Electrospray Ionization–MS(/MS)

Separation and measurements were performed using liquid chromatography coupled to a Compact mass spectrometer (Bruker Daltonics). AGP glycopeptides were separated on a nanoACQUITY ultra-performance liquid chromatography (UPLC) instrument (Waters). The UPLC was coupled to the Compact using an Apollo ion source (Bruker) equipped with CE sprayer (Agilent). Dried samples were reconstituted in 30 μl of ultrapure water and were diluted 10 times before loading onto an Acclaim PepMap100 C8 (5 mm × 300 μm i.d.) trap column (Thermo Fisher Scientific). On the trap column, the glycopeptides were washed 3 min with 0.1% TFA in water (solvent A) at a flow rate of 40 μl/min. Separation of the tryptic AGP glycopeptides was based on differences in their peptide backbone and was performed on a Halo C18 nano-LC column (150 mm × 75 μm i.d., 2.7 μm HALO fused core particles; Advanced Materials Technology). In the first 5 min, solvent B (80% acetonitrile +20% 0.1% TFA in water) was increased from 0% to 20% and during the next 11.5 min from 20% to 50% with the total 16.5 min gradient. Flow rate was 1 μl/min, and column temperature was 30 °C. Mass spectra were recorded with two averages at a frequency of 0.5 Hz in a mass range from m/z 100 to m/z 4000. Collision energy and ion energy were set at 4 eV. Argon was used as a collision gas. For the annotation of AGP glycopeptides, fragmentation spectra of glycan and peptide portion of each analyte were recorded in a stepwise mode as previously described, with some modifications (55). Briefly, data were acquired with the MS fixed to 1 Hz while the fragment spectra rate was variable, between 1 and 2 Hz, depending on the parent ion intensity. Precursors were automatically selected, whereas for the glycopeptides which were not selected in the automatic mode, include list was created making sure to capture MS/MS data of low abundant AGP glycopeptides. Isolation windows were set at Otof control (Bruker Daltonics) default values depending on the precursor m/z. Collision energies were defined as linear curve dependent on the m/z value of the selected precursor and its charge state, starting from 25 eV at m/z 1000 up to 40 eV at m/z 2000. These values were applied to 20% of time of the MS^2^ event. For the remaining 80% of the time, collision energy was doubled. The nanoACQUITY UPLC system was operated by HyStar software, version 4.2 (Bruker).

### Data Processing

For the analysis of proteomic data MaxQuant (version 1.6.10.43) software was used. Human reference proteome sequence (release version: 2019_07) was downloaded from Uniprot (ProteinID: UP000005640) and uploaded to MaxQuant, containing 75,069 protein entries associated with human proteome. Decoy mode was set at revert. Instrument type was set to Bruker Q-TOF with first search peptide tolerance set to 0.1 Da. All other peak picking parameters (MaxQuant group specific parameters) were left at default settings for Bruker Q-TOF instrument. Main peptide search tolerance (precursor mass tolerance) was set to 0.006 Da. TOF MS/MS match tolerance (fragment mass tolerance) was set at default value of 50 ppm. Enzyme was set to Trypsin/P with specific cleavages and with maximum of two missed cleavages. FDR is determined by the target-decoy approach. At both levels, the protein and at the peptide spectrum match level, FDR was set to 1%. Carbamidomethyl(C) was set as fixed modification with variable modifications of Oxidation (M) and Acetyl (Protein N-term). Spontaneous cyclization of glutamine to pyroglutamic acid was searched manually for the tryptic glycopeptides from glycosylation site IV. Data from MS^1^ level were used for quantification of identified proteins in the processed raw files. Intensity column (protein groups table of the MaxQuant output) represents summed up extracted ion current of all isotopic clusters associated with the identified amino acid sequence. Relative abundance of each identified protein was expressed as percentage of total intensity (supplemental Table S2).

AGP glycopeptides were manually annotated using MS and MS/MS spectra generated in the data dependent acquisition mode. Precursor ions were selected in auto MS/MS mode where three most abundant signals in the MS spectra were picked for the subsequent fragmentation. Additionally, include list was created to obtain AGP glycopeptide MS/MS spectra not recorded during the automatic precursor selection. During manual analysis of the recorded data, several MS/MS extracted ion chromatograms of diagnostic glycan fragments were created to identify potential glycopeptide elution times: 366^1+^ for LacNAc; 657^1+^ for LacNAc carrying sialic acid; 292^1+^ and 274^1+^ for sialic acid; 204^1+^ for the GlcNAc; and Y1 ion of each glycopeptide (peptide carrying only innermost GlcNAc). The m/z values of the most abundant glycopeptides in each identified retention time window were crosschecked with the m/z values from the internal AGP glycopeptide database, generated after the elaborate literature search of the reported AGP glycan structures (46, 47). If the match was confirmed, the MS/MS spectra of every matched glycopeptide were in detail manually annotated using Compass DataAnalysis (Bruker Daltonik GmbH), GlycoWorkbench (56), and GlycoMod (57), the latter two being operated under permissive free software licenses. During the annotation, all literature data on AGP glycan structures as well as all potential pitfalls of MS-based composition annotation were taken into consideration (58). Both, amino acid sequence and glycan composition were confirmed in the MS/MS spectra of the most abundant AGP glycopeptides (supplemental Fig. S2, showing representative annotated fragmentation spectra for each glycosylation site identified). Furthermore, additional level of conformation was performed using specialized software for glycopeptide identification—Byonic search engine (version 3.9.6, Protein Metrics). Protein database included truncated AGP1 (UniProtKB entry: P02763; release version: 2020_04) and AGP2 (UniProtKB entry: P19652; release version: 2020_04) protein sequences obtained from UniProt database. Digestion parameters reflected trypsin with fully specific digestion and no allowed missed cleavages. Fragmentation type was set to “collision-induced dissociation low energy”, while precursor and fragment mass tolerance were 20 and 30 ppm, respectively. Protein modifications, besides glycosylation, included fixed carbamidomethyl modification on cysteine residues. For glycan database, a custom list was created based on the composition of glycan structures confirmed in previous steps. The analysis was performed on several raw MS/MS files obtained from the pooled plasma standard. Search results were further loaded into Byonic-Preview software (version 3.10-4, Protein Metrics) where automatic spectra annotation was performed. Results generated by Byonic search engine as well as annotated spectra obtained for 87 out of 96 reported glycopeptides can be found in (supplemental Fig. S3, supplemental Table S3). For the glycopeptides which did not have MS/MS data at all (mostly due to low intensity, nine out of 96 reported), the annotation was done according to following criteria: same retention time window (coelution) as confirmed AGP glycopeptides, m/z value matched to internal database, presence of more than one charge states (depending on the size of the glycopeptide), and finally, delta m/z representing mass difference of one or more monosaccharides from the glycopeptide thoroughly confirmed as previously described (supplemental Fig. S4, supplemental Table S4).

Prior the relative quantification of the glycoproteomic data, MSConvert tool (ProteoWizard version 3) was used to convert all Bruker raw data into mzXML file format. LaCyTools (version 1.0.11.0.1 b.9), operated under free software license (59), was used for automated relative quantification of the MS data. Chromatograms were aligned based on the five most abundant glycopeptide signals. Targeted peak integration was performed on triply and quadruply charged species. Signals were integrated to include at least 90% of the theoretical isotopic pattern. The QC parameters for extracted data of the targeted peak integration were automatically calculated for each analyte of every sample: mass accuracy, deviation from the theoretical isotopic patter, and signal to noise ratio. Quality of the data was checked on the batch level and analytes were assessed using average QC values calculated from all analyzed samples. Criteria were set at a strict level to ensure only quality extracted data to be further processed: mass accuracy (between −30 and 30 ppm), the deviation from the theoretical isotopic pattern (IPQ; below 25%), and the signal to noise ratio (above 15) of an integrated signal. Extracted signals were summed for triply and quadruply charged ions, according to the QC assessment and normalized to total integrated area per glycosylation site (supplemental Table S5). Normalization serves the purpose of removing the variation in signal intensity between samples and allows for their comparison.

## Results and Discussion

### Development of Method for High-Throughput and Site-Specific N-Glycosylation Analysis of AGP

**Fig. 1:**
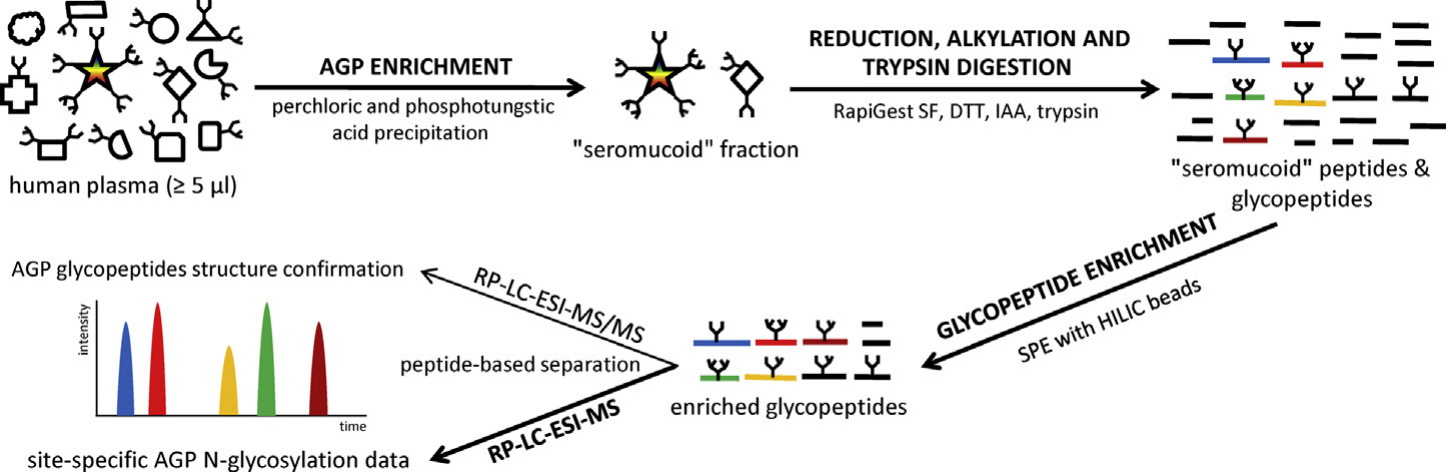
**The scheme of the method for high-throughput and site-specific N-glycosylation analysis of human AGP.** AGP, alpha-1-acid glycoprotein; HILIC-SPE, hydrophilic interaction chromatography based solid-phase extraction.

Low concentrations of AGP in human plasma present a challenge for direct detailed site-specific N-glycosylation analysis. A standard approach to overcome this issue would include protein purification by affinity chromatography, which is often a timely and costly process. To overcome this problem, a “seromucoid” fraction of plasma samples was prepared by sequential perchloric and phosphotungstic acid precipitations (54), scaled down to a 96-well format which enabled cost-effective and fast high-throughput AGP enrichment (Fig. 1). Problematic transfer of small volumes of supernatants during the process was overcome by designing and producing of in-house 3D-printed adapters which enabled reproducible and repeatable procedure (supplemental Fig. S1). According to analysis of proteomics data acquired by LC-MS, AGP1 and AGP2 accounted for an average of 14% of all proteins in the enriched fractions (supplemental Table S2). To further improve the purity of the sample and to additionally remove unglycosylated, more hydrophobic peptides, a hydrophilic interaction chromatography-based solid-phase extraction (HILIC-SPE) was introduced following the trypsin digestion of the enriched sample. The clean-up step increased the relative amount of AGP to an average of 25% (supplemental Table S2), which was adequate for the following analysis of AGP site-specific N-glycosylation.

**Fig. 2:**
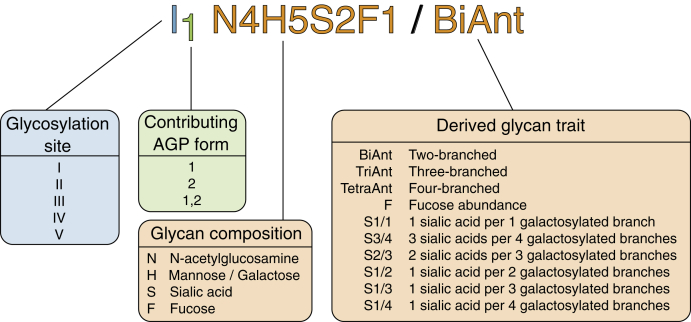
**Explanation of nomenclature used for AGP genetic variants, glycan composition, and calculated derived glycosylation traits.** AGP, alpha-1-acid glycoprotein.

**Fig. 3:**
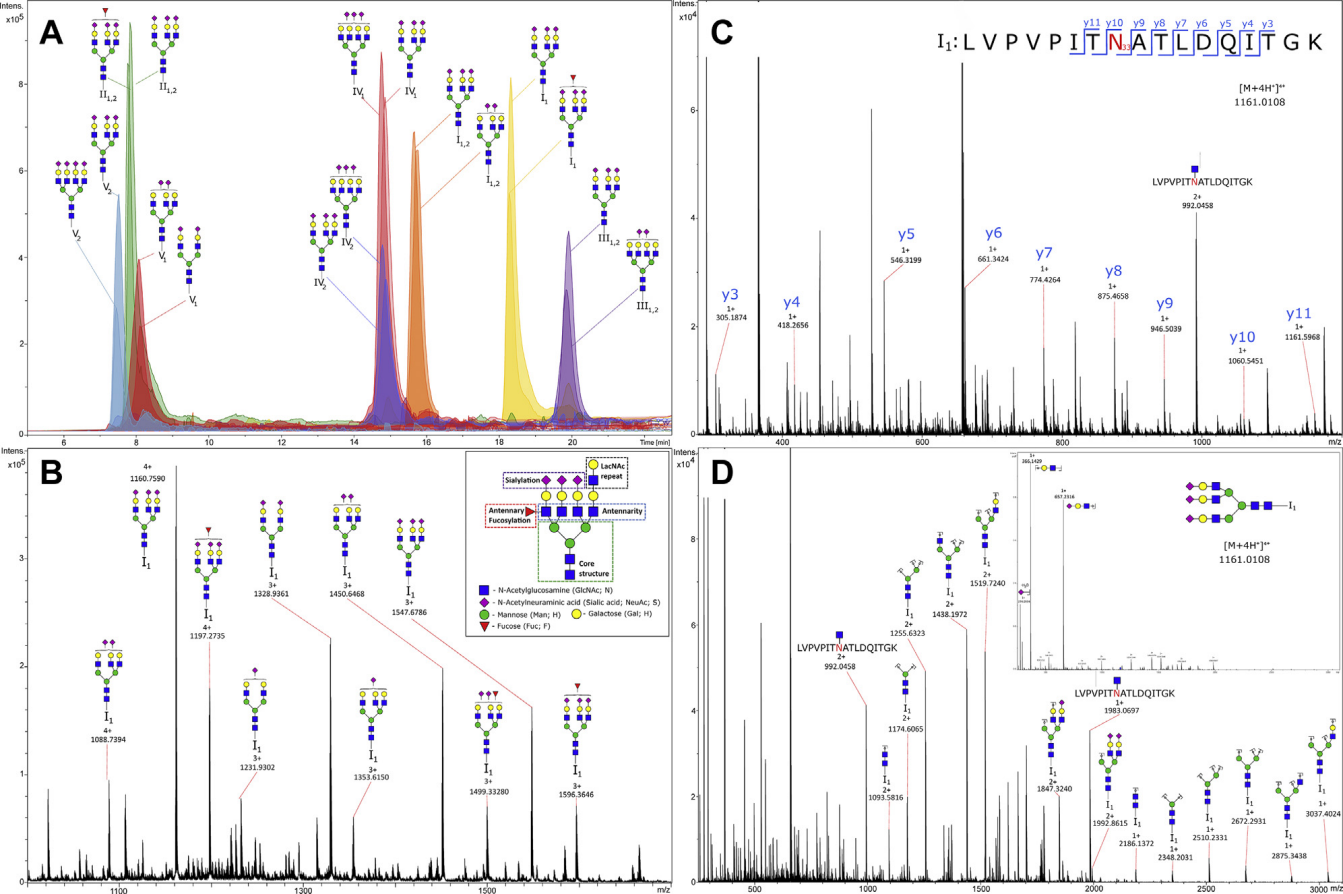
**Results of LC-MS AGP glycosylation analysis by the method.***A*, a typical chromatogram with extracted ion traces of the most abundant glycopeptides from each glycosylation site. TFA was used in the mobile phase as an ion-pairing agent. *B*, summed mass spectrum for I_1_ with the most abundant glycan structures annotated. *C*, MS/MS fragmentation spectrum for the peptide part of I_1_ N5H6S3. D, MS/MS fragmentation spectrum for the glycan part of I_1_ N5H6S3. AGP, alpha-1-acid glycoprotein.

Glycosylation analysis of AGP-enriched sample, which still contains a very complex mixture of number of different proteins, is possible by nanoLC-ESI-MS, where AGP-specific tryptic glycopeptides are identified according to their MS and MS/MS spectra. The biggest challenge in the development of the high-throughput LC method of such a complex sample was to obtain a good LC separation while maintaining the time of the gradient short enough to be able to analyze a cohort of hundreds or thousands of samples in a reasonable time frame. With three genes (*AGP-A*, *AGP-B*, and *AGP-B′*) encoding two protein forms (AGP1 and AGP2), AGP is a complex molecule which, despite five N-glycosylation sites, gives eight potential tryptic glycopeptides. The abbreviations used for naming the AGP glycopeptides are presented in Figure 2. Glycosylation sites were identified by m/z values calculated from the mass of each tryptic peptide carrying N-glycosylation site and the mass of previously reported glycan structures. A typical chromatogram with extracted ion traces of the most abundant glycopeptides from each glycosylation site is shown in Figure 3*A*, and an example summed MS spectrum for I_1_ is shown in Figure 3*B*. Supplemental Fig. S5 shows the chromatogram with extracted ion traces of the most abundant glycopeptides together with base peak intensity chromatogram and indicates a good chromatographic separation, except for the earliest eluting glycopeptides representing the II and V glycosylation sites, which are partially overlapping. The most abundant AGP glycopeptides were confirmed by MS/MS analysis, whereas the less abundant glycoforms were annotated according to retention times and mass differences of monosaccharides (see the Data processing subsection in the Experimental procedures section for more details). Fragmentation spectra were acquired by tuning energy stepping collision-induced dissociation of glycopeptides which enabled acquisition of glycan- and peptide-specific fragments within a single MS/MS spectra (55). Figure 3, *C* and *D* show example fragmentation spectra of I_1_ N5H6S3, for the peptide part and the glycan part, respectively. The MS/MS spectra for the rest of the glycosylation sites are shown in supplemental Fig. S2. Besides expected and previously reported peptide sequences covering AGP N-glycosylation sites (46, 47), we also observed additional peptides carrying glycosylation sites I and IV (supplemental Table S6). N-terminal glutamine in the sequences of the tryptic peptides leads to N-terminal loss of ammonia because of cyclization and formation of pyroglutamic acid. Those modified peptides, together with an additional truncated tryptic peptide from the glycosylation site I_1_ (CANLVPVPITNATLDQITGK), were significantly lower in measured intensities in comparison to their counterparts. Furthermore, they showed only a limited set of glycan structures that passed the quality control (QC) criteria. To avoid additional analytical bias in the glycoprofiling of the analyzed cohorts, only glycoforms originating from the most intense peptide from each of the glycosylation sites were used for the quantification.

All AGP glycopeptides specific for a glycosylation site were adequately separated by either LC part of the analytical system or by the difference in the m/z values. The only analytes partially coeluting in both dimensions were tryptic glycopeptides of the glycosylation site IV (N93) originating from AGP1 and (IV_1_) AGP2 (IV_2_). The two sequences of 15 amino acids are differing in only four amino acids which do not contribute enough to separate the peptides chromatographically in a targeted run time. Furthermore, the mass difference of the two tryptic peptides is only 5 Da which also caused partial overlap of isotopes of coeluting analytes. Because the main objective was to develop a high-throughput screening method, requiring a reasonable time for one single run, the glycopeptides derived from the IV_2_ tryptic peptide (1919.858 Da), which is beginning to overlap with the sixth isotope of the IV_1_ (1914.889 Da) peptide, were excluded from the quantification in the studied samples. For the quantification of the glycopeptides originating from IV_1_, only first six isotopes were used to minimize the confounding effect of possibly coeluting IV_2_ signal and are covering more than 90% of the total isotopic distribution of the measured analyte.

One of the concerns during the development of the method was a possible effect of the enrichment protocol on individual glycans, in terms of altering their relative abundances. More specifically, sialic acids which are prone to dissociation from the glycan structure when exposed to high temperatures and low pH could be lost during the procedure, subsequently altering the relative abundance of glycopeptides compared with the original sample (60). For this reason, an experiment for assessment of presence and level of mentioned effects was performed. Commercial AGP standard aliquots were prepared in two sets of quadruplicates. One set was treated with the enrichment protocol, while the other one was left untreated. Both sets were then processed identically for the remaining steps of reduction, alkylation, and trypsin digestion, as well as HILIC-SPE and LC-MS analysis. Finally, relative abundances of each detected glycopeptide within individual glycosylation sites were calculated and compared between sets. The results show a presence of minimal changes for the enrichment treated samples, with the loss of sialic acids not presenting an issue (supplemental Fig. S6). A short time of exposure to low pH, as well as the samples being kept at 5 °C for the most part during the protocol surely limit the desialylation process. Our results are in agreement with previous finding of Schultze *et al.* (61) who demonstrated that short exposure to perchloric acid at low temperatures does not result in desialylation of AGP glycans.

By applying the enrichment protocol, median CV of all extracted glycopeptides increased from 2% (interquartile range, IQR = 1–4%) to 8% (IQR = 5–13%). This means some variation was introduced by the procedure, but the repeatability of the method is still acceptable after the enrichment. Furthermore, we analyzed two series of pooled plasma standard replicates, which tested intraplate (10 replicates were randomized across one plate) and interplate (10 replicates were randomized across two different plates) repeatability (supplemental Table S7). Median CV was 10% (IQR = 8–15%) for the intraplate and 14% (IQR = 10–22%) for the interplate replicates, which shows that other proteins in a sample matrix do not notably increase variability of the method, but some batch effect is present, similar to other high-throughput glycosylation analysis methods. Therefore, batch effect correction was performed on all samples (for more information see Experimental procedures), which reduced the median interplate CV to 12% (IQR = 9–16%).

To determine the minimal amount of plasma from which a full site-specific AGP N-glycan profile can be obtained with the method, a sensitivity test was performed. AGP was enriched from 50, 20, 10, 5, 2.5, and 1 μl of pooled plasma standard, all in triplicates. AGP glycan profiles obtained from 50, 20, 10, and 5 μl of plasma were comparable, and all glycan structures passed the QC criteria, which was not the case with the profiles obtained from lower starting volumes. Profile obtained from 2.5 μl of plasma had much greater average CV and missed the third glycosylation site because of low signal intensities, whereas chromatograms obtained from starting volume of 1 μl were of insufficient quality to be processed (supplemental Fig. S7). Therefore, it can be concluded that at least 5 μl of human plasma is required to obtain a good AGP glycan profile with the method.

### AGP Site-Specific N-Glycosylation Profile of Pooled Plasma Standard

To identify the full-spectrum of N-glycan structures bound to AGP in the population for the stability and the pilot study, a plasma standard was created by aliquoting and pooling a small percentage of plasma from all samples in the cohorts. On all N-glycosylation sites, complex glycan structures were identified with almost exclusively highly branched and highly sialylated structures. For the quantification of the present glycoforms, seven N-glycopeptides were taken into consideration covering all five N-glycosylation sites and all genetic variants of AGP1 and AGP2 (supplemental Table S5). In total, 96 glycoforms were annotated and included for the quantification and further data analysis. Their relative abundances on each glycosylation site are presented in supplemental Fig. S8. Glycosylation site IV_1_ (N93) was the most diverse with 21 different glycoforms. On the other hand, only eight different glycan structures were found on the site II_1,2_ (N56). Glycosylation sites I_1_, I_1,2_, III_1,2_, V_1_, and V_2_ were annotated with 12, 12, 11, 15, and 17 glycan structures, respectively. A highly branched, triantennary glycan with three sialic acids (N5H6S3) was the most abundant glycoform present on the first glycosylation sites (N33) in both AGP1 and AGP2 (I_1,2_) (supplemental Fig. S2*B*). Glycopeptides carrying additional antennary fucose residue (N5H6S3F1) as well as triantennary, disialylated structures (N5H6S2) were also significantly present on both protein forms. Glycosylation site II_1,2_ (N56) was mostly covered with three glycans: triantennary glycans with three sialic acids, with and without antennary fucosylation (N5H6S3, N5H6S3F1) and diantennary disialylated structure (N4H5S2) (supplemental Fig. S2*D*). Glycosylation site III_1,2_ (N72) displayed relatively higher abundancy of highly sialylated structures, including the tetraantennary glycan with three sialic acids (N6H7S3) as the most abundant one. Peptides with most diverse glycan forms with 24 different structures were the ones originating from glycosylation site IV_1_ (N93). The site contained a variety of structures ranging from simpler diantennary glycans (N4H5S2) up to highly complex glycans with four sialic acids and poly-N-acetyllactosamine repeats (N7H8S4). The most abundant structures were triantennary and tetraantennary ones carrying up to four sialic acids. Glycosylation site V (N103) revealed, on average, a difference in glycosylation profiles of the two AGP forms. Most abundant glycans on V_1_ were triantenary and tetraantenary glycans with up to four sialic acids, whereas the glycosylation profile of V_2_ form was relatively dominated by tetraantennary glycans with up to four sialic acids and up to two fucose residues. Both V_1_ and V_2_ revealed highly complex glycans with poly-N-acetyllactosamine residues with up to four sialic acids and two fucose residues.

Although a larger repertoire of AGP site-specific N-glycopeptides (268 structures) were previously reported (48), they were detected by a method without AGP purification (commercial AGP was used) and no high-throughput potential.

### Derived Glycosylation Traits

All the samples in this study were analyzed at the glycopeptide level, generating a relatively large data set because of different glycan structures across seven different glycosylation sites, originating from different genetic variants of AGP1 and AGP2. Interpreting and understanding this, many individual pieces of information for each sample is challenging, so to simplify this information, glycans within each glycopeptide were grouped by their common structural and compositional characteristics. This makes interpretation and understanding of underlying biological information easier. Three major traits that were looked at included number of branches, presence of fucose, and number of bound sialic acids. Because sialic acids usually only bind to galactosylated branches, their number was shown as a ratio of sialic acids per galactose. Across seven glycosylation sites, a total number of 63 derived traits for the pilot study and 60 derived traits for the intraindividual temporal stability study were extracted, and their nomenclature is explained in Figure 2. The latter cohort is missing three derived traits because of a smaller number of glycopeptides which passed the quantification QC. Specific glycopeptides contributing to each derived trait can be found listed in supplemental Table S8.

### Intraindividual Temporal Stability Study

One of the unknowns about AGP glycosylation is its stability over time within an individual as well as its interindividual differences. To get insight into these factors and put them into perspective of what this newly developed method can measure, we devised another small experiment where samples of 14 healthy age and sex matched individuals were analyzed in three time points. The time gap between the samples was 6 and 4 weeks respectively, which considering the AGP half-life in plasma being of less than a week (62, 63), should ensure analysis of predominantly newly synthesized protein at each point.

Data obtained from this experiment were processed to calculate three categories of CV for each derived glycosylation trait. From longitudinal samples of each subject, the intraindividual CV was calculated. Interindividual CV was calculated from comparison of all subjects’ samples within one time point, whereas the eight random sample duplicates served to calculate the baseline CV of the method itself. The data (Table 1) show that the median of interindividual differences is above both intraindividual and duplicates variation across all derived traits, whereas the intraindividual variation is mostly comparable to duplicate variation or is between duplicate and interindividual variation. This is also shown with box plots in supplemental Fig. S9. As a precondition for a potential use as a biomarker, this way we were able to confirm that AGP glycosylation is mostly stable within a healthy individual and that its variation is smaller than the variation observed between different subjects. Furthermore, the fact that the subjects were age and sex matched allows us to observe interindividual differences where they are expected to be the smallest, so any additional variables should in theory further increase the difference between these two variations. Previous studies similarly confirmed intraindividual temporal stability of total plasma protein and IgG N-glycomes (64, 65), which further confirms that changes observed in glycan profiles are mainly consequence of environmental influences and physiologic responses, and those changes can further increase in some pathophysiological processes and therefore have a great diagnostic potential.

**Table 1:** Temporal stability of AGP glycosylation shown through medians and interquartile ranges for CVs of duplicates (variation of the method), CVs of intraindividual temporal variation, and interindividual differences across all derived traits

Derived trait	CVs of duplicates (%)	CVs of intraindividual temporal variation (%)	CVs of interindividual differences (%)
I_1_ BiAnt	2.2 (1.6–3.8)	5.5 (4–6.8)	10 (8.9–11.3)
I_1_ TriAnt	0.6 (0.5–1.3)	1.8 (1.1–2.2)	3 (2.7–3.5)
I_1_ F	1.9 (1.1–3)	6.2 (4.6–8.1)	25.2 (24.2–26.3)
I_1_ S1/1	0.6 (0.2–0.7)	1.6 (0.7–2)	1.6 (1.4–1.9)
I_1_ S2/3	0.5 (0.4–0.9)	2.3 (1.5–4)	4.2 (4–4.8)
I_1_ S1/2	7 (4.6–8.9)	9.4 (9.1–9.7)	17 (13.6–17.8)
I_1_ S1/3	5.5 (2.3–9.9)	7.8 (4–14.8)	11.4 (10.4–15.1)
I_1,2_ BiAnt	6.5 (4.2–9.6)	8.2 (7.5–13)	18.1 (14.8–18.5)
I_1,2_ TriAnt	1 (0.7–3.7)	2.4 (1.7–4.8)	5.4 (4.8–5.9)
I_1,2_ TetraAnt	17.9 (6.2–25)	23.8 (13.8–50.9)	49.2 (35.6–52.3)
I_1,2_ F	6.4 (3.9–8.7)	8.6 (6.2–16.6)	24.1 (20.8–25.1)
I_1,2_ S1/1	1.8 (1.2–3.5)	2.4 (1.7–6.4)	5.3 (4.5–6.7)
I_1,2_ S3/4	17.6 (3.9–26.9)	21.7 (12.9–51.5)	54.8 (39.4–57.2)
I_1,2_ S2/3	3 (1.5–4.7)	3.4 (3–6)	5.6 (5.4–6.8)
I_1,2_ S1/2	7.9 (6.5–14.6)	8.4 (5–14.6)	15.9 (12.8–22.9)
I_1,2_ S1/3	6.9 (3.3–7.7)	6.8 (3.3–10.1)	13.4 (12.7–13.9)
II_1,2_ BiAnt	0.3 (0.2–0.8)	2.1 (1.2–2.6)	7.2 (6.4–7.6)
II_1,2_ TriAnt	0.2 (0.1–0.3)	1.1 (0.8–1.6)	4.1 (3.9–4.4)
II_1,2_ TetraAnt	6.4 (2.5–11.9)	11.4 (8.8–23.3)	22.5 (22.2–26.4)
II_1,2_ F	0.4 (0.2–1.2)	4.3 (2.7–4.7)	20.4 (19.4–20.5)
II_1,2_ S1/1	0.2 (0.1–0.4)	0.7 (0.4–1.2)	2.1 (2.1–2.3)
II_1,2_ S3/4	6.4 (2.5–11.9)	11.4 (8.8–23.3)	22.5 (22.2–26.4)
II_1,2_ S2/3	1.6 (1.3–2.9)	3.6 (2.1–5.9)	13.5 (13.3–13.9)
II_1,2_ S1/2	7.1 (3.3–9.8)	6.9 (5.3–8.9)	10.5 (9.8–11.4)
II_1,2_ S1/3	6.6 (1.8–10.7)	11.1 (5.4–15)	17.4 (14.1–21.9)
III_1,2_ BiAnt	3 (1.9–4.3)	12.6 (6.9–17)	24.2 (19.6–25)
III_1,2_ TriAnt	1.1 (0.4–2)	2.1 (1.4–3.4)	5.4 (5.4–6)
III_1,2_ TetraAnt	1.7 (0.7–3.9)	4.7 (1.7–5.9)	10.3 (8.8–11)
III_1,2_ F	10 (3.3–11.6)	7.8 (4.7–10.9)	23.7 (20–23.9)
III_1,2_ S1/1	1.1 (0.8–1.5)	1.9 (1.3–2.5)	5.7 (5.3–6.4)
III_1,2_ S3/4	2.2 (0.9–4.2)	2.8 (2.1–4.3)	11.5 (10.7–12.4)
III_1,2_ S2/3	3 (1–5.3)	7.5 (2.8–10.6)	10.7 (8.7–11.1)
III_1,2_ S1/2	3.4 (1.3–5.1)	4.6 (3.5–7.4)	9.7 (9–11)
III_1,2_ S1/3	5.9 (2.2–10.9)	7.4 (4.8–10.5)	9.1 (8.7–10)
IV_1_ BiAnt	1.7 (1–3.4)	5.4 (4.2–6.4)	11.4 (9.8–11.9)
IV_1_ TriAnt	3.9 (1.8–6.3)	4 (2.7–5.7)	10.3 (9.9–10.9)
IV_1_ TetraAnt	2.1 (1.3–3.5)	2.3 (2.2–3.6)	7 (6.4–7.2)
IV_1_ F	2 (1.7–3.9)	5.3 (2.7–7.2)	21.2 (19.4–21.4)
IV_1_ S1/1	1.1 (0.4–1.6)	1.3 (0.7–2.4)	5.7 (5.4–5.8)
IV_1_ S3/4	1.7 (0.8–3.5)	2.3 (2.1–3.8)	7.6 (6.9–7.8)
IV_1_ S2/3	6.7 (2.7–8.6)	5.8 (3.3–7.3)	10.3 (9.8–10.5)
IV_1_ S1/2	2.6 (1.9–3.4)	4.2 (2.5–5.2)	10.2 (9.9–10.8)
IV_1_ S1/3	1.7 (0.9–3.3)	2.7 (1.7–3.8)	4.8 (4.5–4.9)
IV_1_ S1/4	6 (2.5–10.7)	7.4 (4.6–9.4)	19.9 (16.5–20.1)
V_1_ TriAnt	3.6 (1.6–5.6)	5.1 (3.2–5.9)	9.3 (9.2–11.1)
V_1_ TetraAnt	1.6 (0.6–3)	2.1 (1.5–2.9)	4.5 (4.3–5.2)
V_1_ F	1.2 (0.7–2.2)	2.5 (1.7–3.9)	14.4 (13.9–14.8)
V_1_ S1/1	1.1 (0.3–1.3)	1.1 (0.6–1.7)	3.1 (3–3.2)
V_1_ S3/4	0.3 (0.2–0.6)	1.4 (1.2–2.3)	7.6 (7.4–8.2)
V_1_ S2/3	7.1 (1.8–9.8)	6.1 (4.7–11.1)	13.4 (11.7–14.3)
V_1_ S1/2	5.5 (3.4–12.1)	7.7 (3.6–10.1)	16.2 (15.3–16.6)
V_2_ BiAnt	3.2 (2.6–6.6)	10.9 (9.6–12.1)	30.4 (25.8–31.6)
V_2_ TriAnt	3.4 (1.5–5.1)	4.4 (1.5–6.9)	9.9 (9.5–11.3)
V_2_ TetraAnt	1.2 (0.7–2.3)	1.7 (0.8–2.8)	4.5 (4.2–5)
V_2_ F	1.6 (0.3–2.6)	3.9 (2–5.3)	20.3 (19.1–20.4)
V_2_ S1/1	1.4 (0.9–1.9)	1.4 (0.7–1.8)	2.2 (2.1–2.4)
V_2_ S3/4	1.6 (1.1–1.9)	2.1 (1.7–3)	6.6 (6.3–6.9)
V_2_ S2/3	5.2 (2–7.9)	5.6 (2.2–8.9)	13.2 (12.8–14.1)
V_2_ S1/2	6.5 (4.2–9.1)	8.5 (5.7–9.8)	11.3 (11–13)
V_2_ S1/3	5.5 (2.6–8.1)	4.9 (2.7–8.5)	10.8 (10–10.9)
**All derived traits**	**2.4 (1.4–6.1)**	**4.7 (2.3–7.5)**	**10.4 (5.7–17.1)**

AGP, alpha-1-acid glycoprotein; CV, coefficient of variation.

The samples were measured from 14 healthy and age-matched male students, at three time points (0, 6, and 10 weeks) for each student. A total number of eight random sample duplicates (across all three time points) served to calculate the baseline CV of the method itself. From longitudinal samples of each subject, the intraindividual CV was calculated, whereas the interindividual CV was calculated from all subjects’ samples within each time point. The statistically significant derived glycan traits were marked with bold.

### Pilot Study—Individuals With and Without Hyperglycemia During Critical Illness

**Fig. 4:**
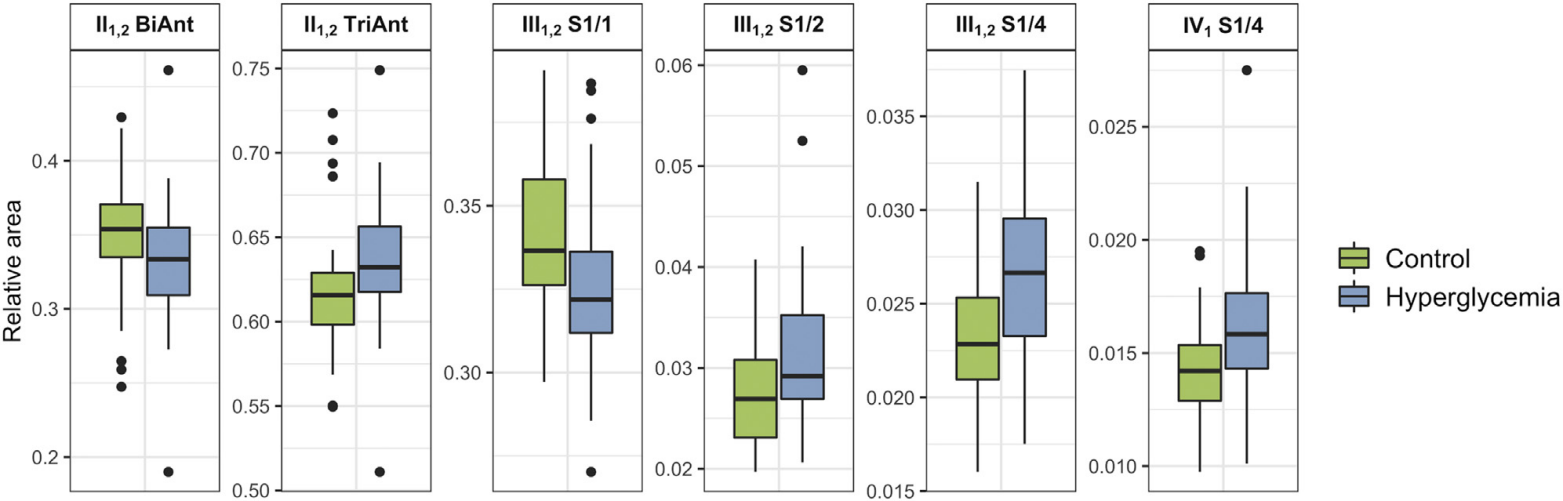
**Box plots showing significant changes in derived AGP glycosylation traits for individuals with and without (controls) hyperglycemia during critical illness.** AGP, alpha-1-acid glycoprotein.

As a pilot study we decided to apply our method on a population including subjects with registered hyperglycemia in critical illness, which represents an increased risk for type 2 diabetes development (relative risk in 5-year period for patients with hyperglycemia is around 5 (51, 52)). A total number of 108 subjects aged between 18 and 79 years were included in the study (supplemental Table S1). Samples were analyzed with the newly developed method and processed as described in Materials and methods. The results were presented as a total of 63 derived glycan trait groups for each sample (supplemental Table S9). Logistic regression analysis showed six glycan groups that were significantly changed in patients with registered hyperglycemia compared with the control group (Table 2, Fig. 4). II_1,2_ glycosylation site showed shift toward more antennae as abundance of biantennary glycans decreased (BiAnt −0.726, *p* = 0.0376) and abundance of triantennary glycans increased (TriAnt 0.791, *p* = 0.0329). Next, changes on III_1,2_ glycosylation site included a decrease in fully sialylated glycans (S1/1 −0.965, *p* = 0.0218), an increase in glycans with half of potential sialic acids present (S1/2 0.720, *p* = 0.0376) as well as increase in glycans with only one quarter of potential sialic acids present (S1/4 0.893, *p* = 0.0218). Finally, IV_1_ glycosylation site showed an increase in glycans with one quarter of potential sialylations present (S1/4 0.780, *p* = 0.0329). It is interesting to note that apart from the changes observed on glycosylation site II_1,2_, derived traits describing branching on other sites, even though not significantly changed, still show similar trends toward increase in glycan branching (Table 2).

**Table 2:** Calculated effects of hyperglycemia during critical illness on levels of individual derived AGP glycosylation traits and their respective unadjusted and adjusted *p*-values

Derived trait	Coefficent (B)	*p*-value	Adj. *p*-value
I_1_ BiAnt	−0.336	0.179	0.388
I_1_ TriAnt	0.314	0.211	0.429
I_1_ TetraAnt	0.133	0.558	0.718
I_1_ F	0.055	0.822	0.893
I_1_ S1/1	−0.273	0.241	0.463
I_1_ S3/4	0.076	0.742	0.835
I_1_ S2/3	0.314	0.185	0.388
I_1_ S1/2	−0.113	0.650	0.799
I_1_ S1/3	0.275	0.251	0.464
I_1,2_ BiAnt	−0.369	0.090	0.320
I_1,2_ TriAnt	0.213	0.317	0.525
I_1,2_ TetraAnt	0.549	0.014	0.107
I_1,2_ F	−0.141	0.515	0.705
I_1,2_ S1/1	−0.298	0.159	0.386
I_1,2_ S3/4	0.491	0.025	0.131
I_1,2_ S2/3	0.177	0.403	0.609
I_1,2_ S1/2	−0.005	0.979	0.979
I_1,2_ S1/3	0.194	0.365	0.575
**II**_**1,2**_**BiAnt**	**−0.726**	**0.004**	**0.038**
**II**_**1,2**_**TriAnt**	**0.791**	**0.002**	**0.037**
II_1,2_ TetraAnt	0.302	0.159	0.386
II_1,2_ F	0.026	0.904	0.918
II_1,2_ S1/1	−0.148	0.487	0.682
II_1,2_ S3/4	0.302	0.159	0.386
II_1,2_ S2/3	0.307	0.168	0.388
II_1,2_ S1/2	−0.482	0.034	0.152
II_1,2_ S1/3	0.129	0.541	0.711
III_1,2_ BiAnt	−0.575	0.023	0.130
III_1,2_ TriAnt	0.177	0.416	0.609
III_1,2_ TetraAnt	0.183	0.432	0.618
III_1,2_ F	0.093	0.674	0.799
**III**_**1,2**_**S1/1**	**−0.965**	**0.001**	**0.022**
III_1,2_ S3/4	0.252	0.284	0.497
III_1,2_ S2/3	0.090	0.676	0.799
**III**_**1,2**_**S1/2**	**0.720**	**0.004**	**0.038**
III_1,2_ S1/3	0.329	0.129	0.370
**III**_**1,2**_**S1/4**	**0.893**	**0.001**	**0.022**
IV_1_ BiAnt	−0.245	0.264	0.475
IV_1_ TriAnt	−0.378	0.105	0.320
IV_1_ TetraAnt	0.386	0.105	0.320
IV_1_ F	−0.058	0.792	0.875
IV_1_ S1/1	−0.593	0.016	0.115
IV_1_ S3/4	0.293	0.183	0.388
IV_1_ S2/3	−0.111	0.613	0.772
IV_1_ S1/2	0.566	0.021	0.129
IV_1_ S1/3	0.086	0.685	0.799
**IV**_**1**_**S1/4**	**0.767**	**0.002**	**0.037**
V_1_ TriAnt	−0.365	0.100	0.320
V_1_ TetraAnt	0.358	0.107	0.320
V_1_ F	−0.031	0.886	0.916
V_1_ S1/1	−0.182	0.408	0.609
V_1_ S3/4	0.399	0.079	0.313
V_1_ S2/3	−0.197	0.361	0.575
V_1_ S1/2	0.265	0.242	0.463
V_2_ BiAnt	−0.634	0.011	0.099
V_2_ TriAnt	−0.484	0.040	0.170
V_2_ TetraAnt	0.515	0.031	0.152
V_2_ F	−0.084	0.699	0.801
V_2_ S1/1	−0.038	0.858	0.901
V_2_ S3/4	0.222	0.308	0.525
V_2_ S2/3	−0.319	0.142	0.386
V_2_ S1/2	0.137	0.532	0.711
V_2_ S1/3	0.038	0.853	0.901

AGP, alpha-1-acid glycoprotein.

A total number of 108 subjects were included in the study (59 who developed hyperglycemia during critical illness *versus* 49 controls), and each subject was sampled once. Associations between AGP glycopeptide derived traits and hyperglycemia were analyzed using logistic regression with age and sex included as additional covariates for each derived glycan trait separately. The glycan trait variables were transformed to standard normal distribution. Coefficient (B) corresponds to natural logarithm of odds ratio, which always corresponds to change of one standard deviation for any given variable in the calculated glycan trait. Considering multiple tests performed, Benjamini-Hochberg method was used to control the false discovery rate. The *p*-value <0.05 was considered significant. The statistically significant derived glycan traits were marked with bold.

To investigate a potential prediction power of AGP glycans for the predisposition to type 2 diabetes, we tried to build a glycan-based discriminative model using a logistic regression model. In the discriminative model, only six statistically significant derived glycan traits were used as predictors. The models were assessed using a receiver operating characteristic curve analysis (supplemental Fig. S10). While a model based on age and sex did not show significant discriminative power (AUC = 0.54), addition of glycan variables into the model increased its discriminative power considerably (AUC = 0.80). A model based on clinical parameters alone (age, sex, BMI, and family history of diabetes) presented some modest discriminative power (AUC = 0.65); however, when glycans were included in the model, the discriminative power increased again to same level as with the model based on age, sex, and glycans (AUC = 0.80). This indicates that these six glycan traits, when combined, have a solid predictive power for the predisposition to type 2 diabetes, although this should be confirmed in prospective studies with a larger sample size. Furthermore, although BMI and family history of diabetes present some modest predictive power, they do not improve the prediction when they are added to the model with the glycans.

As it can be seen from the results of the logistic regression analysis, AGP glycosylation changes were more pronounced or present on some glycosylation sites and genetic variants, while less noticeable or absent on the other. This demonstrates the importance of site-specific AGP glycosylation information which can be obtained with our method and which could be important for the elucidation of AGP’s function in the future.

Recently, we have measured total plasma protein N-glycome, which is comprised of different glycans originating from many different glycoproteins, in the same population (47). There we have shown that increased branching and complexity of plasma N-glycan structures is associated with a higher risk of developing type 2 diabetes and poorer regulation of blood glucose levels. However, it was not possible to find the real cause and significance of that change because measured glycan levels in total plasma protein N-glycome are product of both composition of glycans, as well as the relative abundance of those proteins. Here, we show that the observed change is at least in part caused by increased branching of AGP glycans, and these effects are even larger in the AGP N-glycome than those observed in the total plasma N-glycome. Increased branching of AGP glycans was shown in many chronic inflammatory conditions (26, 31, 32, 33, 34). Likewise, previous studies showed that multibranched N-glycans were also elevated in total plasma protein N-glycome in response to chronic inflammatory diseases (66, 67, 68). Therefore, the higher branching of plasma N-glycome could be generally caused by higher AGP glycan branching in response to chronic inflammation. Furthermore, it is also well known that individuals with the metabolic syndrome and type 2 diabetes suffer from chronic low-grade inflammation (69). Thus, the changes we observed in AGP N-glycans may also reflect the start of a subtle chronic inflammation and the susceptibility for developing a metabolic syndrome.

On the other hand, the observed relative decrease in sialylation in AGP glycans presents a new finding, because this effect was not observed in the total plasma N-glycome and was probably masked by other plasma proteins’ glycans. To the best of our knowledge, the relative decrease in AGP sialyation was not reported before in any pathophysiological condition and could be specific for a predisposition to type 2 diabetes. If this is confirmed in larger longitudinal prospective studies, it will help to develop specific glycopeptide-level diagnostic marker for early detection of increased type 2 diabetes development risk.

No significant associations between type 2 diabetes and AGP glycosylation were reported previously (50), but higher AGP levels were found in obese mice and humans with metabolic syndrome and type 2 diabetes (70, 71, 72). It is possible that AGP could function through leptin receptor to regulate food intake and energy homeostasis in response to nutrition status (70). Furthermore, some suggest that AGP is induced selectively in the adipose tissue of obese mice to suppress excess inflammation and to protect adipose tissue from excessive inflammation and thereby from metabolic dysfunction (73). Therefore, it is easy to perceive that AGP could have an important function in response to metabolic dysfunction which may lead to diabetes type 2. As a molecule which is 45% composed of glycans, it is highly unlikely that the glycan part might not contribute to this function. The specificity of the interaction between AGP and its potential receptors may be modulated by the glycan moiety and therefore may change whenever its glycosylation changes.

## Conclusion

Here, we present a cost-effective method for a high-throughput and site-specific N-glycosylation LC-MS analysis of AGP on a glycopeptide level, which offers a detailed analysis of AGP glycosylation in hundreds of plasma samples in 1 week. It does not require isolation of AGP with antibodies and affinity chromatography, but AGP is analyzed directly from the “seromucoid” fraction obtained from a small amount (≥5 μl) of human plasma.

Previously, it was shown that AGP glycosylation is altered in many different pathophysiological conditions and the observed glycan changes appear to be specific for some diseases. Therefore, AGP glycosylation has a great potential for new, disease-specific biomarker discovery, and our method provides a new valuable tool for its research.

We used the method to show for the first time that AGP glycosylation is temporally stable within a healthy individual and that its variation is smaller than the variation observed between different age and sex matched subjects, which is a precondition for its potential use as a biomarker.

Furthermore, we tested the method on a population including individuals with an increased risk of developing type 2 diabetes. The individuals at higher risk of diabetes presented increased AGP N-glycan branching and lower sialylation on some glycosylation sites compared with the controls. This demonstrates the importance of the site-specific AGP glycosylation information which can be obtained with the presented method and which could be important for the elucidation of AGP’s function in the future. The observed relative decrease in sialylation in AGP glycans, to the best of our knowledge, was not reported before in any pathophysiological condition and could be specific for a predisposition to type 2 diabetes. Even though this should be additionally confirmed in larger longitudinal prospective studies, it indicates that site-specific AGP N-glycan profile could help distinguish individuals who are at risk of type 2 diabetes development.

Although many possible functions of AGP have been described, the detailed knowledge about its exact role in pathophysiological processes is still elusive. A large part of AGP is modified by glycans, so they certainly play a very important role in its biological activity. Various possible functions of AGP could be a consequence of its numerous different glycoforms, which may exert diverse effects. Thus, site-specific information about AGP N-glycosylation changes caused by many different physiological conditions, diseases, and environmental influences could help with the elucidation of AGP’s role in health and disease.

## Data Availability

MaxQuant data and all raw data files are publicly available on the PRIDE archive (http://www.ebi.ac.uk/pride) under the identifier: PXD020387.

## Conflict of interest

G. L. is the founder and owner and M. N. is an employee of Genos Ltd, a company that specializes in high-throughput glycomics and has several patents in this field. The remaining authors declare that the research was conducted in the absence of any commercial or financial relationships that could be construed as a potential conflict of interest.
