# Supplementary material for: High-Throughput and Site-Specific N-Glycosylation Analysis of Human Alpha-1-Acid Glycoprotein Offers a Great Potential for New Biomarker Discovery
Source: Mol Cell Proteomics. 2021 Jan 23;20:100044. doi: 10.1074/mcp.RA120.002433 (PMC7950198; doi:10.1074/mcp.RA120.002433)
Supplement: Supplemental Figures and Tables [file mmc1.pdf]

Supplemental Data

High-throughput and site-specific N-glycosylation analysis of human alpha-1-acid glycoprotein offers a great potential for new biomarker discovery

Toma Keser, Marko Tijardović, Ivan Gornik, Edita Lukić, Gordan Lauc, Olga Gornik, Mislav Novokmet

This PDF file includes:

Supplemental Tables 1 to 9

Supplemental Figures 1 to 10

Supplemental Tables

Table S1: Descriptive data of the pilot study (individuals with and without hyperglycemia during critical illness).

	HG during acute illness	No HG during acute illness	*p* -value (HG vs. no HG)*
Number of patients (N)	59	49	
Age (y)	58 (25 - 79)	54 (18 - 75)	0.388
Female sex (N,%)	18 (30.5%)	14 (28.6%)	0.826
BMI (kg/m2)	24.2±4.1	23.1±3.9	0.734
Family history of diabetes (N,%)	19 (32.2%)	8 (16.3%)	0.039
* p-values were calculated using Mann–Whitney U test (significance level α = 0.05) and for categorical variables χ^2 test was used			
HG – Hyperglycaemia			

Table S2: MaxQuant analysis of the enriched AGP fraction following c18 enrichment protocol (1st worksheet) and HILIC enrichment protocol (2nd worksheet). Reference proteome UPID: UP000005640. Available as a separate file.

Table S3: Automatically annotated spectra based on MS/MS data for 87 glycopeptides identified by Byonic search engine.

Glycosylation site	Glycan composition	Sequence	Charge state	Obs. m/z	Calc. m/z	ppm error	Scan Time	Scan #
I_1	N4H5S1	-.LVPVPITN[+1913.67702]ATLDQITGK.W	3	1231.9246	1231.9044	16.45	17.2376	scan=1021
I_1	N4H5S2	-.LVPVPITN[+2204.77244]ATLDQITGK.W	3	1328.9595	1328.9362	17.55	17.2213	scan=1020
I_1	N4H5S2F1	-.LVPVPITN[+2350.83035]ATLDQITGK.W	3	1377.6343	1377.6221	8.83	17.7884	scan=1044
I_1	N5H6S1	-.LVPVPITN[+2278.80922]ATLDQITGK.W	3	1353.6154	1353.6151	0.26	17.8978	scan=1051
I_1	N5H6S1F1	-.LVPVPITN[+2424.86713]ATLDQITGK.W	3	1402.3231	1402.3011	15.74	17.124	scan=1014
I_1	N5H6S2	-.LVPVPITN[+2569.90464]ATLDQITGK.W	3	1450.6691	1450.6469	15.34	17.0429	scan=1009
I_1	N5H6S2F1	-.LVPVPITN[+2715.96255]ATLDQITGK.W	3	1499.3555	1499.3329	15.08	16.9941	scan=1006
I_1	N5H6S3	-.LVPVPITN[+2861.00005]ATLDQITGK.W	3	1547.7026	1547.6787	15.48	16.9776	scan=1005
I_1	N5H6S3F1	-.LVPVPITN[+3007.05796]ATLDQITGK.W	3	1596.3876	1596.3647	14.37	16.9624	scan=1004
I_1	N6H7S2	-.LVPVPITN[+2935.03683]ATLDQITGK.W	3	1572.3823	1572.3576	15.68	16.9301	scan=1002
I_1	N6H7S3	-.LVPVPITN[+3226.13225]ATLDQITGK.W	3	1669.4001	1669.3894	6.4	17.6621	scan=1037
$I_{1,2}$	N4H5S1	-.LVPVPITN[+1913.67702]ATLDR.I	3	1108.187	1108.1691	16.16	15.0064	scan=888
$I_{1,2}$	N4H5S2	-.LVPVPITN[+2204.77244]ATLDR.I	4	904.1653	904.1525	14.1	14.8638	scan=879
$I_{1,2}$	N5H6S1	-.LVPVPITN[+2278.80922]ATLDR.I	3	1229.8967	1229.8799	13.68	14.7836	scan=874
$I_{1,2}$	N5H6S2	-.LVPVPITN[+2569.90464]ATLDR.I	3	1326.9336	1326.9117	16.52	14.7672	scan=873
$I_{1,2}$	N5H6S2F1	-.LVPVPITN[+2715.96255]ATLDR.I	3	1375.6184	1375.5976	15.11	14.8003	scan=875
$I_{1,2}$	N5H6S3	-.LVPVPITN[+2861.00005]ATLDR.I	3	1423.966	1423.9435	15.84	14.533	scan=858
$I_{1,2}$	N5H6S3F1	-.LVPVPITN[+3007.05796]ATLDR.I	3	1472.6523	1472.6295	15.55	14.5911	scan=862
$I_{1,2}$	N6H7S2	-.LVPVPITN[+2935.03683]ATLDR.I	3	1448.6407	1448.6224	12.62	14.4672	scan=854
$I_{1,2}$	N6H7S2F1	-.LVPVPITN[+3081.09474]ATLDR.I	3	1497.3129	1497.3084	3.05	14.8617	scan=872
$I_{1,2}$	N6H7S3	-.LVPVPITN[+3226.13225]ATLDR.I	3	1545.6762	1545.6542	14.25	14.5495	scan=859
$I_{1,2}$	N6H7S3F1	-.LVPVPITN[+3372.19016]ATLDR.I	3	1594.3575	1594.3402	10.86	14.4505	scan=853
$II_{1,2}$	N4H5S1	R.NEEYN[+1913.67702]K.S	3	904.0185	904.0129	6.18	7.9951	scan=470
$II_{1,2}$	N4H5S2	R.NEEYN[+2204.77244]K.S	3	1001.0548	1001.0447	10.06	7.8336	scan=460
$II_{1,2}$	N4H5S2F1	R.NEEYN[+2350.83035]K.S	3	1049.7284	1049.7307	-2.15	8.1288	scan=473
$II_{1,2}$	N5H6S1	R.NEEYN[+2278.80922]K.S	3	1025.7216	1025.7236	-1.98	8.2111	scan=478
$II_{1,2}$	N5H6S2	R.NEEYN[+2569.90464]K.S	3	1122.765	1122.7555	8.55	7.817	scan=459
$II_{1,2}$	N5H6S3	R.NEEYN[+2861.00005]K.S	3	1219.8008	1219.7873	11.1	7.8007	scan=458
$II_{1,2}$	N5H6S3F1	R.NEEYN[+3007.05796]K.S	3	1268.4885	1268.4732	12.01	7.7098	scan=452
$II_{1,2}$	N6H7S3	R.NEEYN[+3226.13225]K.S	3	1341.4965	1341.498	-1.14	7.9605	scan=463
$III_{1,2}$	N4H5S2	K.SVQEIQATFFYFTPN[+2204.77244]K.T	3	1375.6059	1375.5803	18.64	18.7391	scan=1110
$III_{1,2}$	N5H6S1	K.SVQEIQATFFYFTPN[+2278.80922]K.T	3	1400.2855	1400.2592	18.78	18.6885	scan=1107
$III_{1,2}$	N5H6S2	K.SVQEIQATFFYFTPN[+2569.90464]K.T	3	1497.3172	1497.291	17.5	18.6044	scan=1102
$III_{1,2}$	N5H6S2F1	K.SVQEIQATFFYFTPN[+2715.96255]K.T	3	1545.9912	1545.977	9.19	19.2822	scan=1133
$III_{1,2}$	N5H6S3	K.SVQEIQATFFYFTPN[+2861.00005]K.T	3	1594.3514	1594.3228	17.92	18.5371	scan=1098
$III_{1,2}$	N5H6S3F1	K.SVQEIQATFFYFTPN[+3007.05796]K.T	4	1232.5175	1232.5084	7.35	19.2149	scan=1129
$III_{1,2}$	N6H7S1	K.SVQEIQATFFYFTPN[+2643.94142]K.T	3	1521.9953	1521.9699	16.71	19.2422	scan=1136
$III_{1,2}$	N6H7S2	K.SVQEIQATFFYFTPN[+2935.03683]K.T	3	1619.0298	1619.0017	17.33	18.4024	scan=1090
$III_{1,2}$	N6H7S3	K.SVQEIQATFFYFTPN[+3226.13225]K.T	3	1716.0479	1716.0335	8.39	19.1145	scan=1123
$III_{1,2}$	N6H7S3F1	K.SVQEIQATFFYFTPN[+3372.19016]K.T	4	1323.8138	1323.7914	16.88	18.301	scan=1084
$III_{1,2}$	N6H7S4	K.SVQEIQATFFYFTPN[+3517.22767]K.T	3	1813.0813	1813.0653	8.83	19.0133	scan=1117
IV_1	N5H6S1	R.QDQC[+57.02146]IYN[+2278.80922]TTYLNVQR.E	3	1398.9263	1398.9068	13.93	14.0114	scan=827
IV_1	N5H6S2	R.QDQC[+57.02146]IYN[+2569.90464]TTYLNVQR.E	4	1122.2199	1122.2058	12.61	13.9784	scan=825
IV_1	N5H6S2F1	R.QDQC[+57.02146]IYN[+2715.96255]TTYLNVQR.E	3	1544.6239	1544.6246	-0.44	14.3541	scan=842
IV_1	N5H6S3	R.QDQC[+57.02146]IYN[+2861.00005]TTYLNVQR.E	3	1592.9926	1592.9704	13.9	13.9129	scan=821
IV_1	N5H6S3F1	R.QDQC[+57.02146]IYN[+3007.05796]TTYLNVQR.E	4	1231.5073	1231.4941	10.73	13.7816	scan=813
IV_1	N6H7S1	R.QDQC[+57.02146]IYN[+2643.94142]TTYLNVQR.E	3	1520.6279	1520.6176	6.81	14.4845	scan=854
IV_1	N6H7S2	R.QDQC[+57.02146]IYN[+2935.03683]TTYLNVQR.E	3	1617.6755	1617.6494	16.19	13.7981	scan=814
IV_1	N6H7S2F1	R.QDQC[+57.02146]IYN[+3081.09474]TTYLNVQR.E	4	1250.0115	1250.0033	6.59	14.3691	scan=847
IV_1	N6H7S3	R.QDQC[+57.02146]IYN[+3226.13225]TTYLNVQR.E	3	1714.6791	1714.6812	-1.2	14.1922	scan=832
IV_1	N6H7S3F1	R.QDQC[+57.02146]IYN[+3372.19016]TTYLNVQR.E	3	1763.3642	1763.3671	-1.67	14.2081	scan=833
IV_1	N6H7S4	R.QDQC[+57.02146]IYN[+3517.22767]TTYLNVQR.E	3	1811.7112	1811.713	-0.99	14.16	scan=830
IV_1	N6H7S4F1	R.QDQC[+57.02146]IYN[+3663.28558]TTYLNVQR.E	4	1395.5721	1395.551	15.11	13.6679	scan=806
IV_1	N6H7S4F2	R.QDQC[+57.02146]IYN[+3809.34348]TTYLNVQR.E	4	1432.0885	1432.0655	16.07	14.2568	scan=840
IV_1	N7H8S2	R.QDQC[+57.02146]IYN[+3300.16903]TTYLNVQR.E	3	1739.3646	1739.3601	2.59	14.0617	scan=824
IV_1	N7H8S3	R.QDQC[+57.02146]IYN[+3591.26445]TTYLNVQR.E	4	1377.5675	1377.5457	15.81	13.5355	scan=798
IV_1	N7H8S3F1	R.QDQC[+57.02146]IYN[+3737.32236]TTYLNVQR.E	3	1885.0803	1885.0779	1.28	14.011	scan=821
IV_1	N7H8S4	R.QDQC[+57.02146]IYN[+3882.35986]TTYLNVQR.E	4	1450.3394	1450.3196	13.64	13.3856	scan=789
IV_1	N8H9S3	R.QDQC[+57.02146]IYN[+3956.39664]TTYLNVQR.E	4	1468.8525	1468.8288	16.14	13.4022	scan=790
V_1	N5H6S2	R.EN[+2569.90464]GTISR.Y	3	1116.1121	1116.103	8.22	8.2712	scan=486
V_1	N5H6S3	R.EN[+2861.00005]GTISR.Y	3	1213.1348	1213.1348	-0.02	8.1609	scan=475
V_1	N5H6S3F1	R.EN[+3007.05796]GTISR.Y	3	1261.8256	1261.8207	3.87	8.1635	scan=479
V_1	N6H7S3	R.EN[+3226.13225]GTISR.Y	3	1334.8527	1334.8455	5.38	8.7708	scan=511
V_1	N6H7S3F1	R.EN[+3372.19016]GTISR.Y	3	1383.5386	1383.5315	5.16	8.1186	scan=476
V_1	N6H7S4	R.EN[+3517.22767]GTISR.Y	3	1431.8836	1431.8773	4.38	8.0293	scan=470
V_1	N6H7S4F1	R.EN[+3663.28558]GTISR.Y	3	1480.5679	1480.5633	3.14	8.0589	scan=472
V_1	N6H7S4F2	R.EN[+3809.34348]GTISR.Y	3	1529.2534	1529.2492	2.75	7.9877	scan=465
V_1	N7H8S3	R.EN[+3591.26445]GTISR.Y	3	1456.5617	1456.5562	3.76	7.9541	scan=463
V_1	N7H8S3F1	R.EN[+3737.32236]GTISR.Y	3	1505.2473	1505.2422	3.42	7.9209	scan=461
V_1	N7H8S4	R.EN[+3882.35986]GTISR.Y	3	1553.5991	1553.588	7.1	7.9827	scan=467
V_1	N8H9S4	R.EN[+4247.49206]GTISR.Y	3	1675.313	1675.2988	8.5	7.9332	scan=464
V_2	N4H5S2	R.EN[+2204.77244]GTVSR.Y	3	989.7267	989.7204	6.4	7.7869	scan=453
V_2	N5H6S1	R.EN[+2278.80922]GTVSR.Y	3	1014.4065	1014.3993	7.16	7.8868	scan=459
V_2	N5H6S2	R.EN[+2569.90464]GTVSR.Y	3	1111.4422	1111.4311	10.01	7.7905	scan=455
V_2	N5H6S2F1	R.EN[+2715.96255]GTVSR.Y	3	1160.128	1160.1171	9.44	7.8067	scan=456
V_2	N5H6S3	R.EN[+2861.00005]GTVSR.Y	3	1208.4631	1208.4629	0.18	7.8762	scan=458
V_2	N5H6S3F1	R.EN[+3007.05796]GTVSR.Y	3	1257.1614	1257.1489	10	7.7295	scan=451
V_2	N6H7S2	R.EN[+2935.03683]GTVSR.Y	3	1233.1505	1233.1418	7.05	7.7363	scan=450
V_2	N6H7S2F1	R.EN[+3081.09474]GTVSR.Y	3	1281.835	1281.8278	5.66	7.7529	scan=451
V_2	N6H7S3	R.EN[+3226.13225]GTVSR.Y	3	1330.1873	1330.1736	10.28	7.744	scan=452
V_2	N6H7S3F1	R.EN[+3372.19016]GTVSR.Y	3	1378.8635	1378.8596	2.87	7.7749	scan=452
V_2	N6H7S4	R.EN[+3517.22767]GTVSR.Y	3	1427.2186	1427.2054	9.24	7.6569	scan=446
V_2	N6H7S4F1	R.EN[+3663.28558]GTVSR.Y	3	1475.9078	1475.8914	11.15	7.5984	scan=442
V_2	N6H7S4F2	R.EN[+3809.34348]GTVSR.Y	3	1524.5844	1524.5774	4.59	7.708	scan=448
V_2	N7H8S4F1	R.EN[+4028.41777]GTVSR.Y	3	1597.6188	1597.6021	10.41	7.6307	scan=444
V_2	N8H9S4	R.EN[+4247.49206]GTVSR.Y	4	1253.2329	1253.222	8.74	7.5396	scan=438
V_2	N8H9S4F1	R.EN[+4393.54997]GTVSR.Y	3	1719.334	1719.3129	12.33	7.548	scan=439

Table S4: Manually annotated spectra based on MS data only. The annotation was done according to following criteria: same retention time window (coelution) as confirmed AGP glycopeptides, m/z value matched to internal database, presence of more than one charge state.

Glycosylation site	Glycan composition	Sequence	Obs. m/z 2+	Calc. m/z 2+	ppm err. 2+	Obs. m/z 3+	Calc. m/z 3+	ppm err. 3+	Obs. m/z 4+	Calc. m/z 4+	ppm err. 4+	Scan Time
I_1	N5H5S2	LVPVPITNATLDQITGK	2094.3844	2094.4403	-26.69	1396.6019	1396.62927	-19.60	1047.7188	1047.72377	-4.74	17.1
$I_{1,2}$	N5H5S2	LVPVPITNATLDR	1908.7936	1908.83742	-22.96	1272.8759	1272.894037	-14.25	954.9296	954.922345	7.60	14.8
IV_1	N4H5S2	QDQCIYNTTYLNVQR	2060.7987	2060.83817	-19.15	1374.2076	1374.22787	-14.75	/	1030.92272	n.a.	13.6
IV_1	N5H6S1F1	QDQCIYNTTYLNVQR	2170.8429	2170.88552	-19.63	1447.5692	1447.59277	-16.28	1085.9423	1085.946395	-3.77	14.0
IV_1	N6H7S1F1	QDQCIYNTTYLNVQR	/	2353.45162	n.a.	1569.2771	1569.303503	-16.82	1177.2159	1177.229445	-11.51	13.9
V_1	N6H7S2F1	ENGTISR	/	965.126545	n.a.	1286.4787	1286.499637	-16.27	1929.2153	1929.24582	-15.82	8.1
V_1	N8H9S3	ENGTISR	2366.8619	2366.89677	-14.73	1578.2382	1578.266937	-18.21	1183.938	1183.95202	-11.84	7.9
V_1	N8H9S4F1	ENGTISR	/	2585.47342	n.a.	1723.9506	1723.984703	-19.78	1293.214	1293.240345	-20.37	7.9
V_2	N5H6S3F2	ENGTVSR	1958.2134	1958.24857	-17.96	1305.8157	1305.834803	-14.63	/	979.62792	n.a.	7.6

Table S5: Glycopeptides which were taken for the quantification, covering all 5 N-glycosylation sites and genetic variants of AGP1 and AGP2 (except IV2). Monoisotopic masses that were used for the quantitation, along with the corresponding QC values are shown in bold. Available as a separate file.

Table S6. List of peptide sequences carrying AGP N-glycosylation sites observed in the pooled plasma standard. Sequences in gray are detected but were not used for the glycopeptide quantification in the analyzed cohorts. PyroQ represents a modified peptide with N-terminally cyclized glutamine.

N-glycosylation site	AGP 1 Uniprot Accesion No. P02763	AGP 2 Uniprot Accesion No. P19652
I	LVPVPIT**N**ATLDQITGK	LVPVPIT**N**ATLDR
	CANLVPVPIT**N**ATLDQITGK	CANLVPVPIT**N**ATLDR
	pyroQIPLCANLVPVPIT**N**ATLDQITGK	pyroQIPLCANLVPVPIT**N**ATLDR
II	NEEY**N**K	
III	SVQEIQATFFYFTP**N**K	
IV	QDQCIY**N**TTYLNVQR	QNQCFY**N**SSYLNVQR
	pyroQDQCIY**N**TTYLNVQR	pyroQNQCFY**N**SSYLNVQR
V	E**N**GTISR	E**N**GTVSR

Table S7: The repeatability of measurement of glycopeptides reported in the pilot study.

			CVs of untreated AGP standard (%)	CVs of treated AGP standard (%)	CVs of intra-plate plasma pool standard (%)	CVs of inter-plate plasma pool standard (%)	CVs of inter-plate plasma pool standard after BC (%)
		No. of replicates	4	4	10	10	10
		Q1 CV	1.4	5.3	7.8	9.7	8.5
		**Median CV**	**2.0**	**8.0**	**10.2**	**14.1**	**11.9**
		Q3 CV	3.6	12.9	15.0	21.6	16.4
**Glycosylation site**	**Glycan composition**						
I_1	N4H5S1		4.3	18.7	9.7	21.2	13.8
I_1	N4H5S2		1.6	8.8	11.4	13.1	14.4
I_1	N4H5S2F1		/	/	6.3	8.4	10.5
I_1	N5H5S2		3.1	38.8	8.3	10.3	10.0
I_1	N5H6S1		7.5	35.7	8.3	8.3	13.2
I_1	N5H6S1F1		/	/	12.1	17.6	17.9
I_1	N5H6S2		2.7	7.9	10.8	12.9	13.4
I_1	N5H6S2F1		2.6	5.2	18.6	27.9	21.2
I_1	N5H6S3		1.2	4.1	9.1	9.8	12.3
I_1	N5H6S3F1		2.0	3.7	6.5	6.4	6.5
I_1	N6H7S2		6.3	19.9	45.9	71.9	42.9
I_1	N6H7S3		4.3	16.0	11.4	14.2	12.2
$I_{1,2}$	N4H5S1		1.8	23.7	7.8	19.1	8.1
$I_{1,2}$	N4H5S2		2.0	14.0	9.0	16.9	9.5
$I_{1,2}$	N5H5S2		/	/	7.2	7.9	8.1
$I_{1,2}$	N5H6S1		4.9	21.8	9.9	11.3	11.8
$I_{1,2}$	N5H6S2		2.0	10.9	5.2	5.0	5.5
$I_{1,2}$	N5H6S2F1		2.9	16.6	4.2	9.5	7.0
$I_{1,2}$	N5H6S3		0.9	6.3	3.1	3.6	6.5
$I_{1,2}$	N5H6S3F1		1.2	8.4	5.8	11.1	5.8
$I_{1,2}$	N6H7S2		/	/	23.9	36.0	21.3
$I_{1,2}$	N6H7S2F1		4.7	37.6	19.9	34.1	18.6
$I_{1,2}$	N6H7S3		1.9	27.7	11.5	11.8	12.1
$I_{1,2}$	N6H7S3F1		/	/	36.1	61.6	35.6
$II_{1,2}$	N4H5S1		10.0	12.0	16.0	21.4	16.8
$II_{1,2}$	N4H5S2		5.2	2.7	13.5	17.7	15.1
$II_{1,2}$	N4H5S2F1		0.4	4.7	9.4	11.2	9.7
$II_{1,2}$	N5H6S1		7.5	15.4	9.0	9.0	10.5
$II_{1,2}$	N5H6S2		3.1	9.8	4.9	5.8	6.0
$II_{1,2}$	N5H6S3		1.2	2.3	3.2	4.7	5.3
$II_{1,2}$	N5H6S3F1		0.9	2.9	13.1	17.9	12.8
$II_{1,2}$	N6H7S3		/	/	20.4	31.9	19.4
$III_{1,2}$	N4H5S2		5.7	22.3	9.6	9.3	9.3
$III_{1,2}$	N5H6S1		6.3	26.5	8.2	11.6	9.1
$III_{1,2}$	N5H6S2		5.6	13.3	12.6	14.0	12.1
$III_{1,2}$	N5H6S2F1		4.7	9.8	10.4	16.6	12.4
$III_{1,2}$	N5H6S3		0.3	6.8	13.1	34.8	14.5
$III_{1,2}$	N5H6S3F1		1.5	8.9	15.0	19.4	15.2
$III_{1,2}$	N6H7S1		4.1	32.6	16.8	18.5	16.3
$III_{1,2}$	N6H7S2		1.6	7.1	26.6	44.2	27.5
$III_{1,2}$	N6H7S3		2.9	3.5	6.4	11.9	6.4
$III_{1,2}$	N6H7S3F1		3.2	10.3	8.0	12.4	8.7
$III_{1,2}$	N6H7S4		5.5	9.9	13.6	13.9	13.3
IV_1	N4H5S2		3.1	6.3	10.8	11.1	11.7
IV_1	N5H6S1		/	/	8.7	9.0	9.1
IV_1	N5H6S1F1		/	/	7.5	16.5	10.1
IV_1	N5H6S2		2.5	7.5	14.0	17.1	14.0
IV_1	N5H6S2F1		1.9	6.1	8.0	9.7	9.0
IV_1	N5H6S3		0.9	4.0	11.6	17.0	11.5
IV_1	N5H6S3F1		1.2	7.7	7.8	9.8	8.1
IV_1	N6H7S1		3.8	11.2	18.5	27.6	17.6
IV_1	N6H7S1F1		/	/	31.4	49.3	30.7
IV_1	N6H7S2		1.5	3.4	6.2	10.4	6.7
IV_1	N6H7S2F1		0.7	4.2	4.0	3.8	4.3
IV_1	N6H7S3		1.3	7.0	2.1	2.6	2.3
IV_1	N6H7S3F1		1.5	1.3	6.0	7.8	7.5
IV_1	N6H7S4		2.7	8.0	7.2	11.0	6.8
IV_1	N6H7S4F1		1.6	5.1	9.4	15.0	12.8
IV_1	N6H7S4F2		1.0	6.7	11.8	22.2	17.1
IV_1	N7H8S2		2.4	12.8	20.6	24.2	20.2
IV_1	N7H8S3		1.6	18.3	19.7	24.3	18.5
IV_1	N7H8S3F1		2.1	16.3	23.9	33.3	22.9
IV_1	N7H8S4		1.2	22.3	23.9	33.9	21.5
IV_1	N8H9S3		2.4	19.8	22.1	31.7	20.8
V_1	N5H6S2		3.1	12.2	21.5	30.2	21.4
V_1	N5H6S3		1.1	5.3	25.0	32.5	26.0
V_1	N5H6S3F1		1.6	4.4	14.1	20.0	14.0
V_1	N6H7S2F1		5.5	15.5	7.7	7.7	8.6
V_1	N6H7S3		1.8	6.8	11.7	12.6	12.6
V_1	N6H7S3F1		1.9	9.0	7.8	9.9	10.1
V_1	N6H7S4		1.3	6.7	13.0	15.2	13.5
V_1	N6H7S4F1		1.5	6.4	12.7	22.2	15.8
V_1	N6H7S4F2		1.4	3.0	15.2	26.7	18.0
V_1	N7H8S3		1.0	7.4	17.2	23.9	16.0
V_1	N7H8S3F1		2.7	7.8	18.3	28.6	17.1
V_1	N7H8S4		0.8	2.7	25.3	41.3	24.8
V_1	N8H9S3		1.5	3.5	11.8	15.5	13.6
V_1	N8H9S4		2.6	7.9	20.4	23.6	25.3
V_1	N8H9S4F1		2.5	13.0	15.4	18.3	17.3
V_2	N4H5S2		1.7	6.9	7.9	17.4	7.6
V_2	N5H6S1		1.6	9.1	10.2	12.7	10.7
V_2	N5H6S2		4.0	10.2	12.7	18.7	12.6
V_2	N5H6S2F1		4.1	9.5	7.8	12.9	8.5
V_2	N5H6S3		1.6	4.1	10.2	16.1	10.6
V_2	N5H6S3F1		1.4	1.2	7.6	10.8	7.2
V_2	N5H6S3F2		0.3	7.0	5.0	5.6	7.4
V_2	N6H7S2		5.3	10.8	8.2	8.8	8.9
V_2	N6H7S2F1		/	/	9.4	9.6	10.5
V_2	N6H7S3		4.8	6.7	4.0	3.9	4.1
V_2	N6H7S3F1		4.7	10.7	8.0	10.4	9.7
V_2	N6H7S4		1.0	4.4	4.2	5.6	4.6
V_2	N6H7S4F1		0.6	5.6	5.0	8.5	6.0
V_2	N6H7S4F2		2.2	9.5	9.4	15.4	10.3
V_2	N7H8S4F1		2.3	3.3	10.6	14.5	11.8
V_2	N8H9S4		1.6	7.4	4.2	5.5	6.8
V_2	N8H9S4F1		0.9	8.9	9.2	9.5	8.5

Table S8: Formulas for calculating derived AGP glycosylation traits.

Derived trait	Formula
I1 BiAnt	I1 N4H5S1 + I1 N4H5S2 + I1 N4H5S2F1
I1 TriAnt	I1 N5H5S2 + I1 N5H6S1 + I1 N5H6S2 + I1 N5H6S3 + I1 N5H6S1F1 + I1 N5H6S2F1 + I1 N5H6S3F1
I1 TetraAnt	I1 N6H7S2 + I1 N6H7S3
I1 F	1*(I1 N4H5S2F1 + I1 N5H6S1F1 + I1 N5H6S2F1 + I1 N5H6S3F1) / (I1 N4H5S1 + I1 N4H5S2 + I1 N5H5S2 + I1 N5H6S1 + I1 N5H6S2 + I1 N5H6S3 + I1 N6H7S2 + I1 N6H7S3 + I1 N4H5S2F1 + I1 N5H6S1F1 + I1 N5H6S2F1 + I1 N5H6S3F1)
I1 S1/1	I1 N4H5S2 + I1 N4H5S2F1 + I1 N5H5S2 + I1 N5H6S3 + I1 N5H6S3F1
I1 S1/2	I1 N4H5S1 + I1 N6H7S2
I1 S1/3	I1 N5H6S1 + I1 N5H6S1F1
I1 S2/3	I1 N5H6S2 + I1 N5H6S2F1
I1 S3/4	I1 N6H7S3
I1,2 BiAnt	I1,2 N4H5S1 + I1,2 N4H5S2
I1,2 TriAnt	I1,2 N5H5S2 + I1,2 N5H6S1 + I1,2 N5H6S2 + I1,2 N5H6S3 + I1,2 N5H6S2F1 + I1,2 N5H6S3F1
I1,2 TetraAnt	I1,2 N6H7S2 + I1,2 N6H7S3 + I1,2 N6H7S2F1 + I1,2 N6H7S3F1
I1,2 F	1*(I1,2 N5H6S2F1 + I1,2 N5H6S3F1 + I1,2 N6H7S2F1 + I1,2 N6H7S3F1) / (I1,2 N4H5S1 + I1,2 N4H5S2 + I1,2 N5H5S2 + I1,2 N5H6S1 + I1,2 N5H6S2 + I1,2 N5H6S3 + I1,2 N6H7S2 + I1,2 N6H7S3 + I1,2 N5H6S2F1 + I1,2 N5H6S3F1 + I1,2 N6H7S2F1 + I1,2 N6H7S3F1)
I1,2 S1/1	I1,2 N4H5S2 + I1,2 N5H5S2 + I1,2 N5H6S3 + I1,2 N5H6S3F1
I1,2 S1/2	I1,2 N4H5S1 + I1,2 N6H7S2 + I1,2 N6H7S2F1
I1,2 S1/3	I1,2 N5H6S1
I1,2 S2/3	I1,2 N5H6S2 + I1,2 N5H6S2F1
I1,2 S3/4	I1,2 N6H7S3 + I1,2 N6H7S3F1
II1,2 BiAnt	II1,2 N4H5S1 + II1,2 N4H5S2 + II1,2 N4H5S2F1
II1,2 TriAnt	II1,2 N5H6S1 + II1,2 N5H6S2 + II1,2 N5H6S3 + II1,2 N5H6S3F1
II1,2 TetraAnt	II1,2 N6H7S3
II1,2 F	1*(II1,2 N4H5S2F1 + II1,2 N5H6S3F1) / (II1,2 N4H5S1 + II1,2 N4H5S2 + II1,2 N5H6S1 + II1,2 N5H6S2 + II1,2 N5H6S3 + II1,2 N6H7S3 + II1,2 N4H5S2F1 + II1,2 N5H6S3F1)
II1,2 S1/1	II1,2 N4H5S2 + II1,2 N4H5S2F1 + II1,2 N5H6S3 + II1,2 N5H6S3F1
II1,2 S1/2	II1,2 N4H5S1
II1,2 S1/3	II1,2 N5H6S1
II1,2 S2/3	II1,2 N5H6S2
II1,2 S3/4	II1,2 N6H7S3
III1,2 BiAnt	III1,2 N4H5S2
III1,2 TriAnt	III1,2 N5H6S1 + III1,2 N5H6S2 + III1,2 N5H6S3 + III1,2 N5H6S2F1 + III1,2 N5H6S3F1
III1,2 TetraAnt	III1,2 N6H7S1 + III1,2 N6H7S2 + III1,2 N6H7S3 + III1,2 N6H7S4 + III1,2 N6H7S3F1
III1,2 F	1*(III1,2 N5H6S2F1 + III1,2 N5H6S3F1 + III1,2 N6H7S3F1) / (III1,2 N4H5S2 + III1,2 N5H6S1 + III1,2 N5H6S2 + III1,2 N5H6S3 + III1,2 N6H7S1 + III1,2 N6H7S2 + III1,2 N6H7S3 + III1,2 N6H7S4 + III1,2 N5H6S2F1 + III1,2 N5H6S3F1 + III1,2 N6H7S3F1)
III1,2 S1/1	III1,2 N4H5S2 + III1,2 N5H6S3 + III1,2 N5H6S3F1 + III1,2 N6H7S4
III1,2 S1/2	III1,2 N6H7S2
III1,2 S1/3	III1,2 N5H6S1
III1,2 S2/3	III1,2 N5H6S2 + III1,2 N5H6S2F1
III1,2 S1/4	III1,2 N6H7S1
III1,2 S3/4	III1,2 N6H7S3 + III1,2 N6H7S3F1
IV1 BiAnt	IV1 N4H5S2
IV1 TriAnt	IV1 N5H6S1 + IV1 N5H6S2 + IV1 N5H6S3 + IV1 N5H6S1F1 + IV1 N5H6S2F1 + IV1 N5H6S3F1
IV1 TetraAnt	IV1 N6H7S1 + IV1 N6H7S2 + IV1 N6H7S3 + IV1 N6H7S4 + IV1 N6H7S1F1 + IV1 N6H7S2F1 + IV1 N6H7S3F1 + IV1 N6H7S4F1 + IV1 N6H7S4F2 + IV1 N7H8S2 + IV1 N7H8S3 + IV1 N7H8S4 + IV1 N7H8S3F1 + IV1 N8H9S3
IV1 F	(1*(IV1 N5H6S1F1 + IV1 N5H6S2F1 + IV1 N5H6S3F1 + IV1 N6H7S1F1 + IV1 N6H7S2F1 + IV1 N6H7S3F1 + IV1 N6H7S4F1 + IV1 N7H8S3F1) + 2*(IV1 N6H7S4F2)) / (IV1 N4H5S2 + IV1 N5H6S1 + IV1 N5H6S2 + IV1 N5H6S3 + IV1 N6H7S1 + IV1 N6H7S2 + IV1 N6H7S3 + IV1 N6H7S4 + IV1 N7H8S2 + IV1 N7H8S3 + IV1 N7H8S4 + IV1 N8H9S3 + IV1 N5H6S1F1 + IV1 N5H6S2F1 + IV1 N5H6S3F1 + IV1 N6H7S1F1 + IV1 N6H7S2F1 + IV1 N6H7S3F1 + IV1 N6H7S4F1 + IV1 N7H8S3F1 + IV1 N6H7S4F2)
IV1 S1/1	IV1 N4H5S2 + IV1 N5H6S3 + IV1 N5H6S3F1 + IV1 N6H7S4 + IV1 N6H7S4F1 + IV1 N6H7S4F2 + IV1 N7H8S4
IV1 S1/2	IV1 N6H7S2 + IV1 N6H7S2F1 + IV1 N7H8S2
IV1 S1/3	IV1 N5H6S1 + IV1 N5H6S1F1
IV1 S2/3	IV1 N5H6S2 + IV1 N5H6S2F1
IV1 S1/4	IV1 N6H7S1 + IV1 N6H7S1F1
IV1 S3/4	IV1 N6H7S3 + IV1 N6H7S3F1 + IV1 N7H8S3 + IV1 N7H8S3F1 + IV1 N8H9S3
V1 TriAnt	V1 N5H6S2 + V1 N5H6S3 + V1 N5H6S3F1
V1 TetraAnt	V1 N6H7S3 + V1 N6H7S4 + V1 N6H7S2F1 + V1 N6H7S3F1 + V1 N6H7S4F1 + V1 N6H7S4F2 + V1 N7H8S3 + V1 N7H8S4 + V1 N7H8S3F1 + V1 N8H9S3 + V1 N8H9S4 + V1 N8H9S4F1
V1 F	(1*(V1 N5H6S3F1 + V1 N6H7S2F1 + V1 N6H7S3F1 + V1 N6H7S4F1 + V1 N7H8S3F1 + V1 N8H9S4F1) + 2*(V1 N6H7S4F2)) / (V1 N5H6S2 + V1 N5H6S3 + V1 N6H7S3 + V1 N6H7S4 + V1 N7H8S3 + V1 N7H8S4 + V1 N8H9S3 + V1 N8H9S4 + V1 N5H6S3F1 + V1 N6H7S2F1 + V1 N6H7S3F1 + V1 N6H7S4F1 + V1 N7H8S3F1 + V1 N8H9S4F1 + V1 N6H7S2F1)
V1 S1/1	V1 N5H6S3 + V1 N5H6S3F1 + V1 N6H7S4 + V1 N6H7S4F1 + V1 N6H7S4F2 + V1 N7H8S4 + V1 N8H9S4 + V1 N8H9S4F1
V1 S1/2	V1 N6H7S2F1
V1 S2/3	V1 N5H6S2
V1 S3/4	V1 N6H7S3 + V1 N6H7S3F1 + V1 N7H8S3 + V1 N7H8S3F1 + V1 N8H9S3
V2 BiAnt	V2 N4H5S2
V2 TriAnt	V2 N5H6S1 + V2 N5H6S2 + V2 N5H6S3 + V2 N5H6S2F1 + V2 N5H6S3F1 + V2 N5H6S3F2
V2 TetraAnt	V2 N6H7S2 + V2 N6H7S3 + V2 N6H7S4 + V2 N6H7S2F1 + V2 N6H7S3F1 + V2 N6H7S4F1 + V2 N6H7S4F2 + V2 N7H8S4F1 + V2 N8H9S4 + V2 N8H9S4F1
V2 F	(1*(V2 N5H6S2F1 + V2 N5H6S3F1 + V2 N6H7S2F1 + V2 N6H7S3F1 + V2 N6H7S4F1 + V2 N7H8S4F1 + V2 N8H9S4F1) + 2*(V2 N5H6S3F2 + V2 N6H7S4F2)) / (V2 N4H5S2 + V2 N5H6S1 + V2 N5H6S2 + V2 N5H6S3 + V2 N6H7S2 + V2 N6H7S3 + V2 N6H7S4 + V2 N8H9S4 + V2 N5H6S2F1 + V2 N5H6S3F1 + V2 N6H7S2F1 + V2 N6H7S3F1 + V2 N6H7S4F1 + V2 N7H8S4F1 + V2 N8H9S4F1 + V2 N5H6S3F2 + V2 N6H7S4F2)
V2 S1/1	V2 N4H5S2 + V2 N5H6S3 + V2 N5H6S3F1 + V2 N5H6S3F2 + V2 N6H7S4 + V2 N6H7S4F1 + V2 N6H7S4F2 + V2 N7H8S4F1 + V2 N8H9S4 + V2 N8H9S4F1
V2 S1/2	V2 N6H7S2 + V2 N6H7S2F1
V2 S1/3	V2 N5H6S1
V2 S2/3	V2 N5H6S2 + V2 N5H6S2F1
V2 S3/4	V2 N6H7S3 + V2 N6H7S3F1

Table S9: Clinical data and calculated values for derived AGP glycosylation traits for every sample used in the pilot study. Missing values in the I1 glycosylation site are result of genetic variations where patients do not possess F1 or F2 AGP1 variant. The missing values in the $III_{1,2}$ glycosylation site are result of unsuccessful quantification due to low signal intensity. Available as a separate file.

Supplemental Figures

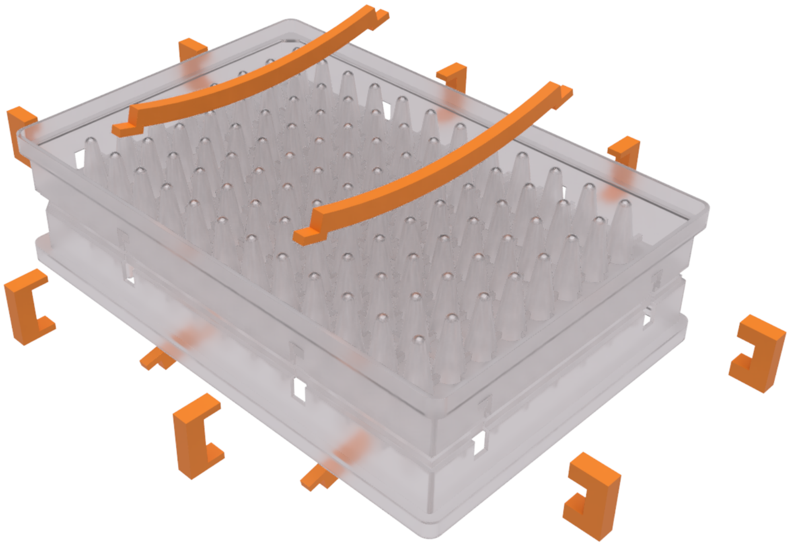

Figure S1: In-house 3D printed adapters for high-throughput supernatant transfer.

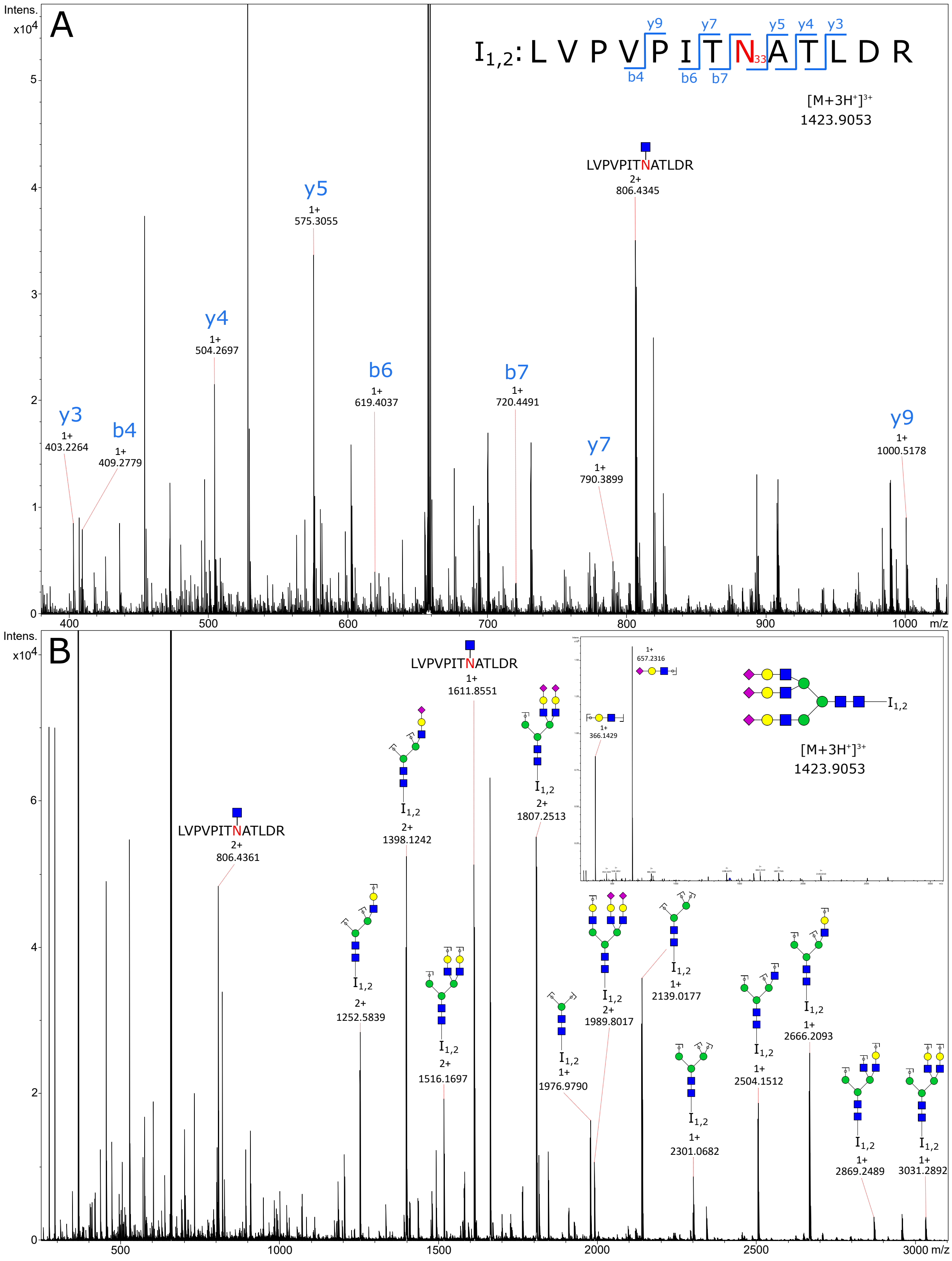

A
Intens.
x10^4
I1,2: L V P V P I T N33 A T L D R
y9 y7 y5 y4 y3
b4 b6 b7
[M+3H+]3+
1423.9053
LVPVPITNATLDR
2+
806.4345
y5
1+
575.3055
y4
1+
504.2697
b6
1+
619.4037
b7
1+
720.4491
y3
1+
403.2264
b4
1+
409.2779
y7
1+
790.3899
y9
1+
1000.5178
m/z
B
Intens.
x10^4
LVPVPITNATLDR
1+
1611.8551
LVPVPITNATLDR
2+
806.4361
I1,2
2+
1398.1242
I1,2
2+
1807.2513
1+
657.2316
1+
366.1429
I1,2
[M+3H+]3+
1423.9053
I1,2
2+
1252.5839
I1,2
2+
1516.1697
I1,2
1+
1976.9790
I1,2
2+
1989.8017
I1,2
1+
2139.0177
I1,2
1+
2301.0682
I1,2
1+
2504.1512
I1,2
1+
2666.2093
I1,2
1+
2869.2489
I1,2
1+
3031.2892
m/z

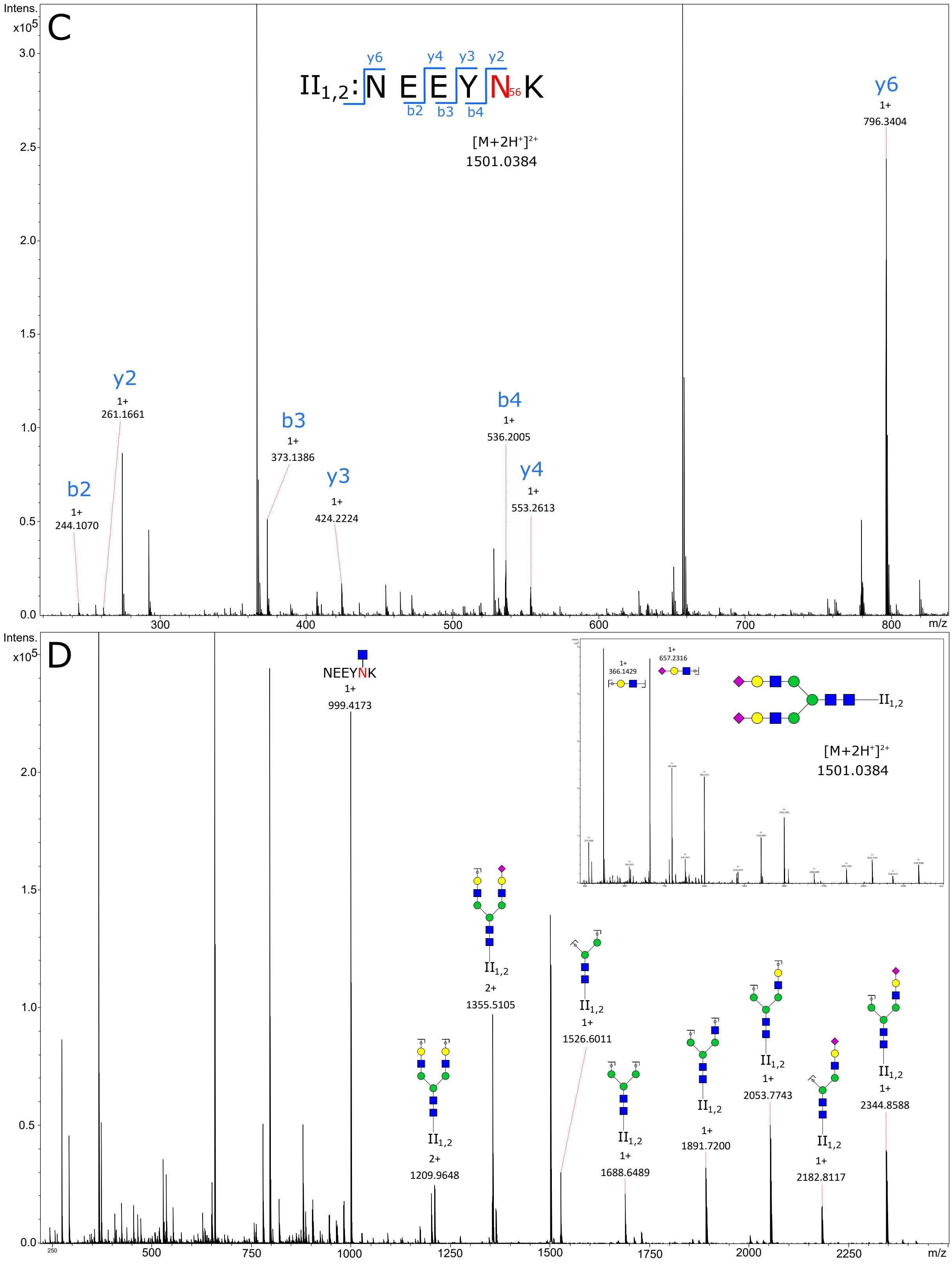
C
Intens. x10^5
II1,2: N E E Y N56 K
y6 y4 y3 y2
b2 b3 b4
[M+2H+]2+
1501.0384
y6 1+ 796.3404
y2 1+ 261.1661
b2 1+ 244.1070
b3 1+ 373.1386
y3 1+ 424.2224
b4 1+ 536.2005
y4 1+ 553.2613
m/z
D
Intens. x10^5
NEEYNK 1+ 999.4173
1+ 366.1429
1+ 657.2316
II1,2
[M+2H+]2+
1501.0384
II1,2 2+ 1355.5105
II1,2 1+ 1526.6011
II1,2 2+ 1209.9648
II1,2 1+ 1688.6489
II1,2 1+ 1891.7200
II1,2 1+ 2053.7743
II1,2 1+ 2182.8117
II1,2 1+ 2344.8588
m/z

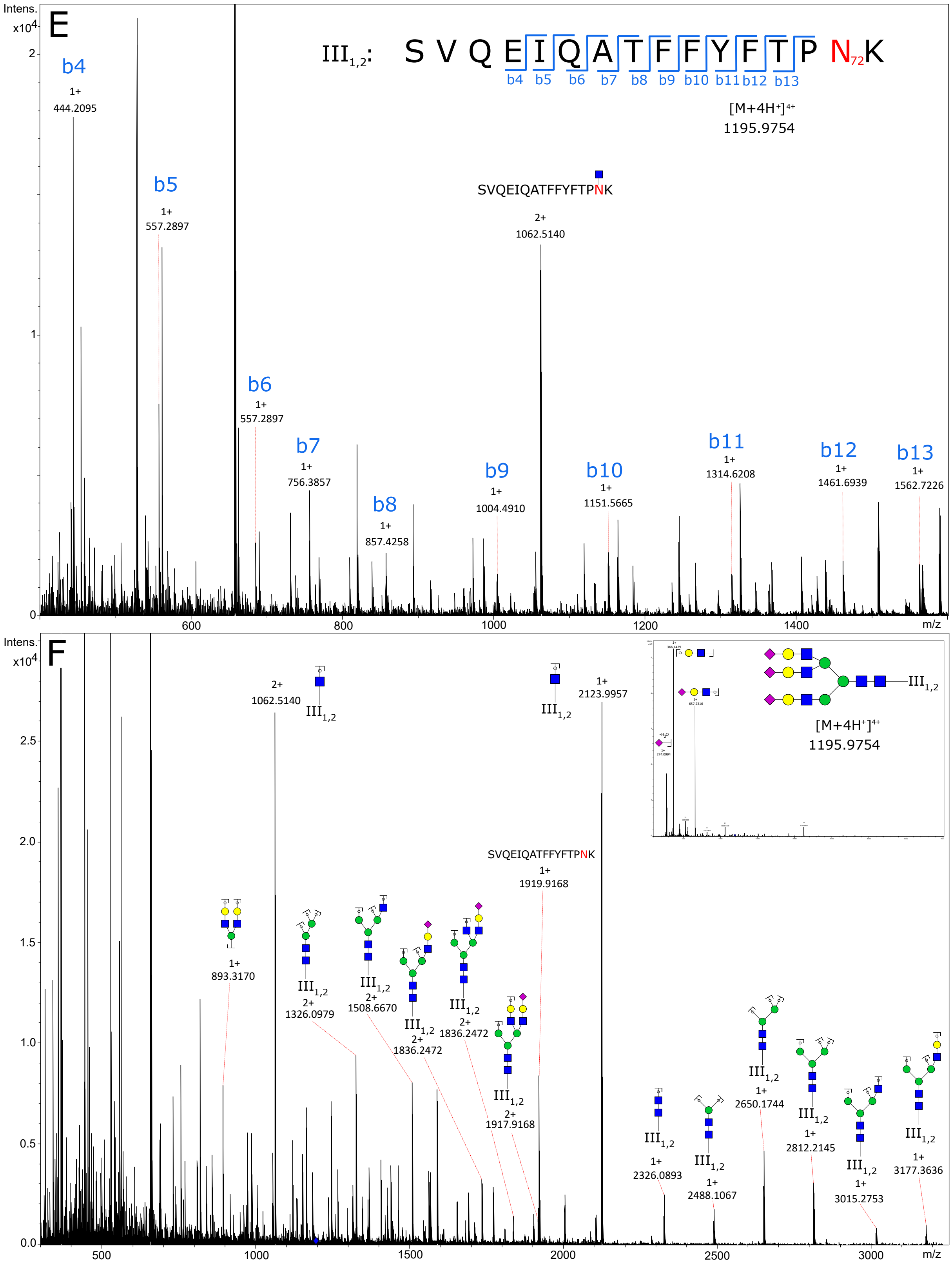
E
Intens. x10^4
III1,2: SVQEIQATFFYFTPN72K
b4 b5 b6 b7 b8 b9 b10 b11 b12 b13
[M+4H+]4+
1195.9754
b4 1+ 444.2095
b5 1+ 557.2897
SVQEIQATFFYFTPNK 2+ 1062.5140
b6 1+ 557.2897
b7 1+ 756.3857
b8 1+ 857.4258
b9 1+ 1004.4910
b10 1+ 1151.5665
b11 1+ 1314.6208
b12 1+ 1461.6939
b13 1+ 1562.7226
m/z
F
Intens. x10^4
2+ 1062.5140 III1,2
III1,2 1+ 2123.9957
III1,2
[M+4H+]4+
1195.9754
SVQEIQATFFYFTPNK 1+ 1919.9168
1+ 893.3170
III1,2 2+ 1326.0979
III1,2 2+ 1508.6670
III1,2 2+ 1836.2472
III1,2 2+ 1836.2472
III1,2 2+ 1917.9168
III1,2 1+ 2326.0893
III1,2 1+ 2488.1067
III1,2 1+ 2650.1744
III1,2 1+ 2812.2145
III1,2 1+ 3015.2753
III1,2 1+ 3177.3636
m/z

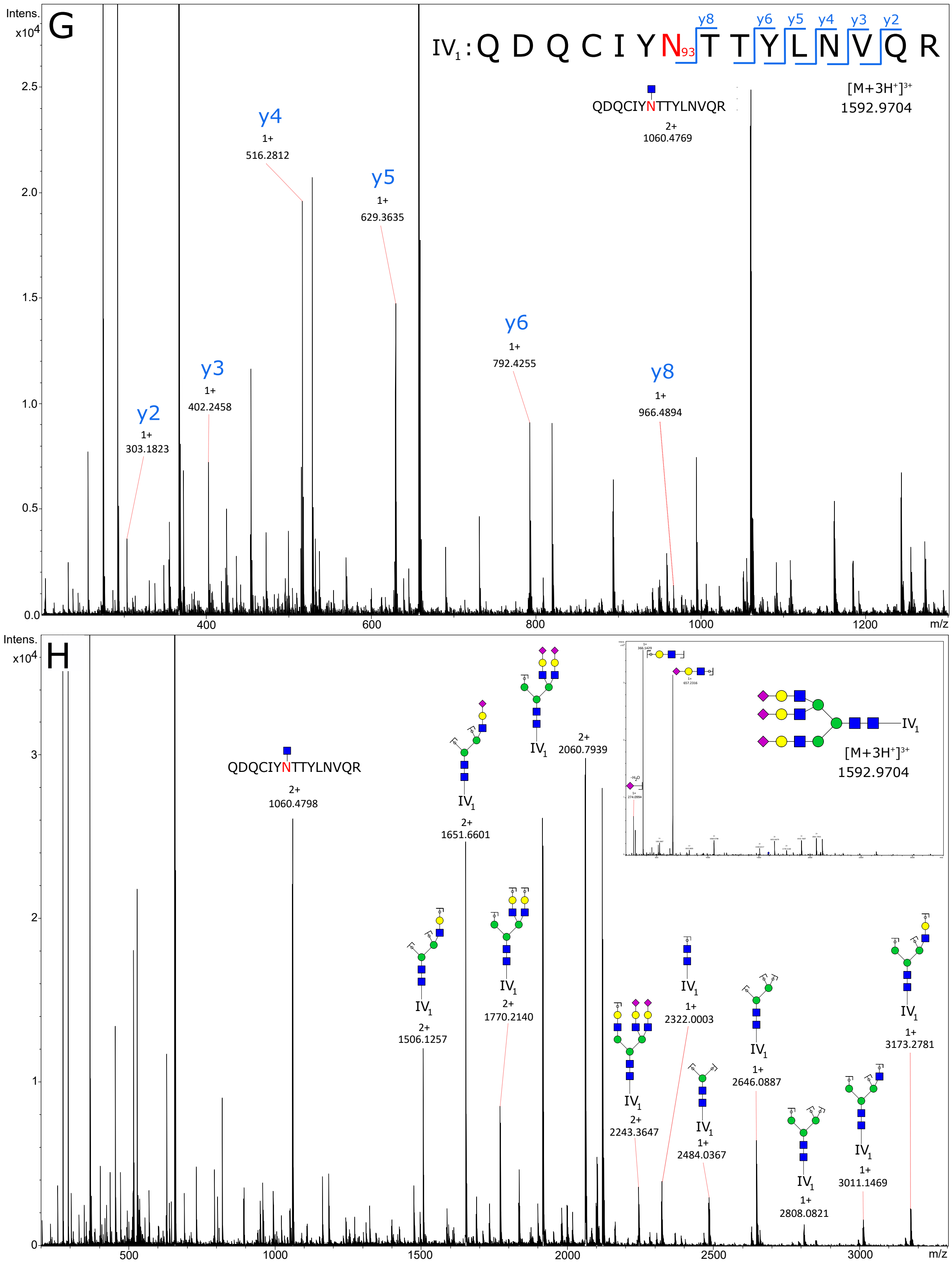

G
Intens. x10^4
IV1 : Q D Q C I Y N93 T T Y L N V Q R
y8 y6 y5 y4 y3 y2
QDQCIYNTTYLNVQR
2+
1060.4769
[M+3H+]3+
1592.9704
y4 1+ 516.2812
y5 1+ 629.3635
y6 1+ 792.4255
y8 1+ 966.4894
y3 1+ 402.2458
y2 1+ 303.1823
m/z
H
Intens. x10^4
QDQCIYNTTYLNVQR
2+
1060.4798
IV1 2+ 1651.6601
IV1 2+ 2060.7939
IV1 2+ 1506.1257
IV1 2+ 1770.2140
IV1 2+ 2243.3647
IV1 1+ 2322.0003
IV1 1+ 2484.0367
IV1 1+ 2646.0887
IV1 1+ 2808.0821
IV1 1+ 3011.1469
IV1 1+ 3173.2781
IV1
[M+3H+]3+
1592.9704
m/z

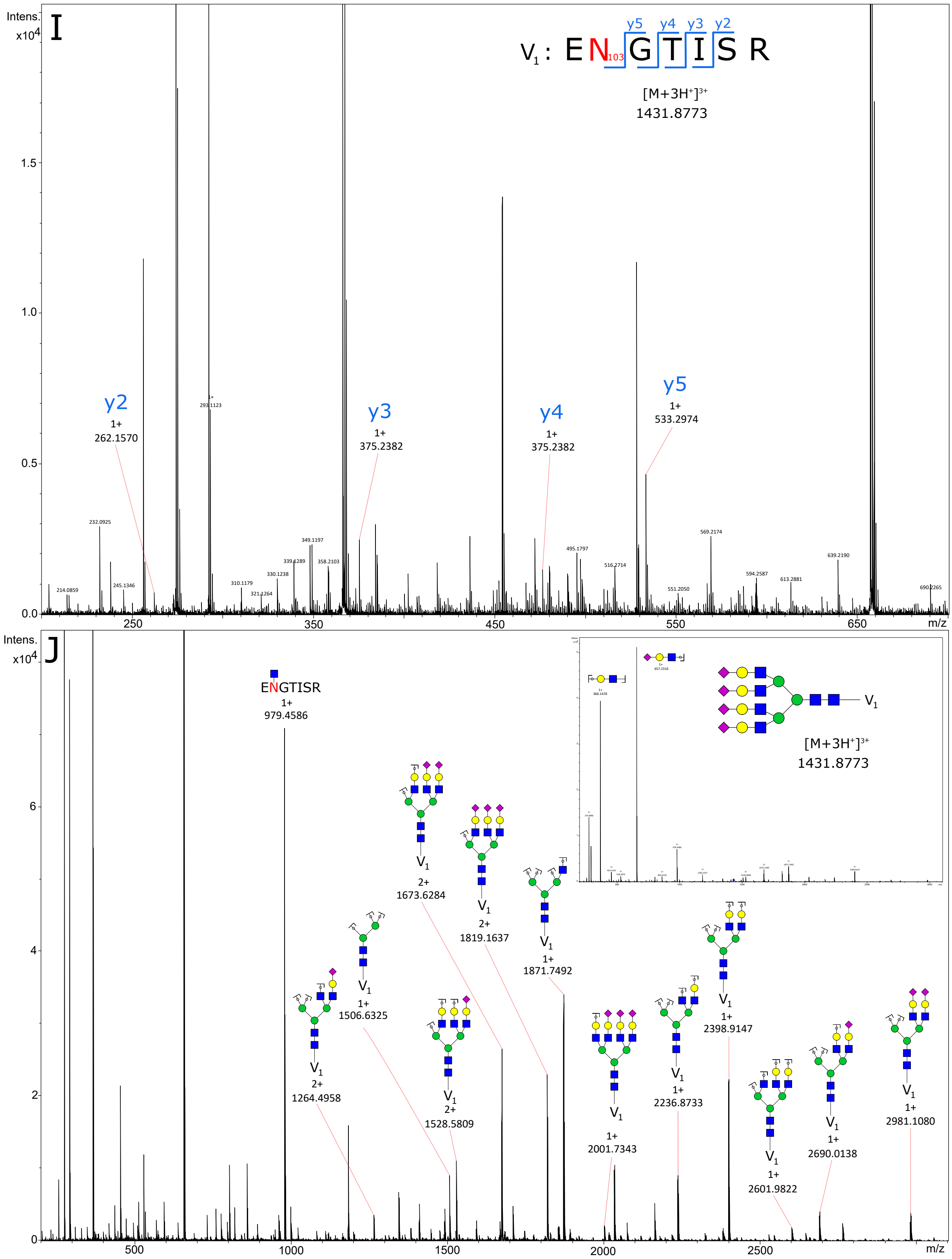
I
V1: ENGTISR
y5 y4 y3 y2
[M+3H+]3+
1431.8773
y2
1+
262.1570
y3
1+
375.2382
y4
1+
375.2382
y5
1+
533.2974
Intens.
x10^4
m/z
J
ENGTISR
1+
979.4586
[M+3H+]3+
1431.8773
2+
1264.4958
1+
1506.6325
2+
1528.5809
2+
1673.6284
2+
1819.1637
1+
1871.7492
1+
2001.7343
1+
2236.8733
1+
2398.9147
1+
2601.9822
1+
2690.0138
1+
2981.1080

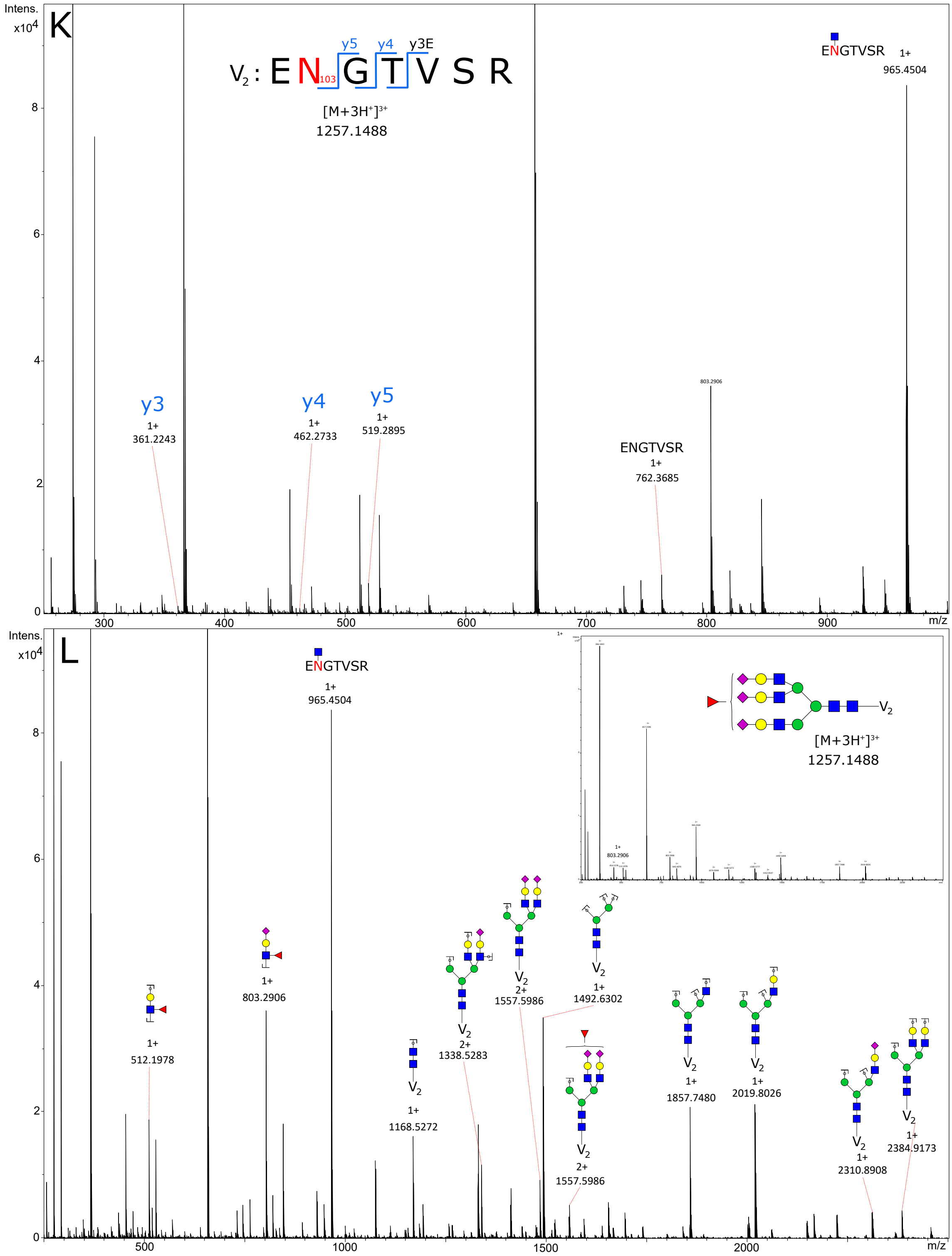

Figure S2: MS/MS fragmentation spectra for both the peptide and the glycan parts (for various glycoforms), respectively, of $I_{1,2}$ (A and B), $II_{1,2}$ (C and D), $III_{1,2}$ (E and F), IV_1 (G and H), V_1 (I and J) and V_2 (K and L).

Figure S3: Automatically annotated spectra based on MS/MS data for 87 glycopeptides identified by Byonic search engine. Available as a separate file.

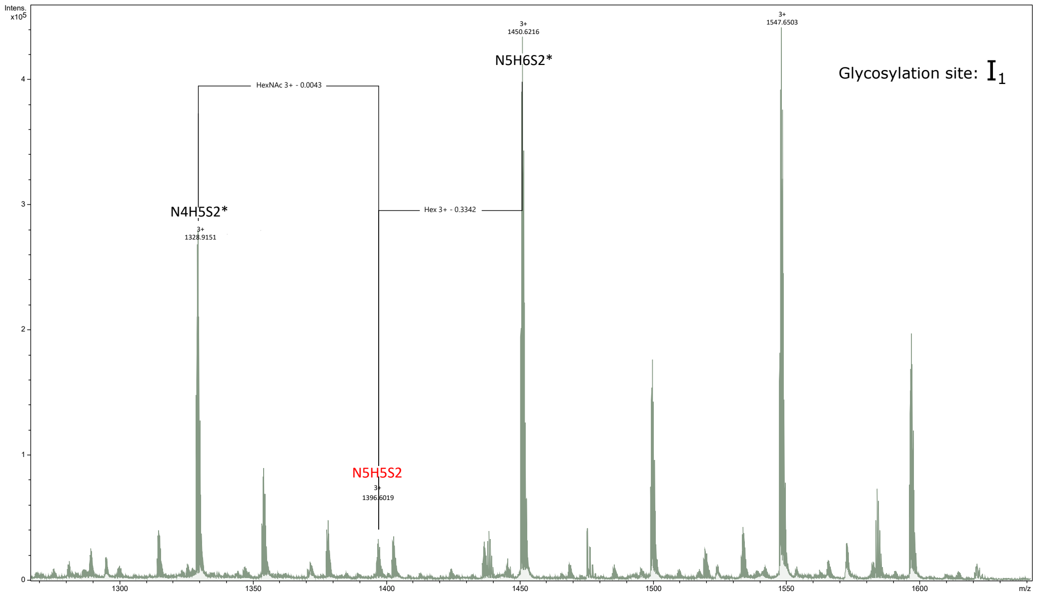
Glycosylation site: I_1
N5H6S2*
N4H5S2*
N5H5S2
HexNAc 3+ - 0.0043
Hex 3+ - 0.3342

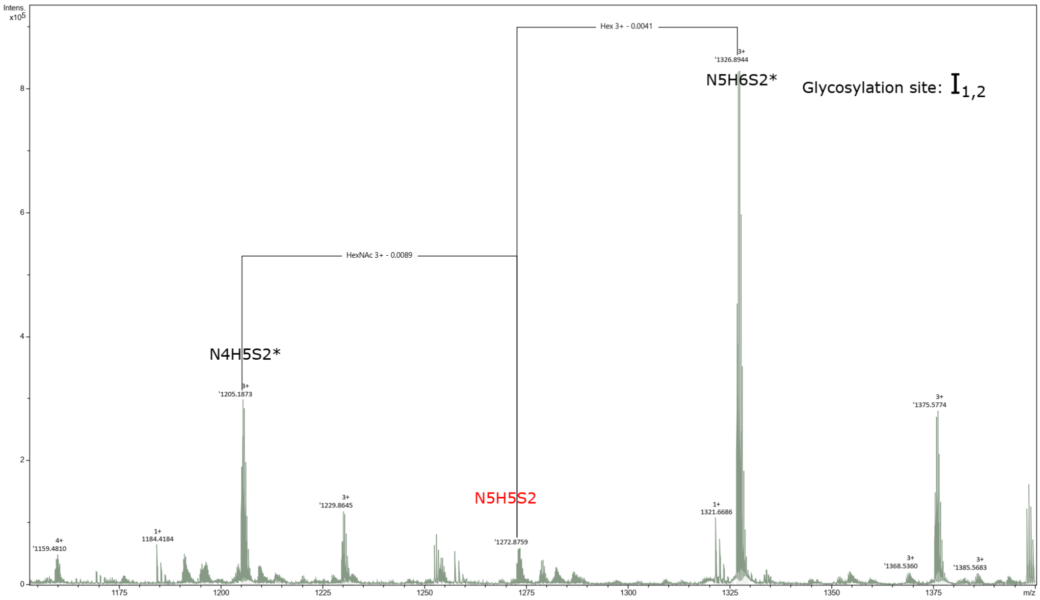
Glycosylation site: $I_{1,2}$
N5H6S2*
N4H5S2*
N5H5S2
Hex 3+ - 0.0041
HexNAc 3+ - 0.0089

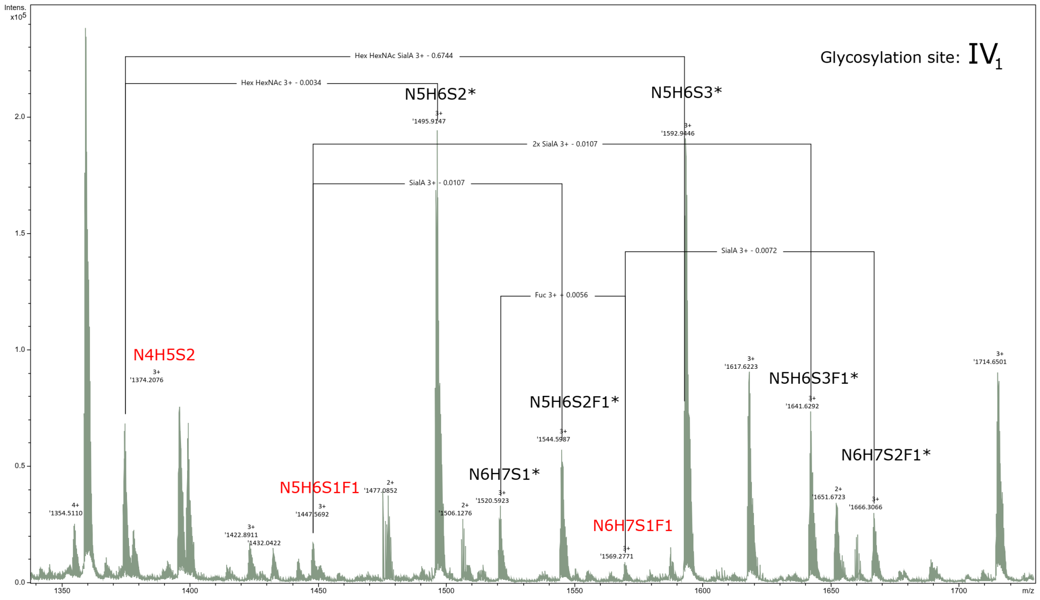
Glycosylation site: IV_1
N5H6S2*
N5H6S3*
N4H5S2
N5H6S3F1*
N5H6S2F1*
N6H7S2F1*
N6H7S1*
N5H6S1F1
N6H7S1F1
Hex HexNAc SialA 3+ - 0.6744
Hex HexNAc 3+ - 0.0034
2x SialA 3+ - 0.0107
SialA 3+ - 0.0107
SialA 3+ - 0.0072
Fuc 3+ + 0.0056

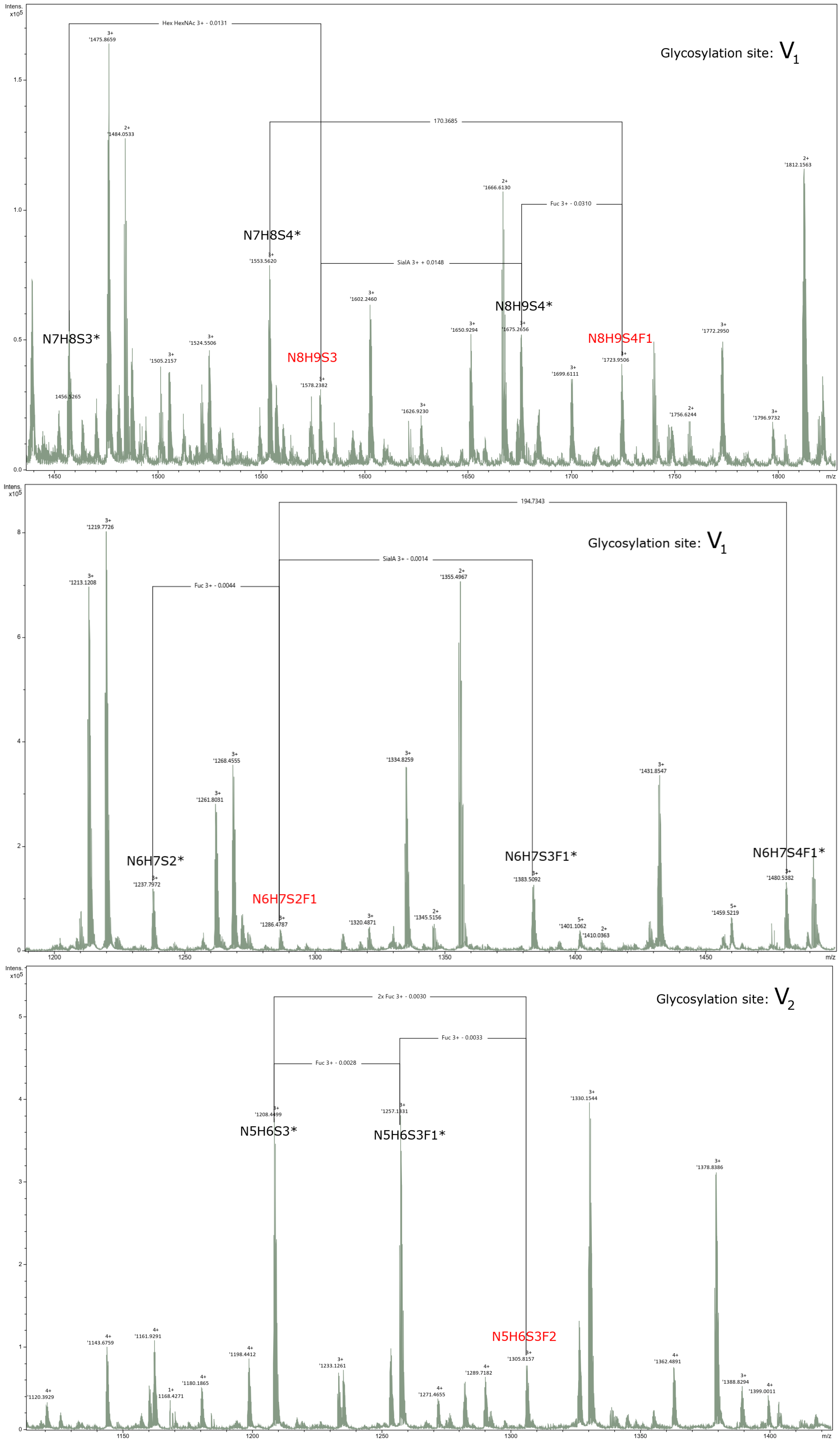

Figure S4: Annotation of AGP glycopeptides based on m/z value matched to internal database and delta m/z representing mass differences of one or more monosaccharides from the glycopeptides confirmed by MS/MS. Compositions in red represent annotated glycopeptides; Compositions in black with the asterisk represent the AGP glycopeptides confirmed by MS/MS data.

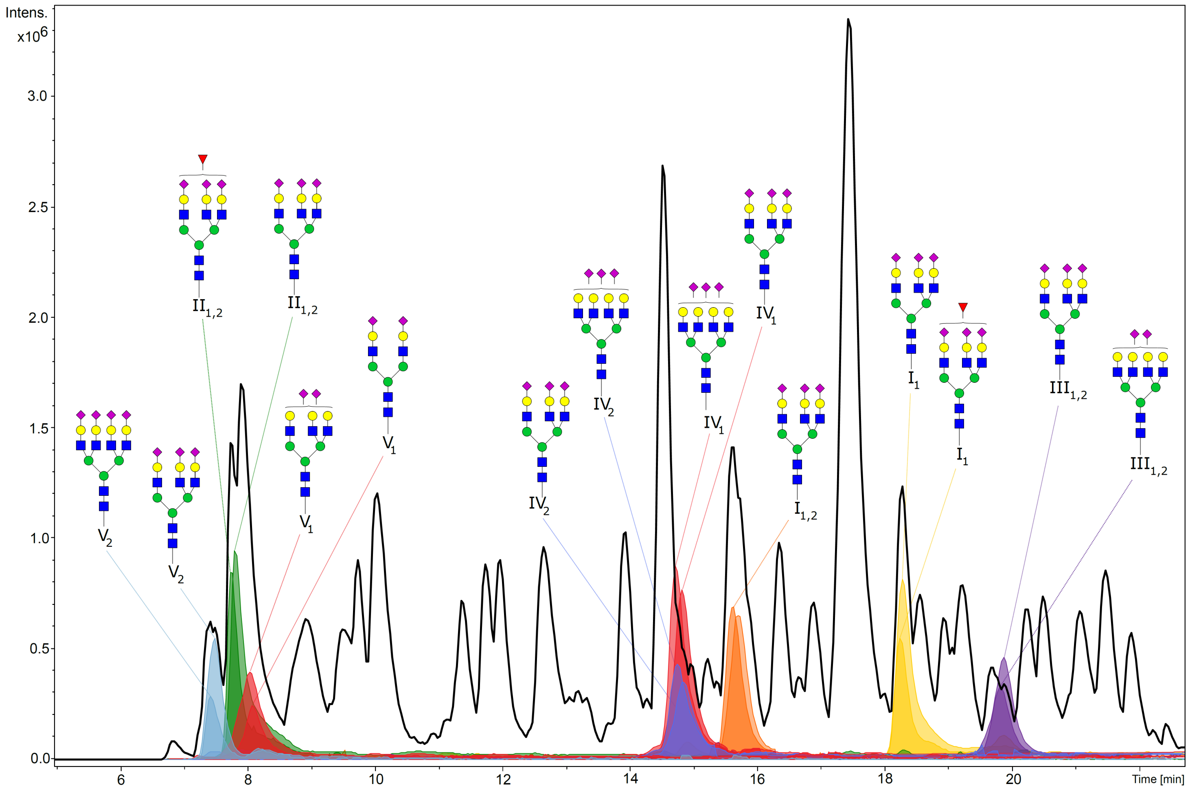

Figure S5: An overlapped chromatogram including BPI and extracted ion traces of the most abundant glycopeptides from each glycosylation site. TFA was used in the mobile phase as an ion-pairing agent.

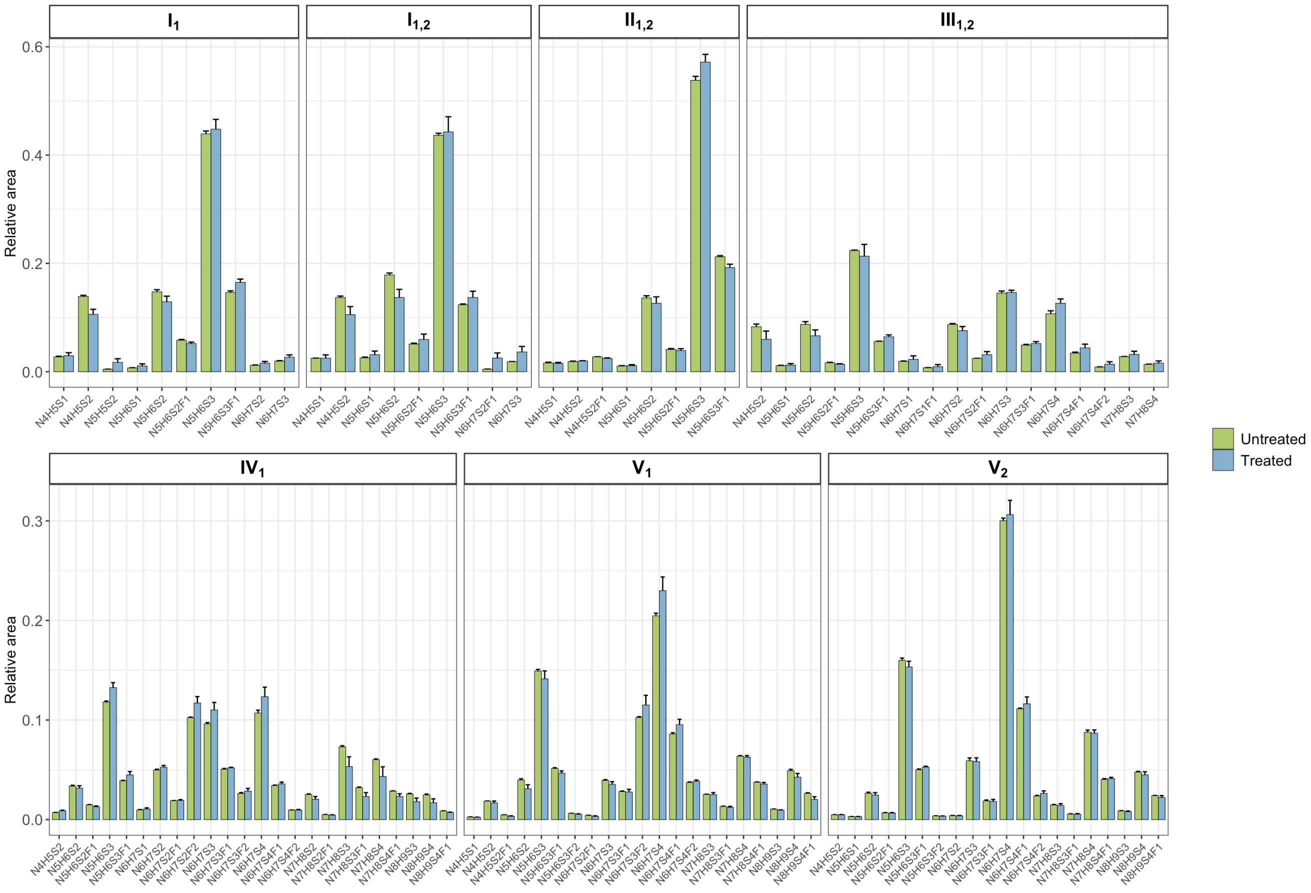

Figure S6: Differences in relative abundances of all AGP glycopeptides obtained from the commercial AGP standard untreated and treated with the method's enrichment protocol. The error bars represent standard deviation of the quadruplicates.

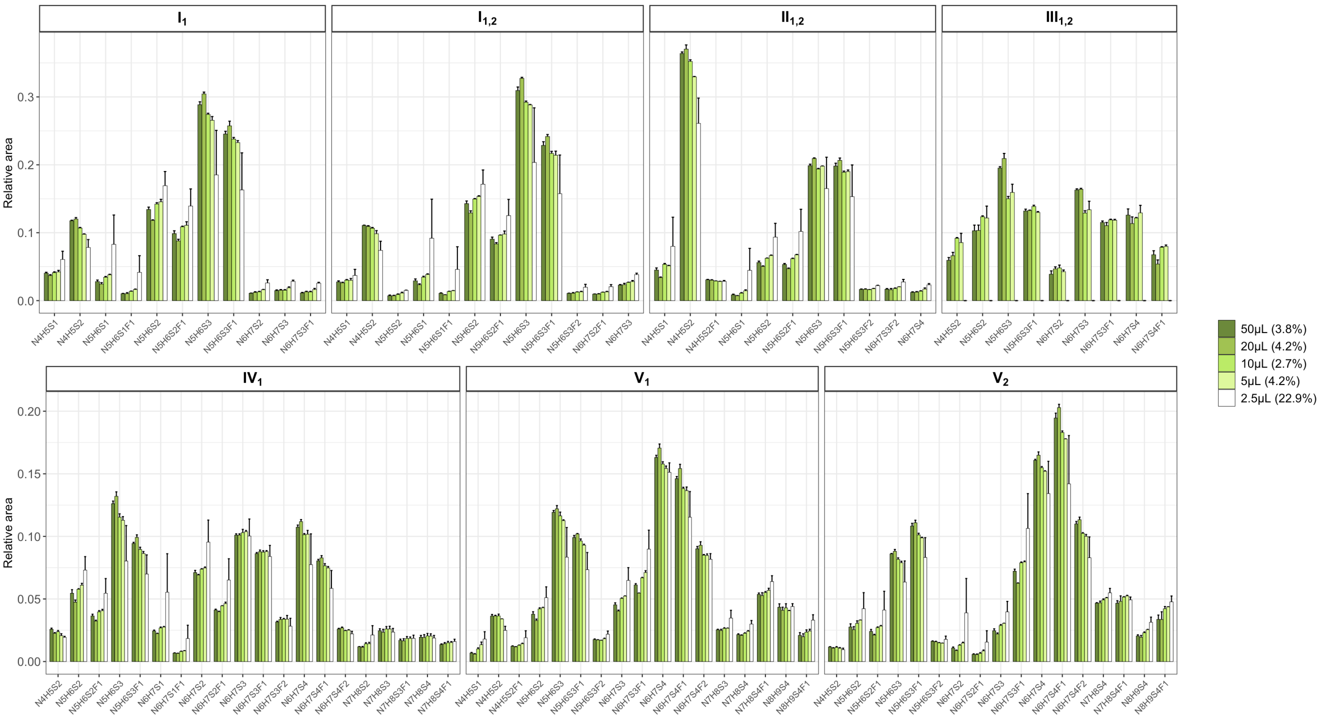

Figure S7: Comparison of AGP glycan profiles obtained from 50, 20, 10, 5 and 2.5 µl of a pooled plasma standard. The error bars represent standard deviation of the triplicates and the values next to volumes in the legend denote the average coefficient of variation for all quantified glycopeptides.

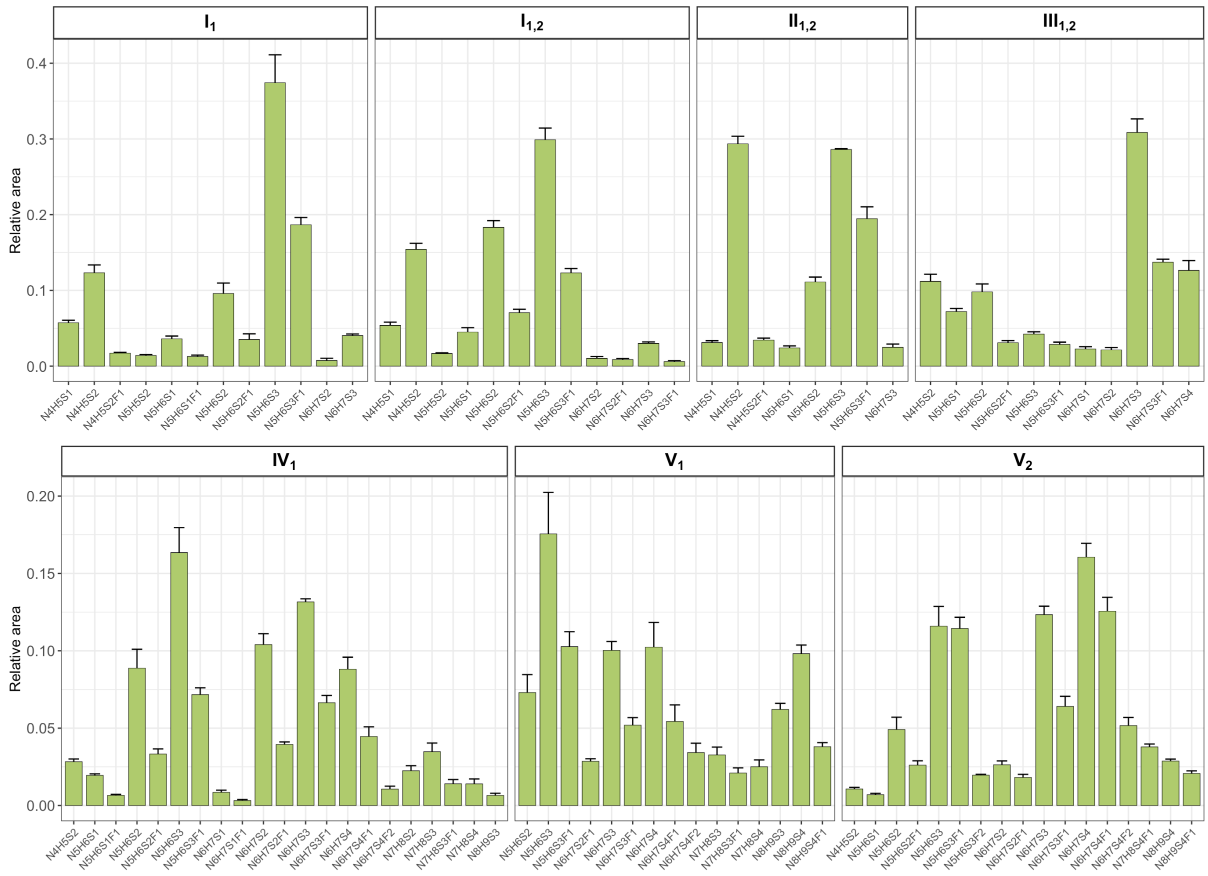

Figure S8: Differences in relative abundances of all AGP glycopeptides obtained from the pooled plasma standard. The error bars represent standard deviation of the decaplicates.

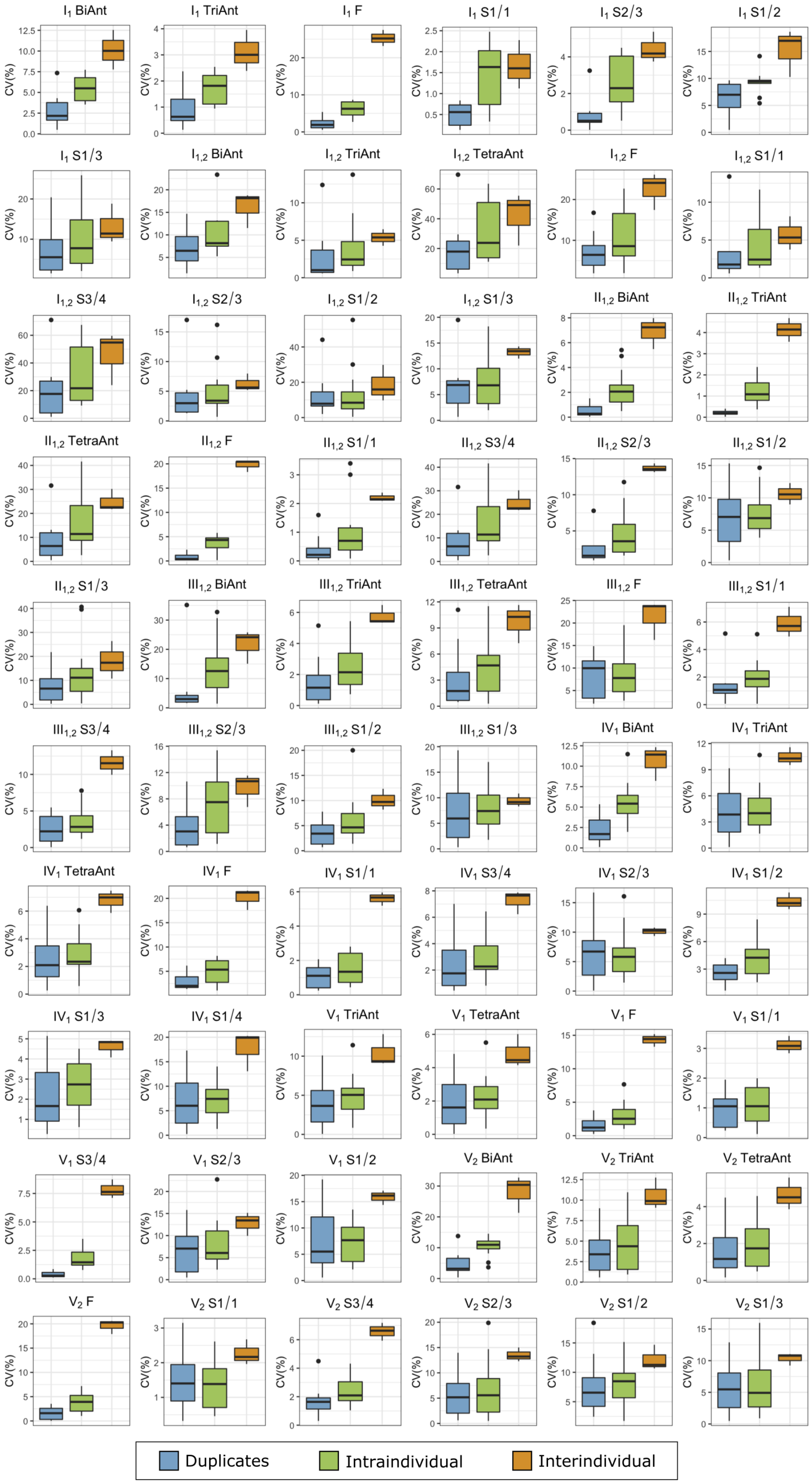

Figure S9: Temporal stability of AGP glycosylation shown with box plots.

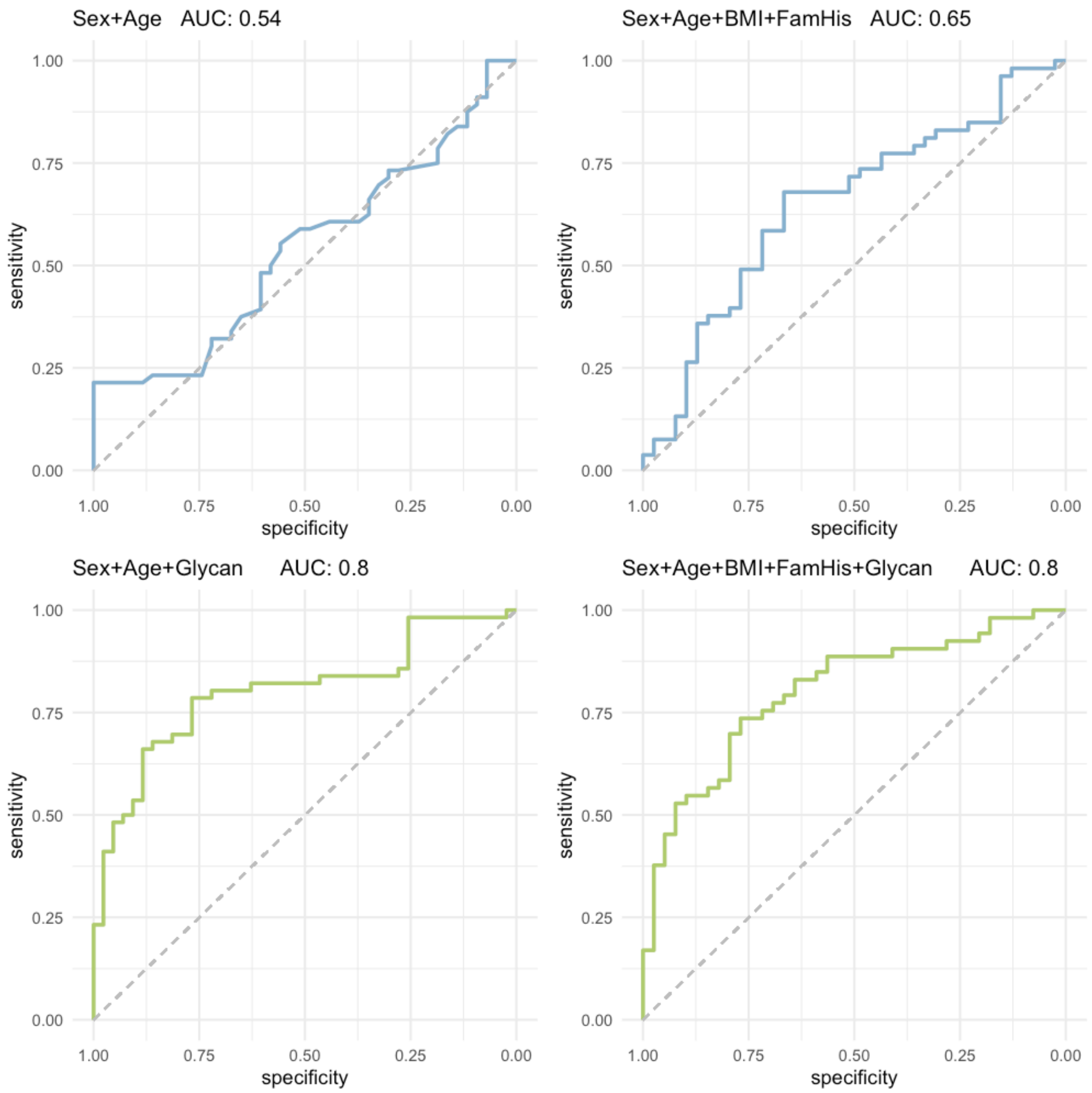

Figure S10: ROC curve analysis of four models for prediction of increased type 2 diabetes risk. Areas under the curve (AUC) and predictor combinations are listed above each curve.
