# Supplementary material for: High-Throughput and Site-Specific N-Glycosylation Analysis of Human Alpha-1-Acid Glycoprotein Offers a Great Potential for New Biomarker Discovery
Source: Mol Cell Proteomics. 2021 Jan 23;20:100044. doi: 10.1074/mcp.RA120.002433 (PMC7950198; doi:10.1074/mcp.RA120.002433)
Supplement: Supplemental Data 4 [file mmc5.pdf]

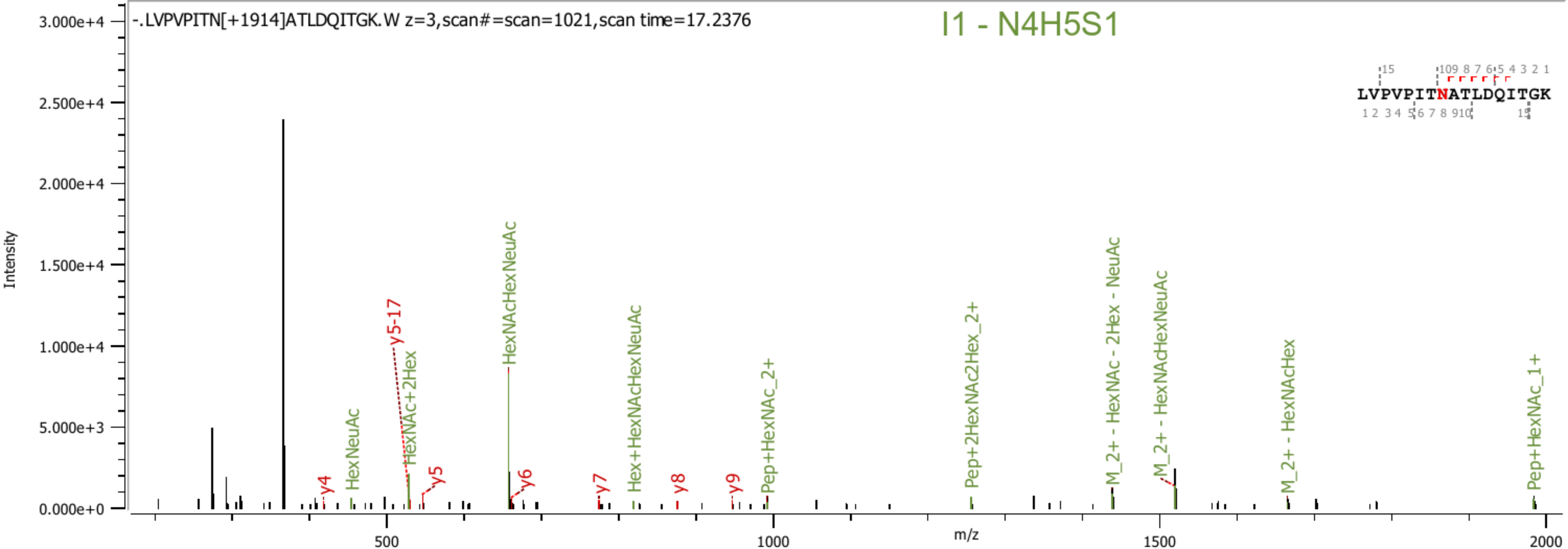

-.LVPVPITN[+1914]ATLDQITGK.W z=3,scan#=scan=1021,scan time=17.2376
I1 - N4H5S1
LVPVPITNATLDQITGK
Intensity
m/z
y4
HexNeuAc
y5-17
HexNAc+2Hex
y5
HexNAcHexNeuAc
y6
y7
Hex+HexNAcHexNeuAc
y8
y9
Pep+HexNAc_2+
Pep+2HexNAc2Hex_2+
M_2+ - HexNAc - 2Hex - NeuAc
M_2+ - HexNAcHexNeuAc
M_2+ - HexNAcHex
Pep+HexNAc_1+

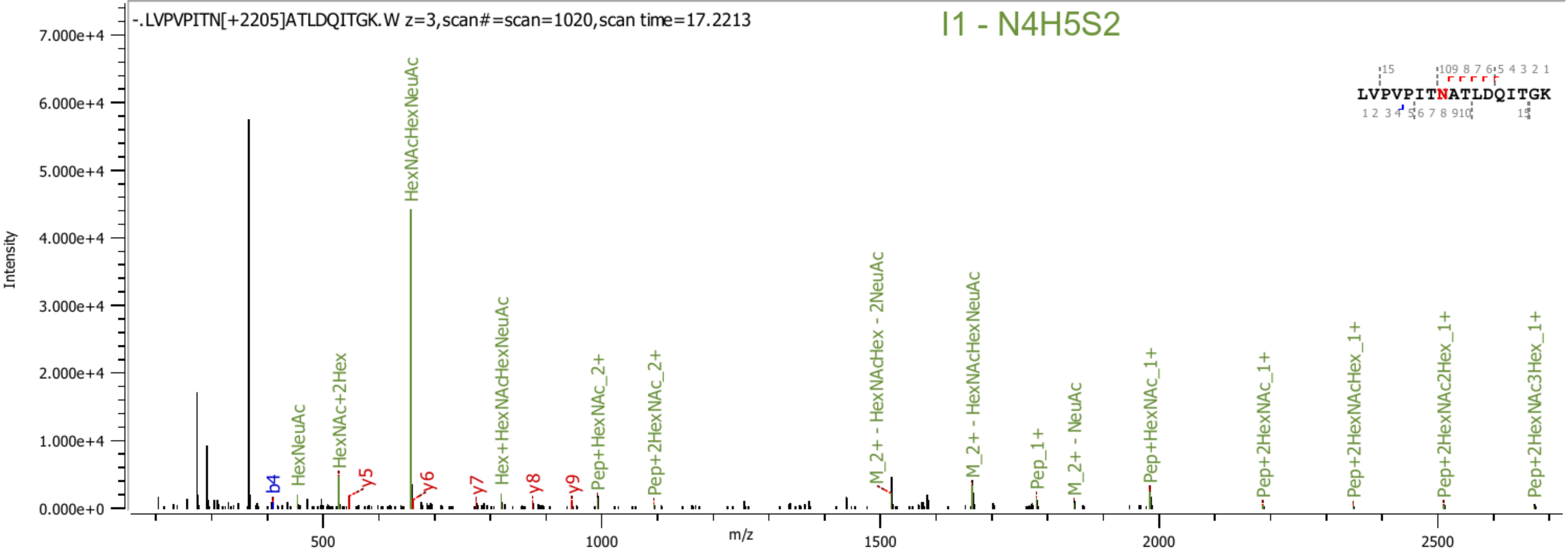

-.LVPVPITN[+2205]ATLDQITGK.W z=3,scan#=scan=1020,scan time=17.2213
I1 - N4H5S2
LVPVPITNATLDQITGK
Intensity
m/z
b4
HexNeuAc
HexNAc+2Hex
y5
HexNAcHexNeuAc
y6
y7
Hex+HexNAcHexNeuAc
y8
y9
Pep+HexNAc_2+
Pep+2HexNAc_2+
M_2+ - HexNAcHex - 2NeuAc
M_2+ - HexNAcHexNeuAc
Pep_1+
M_2+ - NeuAc
Pep+HexNAc_1+
Pep+2HexNAc_1+
Pep+2HexNAcHex_1+
Pep+2HexNAc2Hex_1+
Pep+2HexNAc3Hex_1+

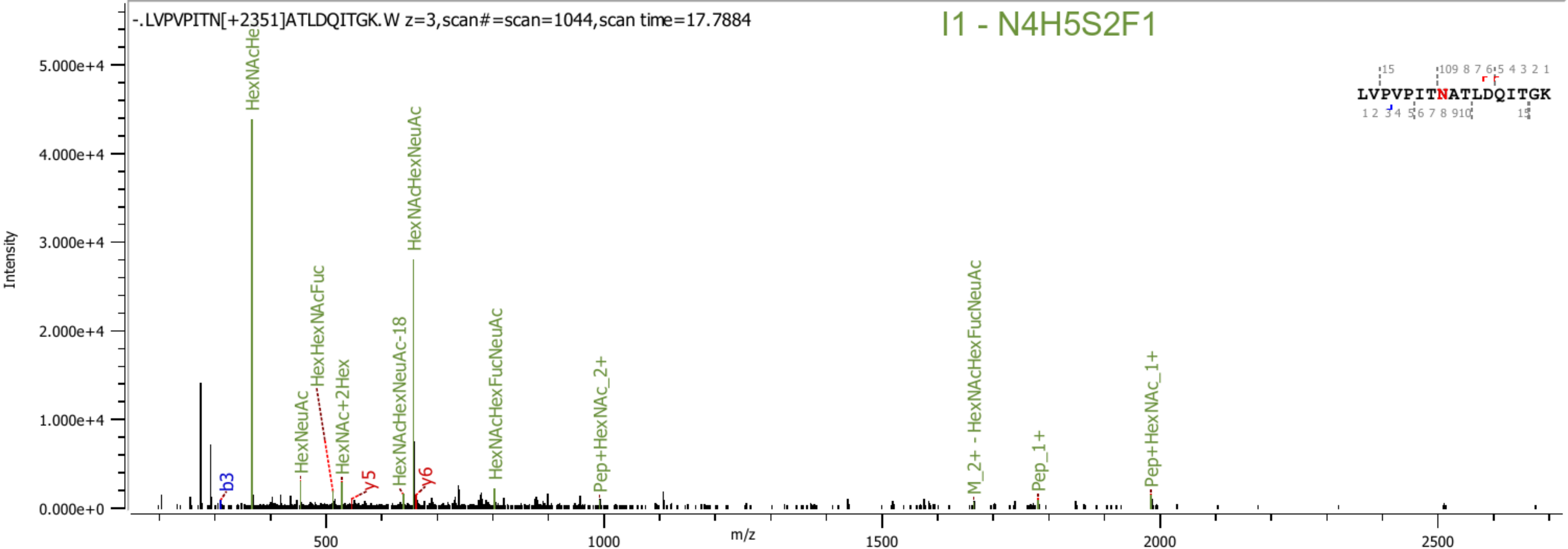

-.LVPVPITN[+2351]ATLDQITGK.W z=3,scan#=scan=1044,scan time=17.7884
I1 - N4H5S2F1
LVPVPITNATLDQITGK
Intensity
m/z
HexNAcHex
HexNAcHexNeuAc
HexHexNAcFuc
HexNeuAc
HexNAc+2Hex
HexNAcHexNeuAc-18
HexNAcHexFucNeuAc
Pep+HexNAc_2+
M_2+ - HexNAcHexFucNeuAc
Pep_1+
Pep+HexNAc_1+
b3
y5
y6

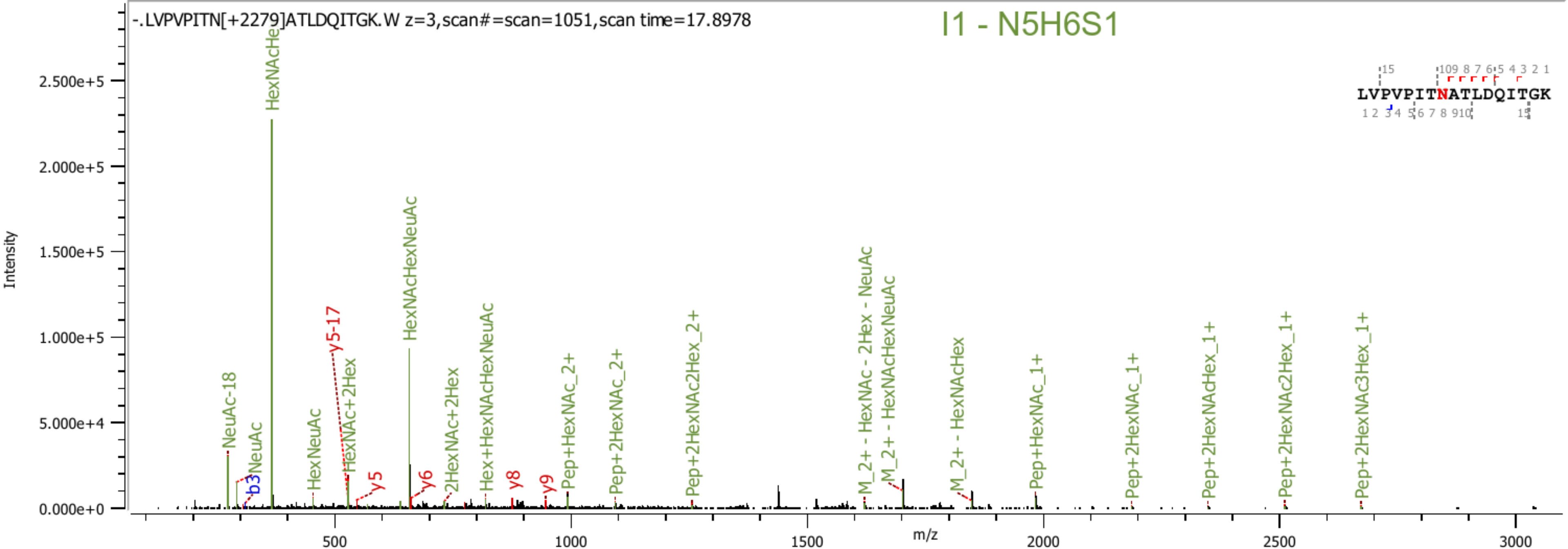

-.LVPVPITN[+2279]ATLDQITGK.W z=3,scan#=scan=1051,scan time=17.8978
I1 - N5H6S1
LVPVPITNATLDQITGK
Intensity
m/z
NeuAc-18
NeuAc
b3
HexNAcHex
HexNeuAc
y5-17
HexNAc+2Hex
y5
HexNAcHexNeuAc
y6
2HexNAc+2Hex
Hex+HexNAcHexNeuAc
y8
y9
Pep+HexNAc_2+
Pep+2HexNAc_2+
Pep+2HexNAc2Hex_2+
M_2+ - HexNAc - 2Hex - NeuAc
M_2+ - HexNAcHexNeuAc
M_2+ - HexNAcHex
Pep+HexNAc_1+
Pep+2HexNAc_1+
Pep+2HexNAcHex_1+
Pep+2HexNAc2Hex_1+
Pep+2HexNAc3Hex_1+

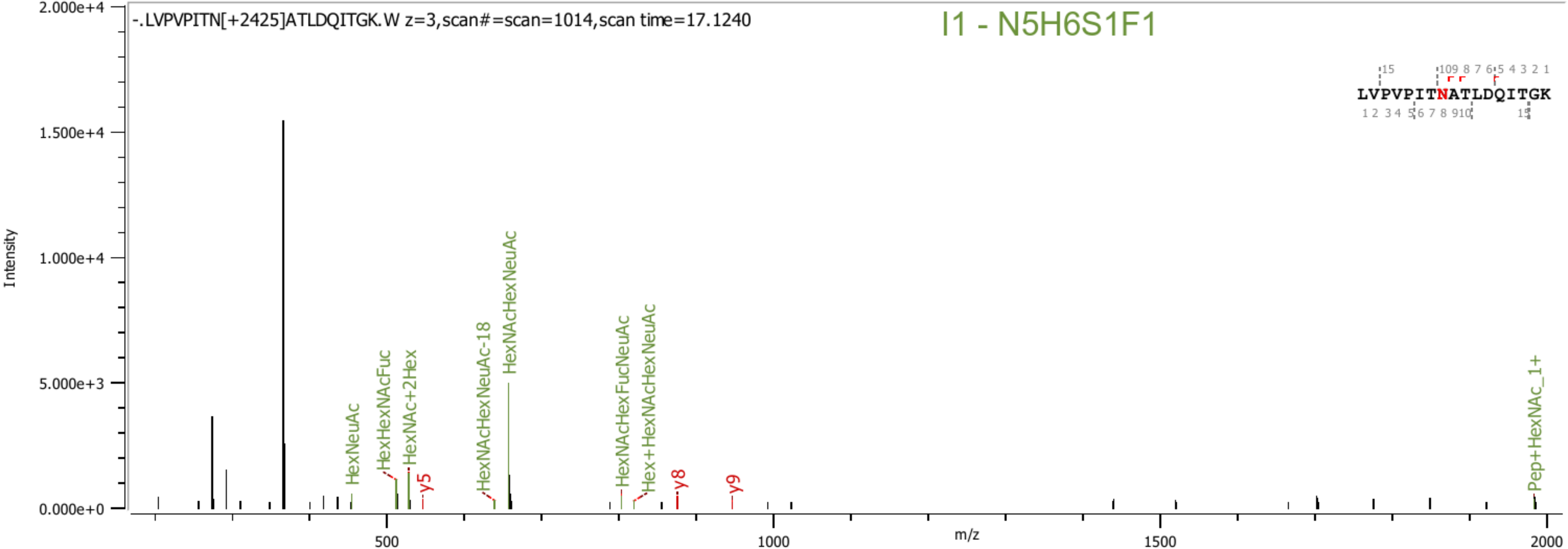

-.LVPVPITN[+2425]ATLDQITGK.W z=3,scan#=scan=1014,scan time=17.1240
I1 - N5H6S1F1
LVPVPITNATLDQITGK
Intensity
2.000e+4
1.500e+4
1.000e+4
5.000e+3
0.000e+0
HexNeuAc
HexHexNAcFuc
HexNAc+2Hex
y5
HexNAcHexNeuAc-18
HexNAcHexNeuAc
HexNAcHexFucNeuAc
Hex+HexNAcHexNeuAc
y8
y9
Pep+HexNAc_1+
500
1000
1500
2000
m/z

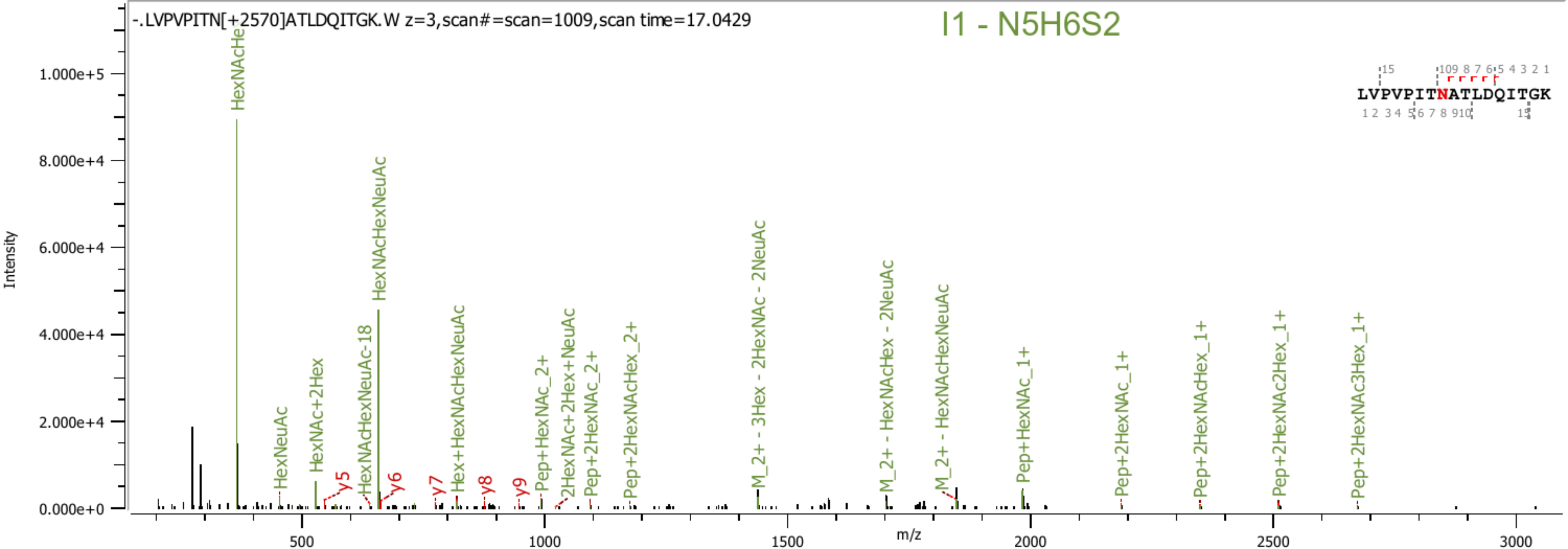
-.LVPVPITN[+2570]ATLDQITGK.W z=3,scan#=scan=1009,scan time=17.0429
I1 - N5H6S2
LVPVPITNATLDQITGK
Intensity
m/z
HexNAcHex
HexNAcHexNeuAc
HexNeuAc
HexNAc+2Hex
HexNAcHexNeuAc-18
Hex+HexNAcHexNeuAc
2HexNAc+2Hex+NeuAc
Pep+HexNAc_2+
Pep+2HexNAc_2+
Pep+2HexNAcHex_2+
M_2+ - 3Hex - 2HexNAc - 2NeuAc
M_2+ - HexNAcHex - 2NeuAc
M_2+ - HexNAcHexNeuAc
Pep+HexNAc_1+
Pep+2HexNAc_1+
Pep+2HexNAcHex_1+
Pep+2HexNAc2Hex_1+
Pep+2HexNAc3Hex_1+
y5
y6
y7
y8
y9
1.000e+5
8.000e+4
6.000e+4
4.000e+4
2.000e+4
0.000e+0
500
1000
1500
2000
2500
3000

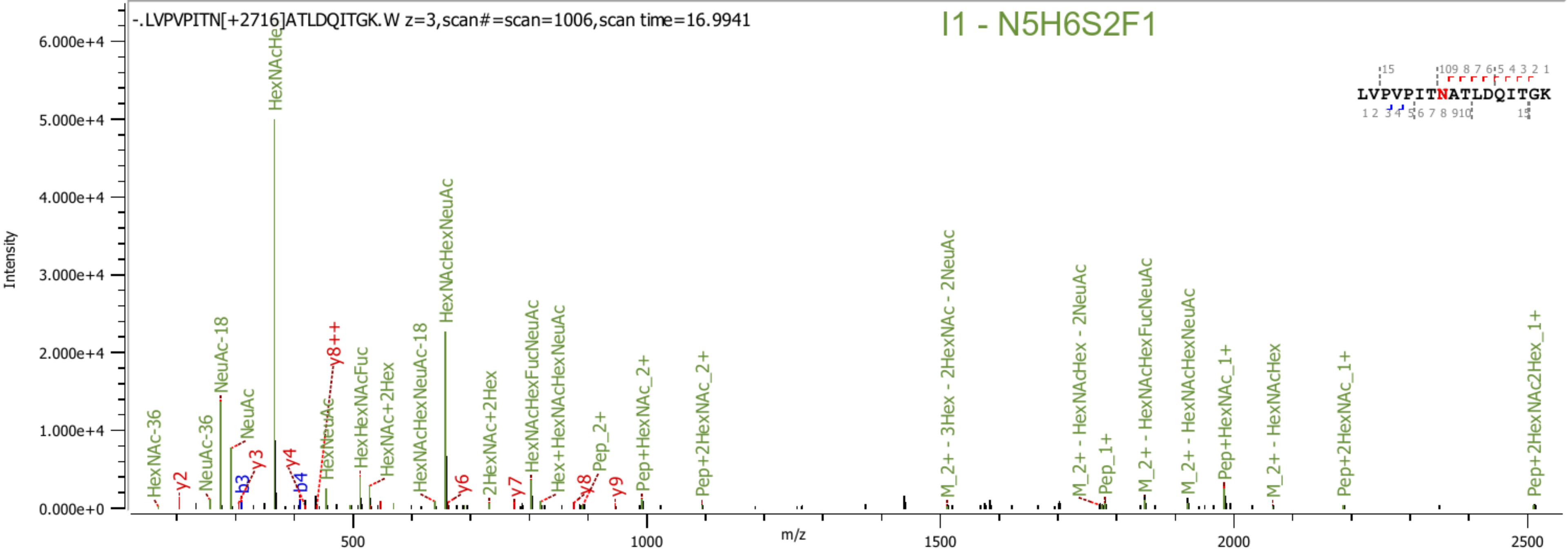
-.LVPVPITN[+2716]ATLDQITGK.W z=3,scan#=scan=1006,scan time=16.9941
I1 - N5H6S2F1
LVPVPITNATLDQITGK
Intensity
m/z
6.000e+4
5.000e+4
4.000e+4
3.000e+4
2.000e+4
1.000e+4
0.000e+0
500
1000
1500
2000
2500
HexNAc-36
y2
NeuAc-36
NeuAc-18
b3
NeuAc
y3
HexNAcHex
y4
b4
HexNeuAc
y8++
HexHexNAcFuc
HexNAc+2Hex
HexNAcHexNeuAc-18
HexNAcHexNeuAc
y6
2HexNAc+2Hex
y7
HexNAcHexFucNeuAc
Hex+HexNAcHexNeuAc
y8
Pep_2+
y9
Pep+HexNAc_2+
Pep+2HexNAc_2+
M_2+ - 3Hex - 2HexNAc - 2NeuAc
M_2+ - HexNAcHex - 2NeuAc
Pep_1+
M_2+ - HexNAcHexFucNeuAc
M_2+ - HexNAcHexNeuAc
Pep+HexNAc_1+
M_2+ - HexNAcHex
Pep+2HexNAc_1+
Pep+2HexNAc2Hex_1+

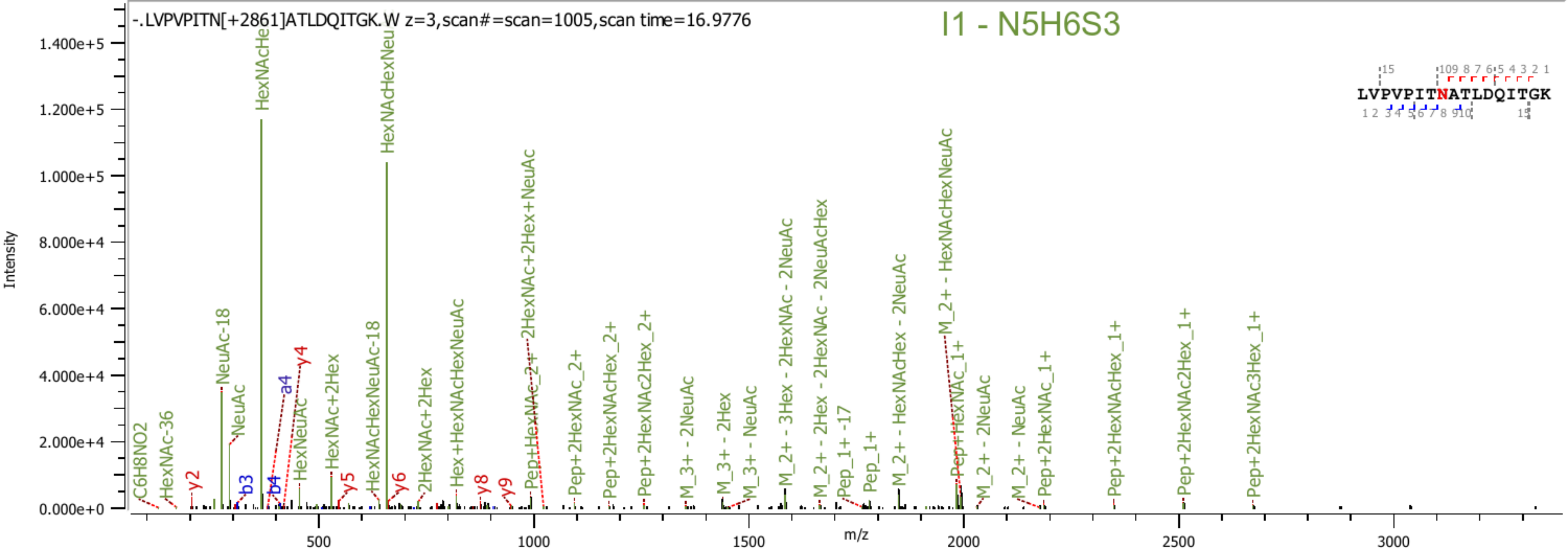
-.LVPVPITN[+2861]ATLDQITGK.W z=3,scan#=scan=1005,scan time=16.9776
I1 - N5H6S3
LVPVPITNATLDQITGK
Intensity
m/z
HexNAcHex
HexNAcHexNeu
2HexNAc+2Hex+NeuAc
M_2+ - HexNAcHexNeuAc
NeuAc-18
NeuAc
HexNAcHexNeuAc-18
Hex+HexNAcHexNeuAc
M_3+ - 2HexNAc - 2NeuAc
M_2+ - 2HexNAc - 2NeuAcHex
M_2+ - HexNAcHex - 2NeuAc
Pep+HexNAc_1+
Pep+2HexNAcHex_1+
Pep+2HexNAc2Hex_1+
Pep+2HexNAc3Hex_1+
Pep+2HexNAc_2+
Pep+2HexNAcHex_2+
Pep+2HexNAc2Hex_2+
Pep+HexNAc_2+
M_3+ - 2NeuAc
M_3+ - 2Hex
M_3+ - NeuAc
M_2+ - 3Hex
M_2+ - 2Hex
Pep_1+ -17
Pep_1+
M_2+ - 2NeuAc
M_2+ - NeuAc
Pep+2HexNAc_1+
C6H8NO2
HexNAc-36
HexNeuAc
HexNAc+2Hex
2HexNAc+2Hex
y2
y4
y5
y6
y8
y9
a4
b3
b4

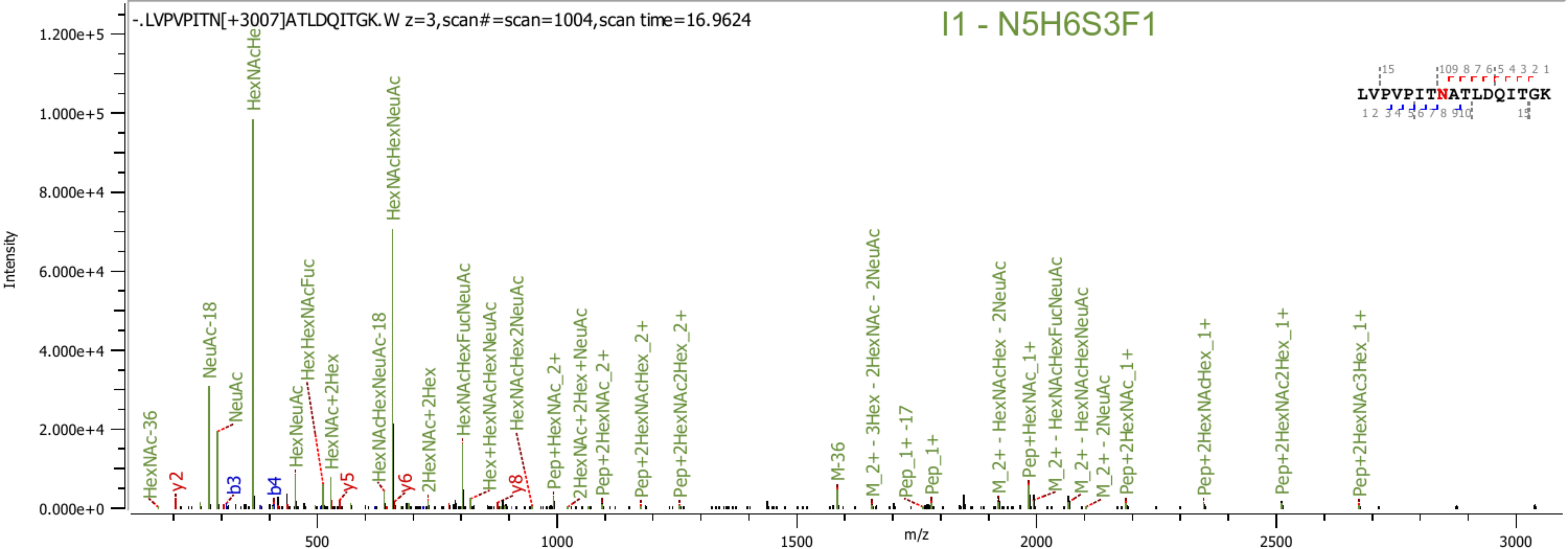

-.LVPVPITN[+3007]ATLDQITGK.W z=3,scan#=scan=1004,scan time=16.9624
I1 - N5H6S3F1
LVPVPITNATLDQITGK
Intensity
m/z
HexNAc-36
y2
NeuAc-18
b3
NeuAc
HexNAcHe
b4
HexNeuAc
HexHexNAcFuc
HexNAc+2Hex
y5
HexNAcHexNeuAc-18
HexNAcHexNeuAc
y6
2HexNAc+2Hex
HexNAcHexFucNeuAc
Hex+HexNAcHexNeuAc
y8
HexNAcHex2NeuAc
Pep+HexNAc_2+
2HexNAc+2Hex+NeuAc
Pep+2HexNAc_2+
Pep+2HexNAcHex_2+
Pep+2HexNAc2Hex_2+
M-36
M_2+ - 3Hex - 2HexNAc - 2NeuAc
Pep_1+ -17
Pep_1+
M_2+ - HexNAcHex - 2NeuAc
Pep+HexNAc_1+
M_2+ - HexNAcHexFucNeuAc
M_2+ - HexNAcHexNeuAc
M_2+ - 2NeuAc
Pep+2HexNAc_1+
Pep+2HexNAcHex_1+
Pep+2HexNAc2Hex_1+
Pep+2HexNAc3Hex_1+

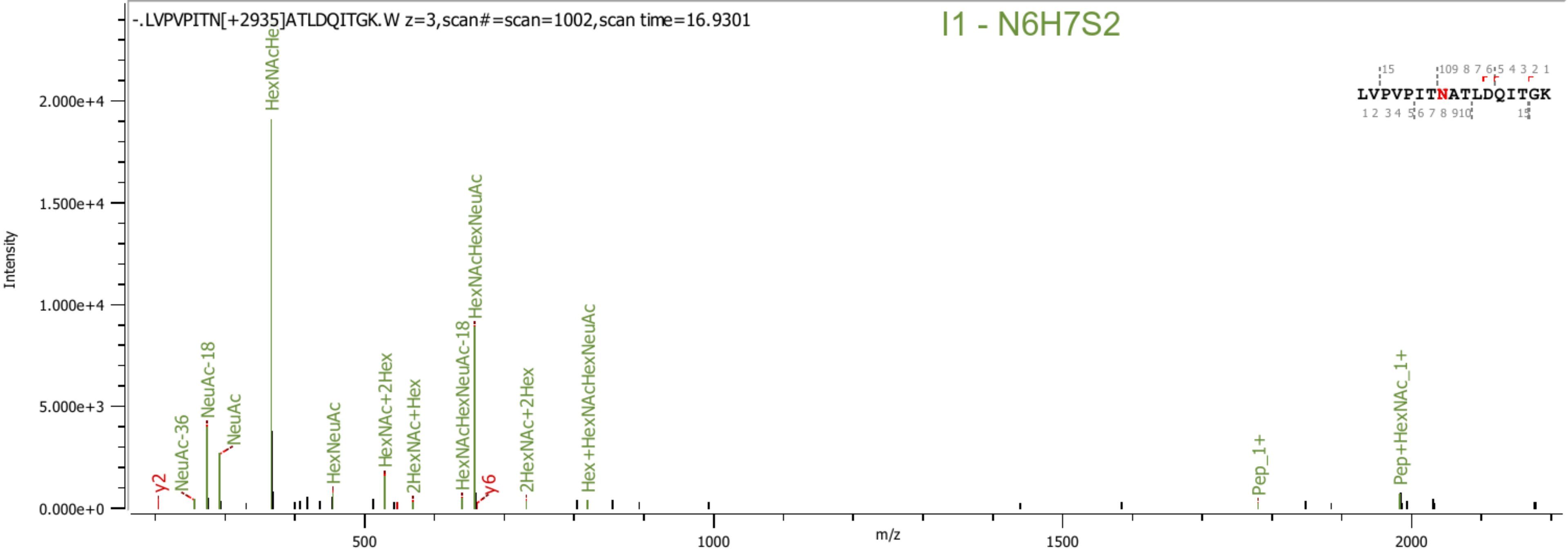
-.LVPVPITN[+2935]ATLDQITGK.W z=3,scan#=scan=1002,scan time=16.9301
I1 - N6H7S2
LVPVPITNATLDQITGK
Intensity
m/z
y2
NeuAc-36
NeuAc-18
NeuAc
HexNAcHex
HexNeuAc
HexNAc+2Hex
2HexNAc+Hex
HexNAcHexNeuAc-18
HexNAcHexNeuAc
y6
2HexNAc+2Hex
Hex+HexNAcHexNeuAc
Pep_1+
Pep+HexNAc_1+

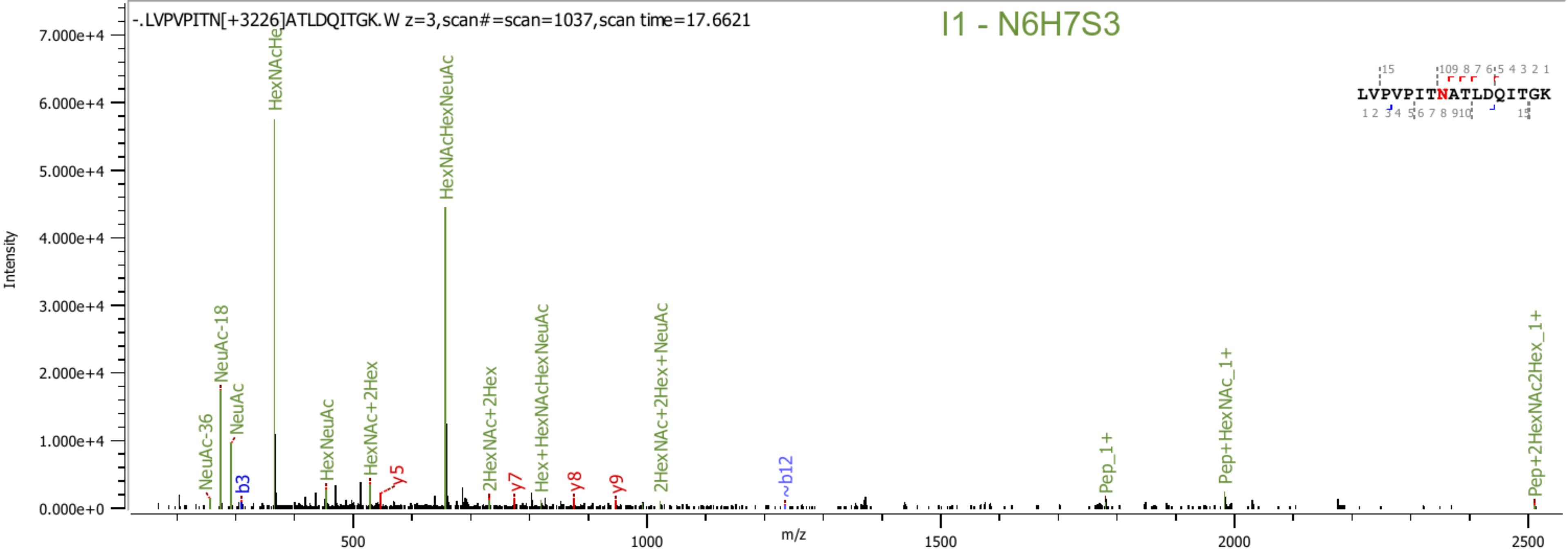

-.LVPVPITN[+3226]ATLDQITGK.W z=3,scan#=scan=1037,scan time=17.6621
I1 - N6H7S3
LVPVPITNATLDQITGK
Intensity
m/z
NeuAc-36
NeuAc-18
NeuAc
b3
HexNAcHex
HexNeuAc
HexNAc+2Hex
y5
HexNAcHexNeuAc
2HexNAc+2Hex
y7
Hex+HexNAcHexNeuAc
y8
y9
2HexNAc+2Hex+NeuAc
~b12
Pep_1+
Pep+HexNAc_1+
Pep+2HexNAc2Hex_1+

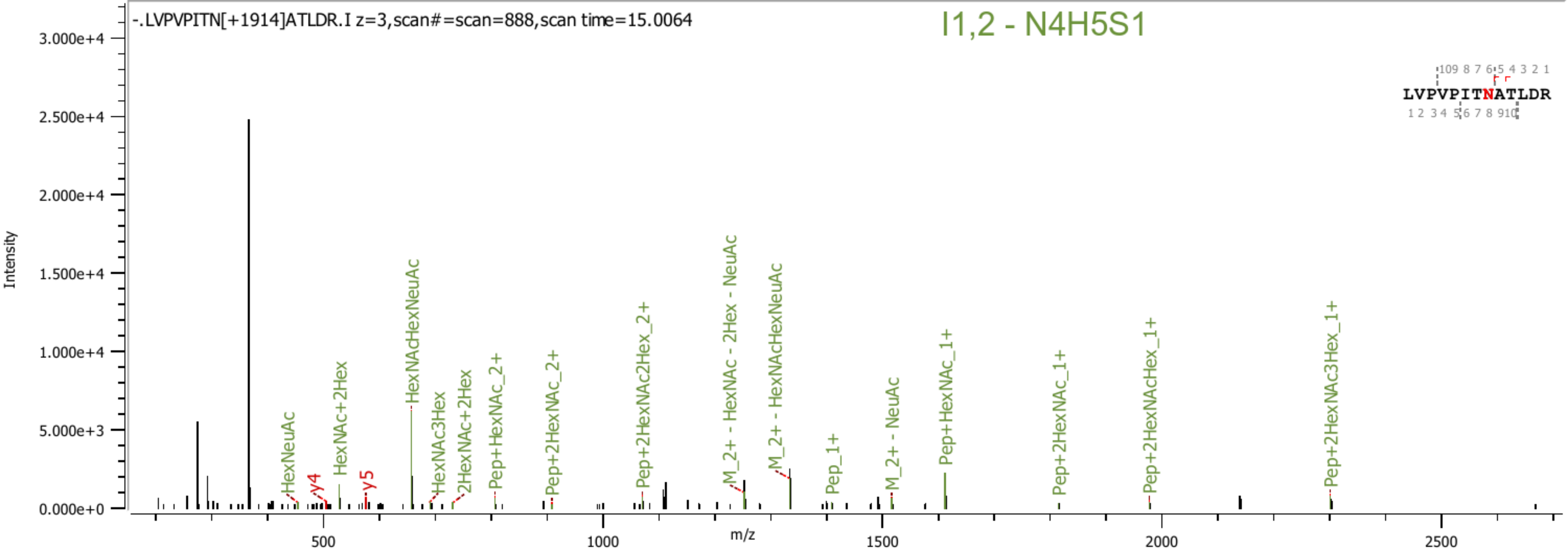

-.LVPVPITN[+1914]ATLDR.I z=3,scan#=scan=888,scan time=15.0064
I1,2 - N4H5S1
LVPVPITNATLDR
Intensity
m/z
HexNeuAc
y4
HexNAc+2Hex
y5
HexNAcHexNeuAc
HexNAc3Hex
2HexNAc+2Hex
Pep+HexNAc_2+
Pep+2HexNAc_2+
Pep+2HexNAc2Hex_2+
M_2+ - HexNAc - 2Hex - NeuAc
M_2+ - HexNAcHexNeuAc
Pep_1+
M_2+ - NeuAc
Pep+HexNAc_1+
Pep+2HexNAc_1+
Pep+2HexNAcHex_1+
Pep+2HexNAc3Hex_1+

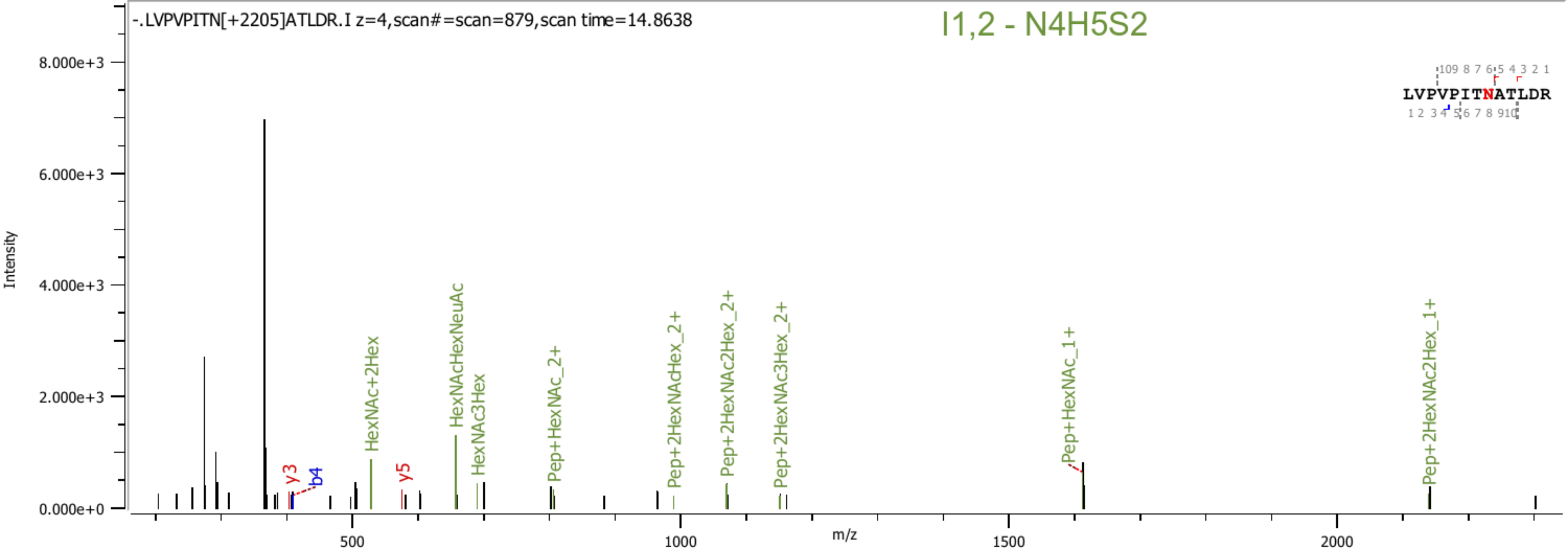

-.LVPVPITN[+2205]ATLDR.I z=4,scan#=scan=879,scan time=14.8638
I1,2 - N4H5S2
LVPVPITNATLDR
Intensity
8.000e+3
6.000e+3
4.000e+3
2.000e+3
0.000e+0
m/z
500
1000
1500
2000
y3
b4
HexNAc+2Hex
y5
HexNAcHexNeuAc
HexNAc3Hex
Pep+HexNAc_2+
Pep+2HexNAcHex_2+
Pep+2HexNAc2Hex_2+
Pep+2HexNAc3Hex_2+
Pep+HexNAc_1+
Pep+2HexNAc2Hex_1+

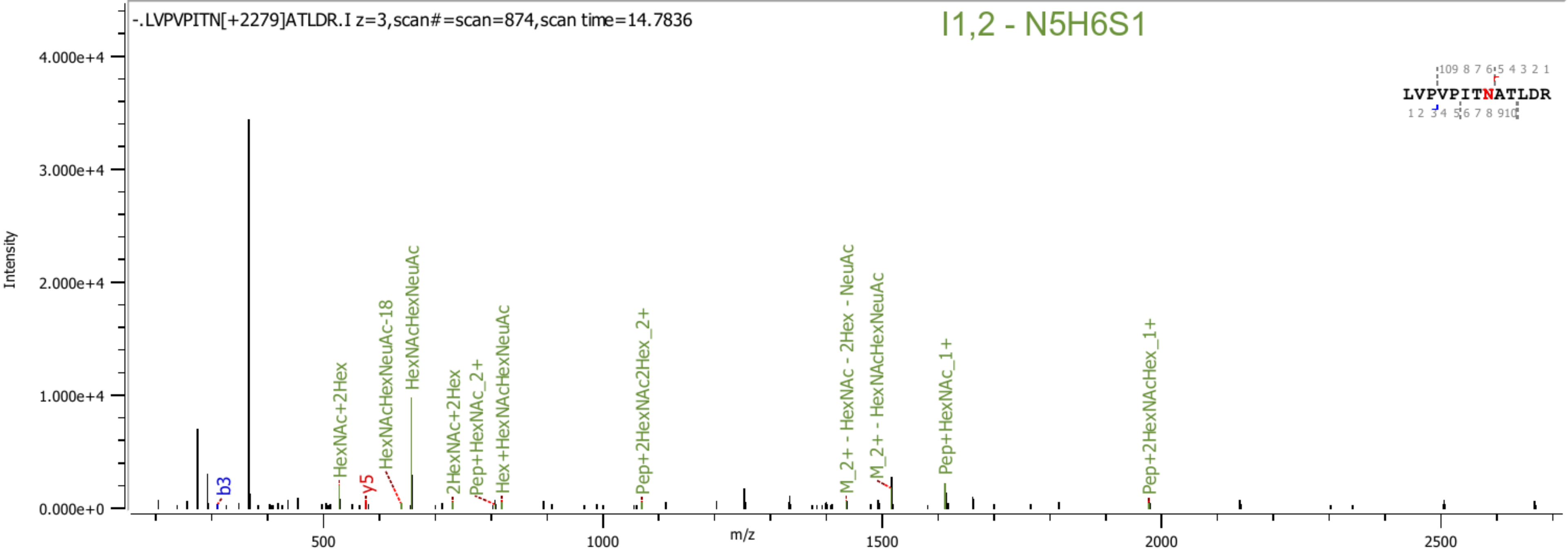
-.LVPVPITN[+2279]ATLDR.I z=3,scan#=scan=874,scan time=14.7836
I1,2 - N5H6S1
LVPVPITNATLDR
Intensity
m/z
b3
y5
HexNAc+2Hex
HexNAcHexNeuAc-18
HexNAcHexNeuAc
2HexNAc+2Hex
Pep+HexNAc_2+
Hex+HexNAcHexNeuAc
Pep+2HexNAc2Hex_2+
M_2+ - HexNAc - 2Hex - NeuAc
M_2+ - HexNAcHexNeuAc
Pep+HexNAc_1+
Pep+2HexNAcHex_1+

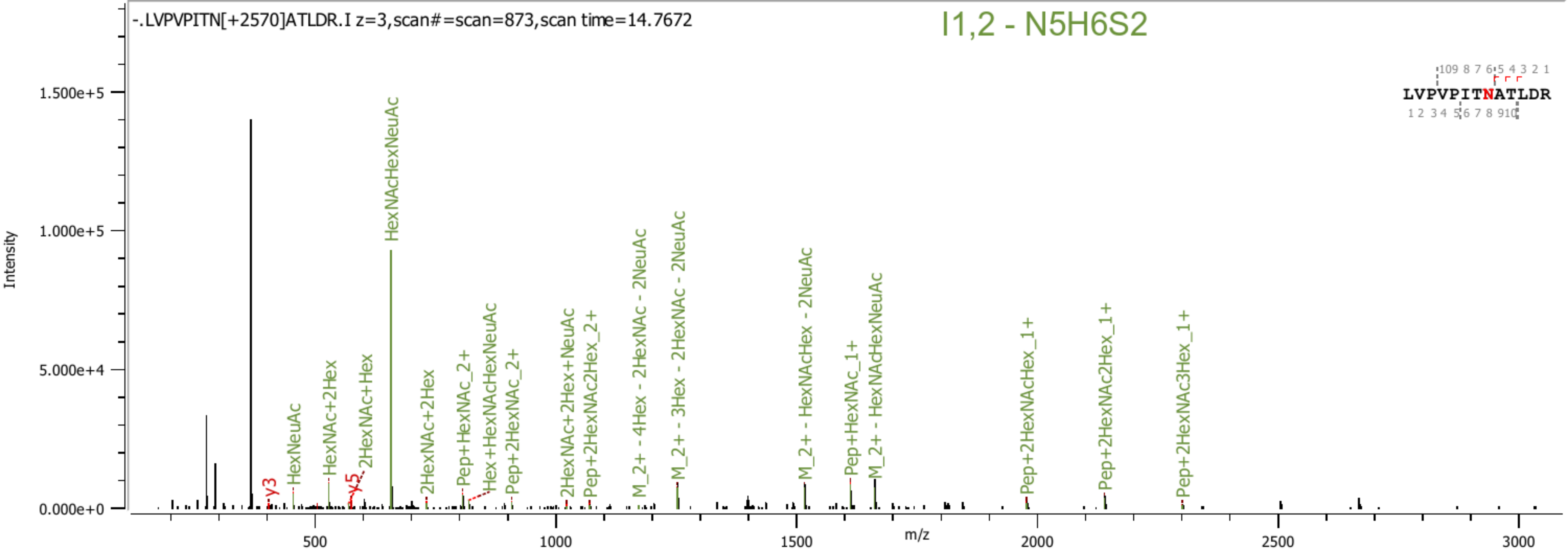

-.LVPVPITN[+2570]ATLDR.I z=3,scan#=scan=873,scan time=14.7672
I1,2 - N5H6S2
LVPVPITNATLDR
Intensity
m/z
y3
HexNeuAc
HexNAc+2Hex
y5
2HexNAc+Hex
HexNAcHexNeuAc
2HexNAc+2Hex
Pep+HexNAc_2+
Hex+HexNAcHexNeuAc
Pep+2HexNAc_2+
2HexNAc+2Hex+NeuAc
Pep+2HexNAc2Hex_2+
M_2+ - 4Hex - 2HexNAc - 2NeuAc
M_2+ - 3Hex - 2HexNAc - 2NeuAc
M_2+ - HexNAcHex - 2NeuAc
Pep+HexNAc_1+
M_2+ - HexNAcHexNeuAc
Pep+2HexNAcHex_1+
Pep+2HexNAc2Hex_1+
Pep+2HexNAc3Hex_1+

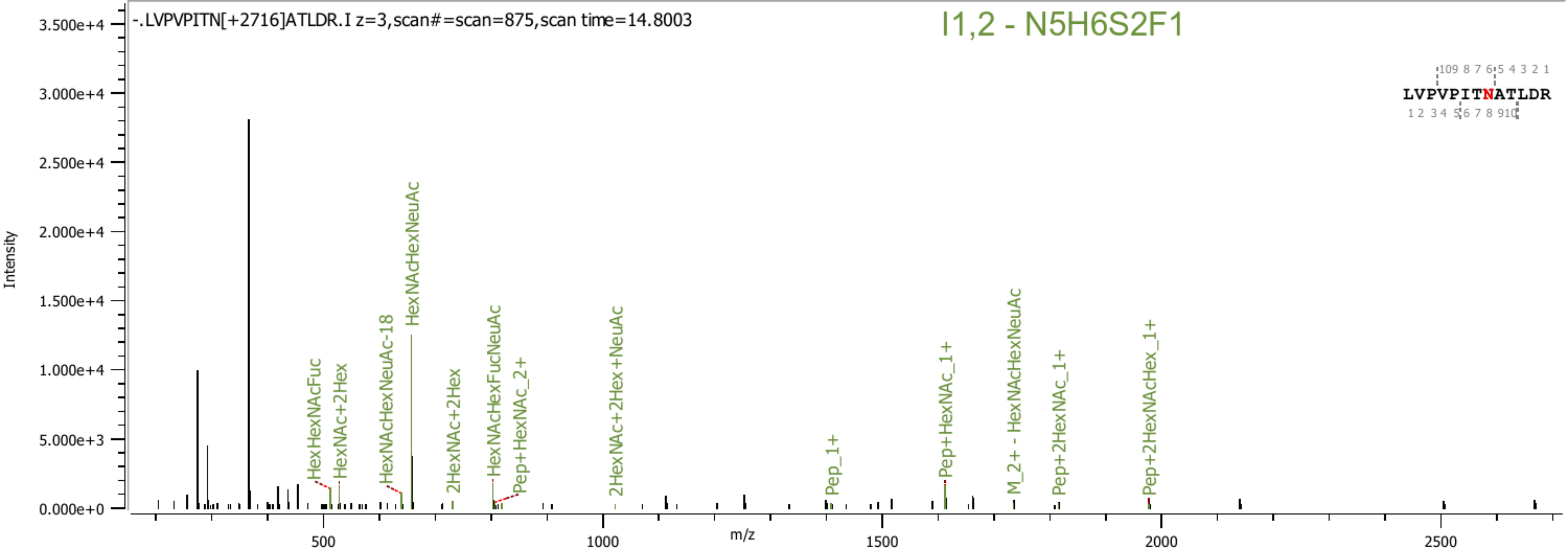

-.LVPVPITN[+2716]ATLDR.I z=3,scan#=scan=875,scan time=14.8003
I1,2 - N5H6S2F1
LVPVPITNATLDR
Intensity
m/z
HexHexNAcFuc
HexNAc+2Hex
HexNAcHexNeuAc-18
HexNAcHexNeuAc
2HexNAc+2Hex
HexNAcHexFucNeuAc
Pep+HexNAc_2+
2HexNAc+2Hex+NeuAc
Pep_1+
Pep+HexNAc_1+
M_2+ - HexNAcHexNeuAc
Pep+2HexNAc_1+
Pep+2HexNAcHex_1+

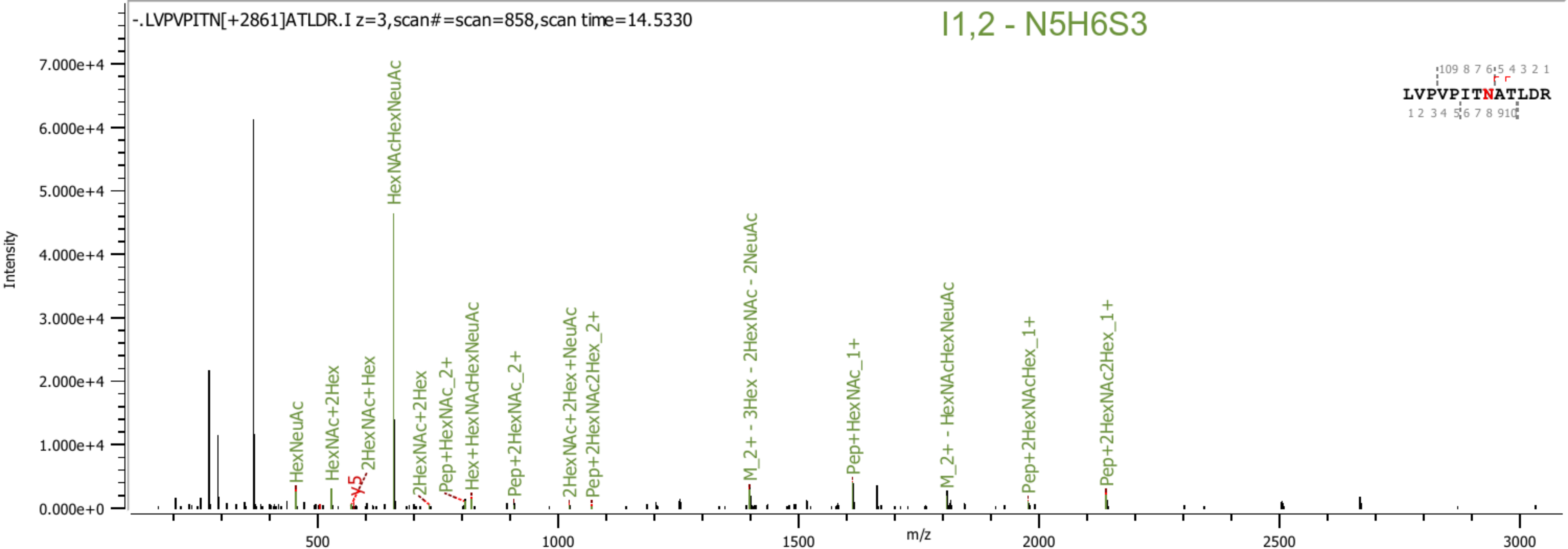

-.LVPVPITN[+2861]ATLDR.I z=3,scan#=scan=858,scan time=14.5330
I1,2 - N5H6S3
Intensity
7.000e+4
6.000e+4
5.000e+4
4.000e+4
3.000e+4
2.000e+4
1.000e+4
0.000e+0
HexNeuAc
HexNAc+2Hex
y5
2HexNAc+Hex
HexNAcHexNeuAc
2HexNAc+2Hex
Pep+HexNAc_2+
Hex+HexNAcHexNeuAc
Pep+2HexNAc_2+
2HexNAc+2Hex+NeuAc
Pep+2HexNAc2Hex_2+
M_2+ - 3Hex - 2HexNAc - 2NeuAc
Pep+HexNAc_1+
M_2+ - HexNAcHexNeuAc
Pep+2HexNAcHex_1+
Pep+2HexNAc2Hex_1+
LVPVPITNATLDR
500
1000
1500
2000
2500
3000
m/z

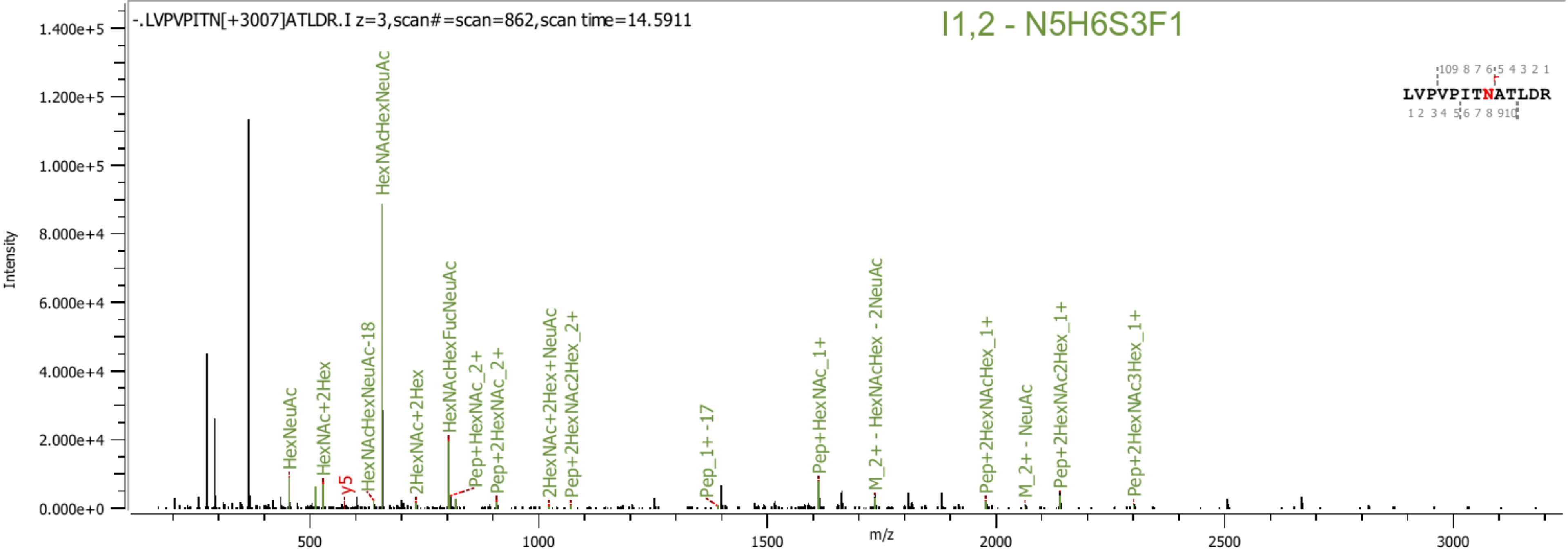

-.LVPVPITN[+3007]ATLDR.I z=3,scan#=scan=862,scan time=14.5911
I1,2 - N5H6S3F1
LVPVPITNATLDR
Intensity
m/z
HexNeuAc
HexNAc+2Hex
y5
HexNAcHexNeuAc-18
HexNAcHexNeuAc
2HexNAc+2Hex
HexNAcHexFucNeuAc
Pep+HexNAc_2+
Pep+2HexNAc_2+
2HexNAc+2Hex+NeuAc
Pep+2HexNAc2Hex_2+
Pep_1+ -17
Pep+HexNAc_1+
M_2+ - HexNAcHex - 2NeuAc
Pep+2HexNAcHex_1+
M_2+ - NeuAc
Pep+2HexNAc2Hex_1+
Pep+2HexNAc3Hex_1+

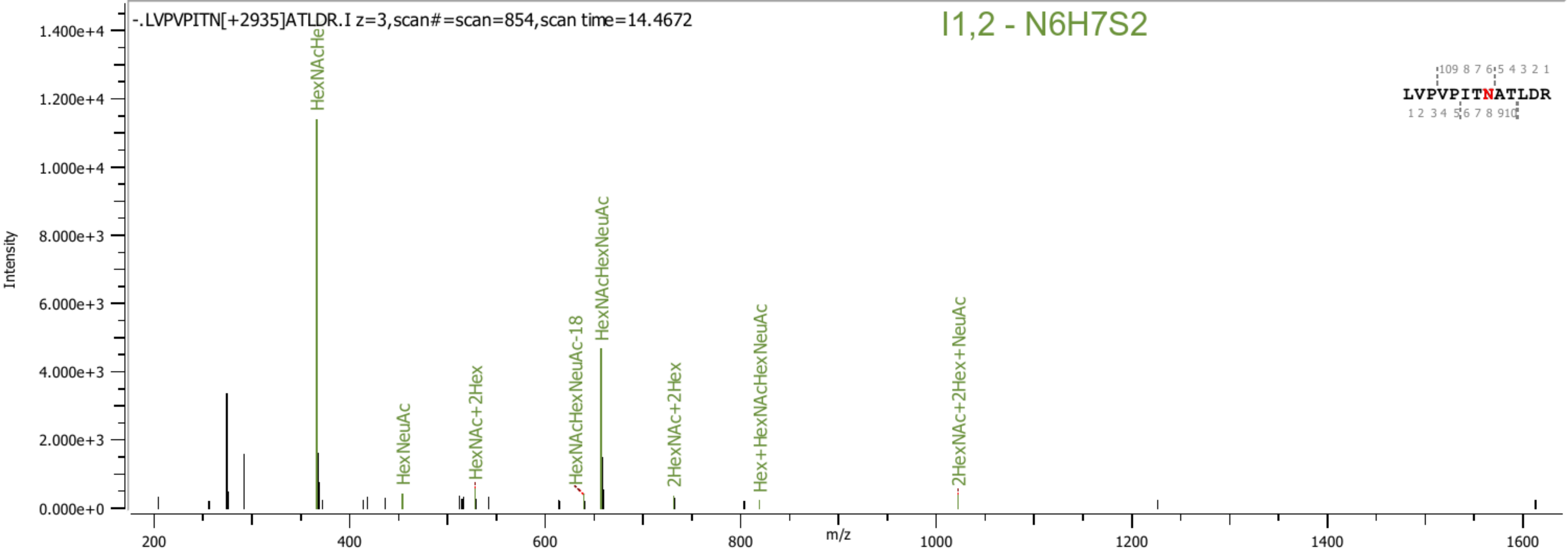

-.LVPVPITN[+2935]ATLDR.I z=3,scan#=scan=854,scan time=14.4672
I1,2 - N6H7S2
LVPVPITNATLDR
HexNAcHe
HexNeuAc
HexNAc+2Hex
HexNAcHexNeuAc-18
HexNAcHexNeuAc
2HexNAc+2Hex
Hex+HexNAcHexNeuAc
2HexNAc+2Hex+NeuAc
Intensity
m/z

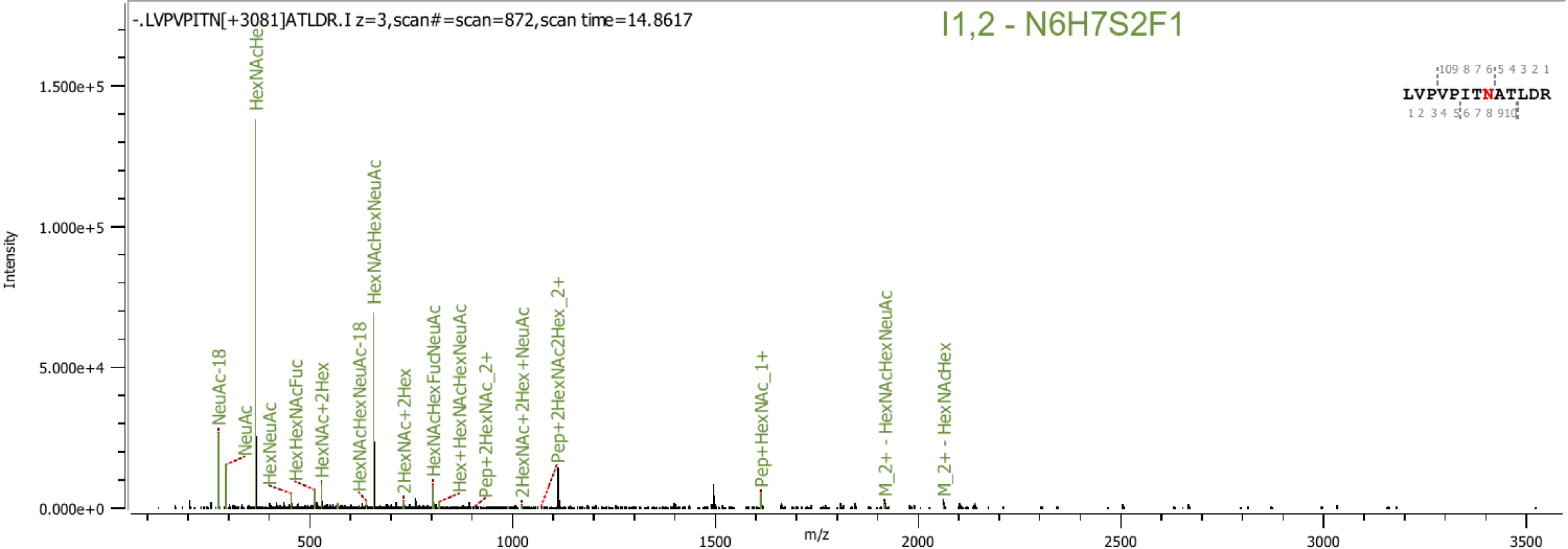

-.LVPVPITN[+3081]ATLDR.I z=3,scan#=scan=872,scan time=14.8617
I1,2 - N6H7S2F1
LVPVPITNATLDR
Intensity
1.500e+5
1.000e+5
5.000e+4
0.000e+0
m/z
500
1000
1500
2000
2500
3000
3500
HexNAcHe
HexNAcHexNeuAc
NeuAc-18
NeuAc
HexNeuAc
HexHexNAcFuc
HexNAc+2Hex
HexNAcHexNeuAc-18
2HexNAc+2Hex
HexNAcHexFucNeuAc
Hex+HexNAcHexNeuAc
Pep+2HexNAc_2+
2HexNAc+2Hex+NeuAc
Pep+2HexNAc2Hex_2+
Pep+HexNAc_1+
M_2+ - HexNAcHexNeuAc
M_2+ - HexNAcHex

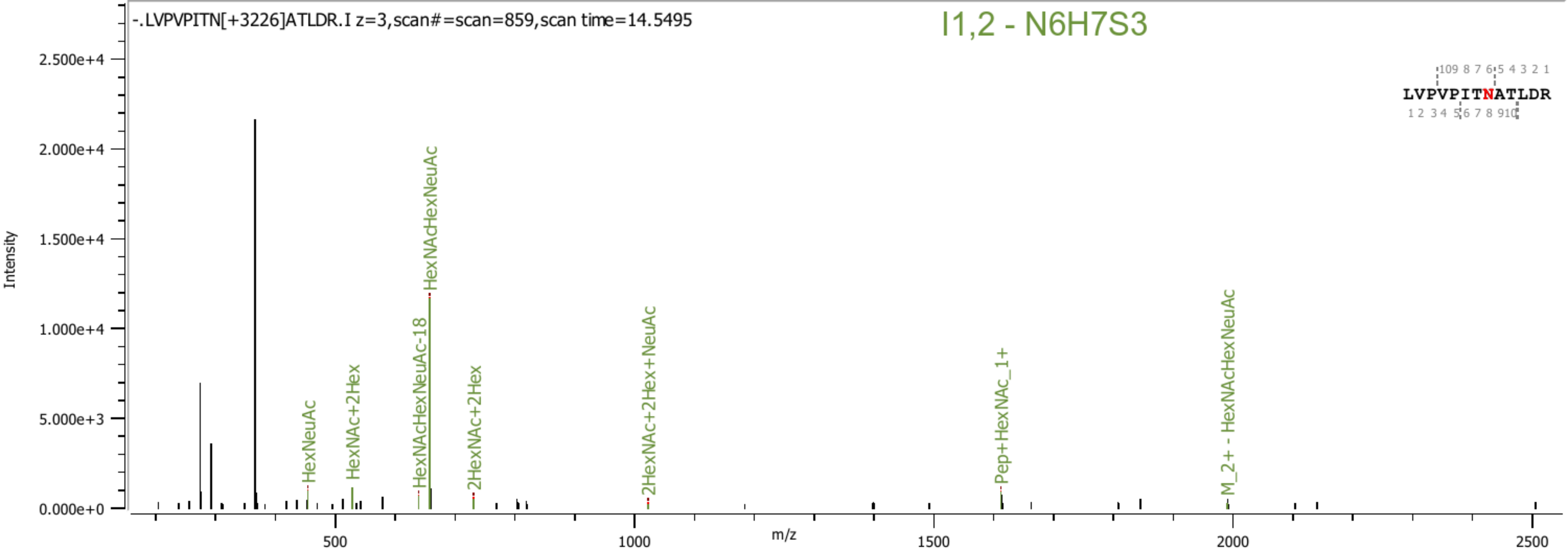

-.LVPVPITN[+3226]ATLDR.I z=3,scan#=scan=859,scan time=14.5495
I1,2 - N6H7S3
109 8 7 6 5 4 3 2 1
LVPVPITNATLDR
1 2 3 4 5 6 7 8 9 10
Intensity
2.500e+4
2.000e+4
1.500e+4
1.000e+4
5.000e+3
0.000e+0
HexNeuAc
HexNAc+2Hex
HexNAcHexNeuAc-18
HexNAcHexNeuAc
2HexNAc+2Hex
2HexNAc+2Hex+NeuAc
Pep+HexNAc_1+
M_2+ - HexNAcHexNeuAc
500
1000
1500
2000
2500
m/z

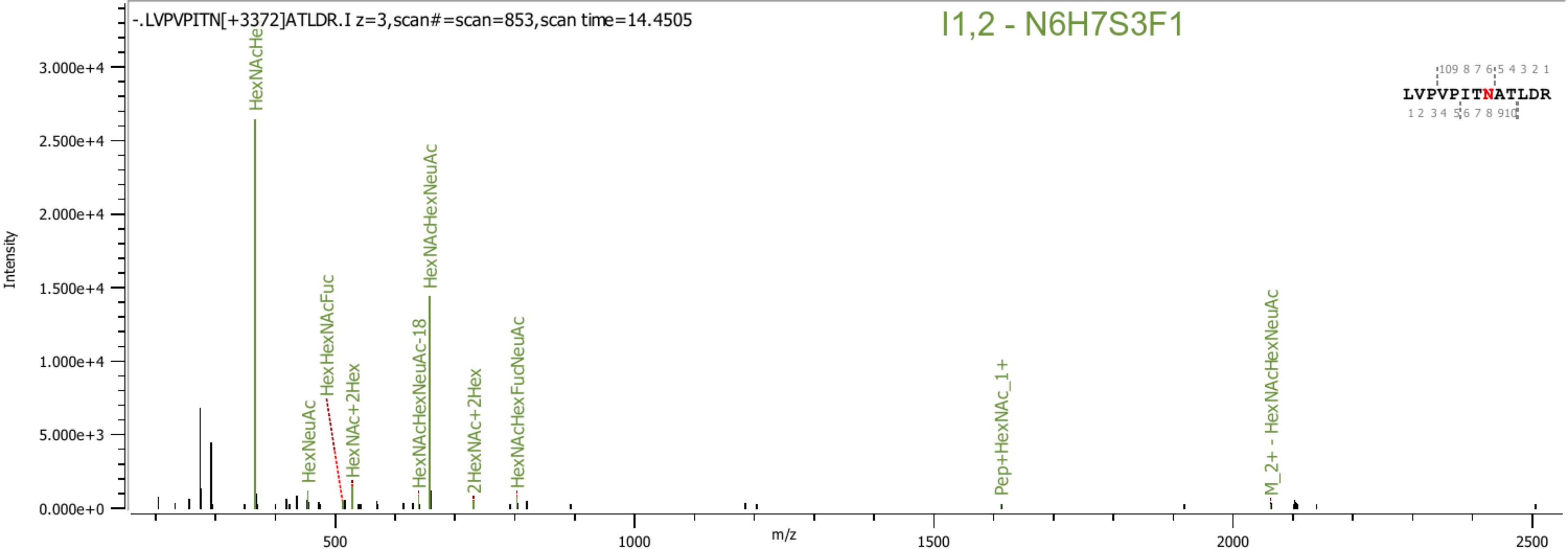
-.LVPVPITN[+3372]ATLDR.I z=3,scan#=scan=853,scan time=14.4505
I1,2 - N6H7S3F1
LVPVPITNATLDR
Intensity
m/z
HexNAcHex
HexNeuAc
HexHexNAcFuc
HexNAc+2Hex
HexNAcHexNeuAc-18
HexNAcHexNeuAc
2HexNAc+2Hex
HexNAcHexFucNeuAc
Pep+HexNAc_1+
M_2+ - HexNAcHexNeuAc

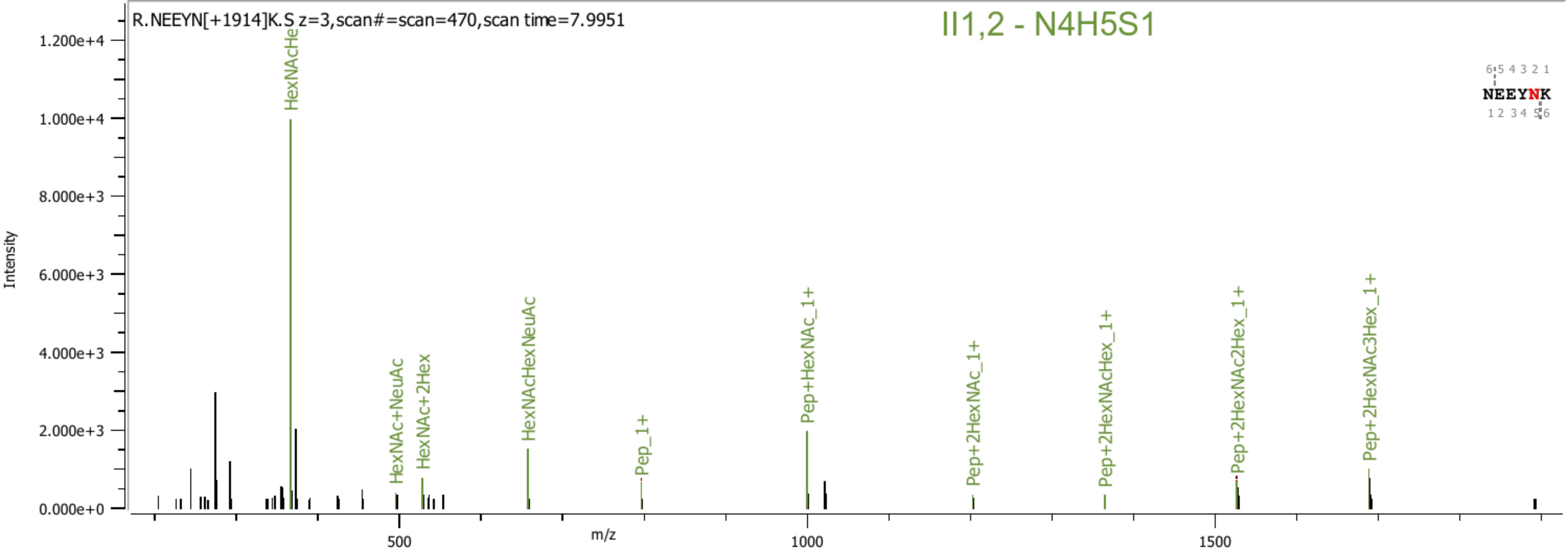
R.NEEYN[+1914]K.S z=3,scan#=scan=470,scan time=7.9951
II1,2 - N4H5S1
NEEYNK
HexNAcHe
HexNAc+NeuAc
HexNAc+2Hex
HexNAcHexNeuAc
Pep_1+
Pep+HexNAc_1+
Pep+2HexNAc_1+
Pep+2HexNAcHex_1+
Pep+2HexNAc2Hex_1+
Pep+2HexNAc3Hex_1+
Intensity
m/z
0.000e+0
2.000e+3
4.000e+3
6.000e+3
8.000e+3
1.000e+4
1.200e+4
500
1000
1500

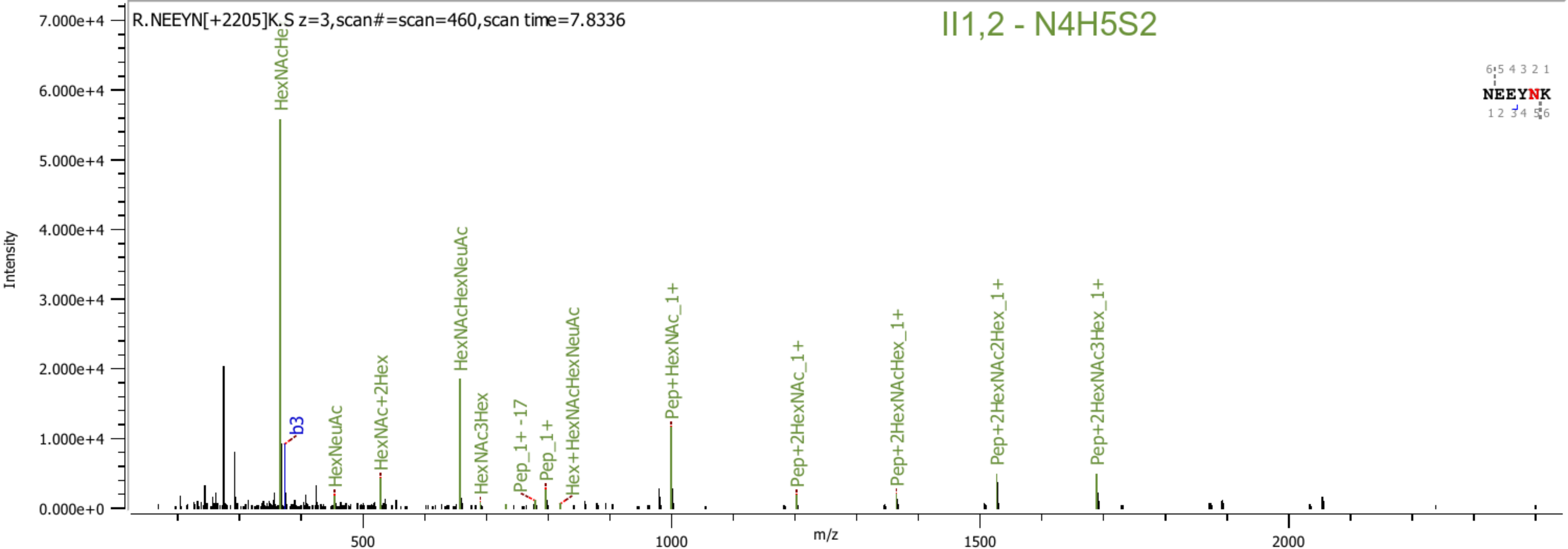
R.NEEYN[+2205]K.S z=3,scan#=scan=460,scan time=7.8336
II1,2 - N4H5S2
6 5 4 3 2 1
NEEYNK
1 2 3 4 5 6
Intensity
7.000e+4
6.000e+4
5.000e+4
4.000e+4
3.000e+4
2.000e+4
1.000e+4
0.000e+0
HexNAcHex
b3
HexNeuAc
HexNAc+2Hex
HexNAcHexNeuAc
HexNAc3Hex
Pep_1+ -17
Pep_1+
Hex+HexNAcHexNeuAc
Pep+HexNAc_1+
Pep+2HexNAc_1+
Pep+2HexNAcHex_1+
Pep+2HexNAc2Hex_1+
Pep+2HexNAc3Hex_1+
500
1000
1500
2000
m/z

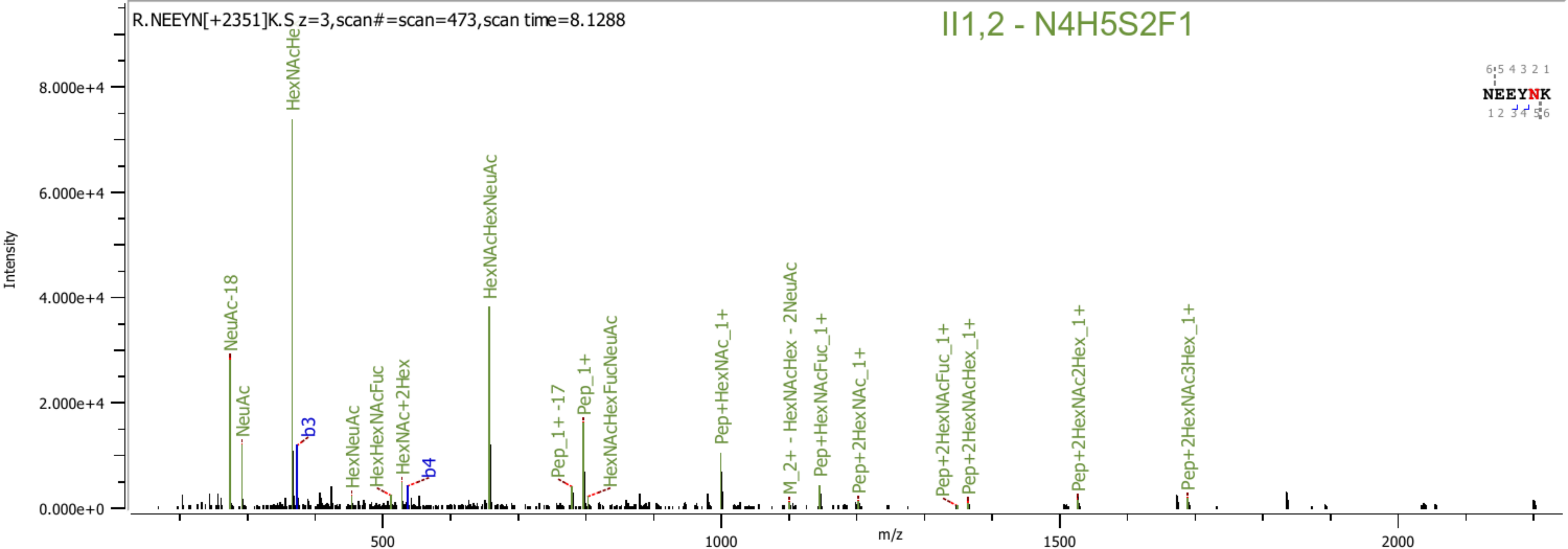

R.NEEYN[+2351]K.S z=3,scan#=scan=473,scan time=8.1288
II1,2 - N4H5S2F1
NEEYNK
Intensity
m/z
8.000e+4
6.000e+4
4.000e+4
2.000e+4
0.000e+0
500
1000
1500
2000
NeuAc-18
NeuAc
HexNAcHe
b3
HexNeuAc
HexHexNAcFuc
HexNAc+2Hex
b4
HexNAcHexNeuAc
Pep_1+ -17
Pep_1+
HexNAcHexFucNeuAc
Pep+HexNAc_1+
M_2+ - HexNAcHex - 2NeuAc
Pep+HexNAcFuc_1+
Pep+2HexNAc_1+
Pep+2HexNAcFuc_1+
Pep+2HexNAcHex_1+
Pep+2HexNAc2Hex_1+
Pep+2HexNAc3Hex_1+

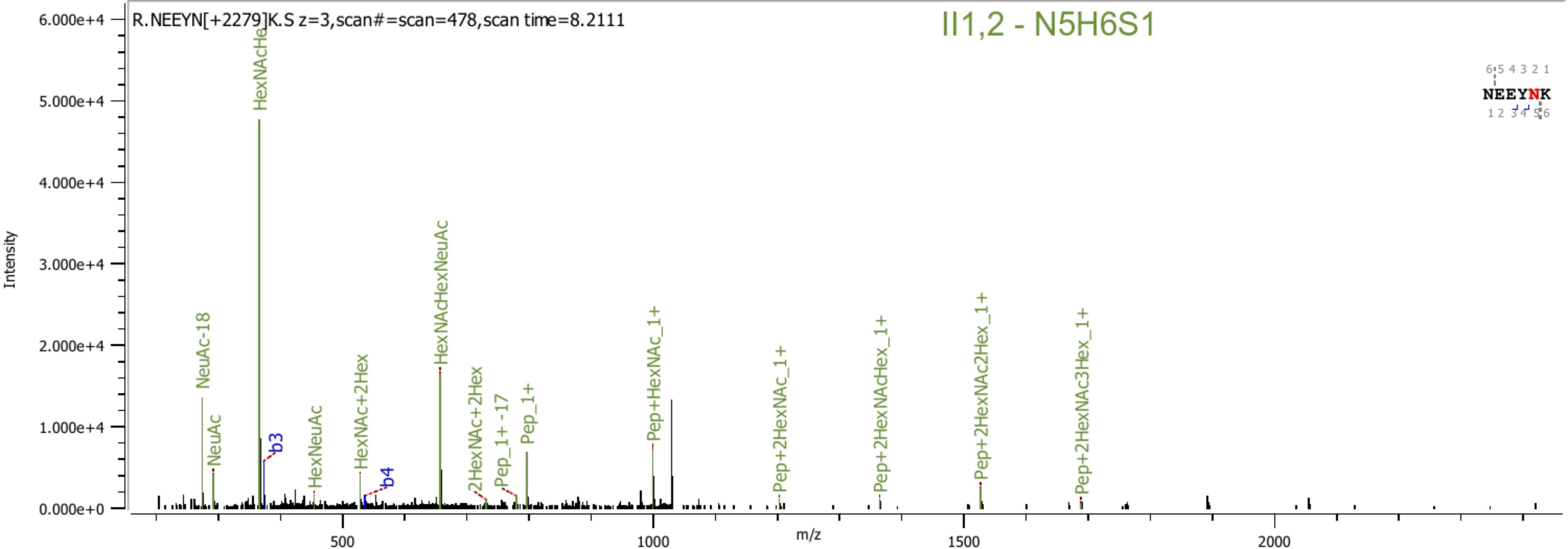
R.NEEYN[+2279]K.S z=3,scan#=scan=478,scan time=8.2111
II1,2 - N5H6S1
Intensity
m/z
NeuAc-18
NeuAc
HexNAcHe
b3
HexNeuAc
HexNAc+2Hex
b4
HexNAcHexNeuAc
2HexNAc+2Hex
Pep_1+ -17
Pep_1+
Pep+HexNAc_1+
Pep+2HexNAc_1+
Pep+2HexNAcHex_1+
Pep+2HexNAc2Hex_1+
Pep+2HexNAc3Hex_1+
NEEYNK

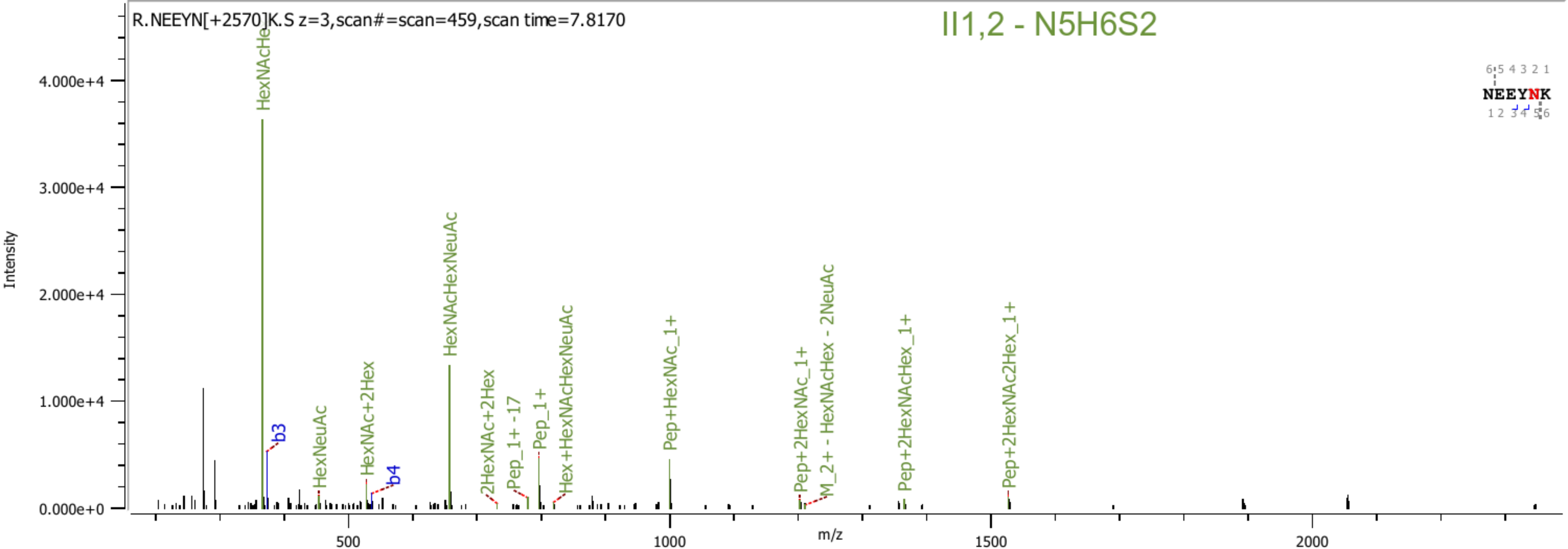
R.NEEYN[+2570]K.S z=3,scan#=scan=459,scan time=7.8170
II1,2 - N5H6S2
Intensity
m/z
4.000e+4
3.000e+4
2.000e+4
1.000e+4
0.000e+0
500
1000
1500
2000
HexNAcHe
b3
HexNeuAc
HexNAc+2Hex
b4
HexNAcHexNeuAc
2HexNAc+2Hex
Pep_1+ -17
Pep_1+
Hex+HexNAcHexNeuAc
Pep+HexNAc_1+
Pep+2HexNAc_1+
M_2+ - HexNAcHex - 2NeuAc
Pep+2HexNAcHex_1+
Pep+2HexNAc2Hex_1+
NEEYNK

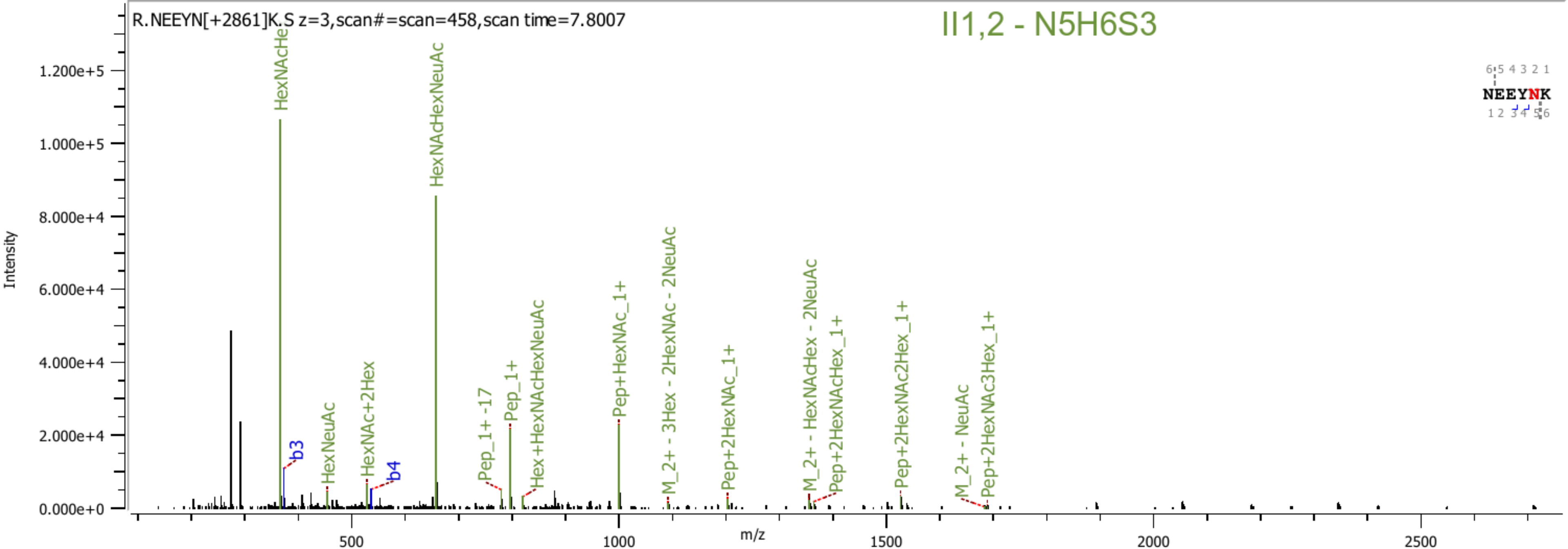

R.NEEYN[+2861]K.S z=3,scan#=scan=458,scan time=7.8007
II1,2 - N5H6S3
NEEYNK
HexNAcHex
HexNAcHexNeuAc
b3
HexNeuAc
HexNAc+2Hex
b4
Pep_1+ -17
Pep_1+
Hex+HexNAcHexNeuAc
Pep+HexNAc_1+
M_2+ - 3Hex - 2HexNAc - 2NeuAc
Pep+2HexNAc_1+
M_2+ - HexNAcHex - 2NeuAc
Pep+2HexNAcHex_1+
Pep+2HexNAc2Hex_1+
M_2+ - NeuAc
Pep+2HexNAc3Hex_1+
Intensity
m/z

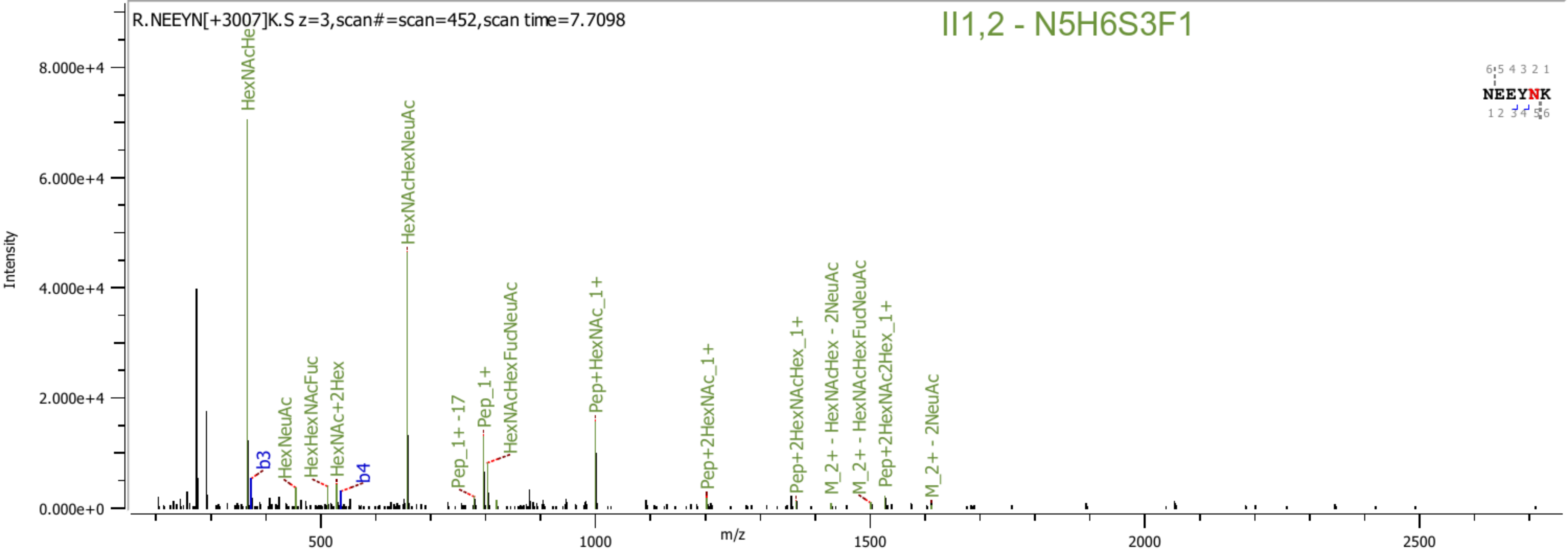

R.NEEYN[+3007]K.S z=3,scan#=scan=452,scan time=7.7098
II1,2 - N5H6S3F1
NEEYNK
Intensity
m/z
HexNAcHex
HexNAcHexNeuAc
HexNeuAc
HexHexNAcFuc
HexNAc+2Hex
b3
b4
Pep_1+ -17
Pep_1+
HexNAcHexFucNeuAc
Pep+HexNAc_1+
Pep+2HexNAc_1+
Pep+2HexNAcHex_1+
M_2+ - HexNAcHex - 2NeuAc
M_2+ - HexNAcHexFucNeuAc
Pep+2HexNAc2Hex_1+
M_2+ - 2NeuAc

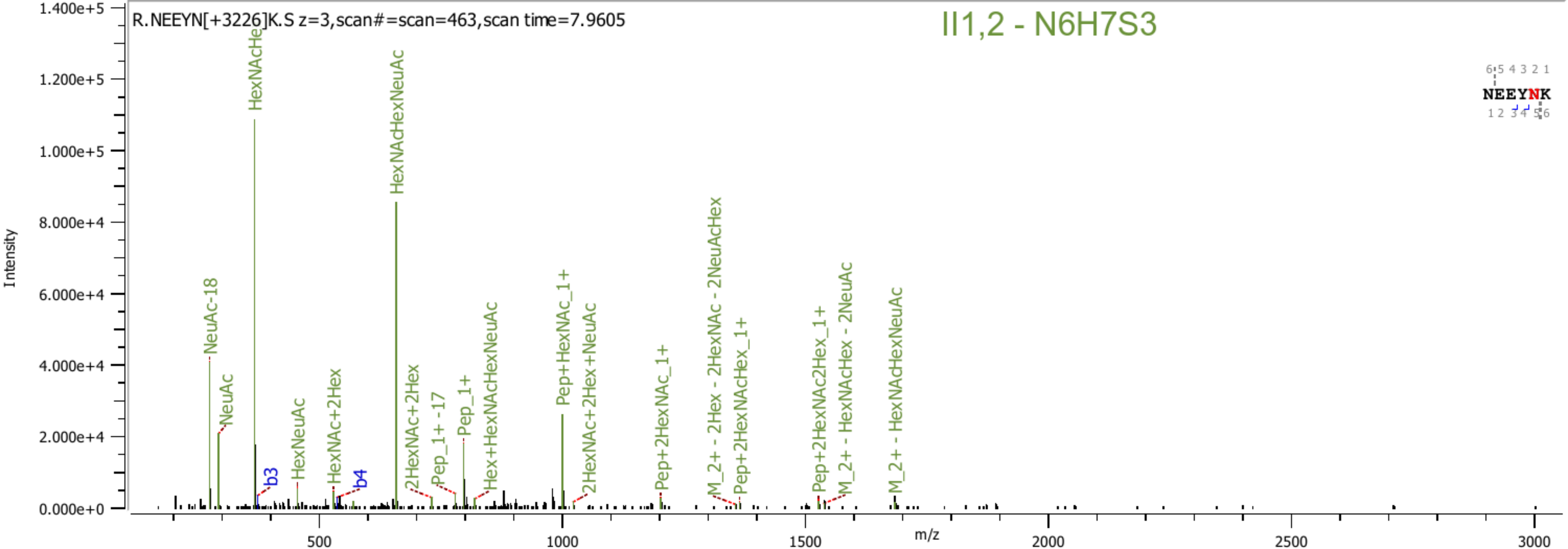

R.NEEYN[+3226]K.S z=3,scan#=scan=463,scan time=7.9605
II1,2 - N6H7S3
NEEYNK
Intensity
m/z
NeuAc-18
NeuAc
HexNAcHex
b3
HexNeuAc
HexNAc+2Hex
b4
HexNAcHexNeuAc
2HexNAc+2Hex
Pep_1+ -17
Pep_1+
Hex+HexNAcHexNeuAc
Pep+HexNAc_1+
2HexNAc+2Hex+NeuAc
Pep+2HexNAc_1+
M_2+ - 2Hex - 2HexNAc - 2NeuAcHex
Pep+2HexNAcHex_1+
Pep+2HexNAc2Hex_1+
M_2+ - HexNAcHex - 2NeuAc
M_2+ - HexNAcHexNeuAc

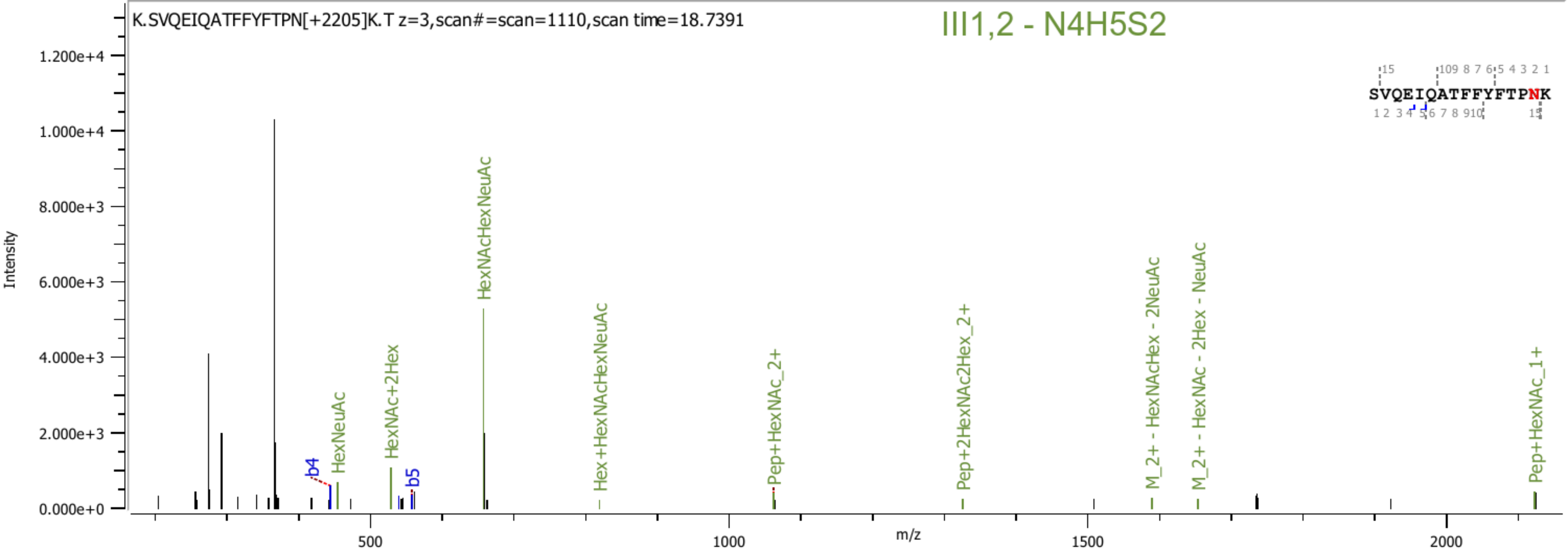

K.SVQEIQATFFYFTPN[+2205]K.T z=3,scan#=scan=1110,scan time=18.7391
III1,2 - N4H5S2
SVQEIQATFFYFTPNK
Intensity
m/z
HexNAcHexNeuAc
Hex+HexNAcHexNeuAc
HexNAc+2Hex
HexNeuAc
b4
b5
Pep+HexNAc_2+
Pep+2HexNAc2Hex_2+
M_2+ - HexNAcHex - 2NeuAc
M_2+ - HexNAc - 2Hex - NeuAc
Pep+HexNAc_1+

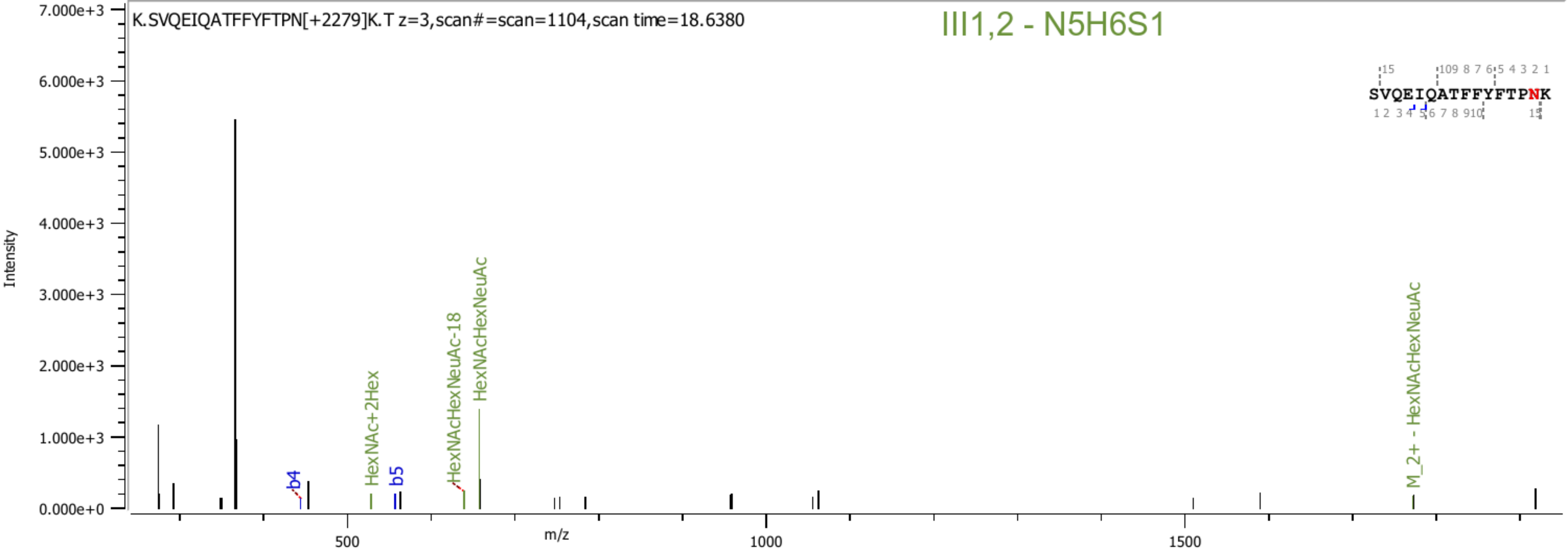

K.SVQEIQATFFYFTPN[+2279]K.T z=3,scan#=scan=1104,scan time=18.6380
III1,2 - N5H6S1
SVQEIQATFFYFTPNK
Intensity
m/z
b4
HexNAc+2Hex
b5
HexNAcHexNeuAc-18
HexNAcHexNeuAc
M_2+ - HexNAcHexNeuAc

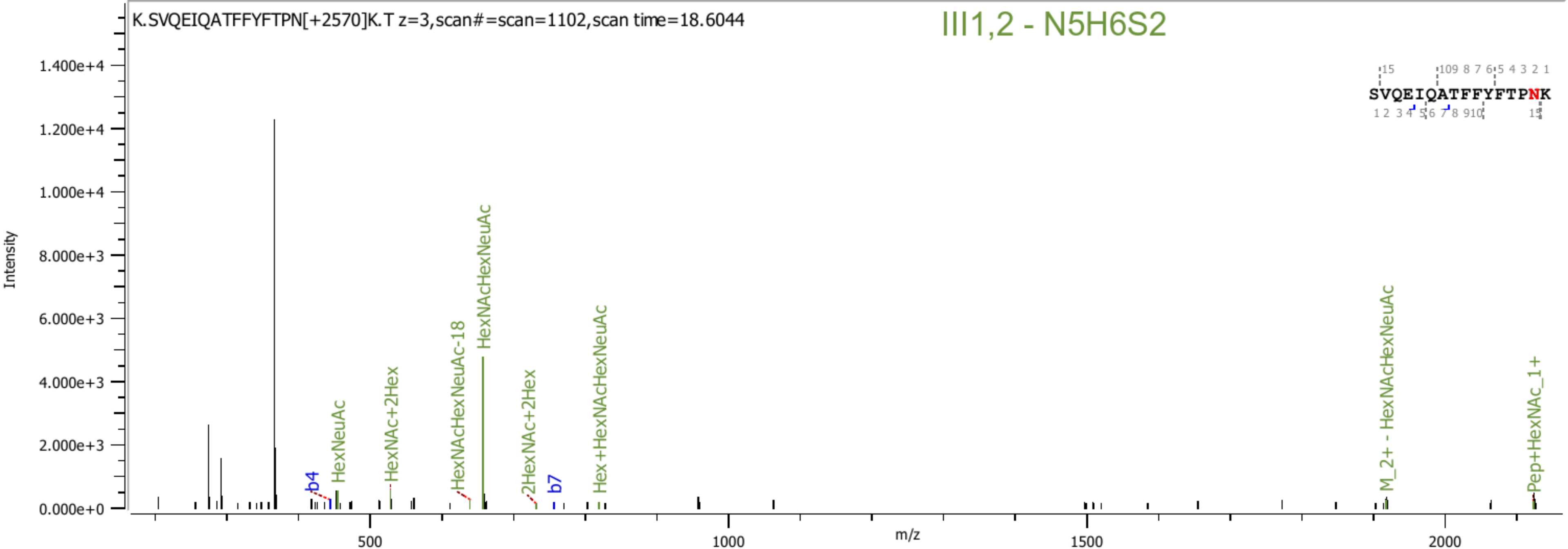
K.SVQEIQATFFYFTPN[+2570]K.T z=3,scan#=scan=1102,scan time=18.6044
III1,2 - N5H6S2
SVQEIQATFFYFTPNK
Intensity
m/z
b4
HexNeuAc
HexNAc+2Hex
HexNAcHexNeuAc-18
HexNAcHexNeuAc
2HexNAc+2Hex
b7
Hex+HexNAcHexNeuAc
M_2+ - HexNAcHexNeuAc
Pep+HexNAc_1+

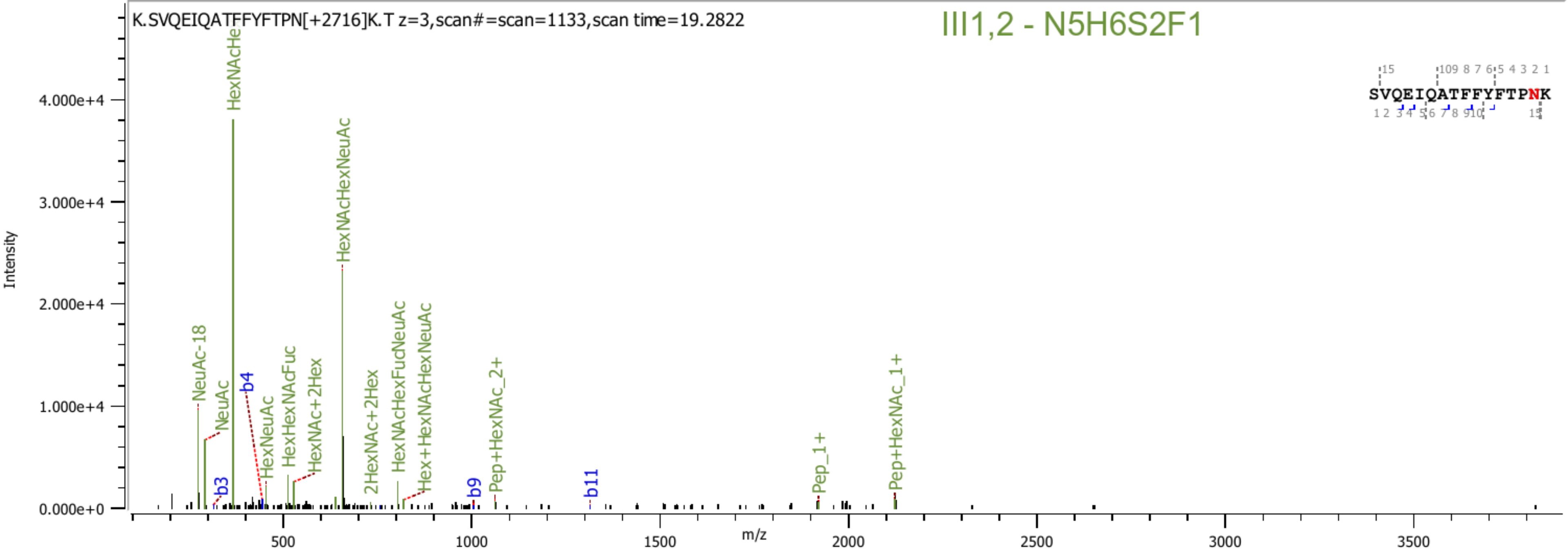

K.SVQEIQATFFYFTPN[+2716]K.T z=3,scan#=scan=1133,scan time=19.2822
III1,2 - N5H6S2F1
SVQEIQATFFYFTPNK
Intensity
m/z
HexNAcHe
HexNAcHexNeuAc
NeuAc-18
NeuAc
b3
b4
HexNeuAc
HexHexNAcFuc
HexNAc+2Hex
2HexNAc+2Hex
HexNAcHexFucNeuAc
Hex+HexNAcHexNeuAc
b9
Pep+HexNAc_2+
b11
Pep_1+
Pep+HexNAc_1+

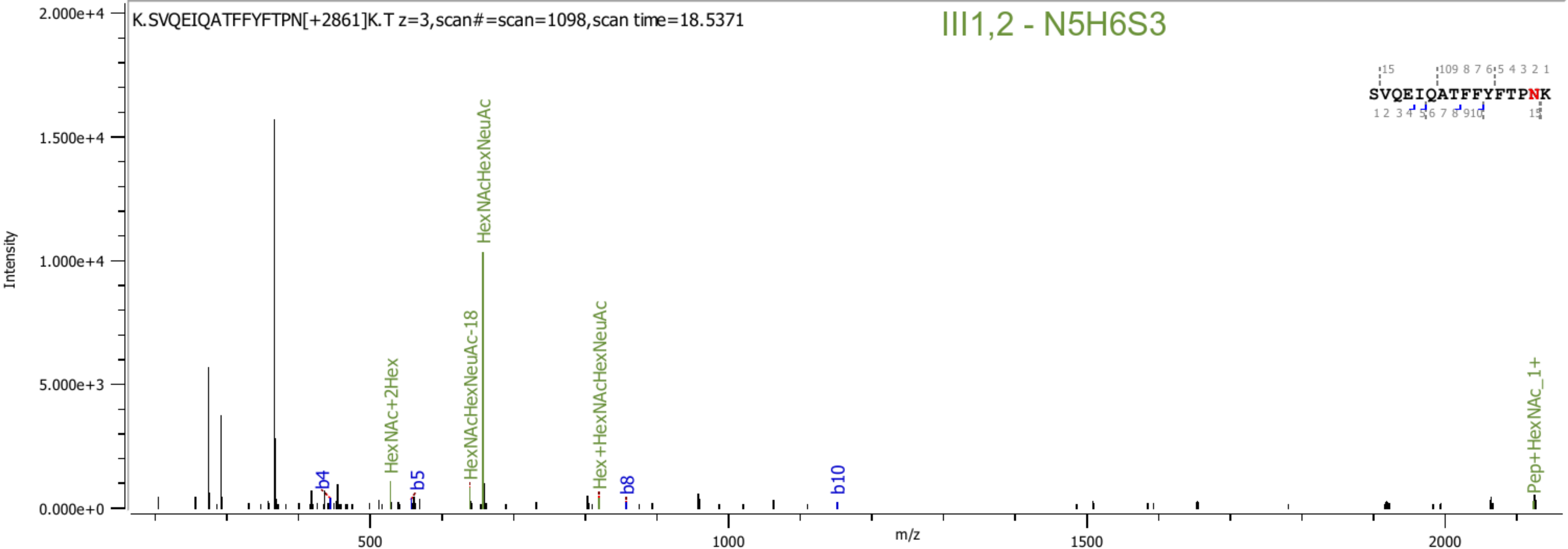
K.SVQEIQATFFYFTPN[+2861]K.T z=3,scan#=scan=1098,scan time=18.5371
III1,2 - N5H6S3
SVQEIQATFFYFTPNK
Intensity
m/z
HexNAcHexNeuAc
HexNAcHexNeuAc-18
HexNAc+2Hex
Hex+HexNAcHexNeuAc
Pep+HexNAc_1+
b4
b5
b8
b10
2.000e+4
1.500e+4
1.000e+4
5.000e+3
0.000e+0
500
1000
1500
2000

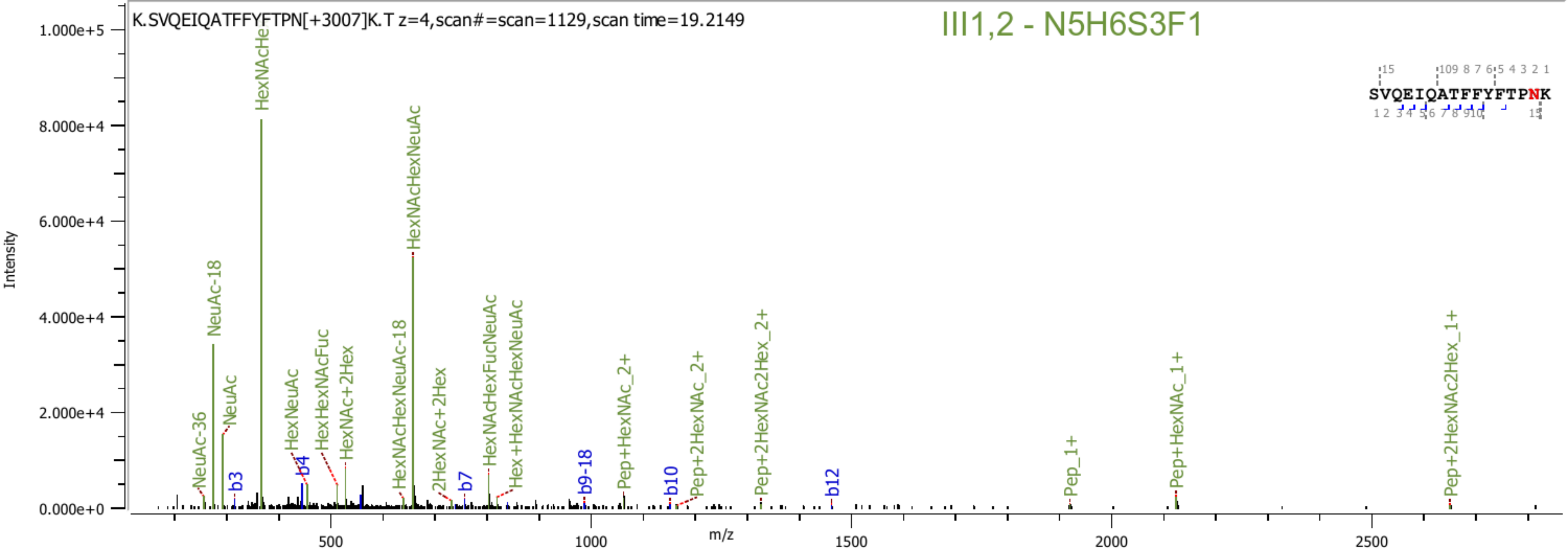
K.SVQEIQATFFYFTPN[+3007]K.T z=4,scan#=scan=1129,scan time=19.2149
III1,2 - N5H6S3F1
SVQEIQATFFYFTPNK
Intensity
m/z
HexNAcHex
HexNAcHexNeuAc
NeuAc-18
NeuAc
NeuAc-36
b3
b4
HexNeuAc
HexHexNAcFuc
HexNAc+2Hex
HexNAcHexNeuAc-18
2HexNAc+2Hex
b7
HexNAcHexFucNeuAc
Hex+HexNAcHexNeuAc
b9-18
Pep+HexNAc_2+
b10
Pep+2HexNAc_2+
Pep+2HexNAc2Hex_2+
b12
Pep_1+
Pep+HexNAc_1+
Pep+2HexNAc2Hex_1+

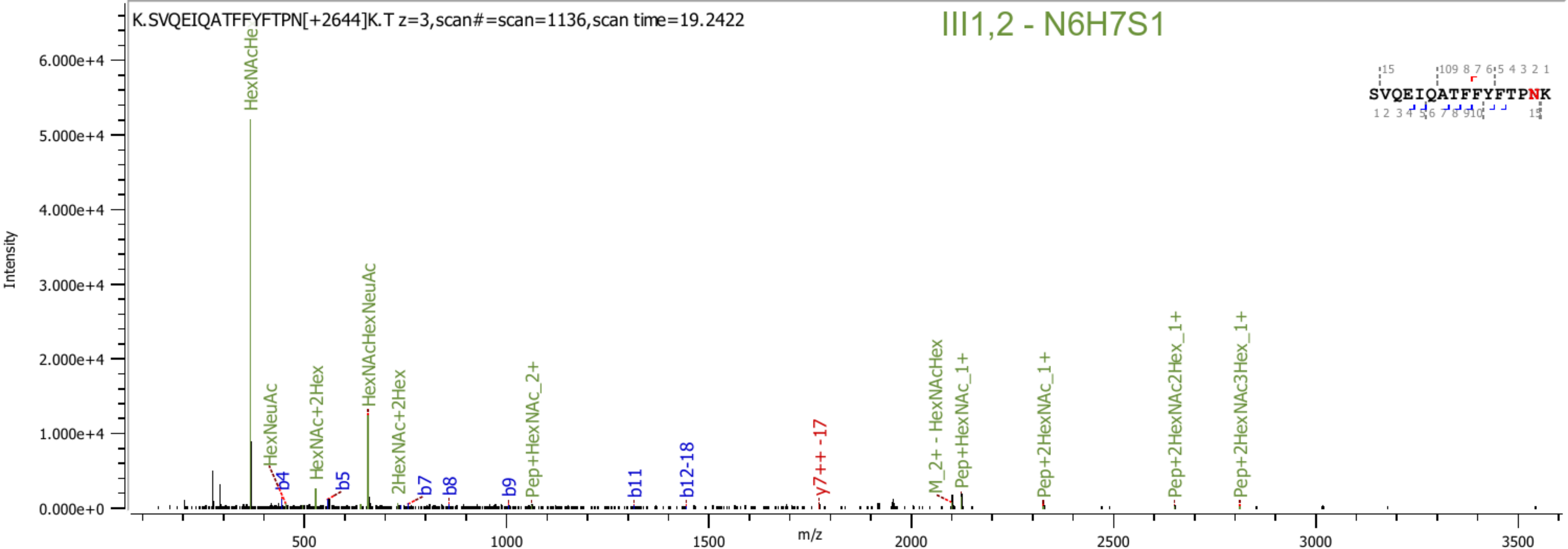

K.SVQEIQATFFYFTPN[+2644]K.T z=3,scan#=scan=1136,scan time=19.2422
III1,2 - N6H7S1
SVQEIQATFFYFTPNK
Intensity
m/z
HexNAcHex
HexNeuAc
HexNAc+2Hex
HexNAcHexNeuAc
2HexNAc+2Hex
Pep+HexNAc_2+
b4
b5
b7
b8
b9
b11
b12-18
y7++ -17
M_2+ - HexNAcHex
Pep+HexNAc_1+
Pep+2HexNAc_1+
Pep+2HexNAc2Hex_1+
Pep+2HexNAc3Hex_1+

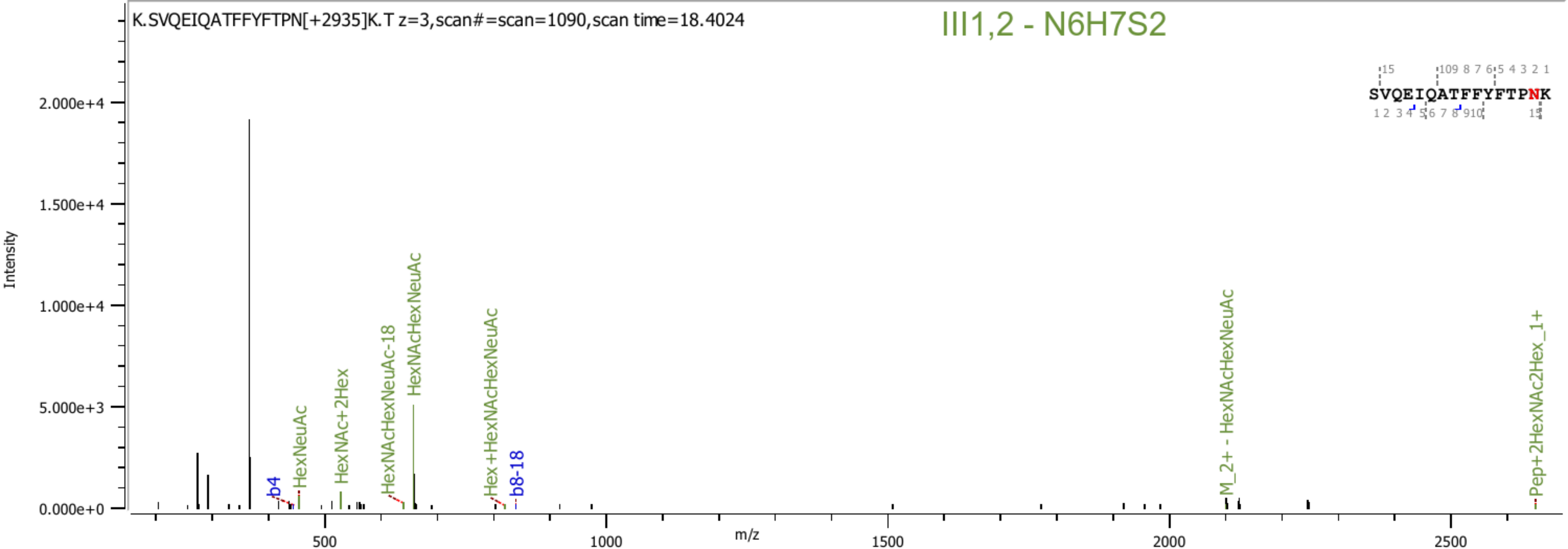
K.SVQEIQATFFYFTPN[+2935]K.T z=3,scan#=scan=1090,scan time=18.4024
III1,2 - N6H7S2
SVQEIQATFFYFTPNK
Intensity
2.000e+4
1.500e+4
1.000e+4
5.000e+3
0.000e+0
b4
HexNeuAc
HexNAc+2Hex
HexNAcHexNeuAc-18
HexNAcHexNeuAc
Hex+HexNAcHexNeuAc
b8-18
M_2+ - HexNAcHexNeuAc
Pep+2HexNAc2Hex_1+
m/z
500
1000
1500
2000
2500

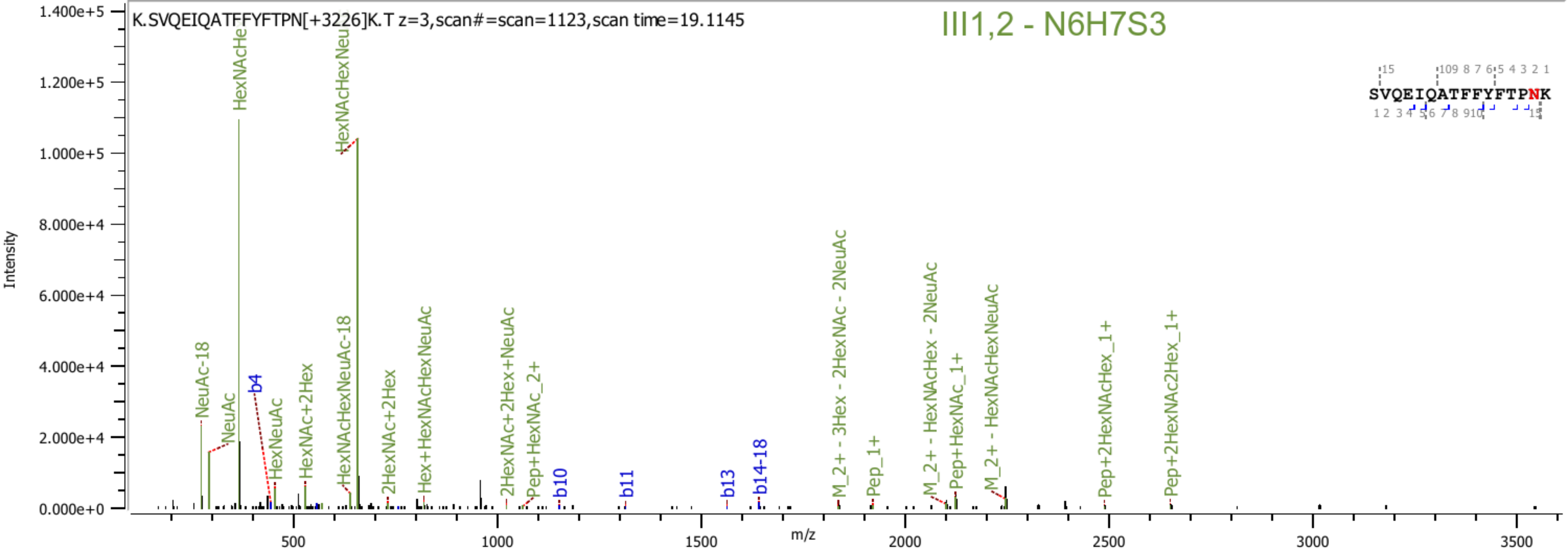
K.SVQEIQATFFYFTPN[+3226]K.T z=3,scan#=scan=1123,scan time=19.1145
III1,2 - N6H7S3
SVQEIQATFFYFTPNK
Intensity
m/z
NeuAc-18
NeuAc
HexNAcHe
b4
HexNeuAc
HexNAc+2Hex
HexNAcHexNeu
HexNAcHexNeuAc-18
2HexNAc+2Hex
Hex+HexNAcHexNeuAc
2HexNAc+2Hex+NeuAc
Pep+HexNAc_2+
b10
b11
b13
b14-18
M_2+ - 3Hex - 2HexNAc - 2NeuAc
Pep_1+
M_2+ - HexNAcHex - 2NeuAc
Pep+HexNAc_1+
M_2+ - HexNAcHexNeuAc
Pep+2HexNAcHex_1+
Pep+2HexNAc2Hex_1+

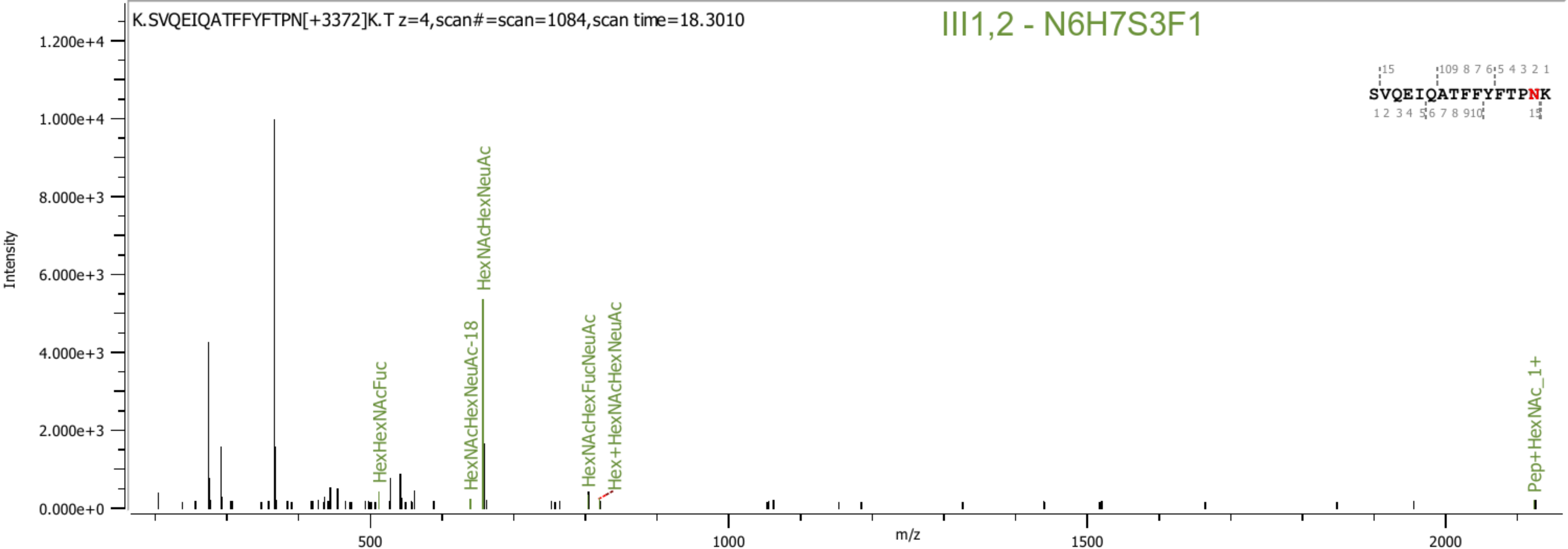
K.SVQEIQATFFYFTPN[+3372]K.T z=4,scan#=scan=1084,scan time=18.3010
III1,2 - N6H7S3F1
SVQEIQATFFYFTPNK
Intensity
m/z
HexHexNAcFuc
HexNAcHexNeuAc-18
HexNAcHexNeuAc
HexNAcHexFucNeuAc
Hex+HexNAcHexNeuAc
Pep+HexNAc_1+

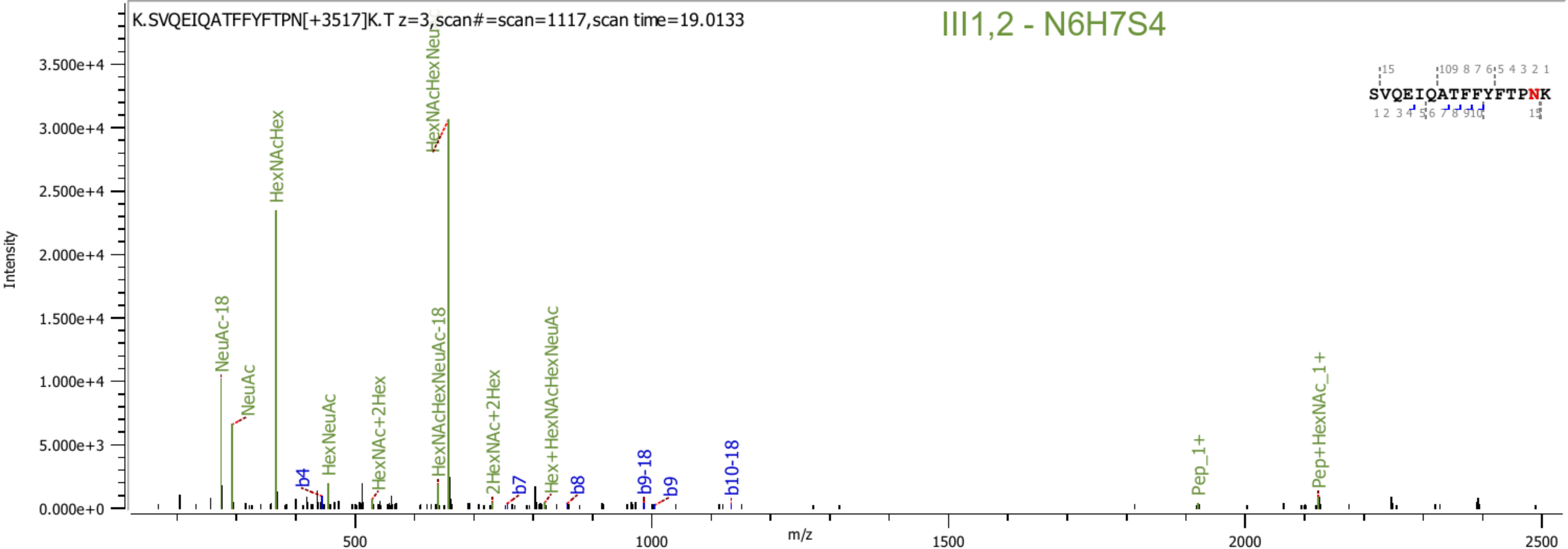
K.SVQEIQATFFYFTPN[+3517]K.T z=3,scan#=scan=1117,scan time=19.0133
III1,2 - N6H7S4
SVQEIQATFFYFTPNK
Intensity
m/z
NeuAc-18
NeuAc
HexNAcHex
b4
HexNeuAc
HexNAc+2Hex
HexNAcHexNeuAc-18
HexNAcHexNeuAc
2HexNAc+2Hex
b7
Hex+HexNAcHexNeuAc
b8
b9-18
b9
b10-18
Pep_1+
Pep+HexNAc_1+

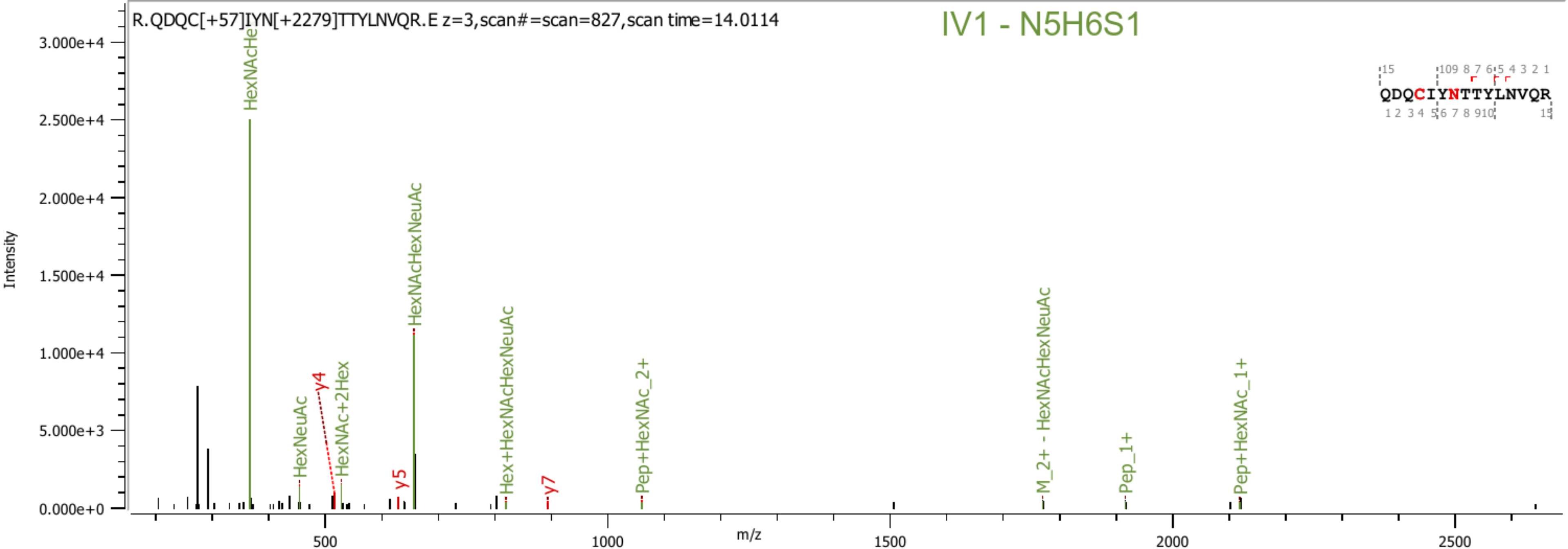
R.QDQC[+57]IYN[+2279]TTYLNVQR.E z=3,scan#=scan=827,scan time=14.0114
IV1 - N5H6S1
QDQCIYNTTYLNVQR
Intensity
m/z
HexNAcHe
HexNeuAc
y4
HexNAc+2Hex
y5
HexNAcHexNeuAc
Hex+HexNAcHexNeuAc
y7
Pep+HexNAc_2+
M_2+ - HexNAcHexNeuAc
Pep_1+
Pep+HexNAc_1+

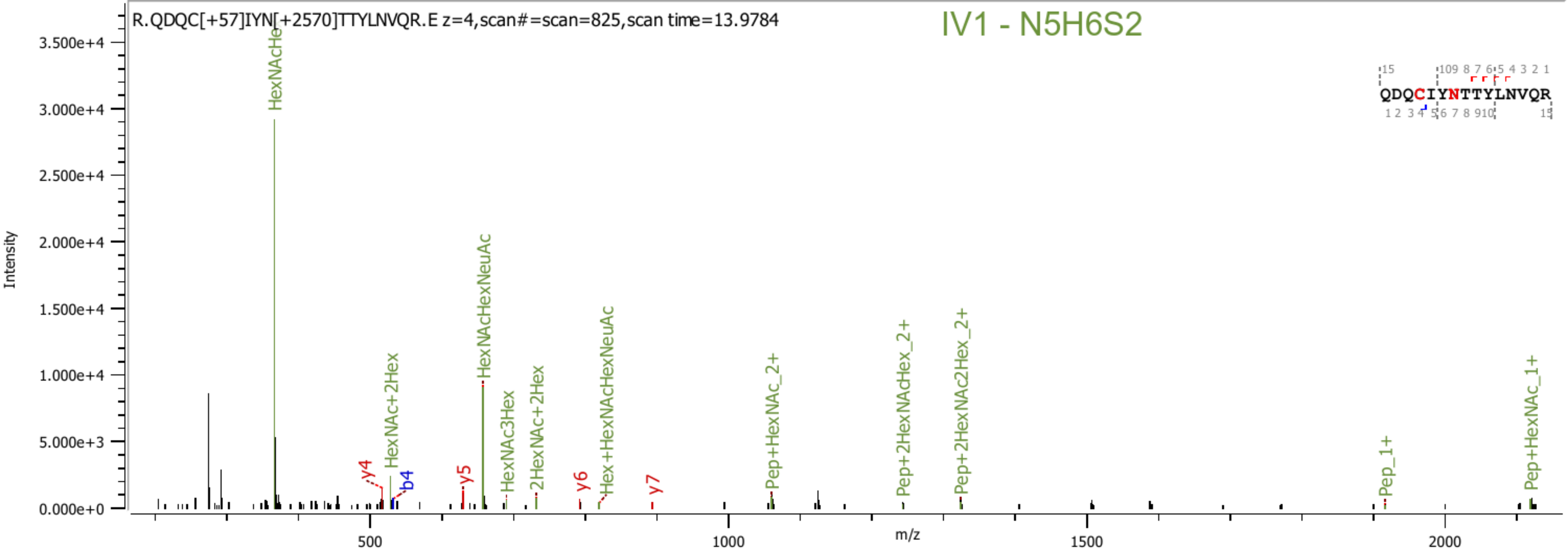
R.QDQC[+57]IYN[+2570]TTYLNVQR.E z=4,scan#=scan=825,scan time=13.9784
IV1 - N5H6S2
QDQCIYNTTYLNVQR
Intensity
m/z
HexNAcHe
HexNAc+2Hex
HexNAcHexNeuAc
HexNAc3Hex
2HexNAc+2Hex
Hex+HexNAcHexNeuAc
Pep+HexNAc_2+
Pep+2HexNAcHex_2+
Pep+2HexNAc2Hex_2+
Pep_1+
Pep+HexNAc_1+
y4
b4
y5
y6
y7
500
1000
1500
2000

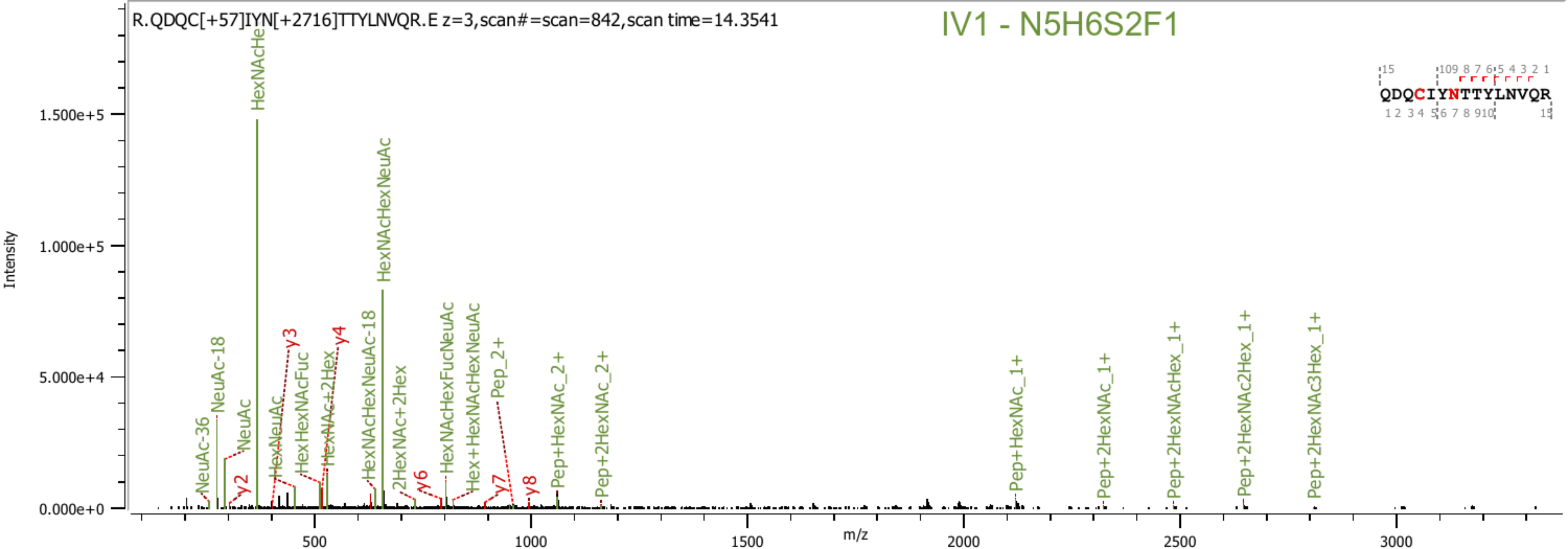
R.QDQC[+57]IYN[+2716]TTYLNVQR.E z=3,scan#=scan=842,scan time=14.3541
IV1 - N5H6S2F1
QDQCIYNTTYLNVQR
Intensity
1.500e+5
1.000e+5
5.000e+4
0.000e+0
m/z
500
1000
1500
2000
2500
3000
NeuAc-36
NeuAc-18
y2
NeuAc
HexNAcHe
HexNeuAc
y3
HexHexNAcFuc
HexNAc+2Hex
y4
HexNAcHexNeuAc-18
HexNAcHexNeuAc
2HexNAc+2Hex
y6
HexNAcHexFucNeuAc
Hex+HexNAcHexNeuAc
y7
Pep_2+
y8
Pep+HexNAc_2+
Pep+2HexNAc_2+
Pep+HexNAc_1+
Pep+2HexNAc_1+
Pep+2HexNAcHex_1+
Pep+2HexNAc2Hex_1+
Pep+2HexNAc3Hex_1+

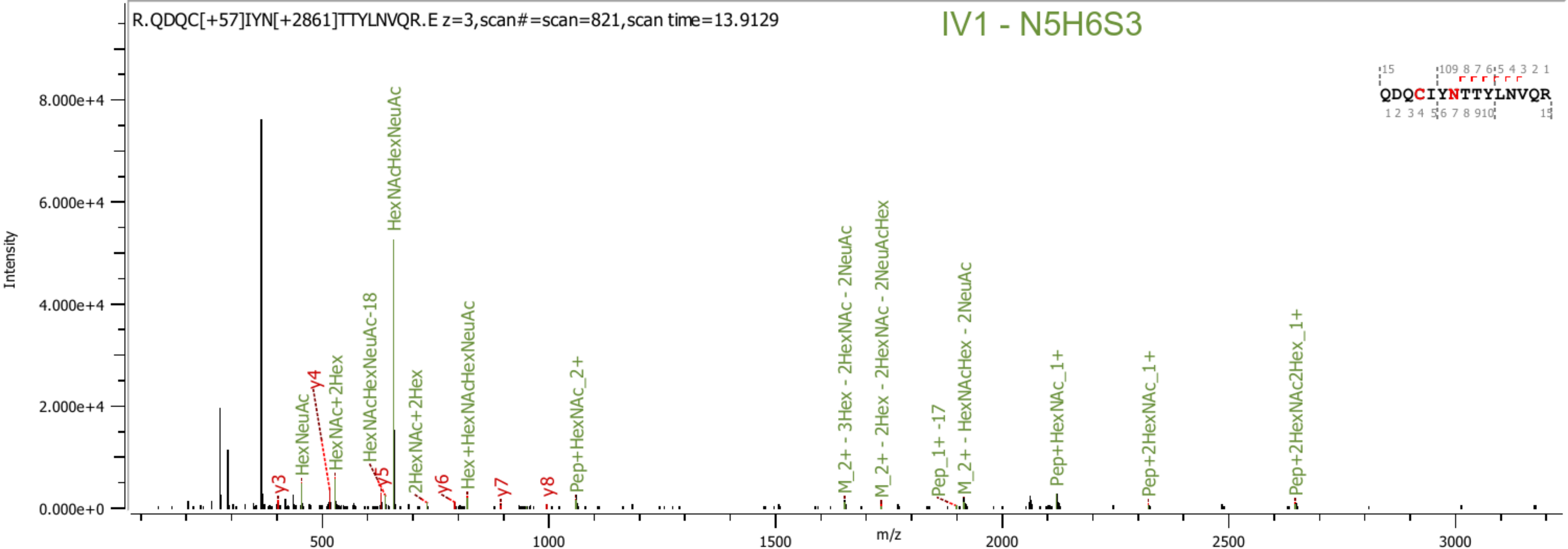
R.QDQC[+57]IYN[+2861]TTYLNVQR.E z=3,scan#=scan=821,scan time=13.9129
IV1 - N5H6S3
QDQCIYNTTYLNVQR
Intensity
8.000e+4
6.000e+4
4.000e+4
2.000e+4
0.000e+0
y3
HexNeuAc
y4
HexNAc+2Hex
HexNAcHexNeuAc-18
y5
HexNAcHexNeuAc
2HexNAc+2Hex
y6
Hex+HexNAcHexNeuAc
y7
y8
Pep+HexNAc_2+
M_2+ - 3Hex - 2HexNAc - 2NeuAc
M_2+ - 2Hex - 2HexNAc - 2NeuAcHex
Pep_1+ -17
M_2+ - HexNAcHex - 2NeuAc
Pep+HexNAc_1+
Pep+2HexNAc_1+
Pep+2HexNAc2Hex_1+
500
1000
1500
2000
2500
3000
m/z

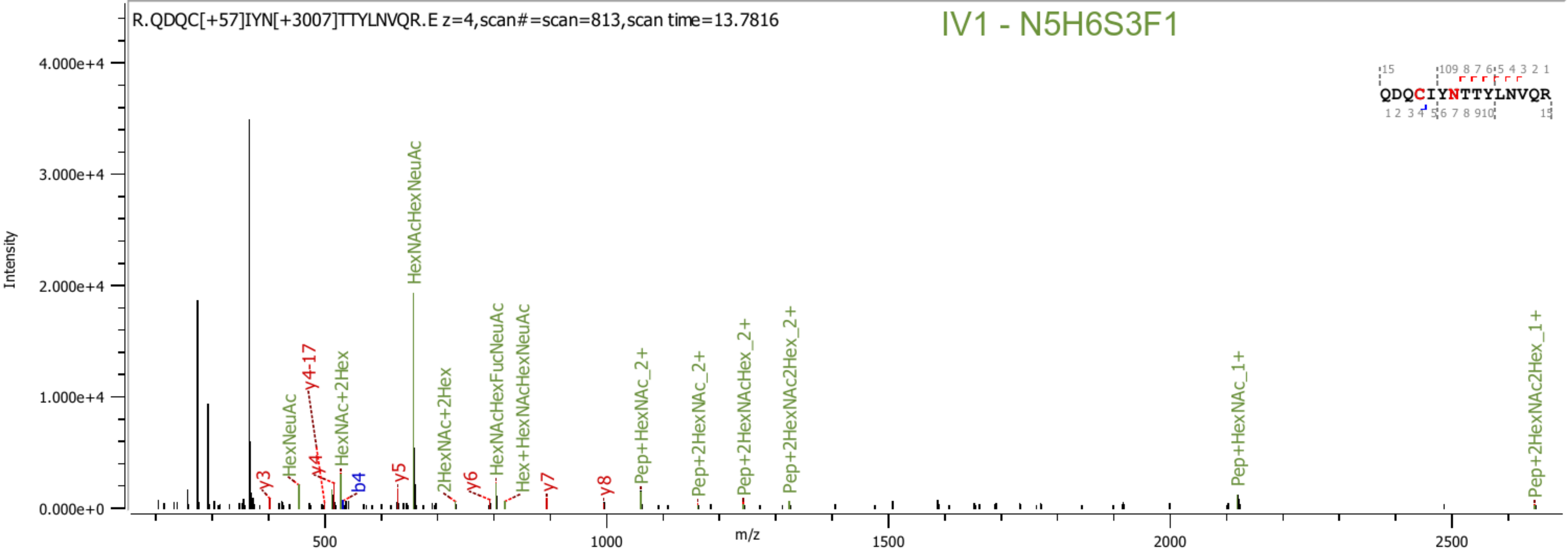

R.QDQC[+57]IYN[+3007]TTYLNVQR.E z=4,scan#=scan=813,scan time=13.7816
IV1 - N5H6S3F1
QDQCIYNTTYLNVQR
Intensity
m/z
4.000e+4
3.000e+4
2.000e+4
1.000e+4
0.000e+0
500
1000
1500
2000
2500
y3
HexNeuAc
y4-17
y4
HexNAc+2Hex
b4
y5
HexNAcHexNeuAc
2HexNAc+2Hex
y6
HexNAcHexFucNeuAc
Hex+HexNAcHexNeuAc
y7
y8
Pep+HexNAc_2+
Pep+2HexNAc_2+
Pep+2HexNAcHex_2+
Pep+2HexNAc2Hex_2+
Pep+HexNAc_1+
Pep+2HexNAc2Hex_1+

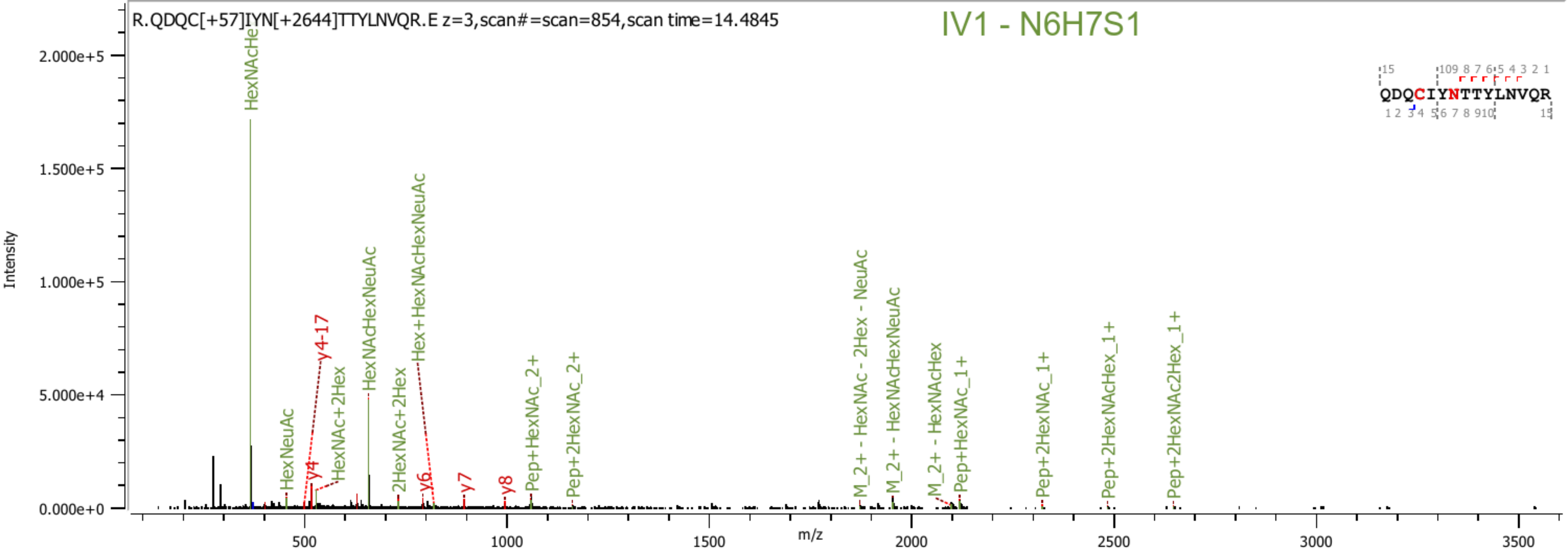

R.QDQC[+57]IYN[+2644]TTYLNVQR.E z=3,scan#=scan=854,scan time=14.4845
IV1 - N6H7S1
QDQCIYNTTYLNVQR
Intensity
m/z
HexNAcHex
HexNeuAc
y4
y4-17
HexNAc+2Hex
HexNAcHexNeuAc
2HexNAc+2Hex
y6
Hex+HexNAcHexNeuAc
y7
y8
Pep+HexNAc_2+
Pep+2HexNAc_2+
M_2+ - HexNAc - 2Hex - NeuAc
M_2+ - HexNAcHexNeuAc
M_2+ - HexNAcHex
Pep+HexNAc_1+
Pep+2HexNAc_1+
Pep+2HexNAcHex_1+
Pep+2HexNAc2Hex_1+

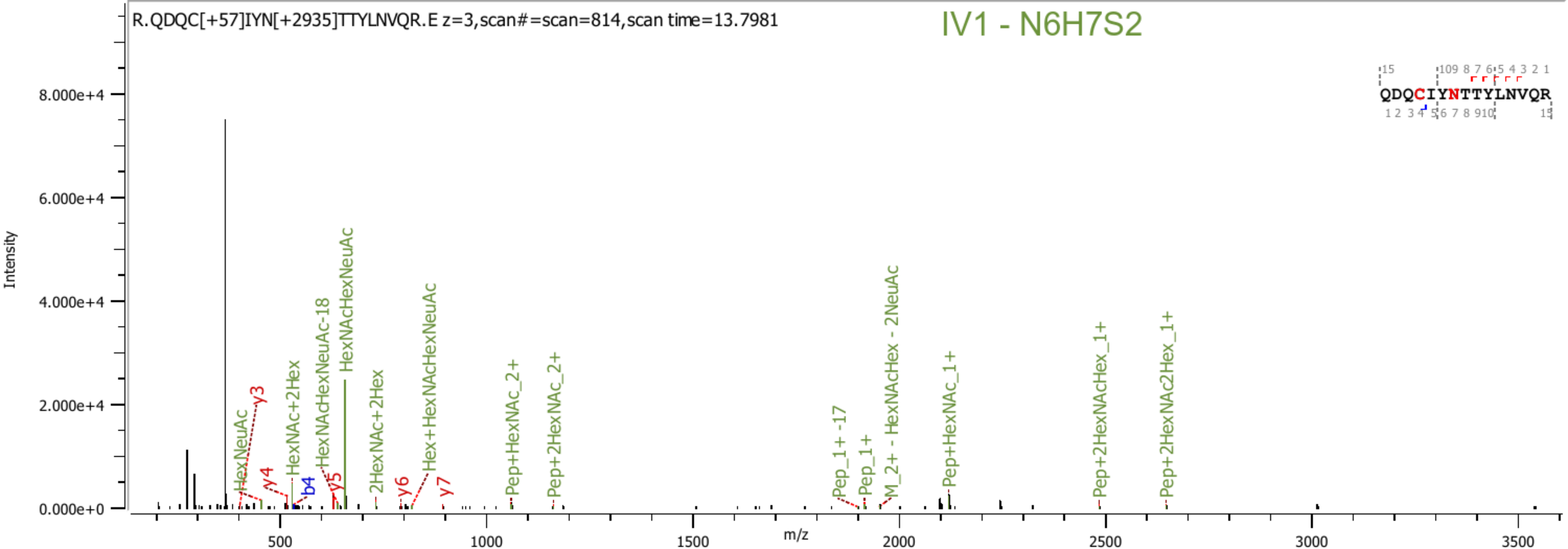

R.QDQC[+57]IYN[+2935]TTYLNVQR.E z=3,scan#=scan=814,scan time=13.7981
IV1 - N6H7S2
QDQCIYNTTYLNVQR
Intensity
m/z
8.000e+4
6.000e+4
4.000e+4
2.000e+4
0.000e+0
500
1000
1500
2000
2500
3000
3500
HexNeuAc
y3
y4
HexNAc+2Hex
b4
HexNAcHexNeuAc-18
y5
HexNAcHexNeuAc
2HexNAc+2Hex
y6
Hex+HexNAcHexNeuAc
y7
Pep+HexNAc_2+
Pep+2HexNAc_2+
Pep_1+ -17
Pep_1+
M_2+ - HexNAcHex - 2NeuAc
Pep+HexNAc_1+
Pep+2HexNAcHex_1+
Pep+2HexNAc2Hex_1+

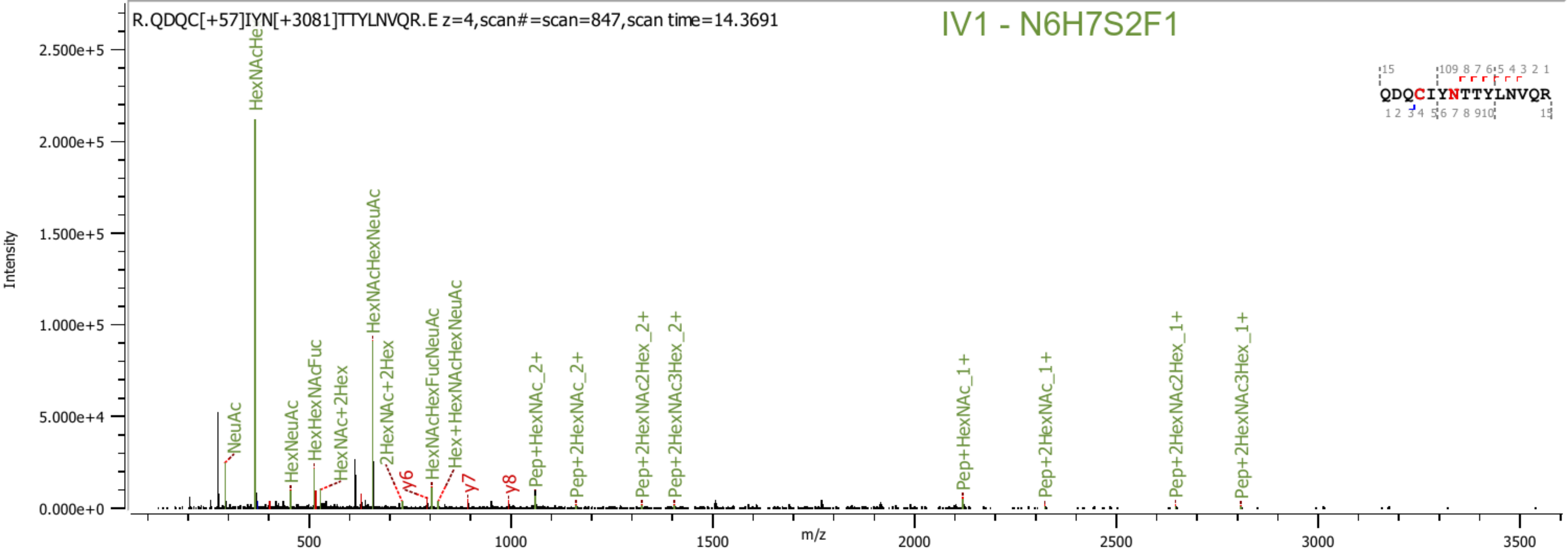
R.QDQC[+57]IYN[+3081]TTYLNVQR.E z=4,scan#=scan=847,scan time=14.3691
IV1 - N6H7S2F1
QDQCIYNTTYLNVQR
Intensity
2.500e+5
2.000e+5
1.500e+5
1.000e+5
5.000e+4
0.000e+0
m/z
500
1000
1500
2000
2500
3000
3500
NeuAc
HexNAcHe
HexNeuAc
HexHexNAcFuc
HexNAc+2Hex
HexNAcHexNeuAc
2HexNAc+2Hex
y6
HexNAcHexFucNeuAc
Hex+HexNAcHexNeuAc
y7
y8
Pep+HexNAc_2+
Pep+2HexNAc_2+
Pep+2HexNAc2Hex_2+
Pep+2HexNAc3Hex_2+
Pep+HexNAc_1+
Pep+2HexNAc_1+
Pep+2HexNAc2Hex_1+
Pep+2HexNAc3Hex_1+

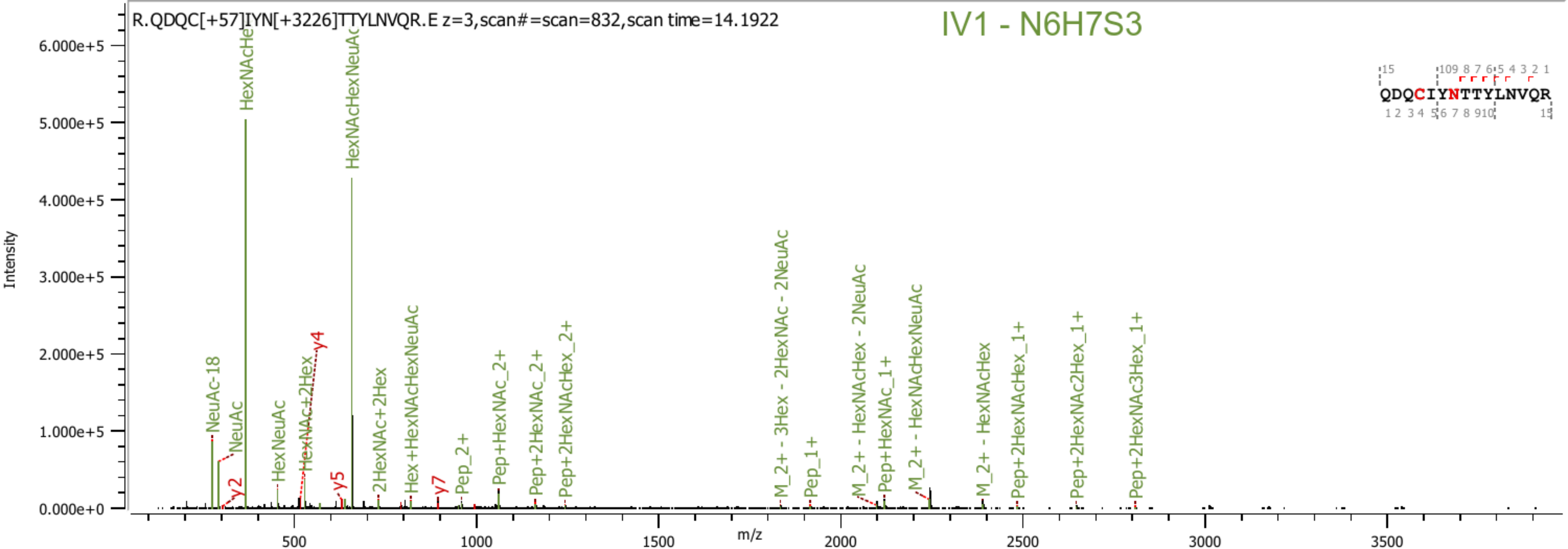
R.QDQC[+57]IYN[+3226]TTYLNVQR.E z=3,scan#=scan=832,scan time=14.1922
IV1 - N6H7S3
QDQCIYNTTYLNVQR
Intensity
m/z
HexNAcHex
HexNAcHexNeuAc
NeuAc-18
NeuAc
y2
HexNeuAc
HexNAc+2Hex
y4
y5
2HexNAc+2Hex
Hex+HexNAcHexNeuAc
y7
Pep_2+
Pep+HexNAc_2+
Pep+2HexNAc_2+
Pep+2HexNAcHex_2+
M_2+ - 3Hex - 2HexNAc - 2NeuAc
Pep_1+
M_2+ - HexNAcHex - 2NeuAc
Pep+HexNAc_1+
M_2+ - HexNAcHexNeuAc
M_2+ - HexNAcHex
Pep+2HexNAcHex_1+
Pep+2HexNAc2Hex_1+
Pep+2HexNAc3Hex_1+

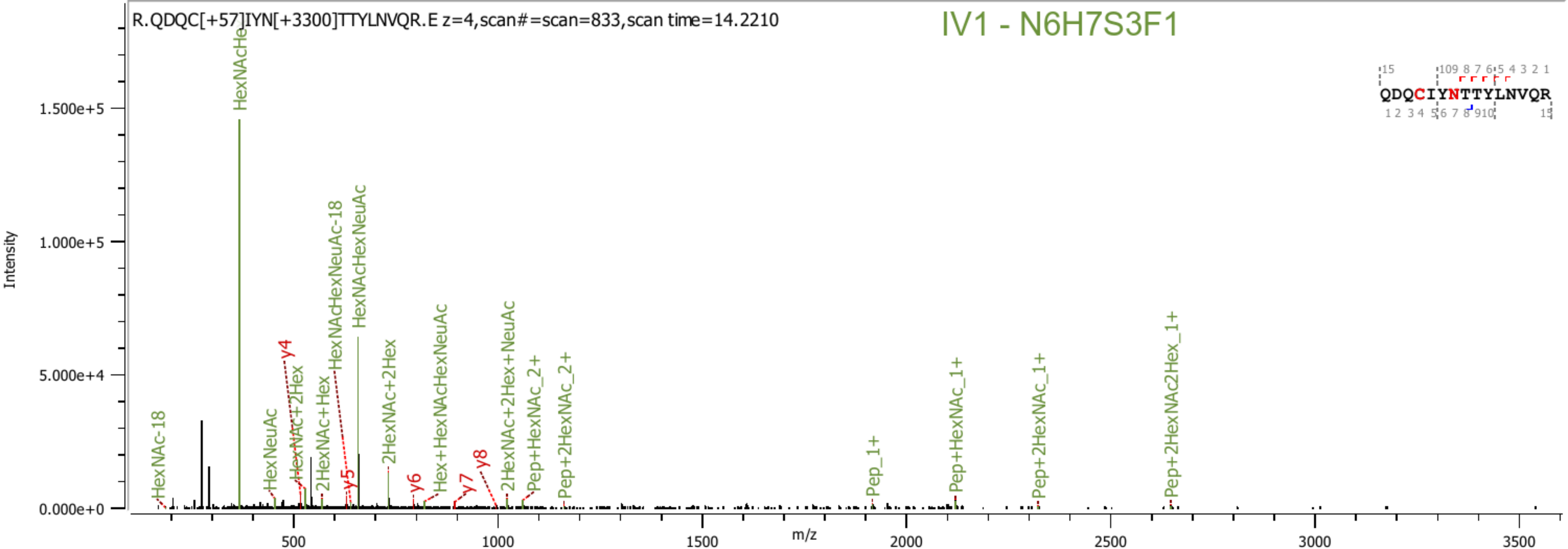
R.QDQC[+57]IYN[+3300]TTYLNVQR.E z=4,scan#=scan=833,scan time=14.2210
IV1 - N6H7S3F1
QDQCIYNTTYLNVQR
Intensity
m/z
HexNAc-18
HexNAcHex
HexNeuAc
HexNAc+2Hex
2HexNAc+Hex
HexNAcHexNeuAc-18
HexNAcHexNeuAc
2HexNAc+2Hex
Hex+HexNAcHexNeuAc
2HexNAc+2Hex+NeuAc
Pep+HexNAc_2+
Pep+2HexNAc_2+
Pep_1+
Pep+HexNAc_1+
Pep+2HexNAc_1+
Pep+2HexNAc2Hex_1+
y4
y5
y6
y7
y8

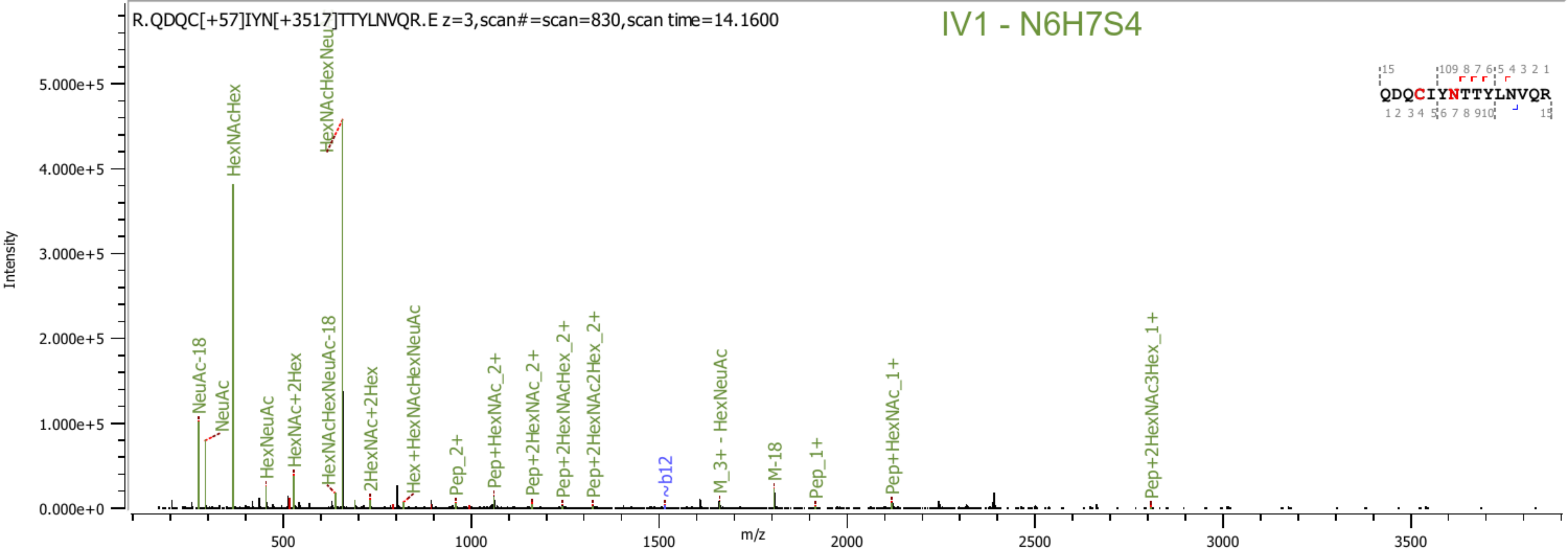
R.QDQC[+57]IYN[+3517]TTYLNVQR.E z=3,scan#=scan=830,scan time=14.1600
IV1 - N6H7S4
QDQCIYNTTYLNVQR
Intensity
m/z
NeuAc-18
NeuAc
HexNAcHex
HexNeuAc
HexNAc+2Hex
HexNAcHexNeuAc-18
HexNAcHexNeu
2HexNAc+2Hex
Hex+HexNAcHexNeuAc
Pep_2+
Pep+HexNAc_2+
Pep+2HexNAc_2+
Pep+2HexNAcHex_2+
Pep+2HexNAc2Hex_2+
~b12
M_3+ - HexNeuAc
M-18
Pep_1+
Pep+HexNAc_1+
Pep+2HexNAc3Hex_1+

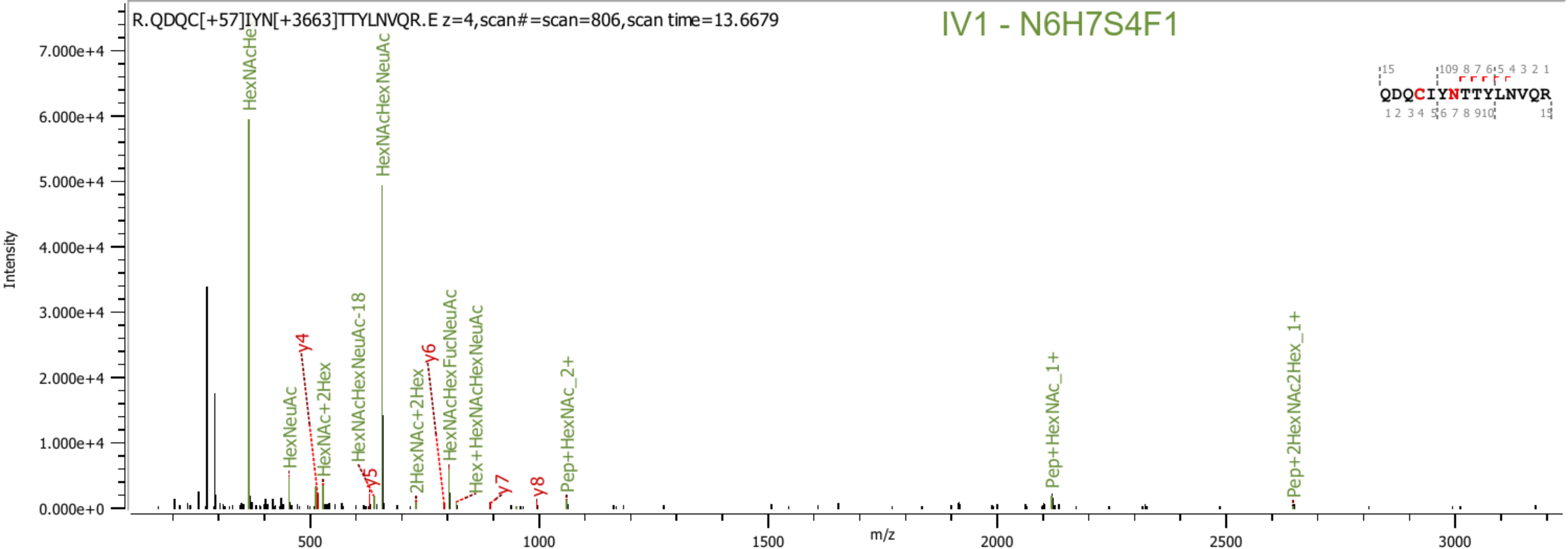

R.QDQC[+57]IYN[+3663]TTYLNVQR.E z=4,scan#=scan=806,scan time=13.6679
IV1 - N6H7S4F1
QDQCIYNTTYLNVQR
Intensity
m/z
HexNAcHex
HexNAcHexNeuAc
HexNeuAc
y4
HexNAc+2Hex
HexNAcHexNeuAc-18
y5
2HexNAc+2Hex
y6
HexNAcHexFucNeuAc
Hex+HexNAcHexNeuAc
y7
y8
Pep+HexNAc_2+
Pep+HexNAc_1+
Pep+2HexNAc2Hex_1+

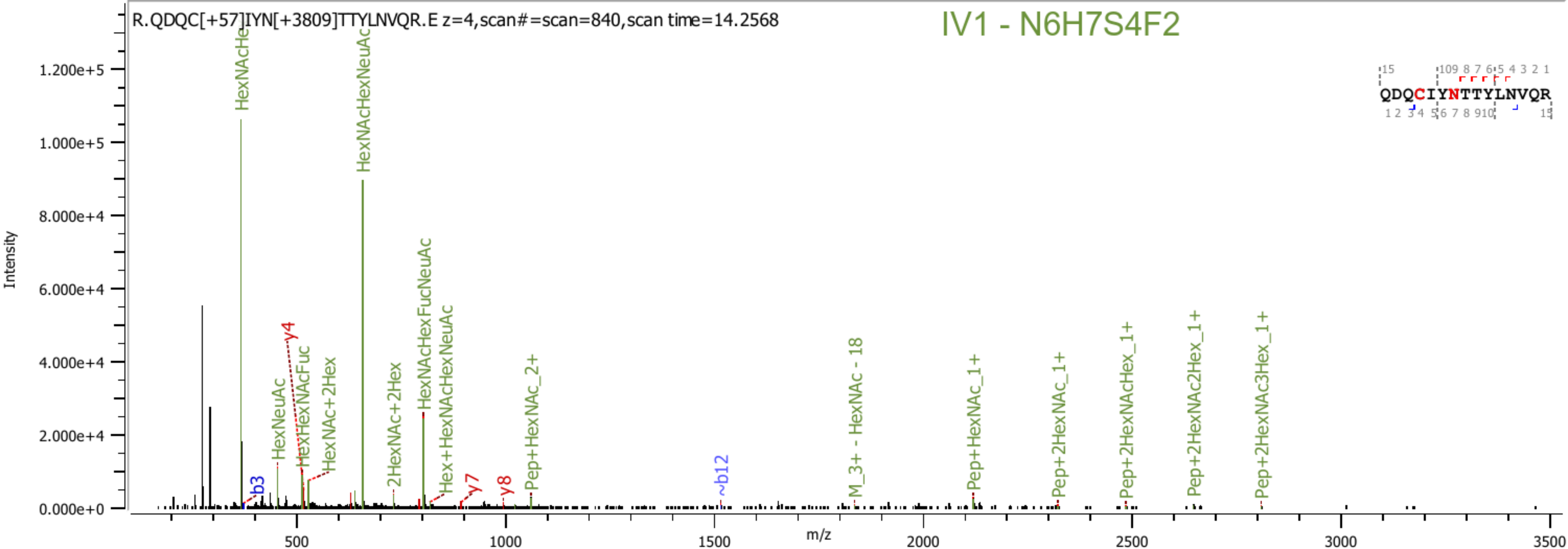

R.QDQC[+57]IYN[+3809]TTYLNVQR.E z=4,scan#=scan=840,scan time=14.2568
IV1 - N6H7S4F2
QDQCIYNTTYLNVQR
Intensity
m/z
HexNAcHex
HexNAcHexNeuAc
HexNeuAc
y4
HexHexNAcFuc
HexNAc+2Hex
2HexNAc+2Hex
HexNAcHexFucNeuAc
Hex+HexNAcHexNeuAc
b3
y7
y8
Pep+HexNAc_2+
~b12
M_3+ - HexNAc - 18
Pep+HexNAc_1+
Pep+2HexNAc_1+
Pep+2HexNAcHex_1+
Pep+2HexNAc2Hex_1+
Pep+2HexNAc3Hex_1+

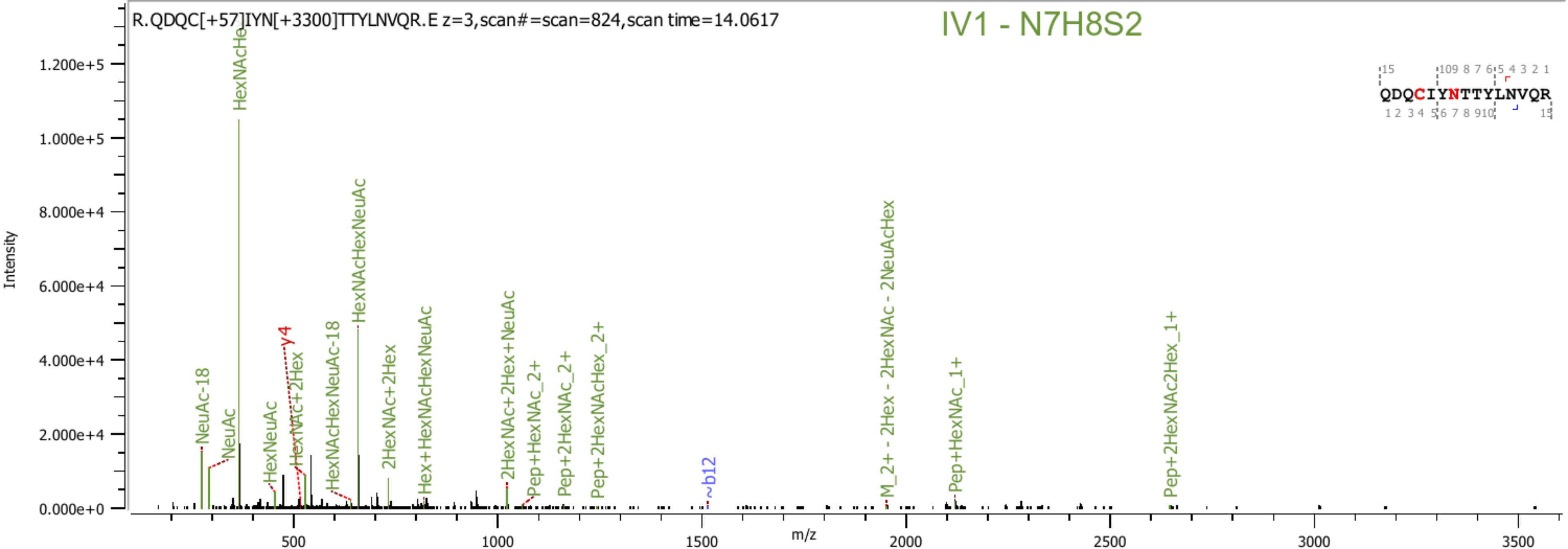

R.QDQC[+57]IYN[+3300]TTYLNVQR.E z=3,scan#=scan=824,scan time=14.0617
IV1 - N7H8S2
QDQCIYNTTYLNVQR
Intensity
m/z
NeuAc-18
NeuAc
HexNAcHex
HexNeuAc
HexNAc+2Hex
y4
HexNAcHexNeuAc-18
HexNAcHexNeuAc
2HexNAc+2Hex
Hex+HexNAcHexNeuAc
2HexNAc+2Hex+NeuAc
Pep+HexNAc_2+
Pep+2HexNAc_2+
Pep+2HexNAcHex_2+
~b12
M_2+ - 2Hex - 2HexNAc - 2NeuAcHex
Pep+HexNAc_1+
Pep+2HexNAc2Hex_1+

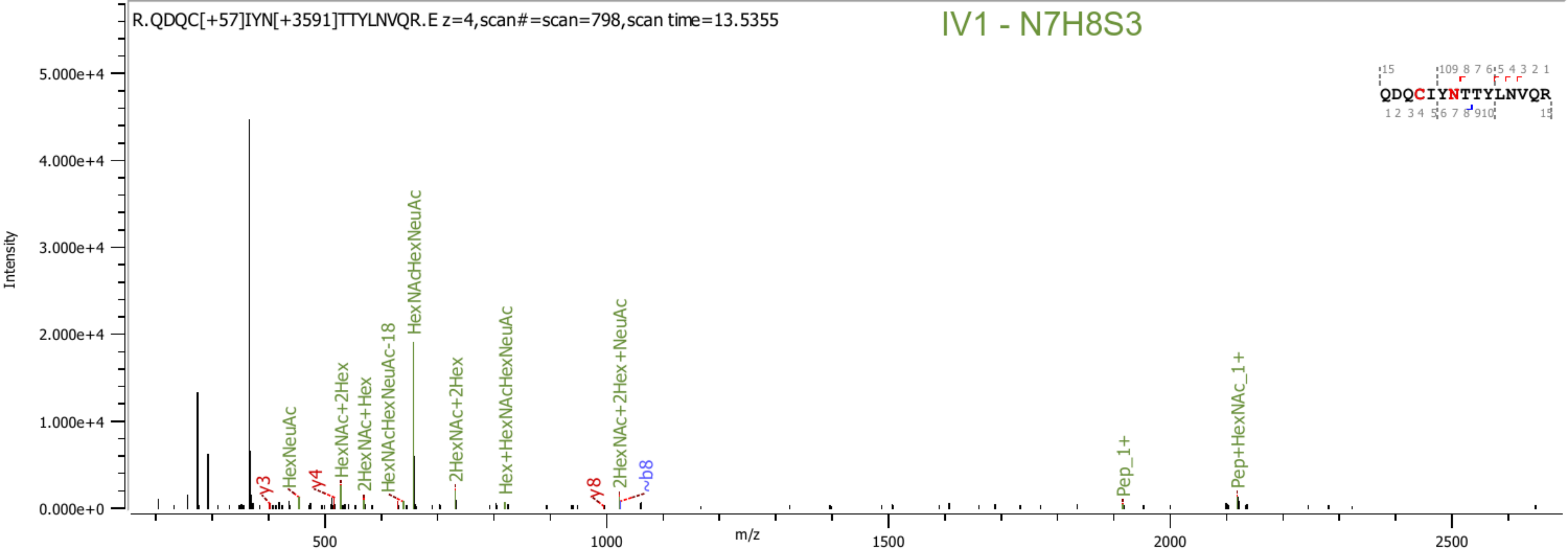
R.QDQC[+57]IYN[+3591]TTYLNVQR.E z=4,scan#=scan=798,scan time=13.5355
IV1 - N7H8S3
QDQCIYNTTYLNVQR
Intensity
5.000e+4
4.000e+4
3.000e+4
2.000e+4
1.000e+4
0.000e+0
y3
HexNeuAc
y4
HexNAc+2Hex
2HexNAc+Hex
HexNAcHexNeuAc-18
HexNAdHexNeuAc
2HexNAc+2Hex
Hex+HexNAcHexNeuAc
y8
2HexNAc+2Hex+NeuAc
~b8
Pep_1+
Pep+HexNAc_1+
500
1000
1500
2000
2500
m/z

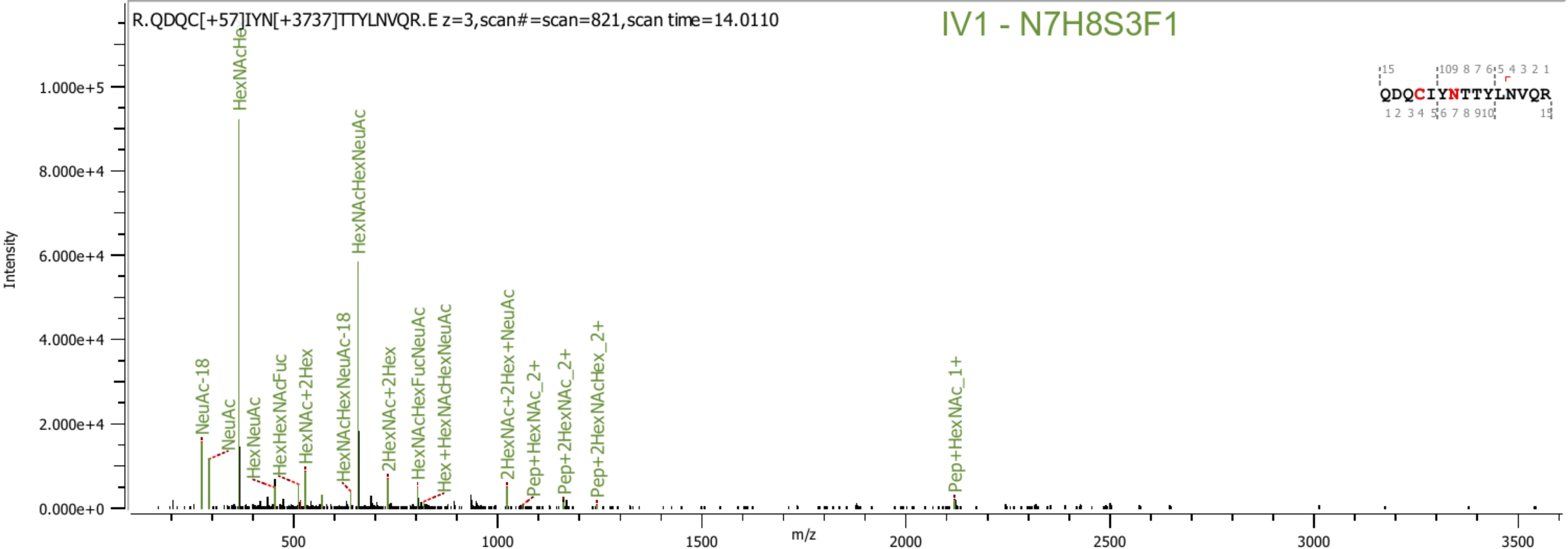
R.QDQC[+57]IYN[+3737]TTYLNVQR.E z=3,scan#=scan=821,scan time=14.0110
IV1 - N7H8S3F1
QDQCIYNTTYLNVQR
Intensity
m/z
NeuAc-18
NeuAc
HexNAcHex
HexNeuAc
HexHexNAcFuc
HexNAc+2Hex
HexNAcHexNeuAc-18
HexNAcHexNeuAc
2HexNAc+2Hex
HexNAcHexFucNeuAc
Hex+HexNAcHexNeuAc
2HexNAc+2Hex+NeuAc
Pep+HexNAc_2+
Pep+2HexNAc_2+
Pep+2HexNAcHex_2+
Pep+HexNAc_1+

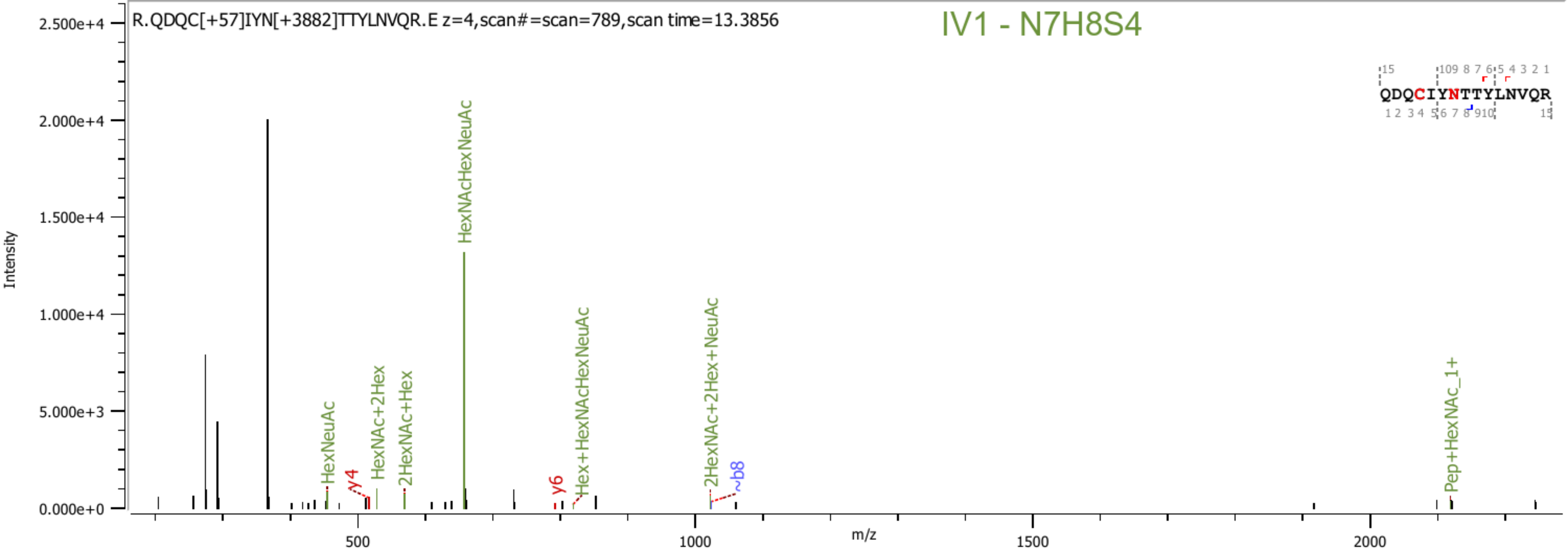
R.QDQC[+57]IYN[+3882]TTYLNVQR.E z=4,scan#=scan=789,scan time=13.3856
IV1 - N7H8S4
QDQCIYNTTYLNVQR
Intensity
m/z
HexNeuAc
y4
HexNAc+2Hex
2HexNAc+Hex
HexNAcHexNeuAc
y6
Hex+HexNAcHexNeuAc
2HexNAc+2Hex+NeuAc
~b8
Pep+HexNAc_1+
2.500e+4
2.000e+4
1.500e+4
1.000e+4
5.000e+3
0.000e+0
500
1000
1500
2000

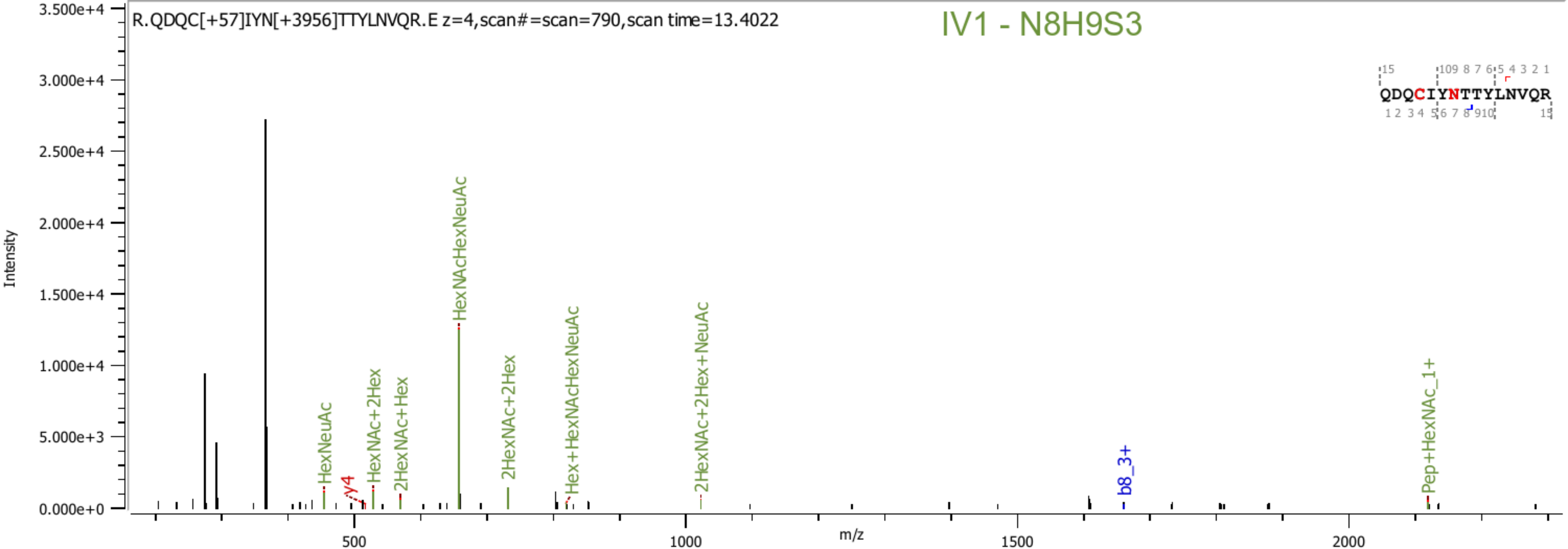

R.QDQC[+57]IYN[+3956]TTYLNVQR.E z=4,scan#=scan=790,scan time=13.4022
IV1 - N8H9S3
QDQCIYNTTYLNVQR
Intensity
m/z
HexNeuAc
y4
HexNAc+2Hex
2HexNAc+Hex
HexNAcHexNeuAc
2HexNAc+2Hex
Hex+HexNAcHexNeuAc
2HexNAc+2Hex+NeuAc
b8_3+
Pep+HexNAc_1+

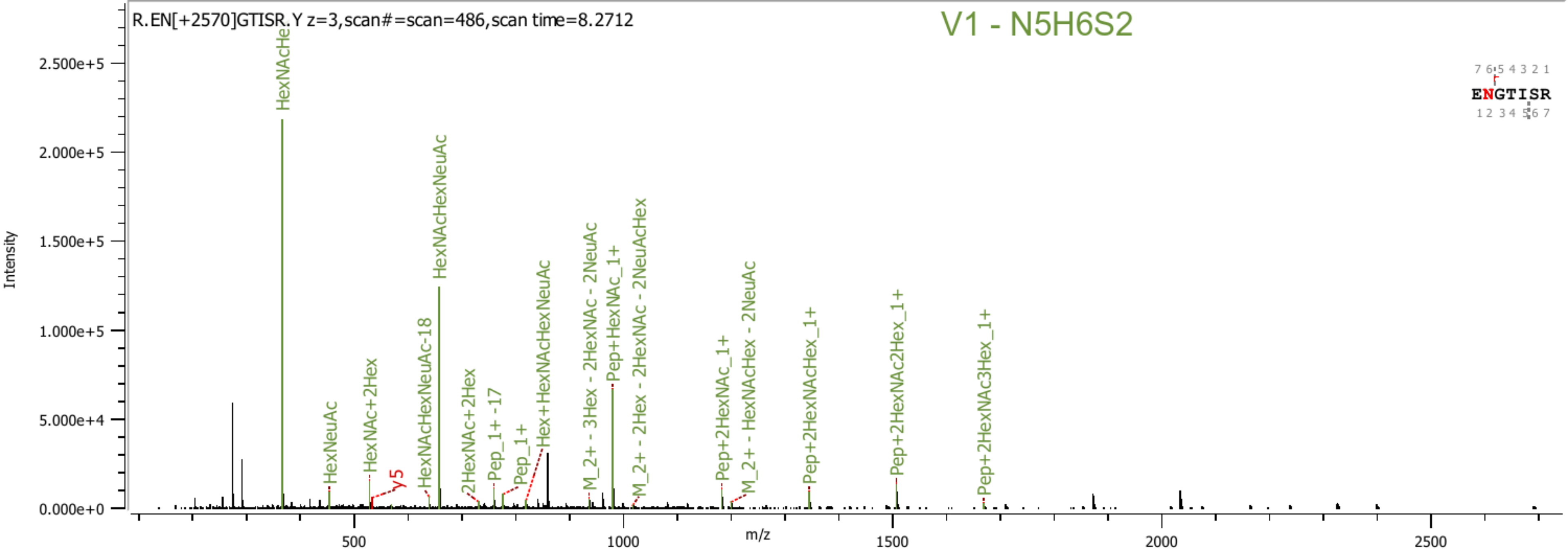
R.EN[+2570]GTISR.Y z=3,scan#=scan=486,scan time=8.2712
V1 - N5H6S2
ENGTISR
Intensity
m/z
HexNAcHex
HexNeuAc
HexNAc+2Hex
y5
HexNAcHexNeuAc-18
HexNAcHexNeuAc
2HexNAc+2Hex
Pep_1+ -17
Pep_1+
Hex+HexNAcHexNeuAc
M_2+ - 3Hex - 2HexNAc - 2NeuAc
Pep+HexNAc_1+
M_2+ - 2Hex - 2HexNAc - 2NeuAcHex
Pep+2HexNAc_1+
M_2+ - HexNAcHex - 2NeuAc
Pep+2HexNAcHex_1+
Pep+2HexNAc2Hex_1+
Pep+2HexNAc3Hex_1+

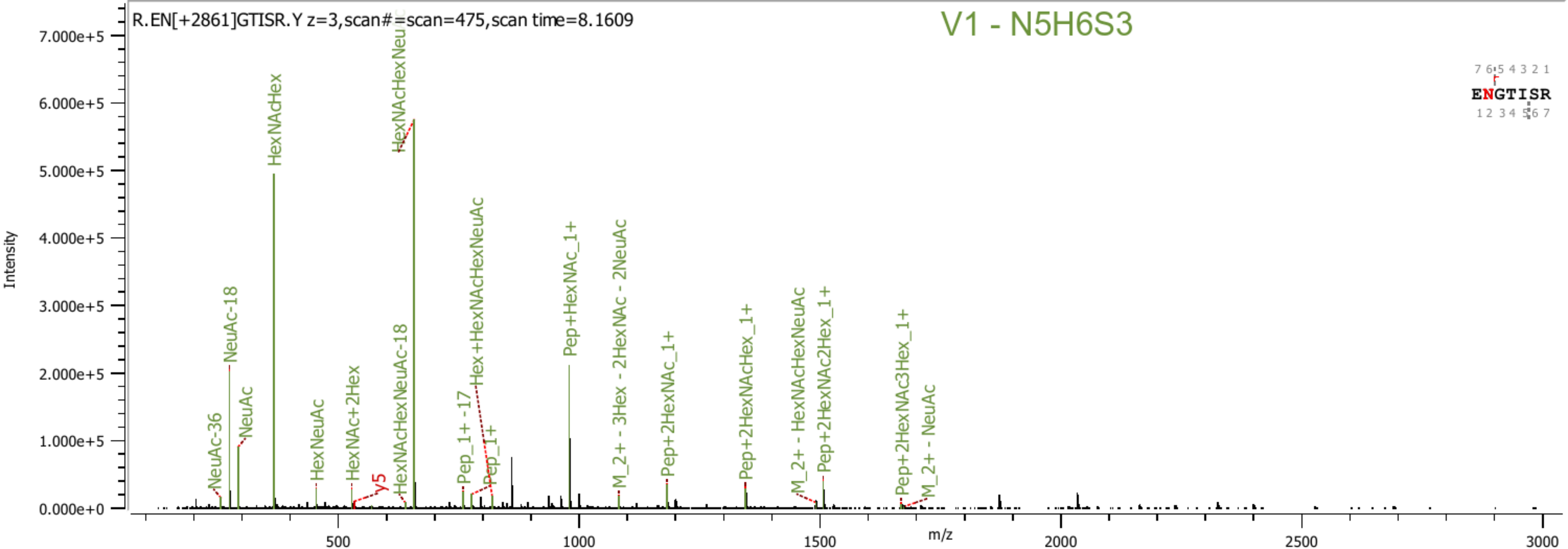

R.EN[+2861]GTISR.Y z=3,scan#=scan=475,scan time=8.1609
V1 - N5H6S3
ENGTISR
Intensity
m/z
NeuAc-36
NeuAc-18
NeuAc
HexNAcHex
HexNeuAc
HexNAc+2Hex
y5
HexNAcHexNeuAc-18
Pep_1+ -17
Pep_1+
Hex+HexNAcHexNeuAc
Pep+HexNAc_1+
M_2+ - 3Hex - 2HexNAc - 2NeuAc
Pep+2HexNAc_1+
Pep+2HexNAcHex_1+
M_2+ - HexNAcHexNeuAc
Pep+2HexNAc2Hex_1+
Pep+2HexNAc3Hex_1+
M_2+ - NeuAc

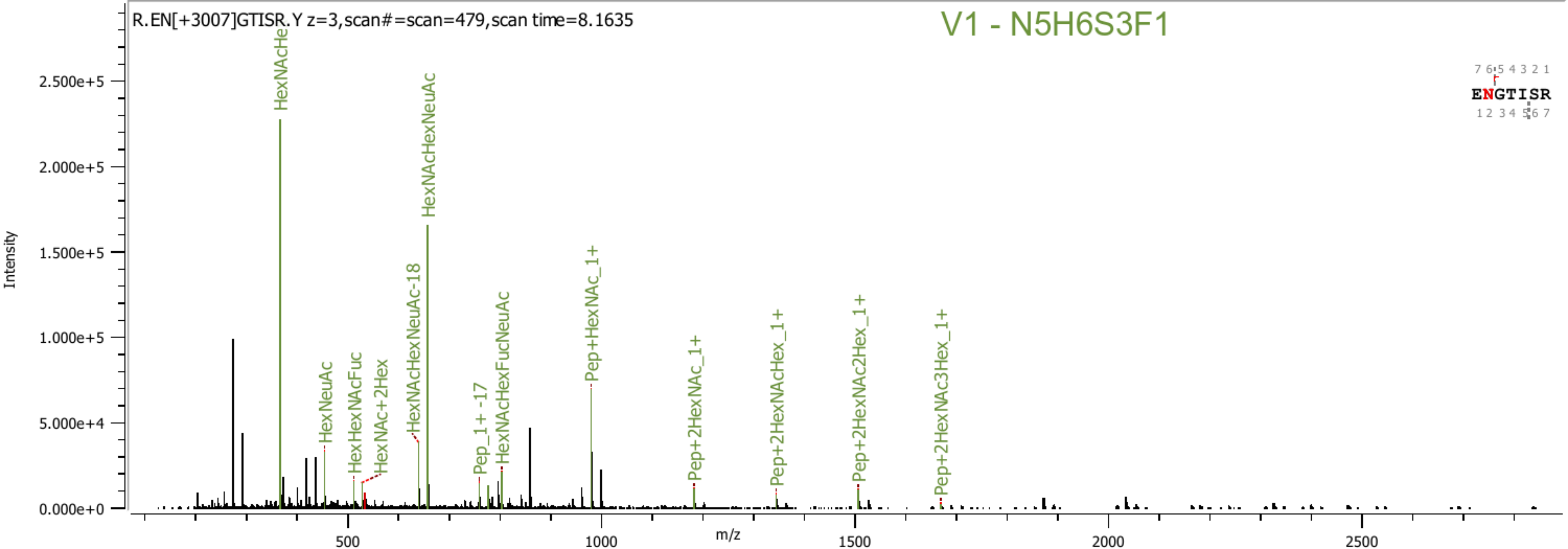

R.EN[+3007]GTISR.Y z=3,scan#=scan=479,scan time=8.1635
V1 - N5H6S3F1
ENGTISR
Intensity
m/z
HexNAcHex
HexNAcHexNeuAc
HexNeuAc
HexHexNAcFuc
HexNAc+2Hex
HexNAcHexNeuAc-18
Pep_1+ -17
HexNAcHexFucNeuAc
Pep+HexNAc_1+
Pep+2HexNAc_1+
Pep+2HexNAcHex_1+
Pep+2HexNAc2Hex_1+
Pep+2HexNAc3Hex_1+

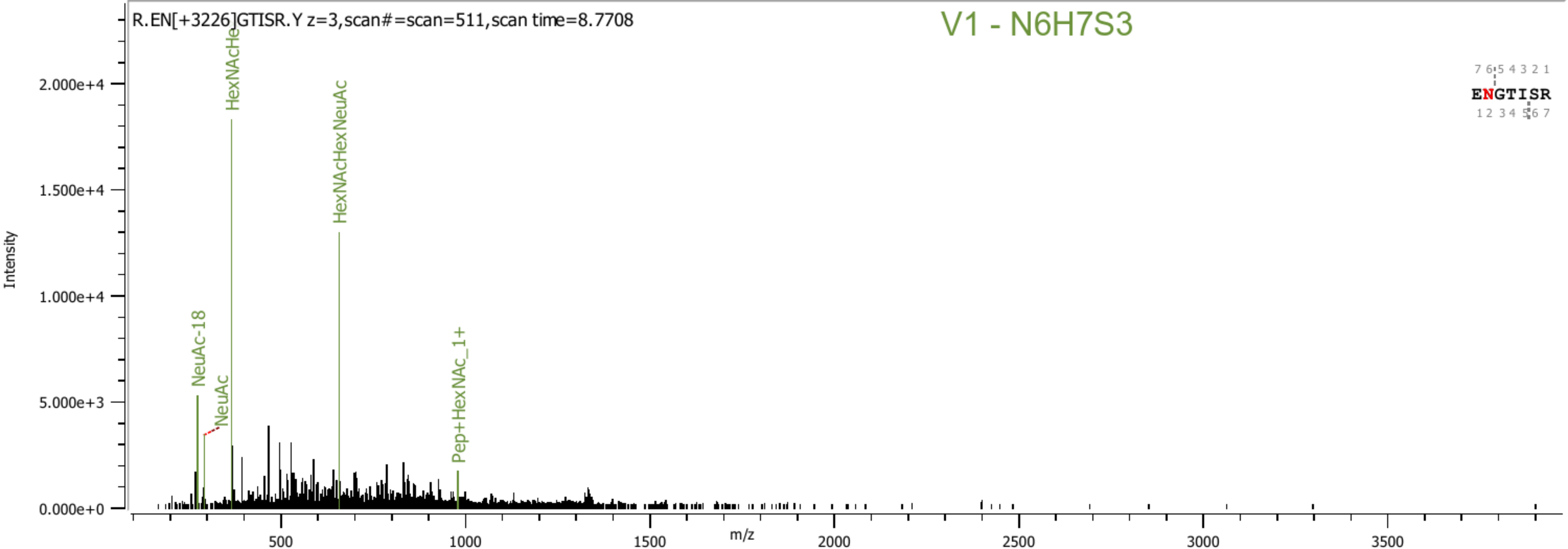

R.EN[+3226]GTISR.Y z=3,scan#=scan=511,scan time=8.7708
V1 - N6H7S3
7 6 5 4 3 2 1
ENGTISR
1 2 3 4 5 6 7
Intensity
2.000e+4
1.500e+4
1.000e+4
5.000e+3
0.000e+0
HexNAcHe
HexNAcHexNeuAc
NeuAc-18
NeuAc
Pep+HexNAc_1+
500
1000
1500
2000
2500
3000
3500
m/z

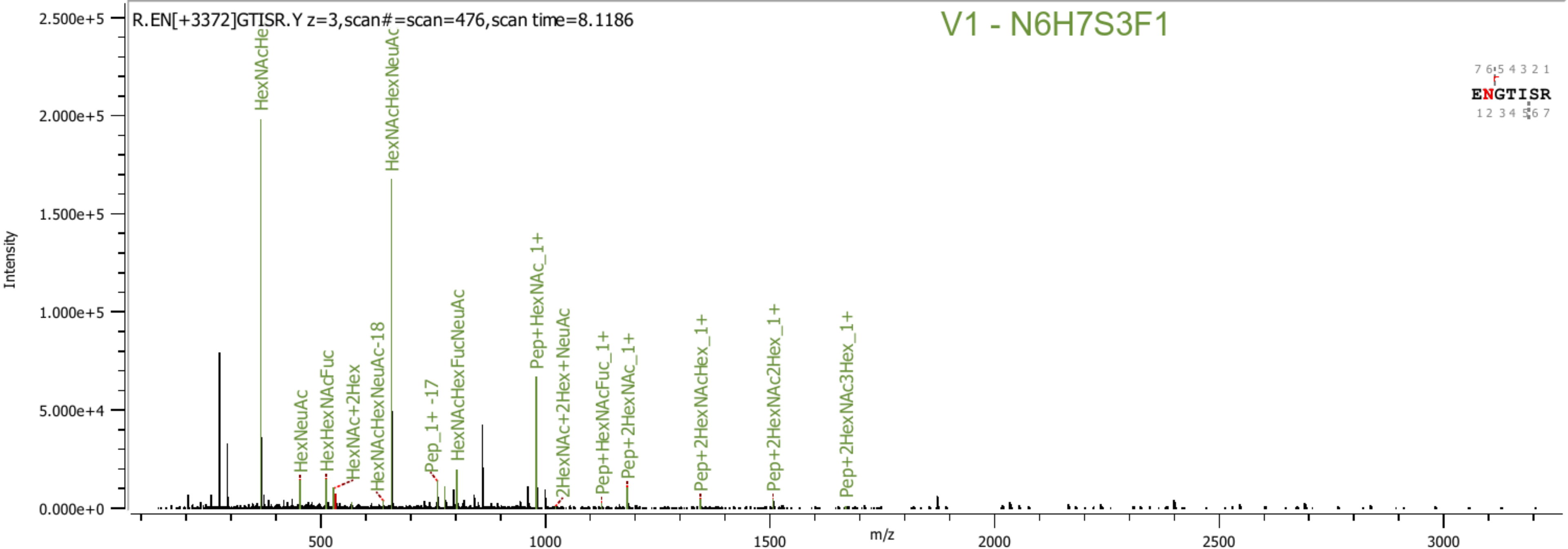
R.EN[+3372]GTISR.Y z=3,scan#=scan=476,scan time=8.1186
V1 - N6H7S3F1
ENGTISR
Intensity
m/z
HexNAcHex
HexNAcHexNeuAc
HexNeuAc
HexHexNAcFuc
HexNAc+2Hex
HexNAcHexNeuAc-18
Pep_1+ -17
HexNAcHexFucNeuAc
Pep+HexNAc_1+
2HexNAc+2Hex+NeuAc
Pep+HexNAcFuc_1+
Pep+2HexNAc_1+
Pep+2HexNAcHex_1+
Pep+2HexNAc2Hex_1+
Pep+2HexNAc3Hex_1+

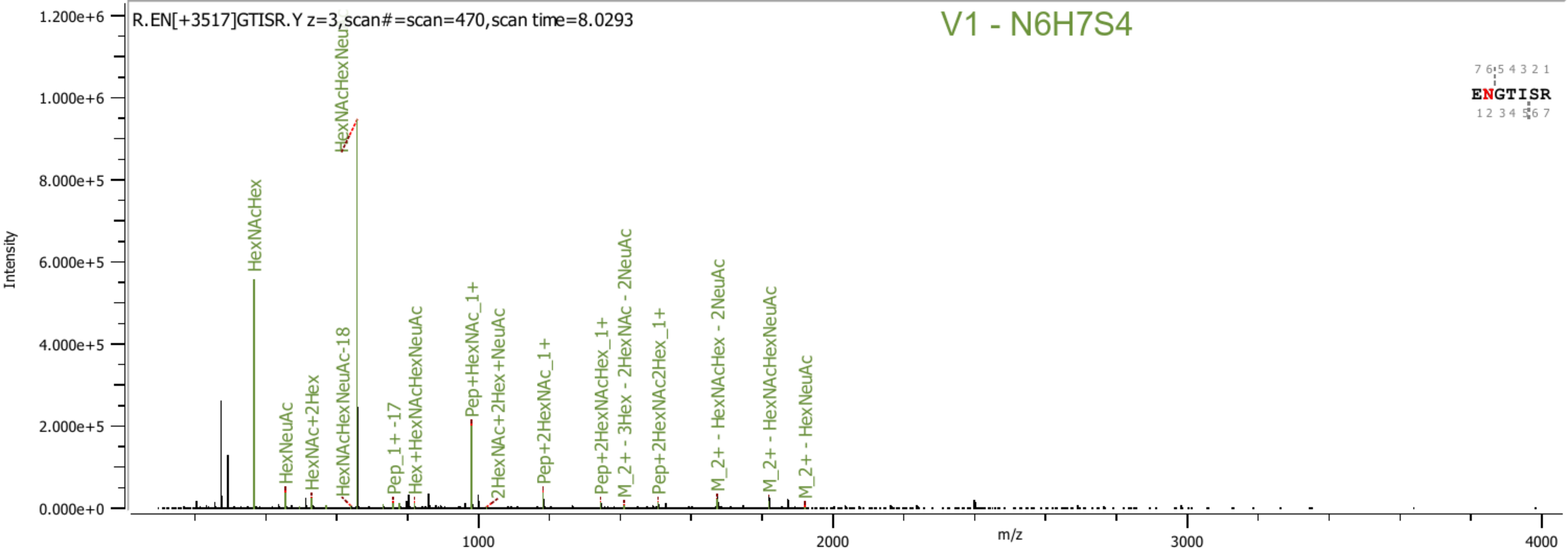
R.EN[+3517]GTISR.Y z=3,scan#=scan=470,scan time=8.0293
V1 - N6H7S4
7 6 5 4 3 2 1
ENGTISR
1 2 3 4 5 6 7
Intensity
1.200e+6
1.000e+6
8.000e+5
6.000e+5
4.000e+5
2.000e+5
0.000e+0
HexNAcHexNeu
HexNAcHex
HexNeuAc
HexNAc+2Hex
HexNAcHexNeuAc-18
Pep_1+ -17
Hex +HexNAcHexNeuAc
Pep+HexNAc_1+
2HexNAc+2Hex+NeuAc
Pep+2HexNAc_1+
Pep+2HexNAcHex_1+
M_2+ - 3Hex - 2HexNAc - 2NeuAc
Pep+2HexNAc2Hex_1+
M_2+ - HexNAcHex - 2NeuAc
M_2+ - HexNAcHexNeuAc
M_2+ - HexNeuAc
m/z
1000
2000
3000
4000

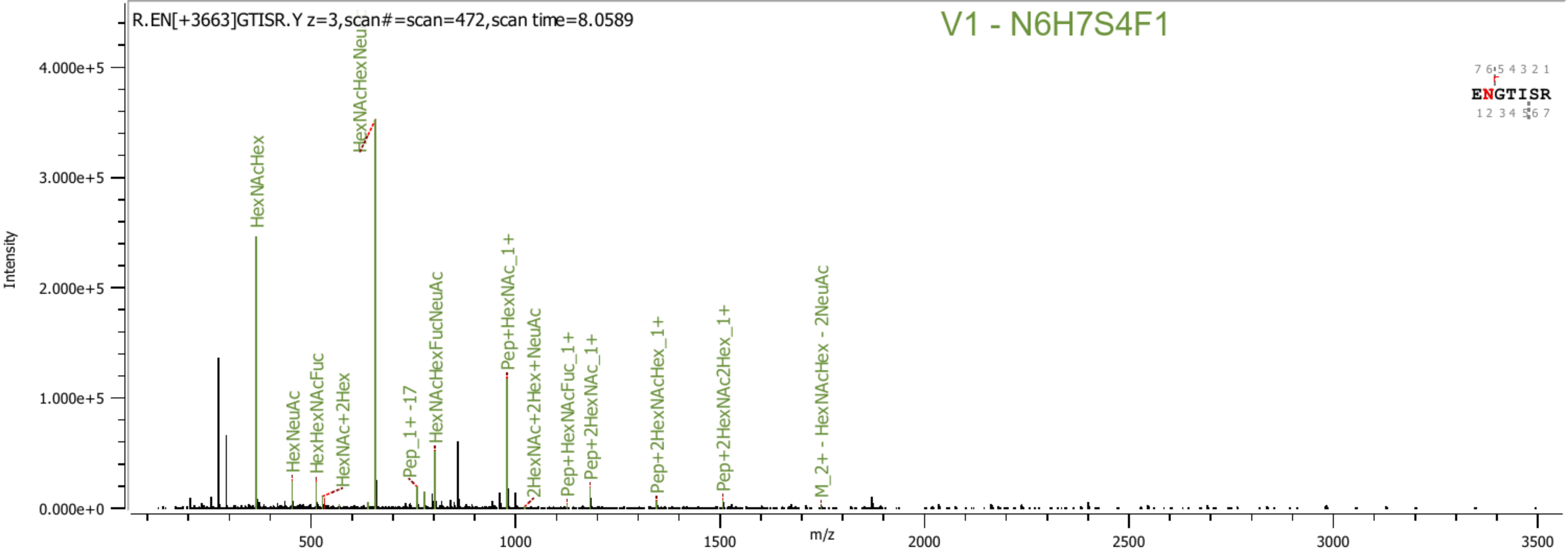

R.EN[+3663]GTISR.Y z=3,scan#=scan=472,scan time=8.0589
V1 - N6H7S4F1
ENGTISR
Intensity
m/z
HexNAc Hex
HexNAcHexNeu
HexNeuAc
HexHexNAcFuc
HexNAc+2Hex
Pep_1+ -17
HexNAcHexFucNeuAc
Pep+HexNAc_1+
2HexNAc+2Hex+NeuAc
Pep+HexNAcFuc_1+
Pep+2HexNAc_1+
Pep+2HexNAcHex_1+
Pep+2HexNAc2Hex_1+
M_2+ - HexNAcHex - 2NeuAc

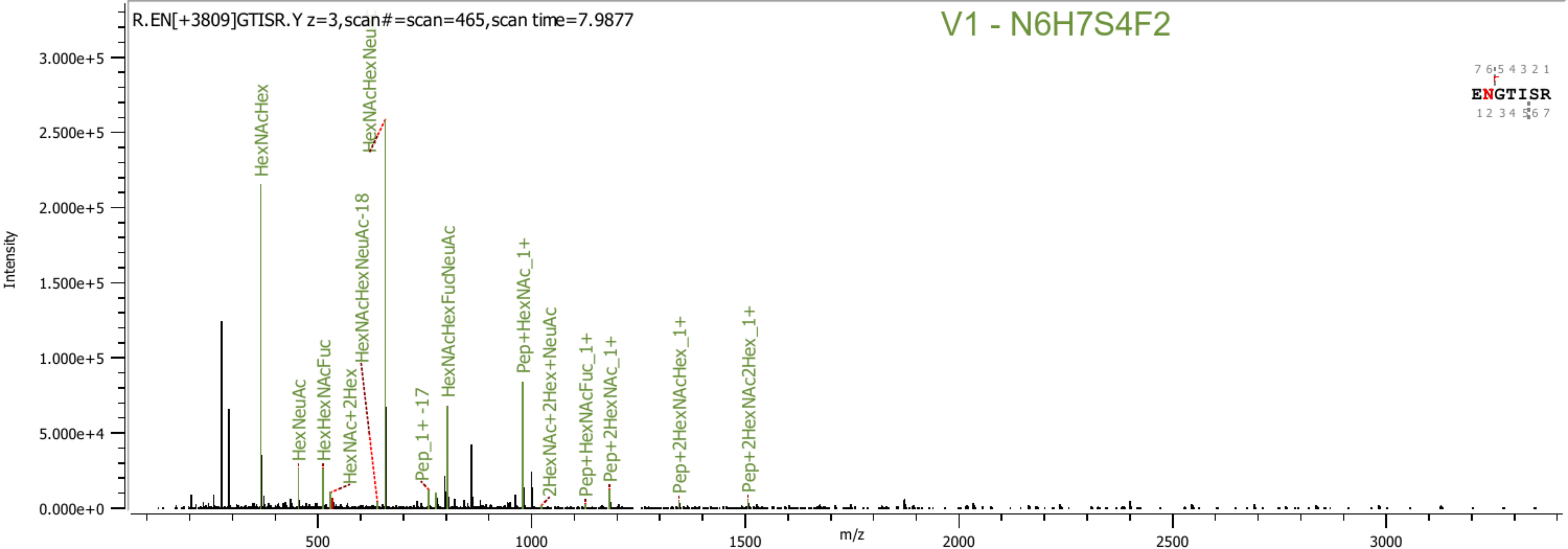

R.EN[+3809]GTISR.Y z=3,scan#=scan=465,scan time=7.9877
V1 - N6H7S4F2
ENGTISR
7 6 5 4 3 2 1
1 2 3 4 5 6 7
Intensity
m/z
0.000e+0
5.000e+4
1.000e+5
1.500e+5
2.000e+5
2.500e+5
3.000e+5
500
1000
1500
2000
2500
3000
HexNAcHex
HexNAcHexNeu
HexNeuAc
HexHexNAcFuc
HexNAc+2Hex
HexNAcHexNeuAc-18
Pep_1+ -17
HexNAcHexFucNeuAc
Pep+HexNAc_1+
2HexNAc+2Hex+NeuAc
Pep+HexNAcFuc_1+
Pep+2HexNAc_1+
Pep+2HexNAcHex_1+
Pep+2HexNAc2Hex_1+

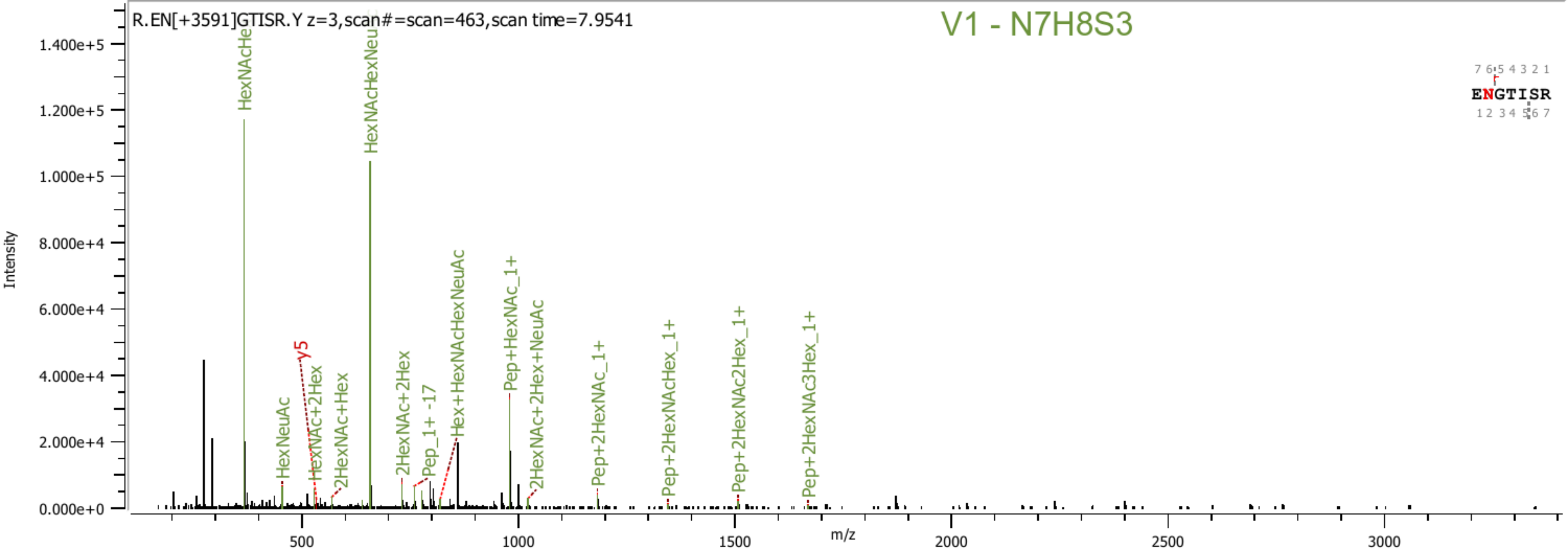

R.EN[+3591]GTISR.Y z=3,scan#=scan=463,scan time=7.9541
V1 - N7H8S3
ENGTISR
Intensity
m/z
HexNAcHe
HexNAcHexNeu
HexNeuAc
y5
HexNAc+2Hex
2HexNAc+Hex
2HexNAc+2Hex
Pep_1+ -17
Hex+HexNAcHexNeuAc
Pep+HexNAc_1+
2HexNAc+2Hex+NeuAc
Pep+2HexNAc_1+
Pep+2HexNAcHex_1+
Pep+2HexNAc2Hex_1+
Pep+2HexNAc3Hex_1+

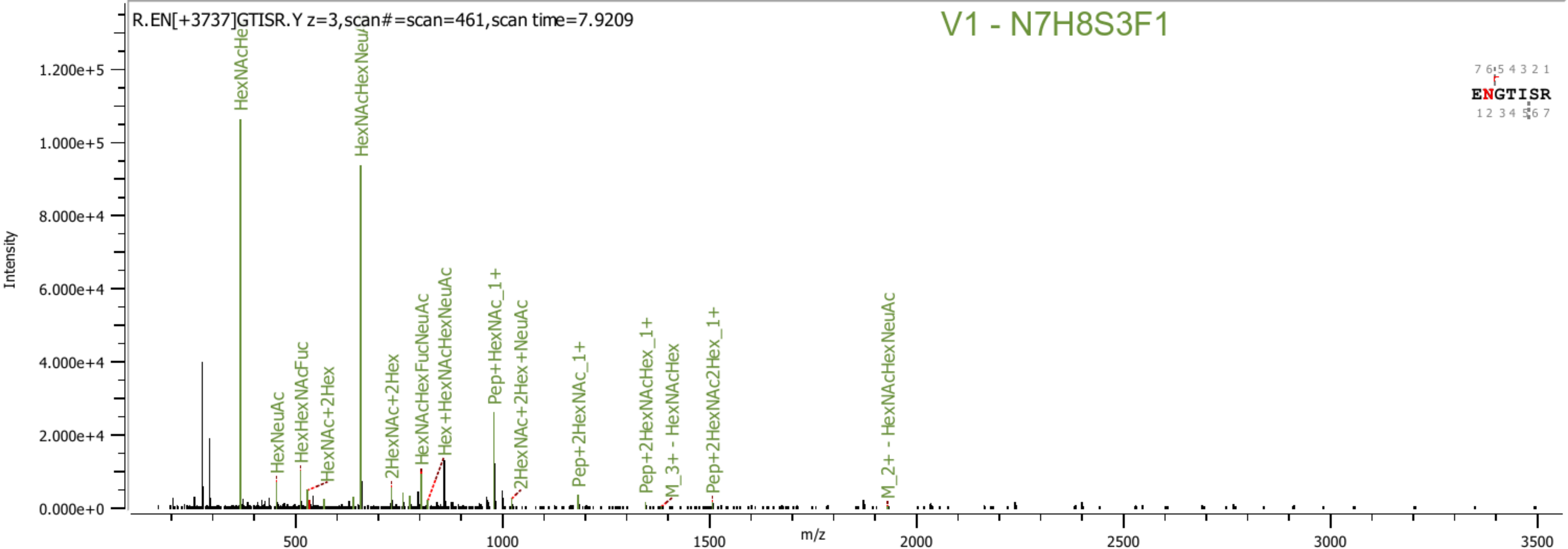
R.EN[+3737]GTISR.Y z=3,scan#=scan=461,scan time=7.9209
V1 - N7H8S3F1
7 6 5 4 3 2 1
ENGTISR
1 2 3 4 5 6 7
Intensity
1.200e+5
1.000e+5
8.000e+4
6.000e+4
4.000e+4
2.000e+4
0.000e+0
HexNAcHe
HexNAcHexNeu
HexNeuAc
HexHexNAcFuc
HexNAc+2Hex
2HexNAc+2Hex
HexNAcHexFucNeuAc
Hex+HexNAcHexNeuAc
Pep+HexNAc_1+
2HexNAc+2Hex+NeuAc
Pep+2HexNAc_1+
Pep+2HexNAcHex_1+
M_3+ - HexNAcHex
Pep+2HexNAc2Hex_1+
M_2+ - HexNAcHexNeuAc
m/z
500
1000
1500
2000
2500
3000
3500

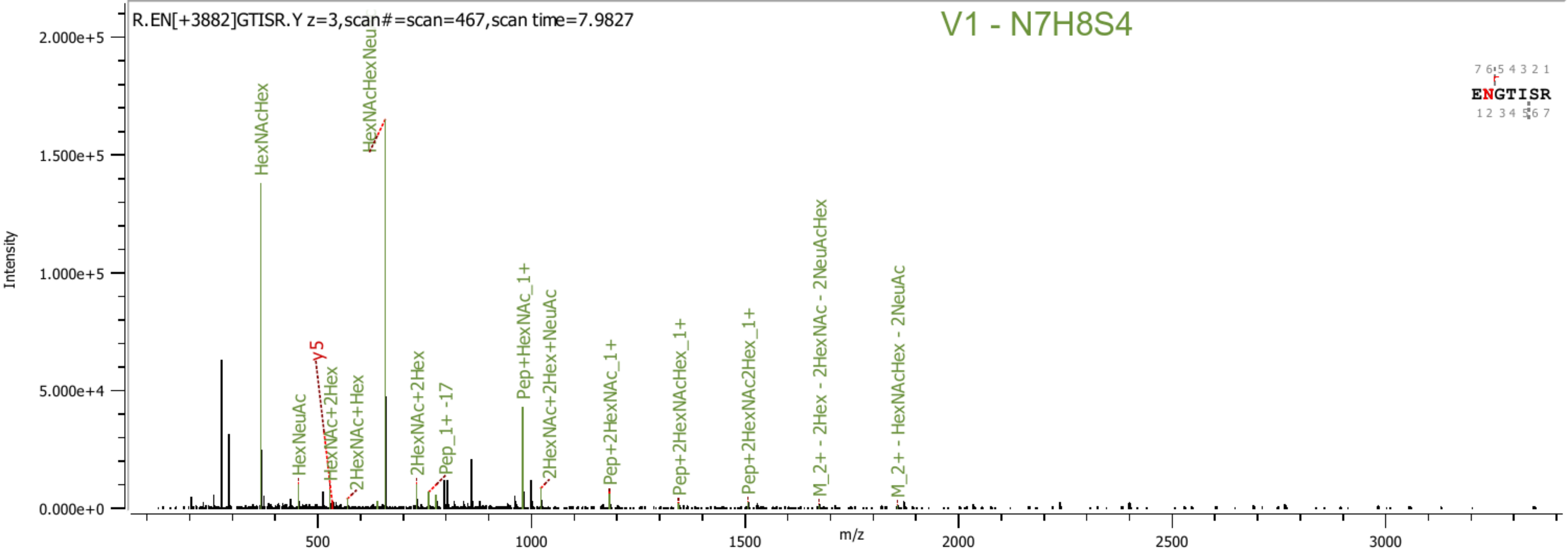

R.EN[+3882]GTISR.Y z=3,scan#=scan=467,scan time=7.9827
V1 - N7H8S4
7 6 5 4 3 2 1
ENGTISR
1 2 3 4 5 6 7
Intensity
2.000e+5
1.500e+5
1.000e+5
5.000e+4
0.000e+0
HexNAcHex
HexNAcHexNeu
HexNeuAc
y5
HexNAc+2Hex
2HexNAc+Hex
2HexNAc+2Hex
Pep_1+ -17
Pep+HexNAc_1+
2HexNAc+2Hex+NeuAc
Pep+2HexNAc_1+
Pep+2HexNAcHex_1+
Pep+2HexNAc2Hex_1+
M_2+ - 2Hex - 2HexNAc - 2NeuAcHex
M_2+ - HexNAcHex - 2NeuAc
500
1000
1500
2000
2500
3000
m/z

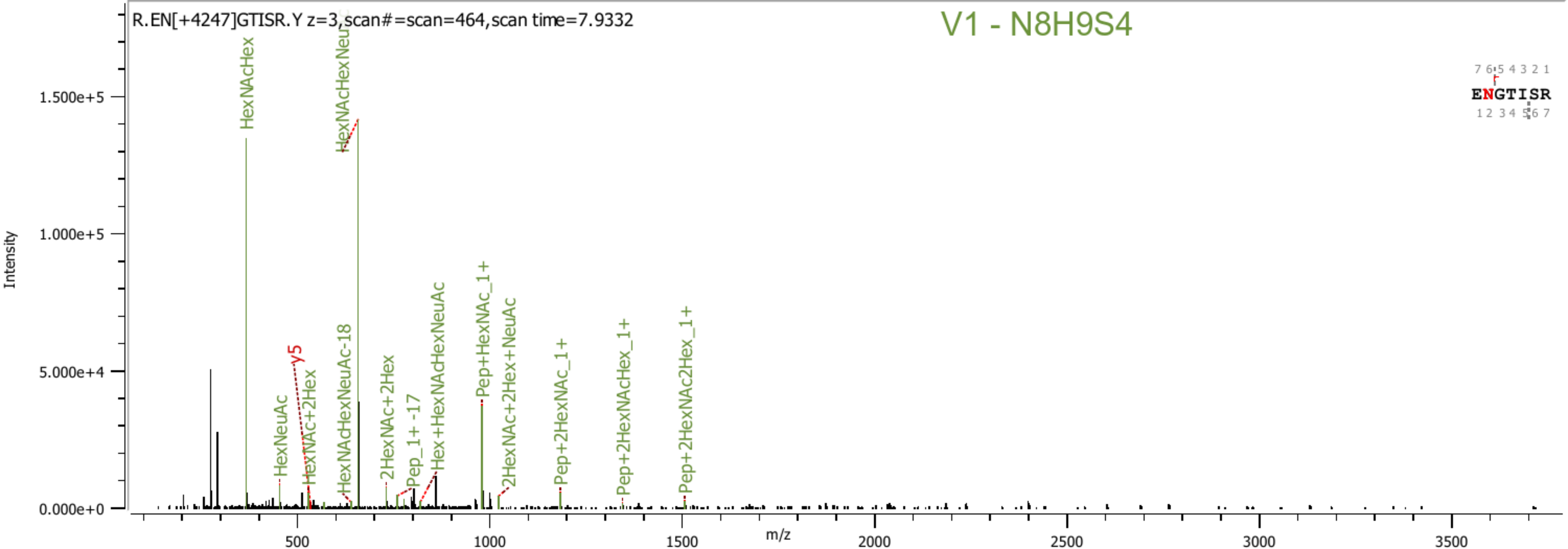
R.EN[+4247]GTISR.Y z=3,scan#=scan=464,scan time=7.9332
V1 - N8H9S4
ENGTISR
Intensity
m/z
1.500e+5
1.000e+5
5.000e+4
0.000e+0
500
1000
1500
2000
2500
3000
3500
HexNAcHex
HexNAcHexNeuAc
HexNeuAc
y5
HexNAc+2Hex
HexNAcHexNeuAc-18
2HexNAc+2Hex
Pep_1+ -17
Hex+HexNAcHexNeuAc
Pep+HexNAc_1+
2HexNAc+2Hex+NeuAc
Pep+2HexNAc_1+
Pep+2HexNAcHex_1+
Pep+2HexNAc2Hex_1+

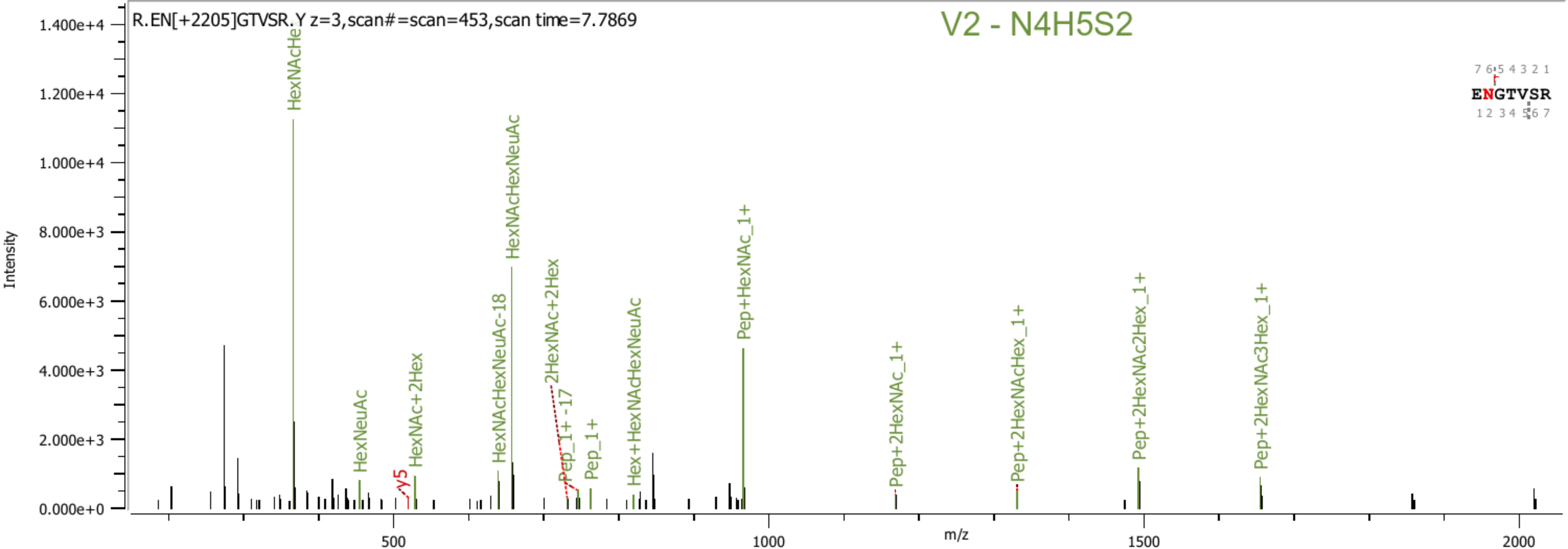

R.EN[+2205]GTVSR.Y z=3,scan#=scan=453,scan time=7.7869
V2 - N4H5S2
ENGTVSR
Intensity
m/z
HexNAcHex
HexNeuAc
y5
HexNAc+2Hex
HexNAcHexNeuAc-18
HexNAcHexNeuAc
2HexNAc+2Hex
Pep_1+ -17
Pep_1+
Hex+HexNAcHexNeuAc
Pep+HexNAc_1+
Pep+2HexNAc_1+
Pep+2HexNAcHex_1+
Pep+2HexNAc2Hex_1+
Pep+2HexNAc3Hex_1+

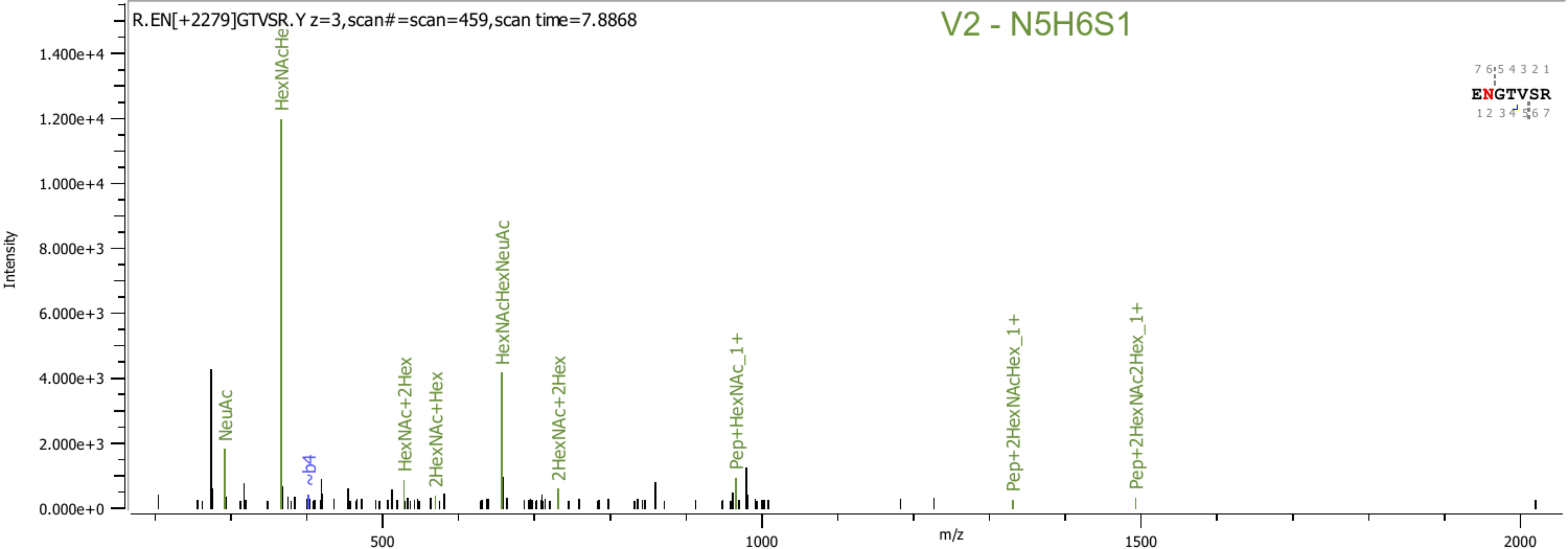
R.EN[+2279]GTVSR.Y z=3,scan#=scan=459,scan time=7.8868
V2 - N5H6S1
Intensity
m/z
NeuAc
HexNAcHe
~b4
HexNAc+2Hex
2HexNAc+Hex
HexNAcHexNeuAc
2HexNAc+2Hex
Pep+HexNAc_1+
Pep+2HexNAcHex_1+
Pep+2HexNAc2Hex_1+
ENGTVSR
0.000e+0
2.000e+3
4.000e+3
6.000e+3
8.000e+3
1.000e+4
1.200e+4
1.400e+4
500
1000
1500
2000

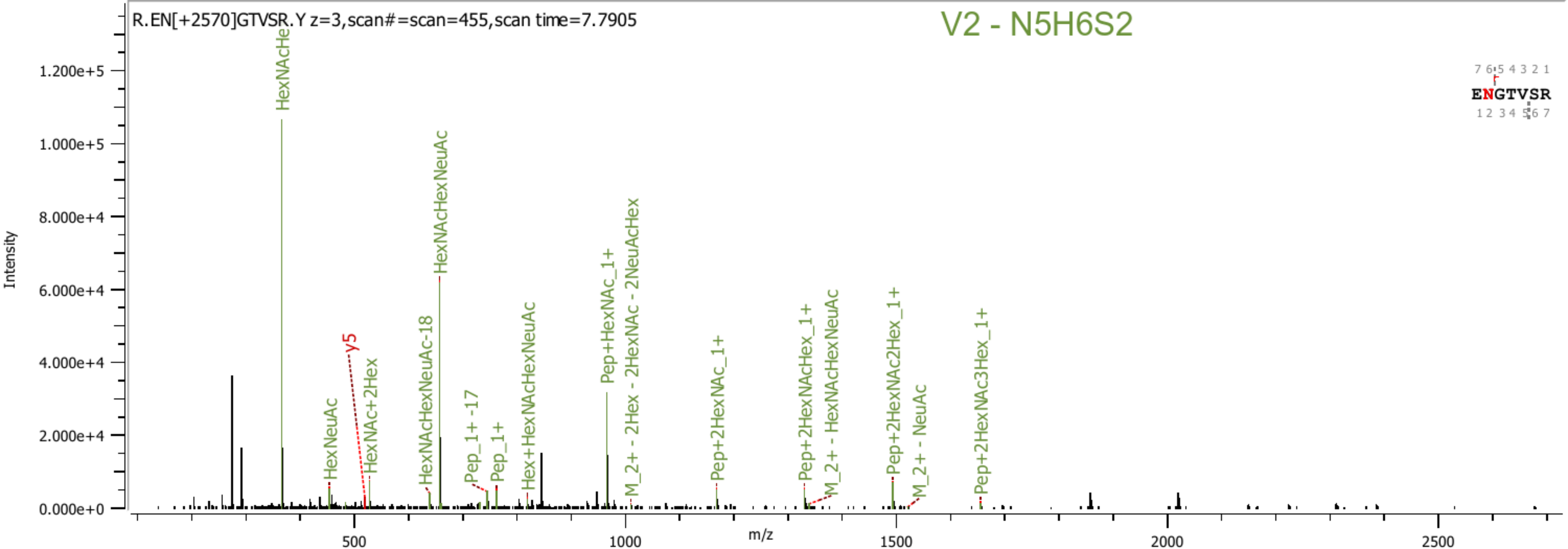

R.EN[+2570]GTVSR.Y z=3,scan#=scan=455,scan time=7.7905
V2 - N5H6S2
ENGTVSR
Intensity
m/z
HexNAcHex
HexNAcHexNeuAc
Pep+HexNAc_1+
M_2+ - 2Hex - 2HexNAc - 2NeuAcHex
y5
HexNeuAc
HexNAc+2Hex
HexNAcHexNeuAc-18
Pep_1+ -17
Pep_1+
Hex+HexNAcHexNeuAc
Pep+2HexNAc_1+
Pep+2HexNAcHex_1+
M_2+ - HexNAcHexNeuAc
Pep+2HexNAc2Hex_1+
M_2+ - NeuAc
Pep+2HexNAc3Hex_1+

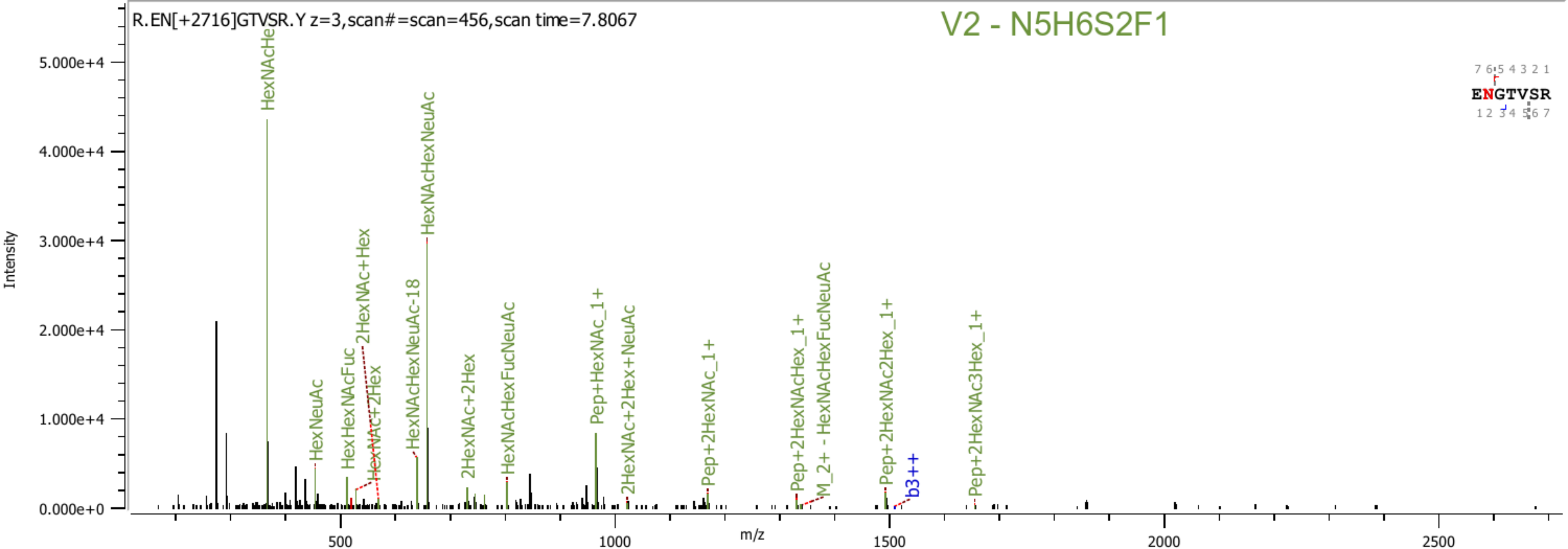
R.EN[+2716]GTVSR.Y z=3,scan#=scan=456,scan time=7.8067
V2 - N5H6S2F1
ENGTVSR
HexNAcHex
HexNAcHexNeuAc
2HexNAc+Hex
HexNAcHexNeuAc-18
HexNeuAc
HexHexNAcFuc
HexNAc+2Hex
2HexNAc+2Hex
HexNAcHexFucNeuAc
Pep+HexNAc_1+
2HexNAc+2Hex+NeuAc
Pep+2HexNAc_1+
Pep+2HexNAcHex_1+
M_2+ - HexNAcHexFucNeuAc
Pep+2HexNAc2Hex_1+
b3++
Pep+2HexNAc3Hex_1+
Intensity
m/z

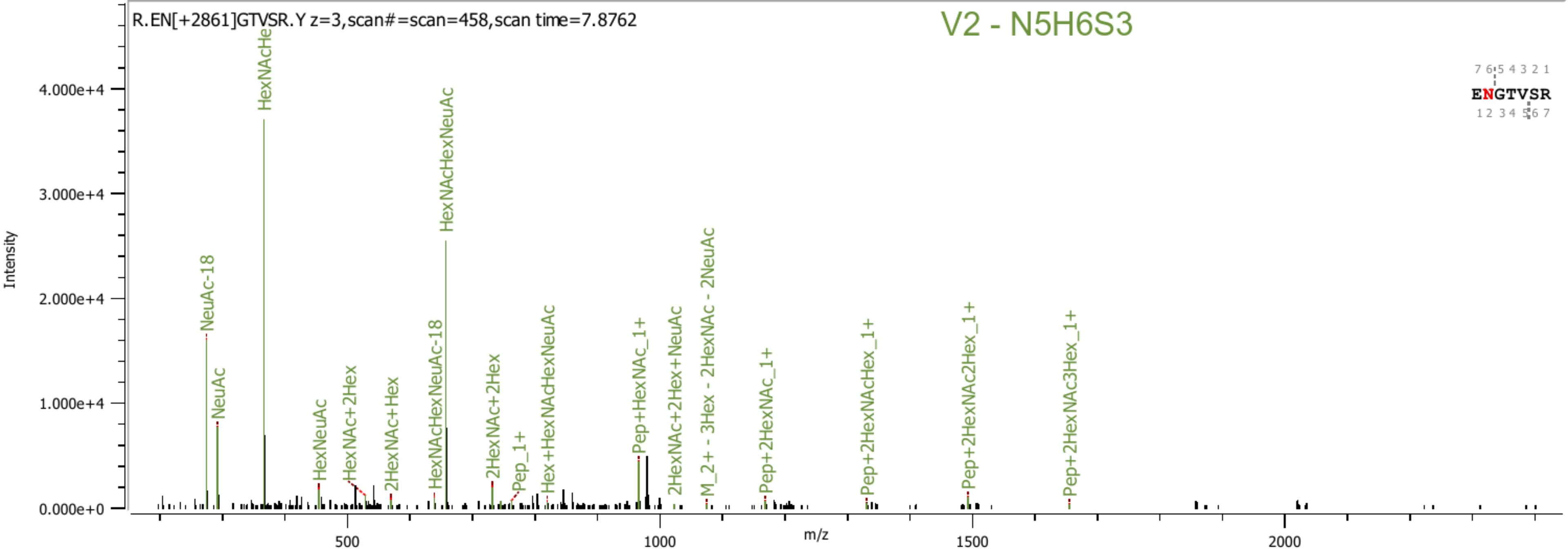

R.EN[+2861]GTVSR.Y z=3,scan#=scan=458,scan time=7.8762
V2 - N5H6S3
ENGTVSR
Intensity
m/z
NeuAc-18
NeuAc
HexNAcHe
HexNeuAc
HexNAc+2Hex
2HexNAc+Hex
HexNAcHexNeuAc-18
HexNAcHexNeuAc
2HexNAc+2Hex
Pep_1+
Hex+HexNAcHexNeuAc
Pep+HexNAc_1+
2HexNAc+2Hex+NeuAc
M_2+ - 3Hex - 2HexNAc - 2NeuAc
Pep+2HexNAc_1+
Pep+2HexNAcHex_1+
Pep+2HexNAc2Hex_1+
Pep+2HexNAc3Hex_1+

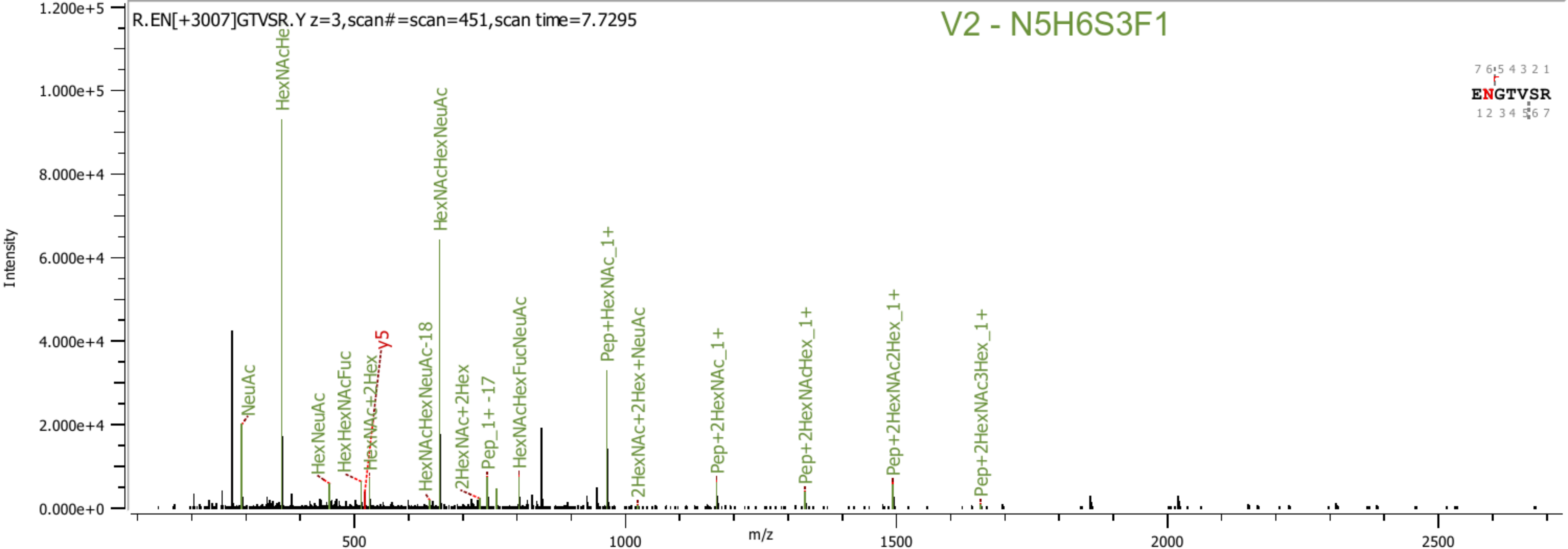

R.EN[+3007]GTVSR.Y z=3,scan#=scan=451,scan time=7.7295
V2 - N5H6S3F1
7 6 5 4 3 2 1
ENGTVSR
1 2 3 4 5 6 7
Intensity
1.200e+5
1.000e+5
8.000e+4
6.000e+4
4.000e+4
2.000e+4
0.000e+0
m/z
500
1000
1500
2000
2500
NeuAc
HexNAcHex
HexNeuAc
HexHexNAcFuc
HexNAc+2Hex
y5
HexNAcHexNeuAc-18
HexNAcHexNeuAc
2HexNAc+2Hex
Pep_1+ -17
HexNAcHexFucNeuAc
Pep+HexNAc_1+
2HexNAc+2Hex+NeuAc
Pep+2HexNAc_1+
Pep+2HexNAcHex_1+
Pep+2HexNAc2Hex_1+
Pep+2HexNAc3Hex_1+

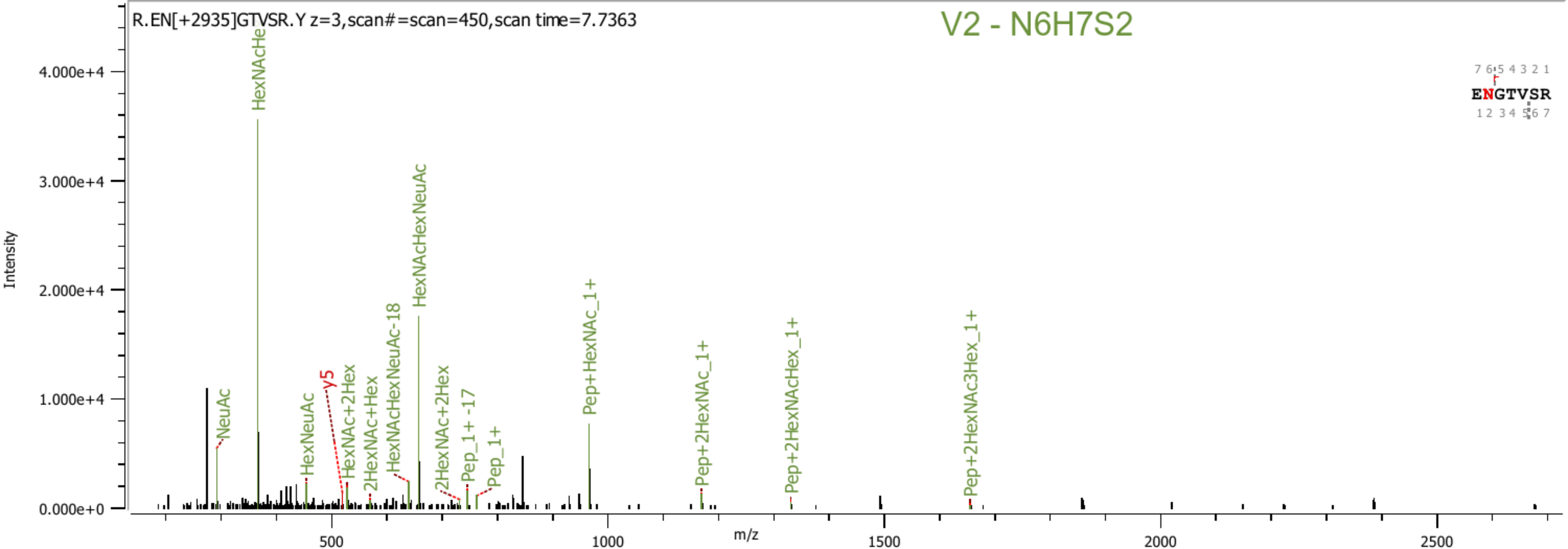
R.EN[+2935]GTVSR.Y z=3,scan#=scan=450,scan time=7.7363
V2 - N6H7S2
ENGTVSR
Intensity
4.000e+4
3.000e+4
2.000e+4
1.000e+4
0.000e+0
m/z
500
1000
1500
2000
2500
HexNAcHe
NeuAc
HexNeuAc
y5
HexNAc+2Hex
2HexNAc+Hex
HexNAcHexNeuAc-18
HexNAcHexNeuAc
2HexNAc+2Hex
Pep_1+ -17
Pep_1+
Pep+HexNAc_1+
Pep+2HexNAc_1+
Pep+2HexNAcHex_1+
Pep+2HexNAc3Hex_1+

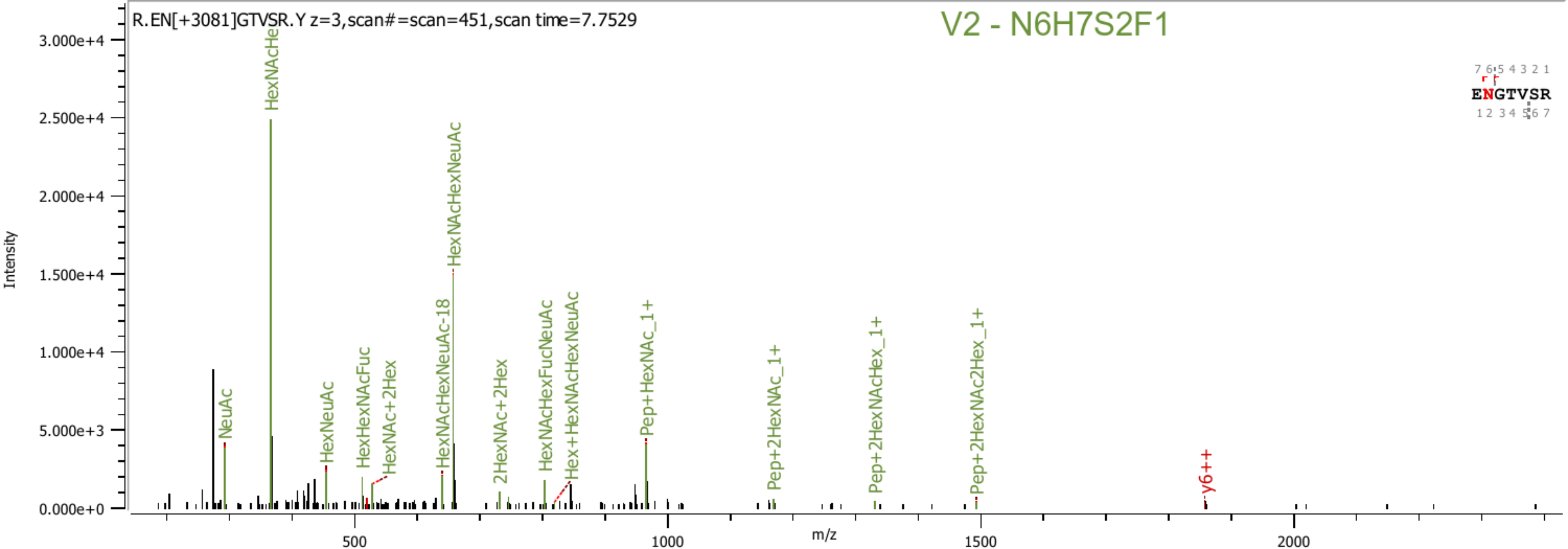
R.EN[+3081]GTVSR.Y z=3,scan#=scan=451,scan time=7.7529
V2 - N6H7S2F1
Intensity
m/z
3.000e+4
2.500e+4
2.000e+4
1.500e+4
1.000e+4
5.000e+3
0.000e+0
500
1000
1500
2000
NeuAc
HexNAcHe
HexNeuAc
HexHexNAcFuc
HexNAc+2Hex
HexNAcHexNeuAc-18
HexNAcHexNeuAc
2HexNAc+2Hex
HexNAcHexFucNeuAc
Hex+HexNAcHexNeuAc
Pep+HexNAc_1+
Pep+2HexNAc_1+
Pep+2HexNAcHex_1+
Pep+2HexNAc2Hex_1+
y6++
ENGTVSR

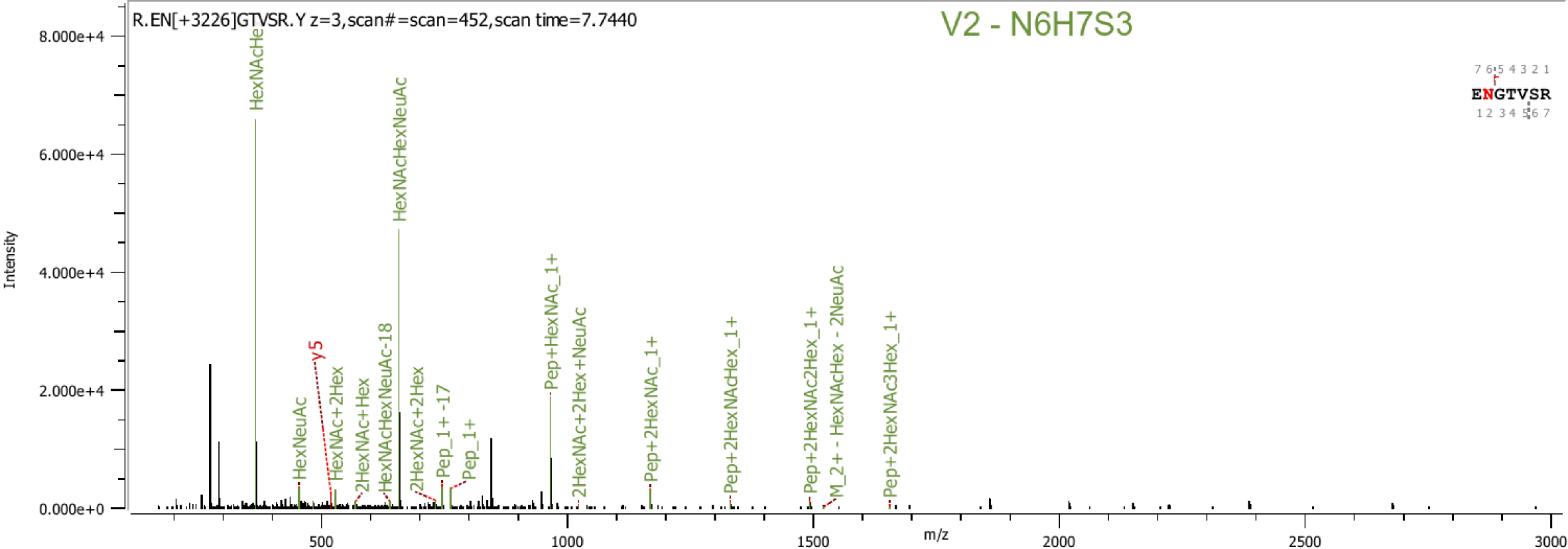

R.EN[+3226]GTVSR.Y z=3,scan#=scan=452,scan time=7.7440
V2 - N6H7S3
Intensity
m/z
HexNAcHe
HexNAcHexNeuAc
HexNeuAc
y5
HexNAc+2Hex
2HexNAc+Hex
HexNAcHexNeuAc-18
2HexNAc+2Hex
Pep_1+ -17
Pep_1+
Pep+HexNAc_1+
2HexNAc+2Hex+NeuAc
Pep+2HexNAc_1+
Pep+2HexNAcHex_1+
Pep+2HexNAc2Hex_1+
M_2+ - HexNAcHex - 2NeuAc
Pep+2HexNAc3Hex_1+
ENGTVSR

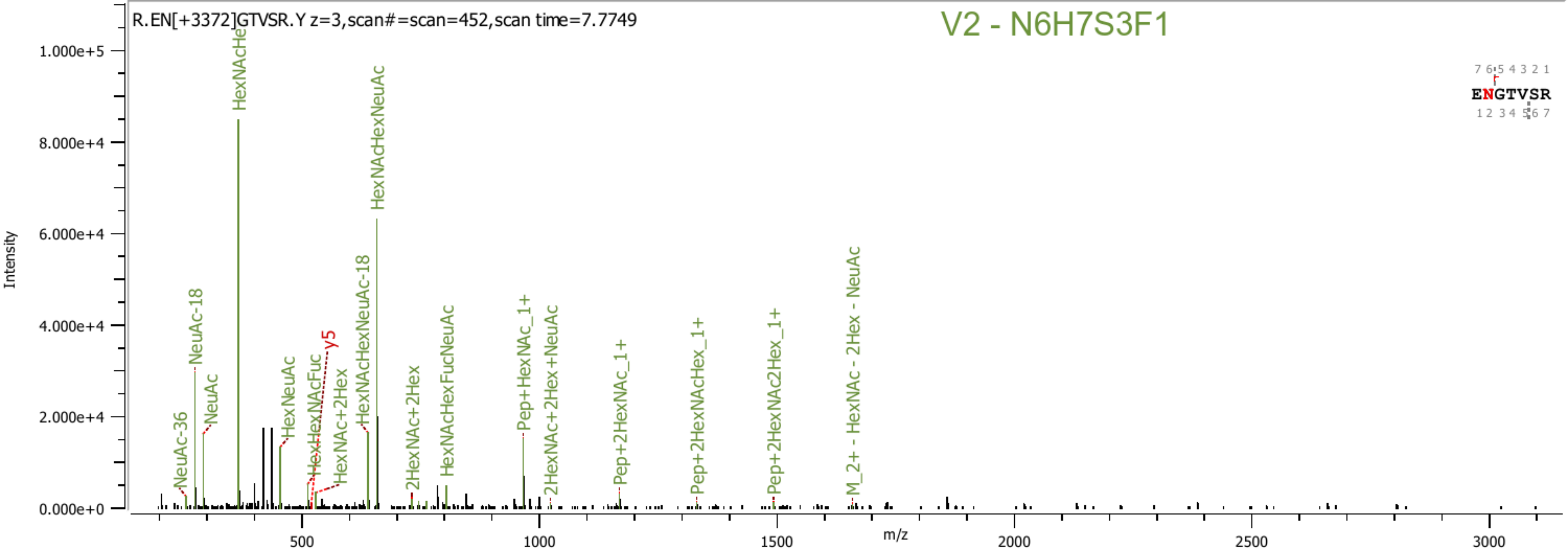
R.EN[+3372]GTVSR.Y z=3,scan#=scan=452,scan time=7.7749
V2 - N6H7S3F1
7 6 5 4 3 2 1
ENGTVSR
1 2 3 4 5 6 7
Intensity
1.000e+5
8.000e+4
6.000e+4
4.000e+4
2.000e+4
0.000e+0
NeuAc-36
NeuAc-18
NeuAc
HexNAcHex
HexNeuAc
HexHexNAcFuc
y5
HexNAc+2Hex
HexNAcHexNeuAc-18
HexNAcHexNeuAc
2HexNAc+2Hex
HexNAcHexFucNeuAc
Pep+HexNAc_1+
2HexNAc+2Hex+NeuAc
Pep+2HexNAc_1+
Pep+2HexNAcHex_1+
Pep+2HexNAc2Hex_1+
M_2+ - HexNAc - 2Hex - NeuAc
500
1000
1500
2000
2500
3000
m/z

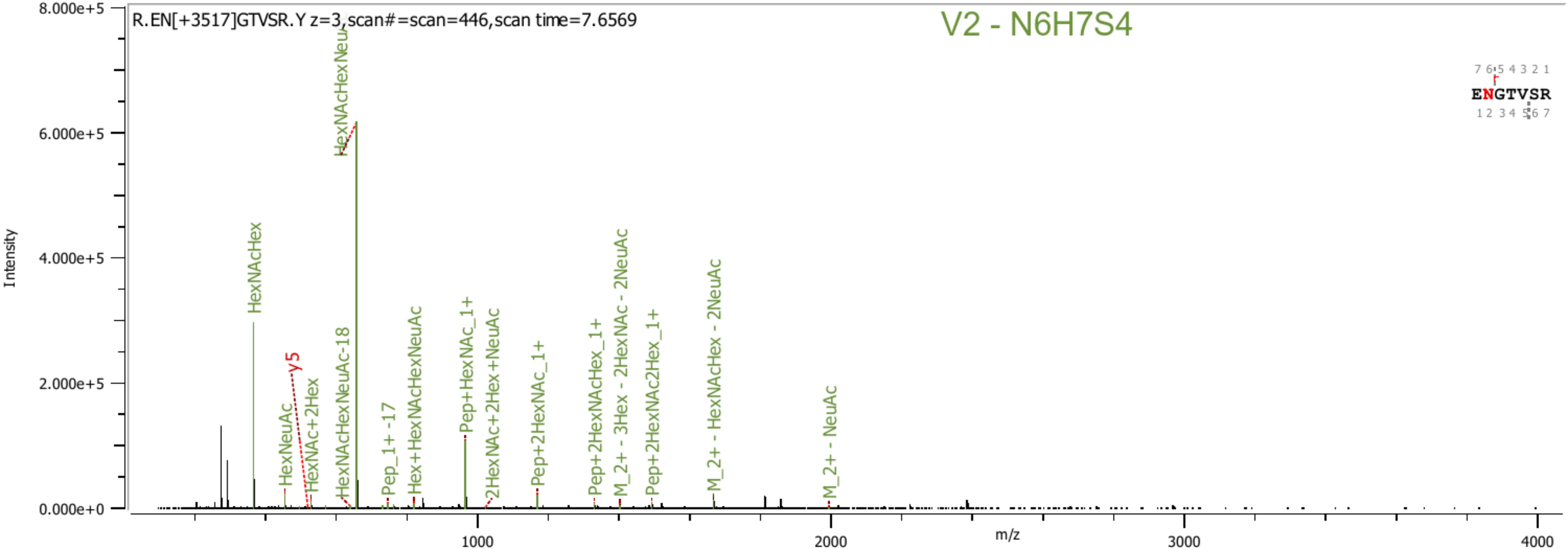
R.EN[+3517]GTVSR.Y z=3,scan#=scan=446,scan time=7.6569
V2 - N6H7S4
ENGTVSR
Intensity
8.000e+5
6.000e+5
4.000e+5
2.000e+5
0.000e+0
HexNAcHex
HexNeuAc
y5
HexNAc+2Hex
HexNAcHexNeuAc
HexNAcHexNeuAc-18
Pep_1+ -17
Hex+HexNAcHexNeuAc
Pep+HexNAc_1+
2HexNAc+2Hex+NeuAc
Pep+2HexNAc_1+
Pep+2HexNAcHex_1+
M_2+ - 3Hex - 2HexNAc - 2NeuAc
Pep+2HexNAc2Hex_1+
M_2+ - HexNAcHex - 2NeuAc
M_2+ - NeuAc
1000
2000
3000
4000
m/z

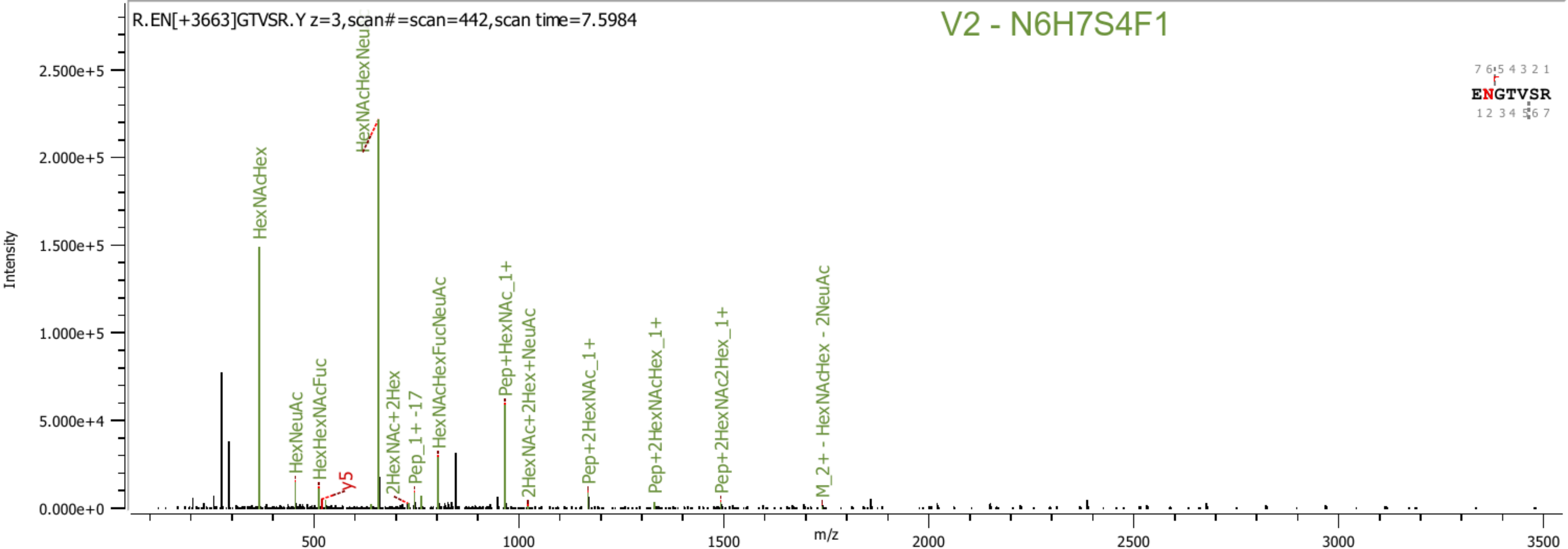
R.EN[+3663]GTVSR.Y z=3,scan#=scan=442,scan time=7.5984
V2 - N6H7S4F1
ENGTVSR
Intensity
m/z
HexNAcHex
HexNAcHexNeuAc
HexNeuAc
HexHexNAcFuc
y5
2HexNAc+2Hex
Pep_1+ -17
HexNAcHexFucNeuAc
Pep+HexNAc_1+
2HexNAc+2Hex+NeuAc
Pep+2HexNAc_1+
Pep+2HexNAcHex_1+
Pep+2HexNAc2Hex_1+
M_2+ - HexNAcHex - 2NeuAc

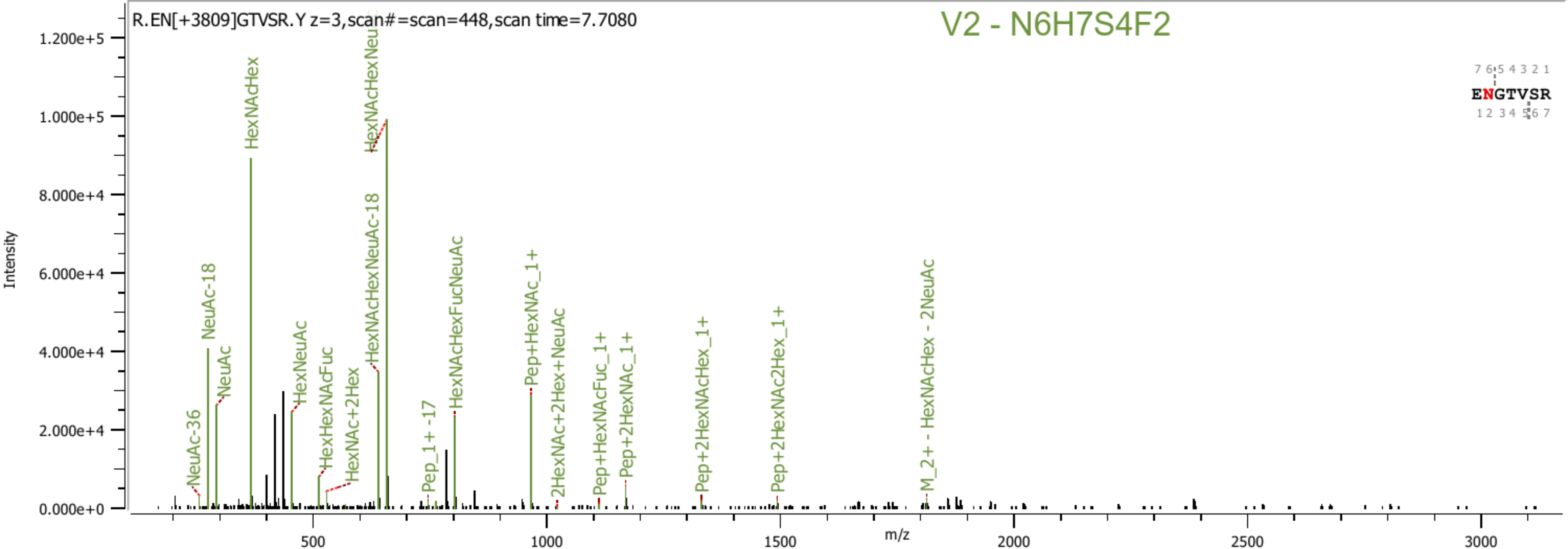

R.EN[+3809]GTVSR.Y z=3,scan#=scan=448,scan time=7.7080
V2 - N6H7S4F2
7 6 5 4 3 2 1
ENGTVSR
1 2 3 4 5 6 7
Intensity
m/z
NeuAc-36
NeuAc-18
NeuAc
HexNAcHex
HexNeuAc
HexHexNAcFuc
HexNAc+2Hex
HexNAcHexNeuAc-18
HexNAcHexNeuAc
Pep_1+ -17
HexNAcHexFucNeuAc
Pep+HexNAc_1+
2HexNAc+2Hex+NeuAc
Pep+HexNAcFuc_1+
Pep+2HexNAc_1+
Pep+2HexNAcHex_1+
Pep+2HexNAc2Hex_1+
M_2+ - HexNAcHex - 2NeuAc

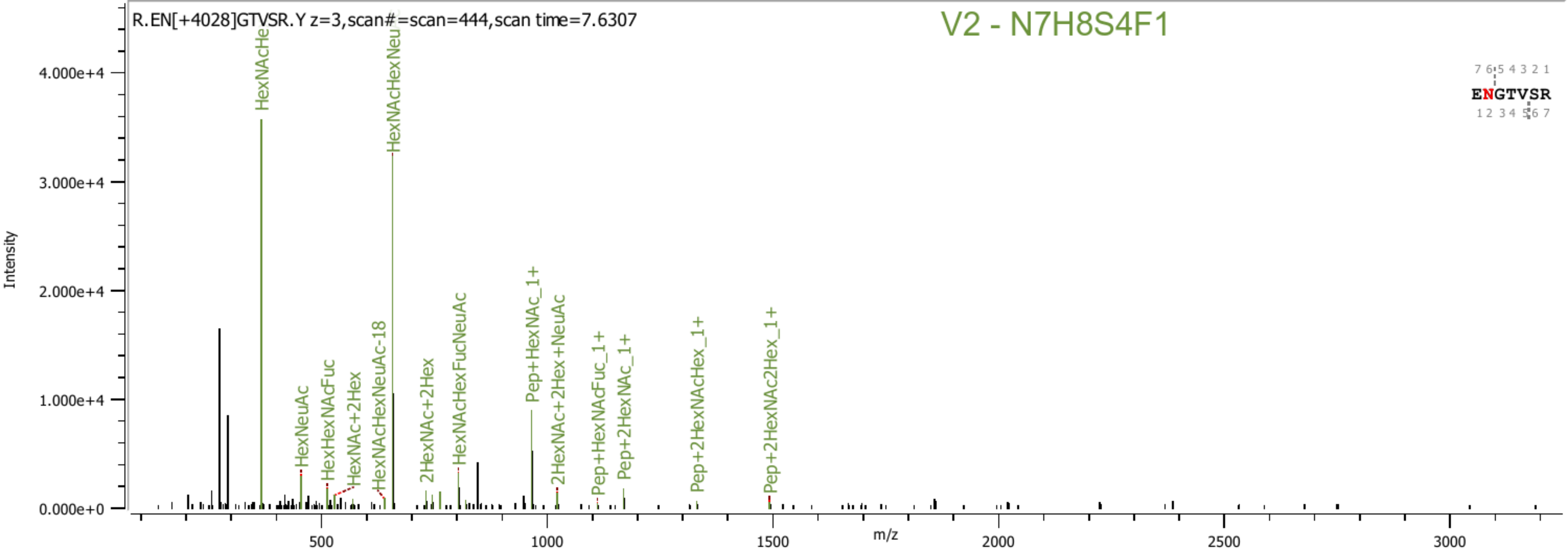
R.EN[+4028]GTVSR.Y z=3,scan#=scan=444,scan time=7.6307
V2 - N7H8S4F1
ENGTVSR
Intensity
m/z
HexNAcHe
HexNAcHexNeu
HexNeuAc
HexHexNAcFuc
HexNAc+2Hex
HexNAcHexNeuAc-18
2HexNAc+2Hex
HexNAcHexFucNeuAc
Pep+HexNAc_1+
2HexNAc+2Hex+NeuAc
Pep+HexNAcFuc_1+
Pep+2HexNAc_1+
Pep+2HexNAcHex_1+
Pep+2HexNAc2Hex_1+

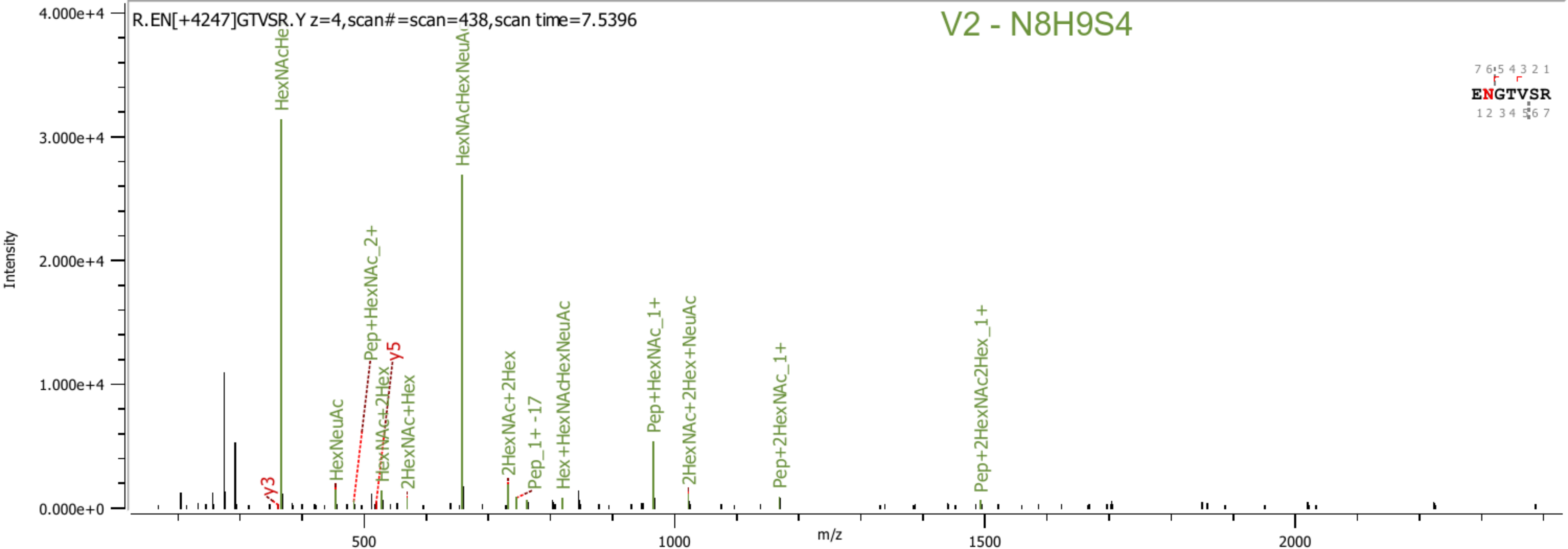
R.EN[+4247]GTVSR.Y z=4,scan#=scan=438,scan time=7.5396
V2 - N8H9S4
ENGTVSR
Intensity
m/z
HexNAcHex
HexNAcHexNeuAc
HexNeuAc
Pep+HexNAc_2+
y5
y3
HexNAc+2Hex
2HexNAc+Hex
2HexNAc+2Hex
Pep_1+ -17
Hex+HexNAcHexNeuAc
Pep+HexNAc_1+
2HexNAc+2Hex+NeuAc
Pep+2HexNAc_1+
Pep+2HexNAc2Hex_1+

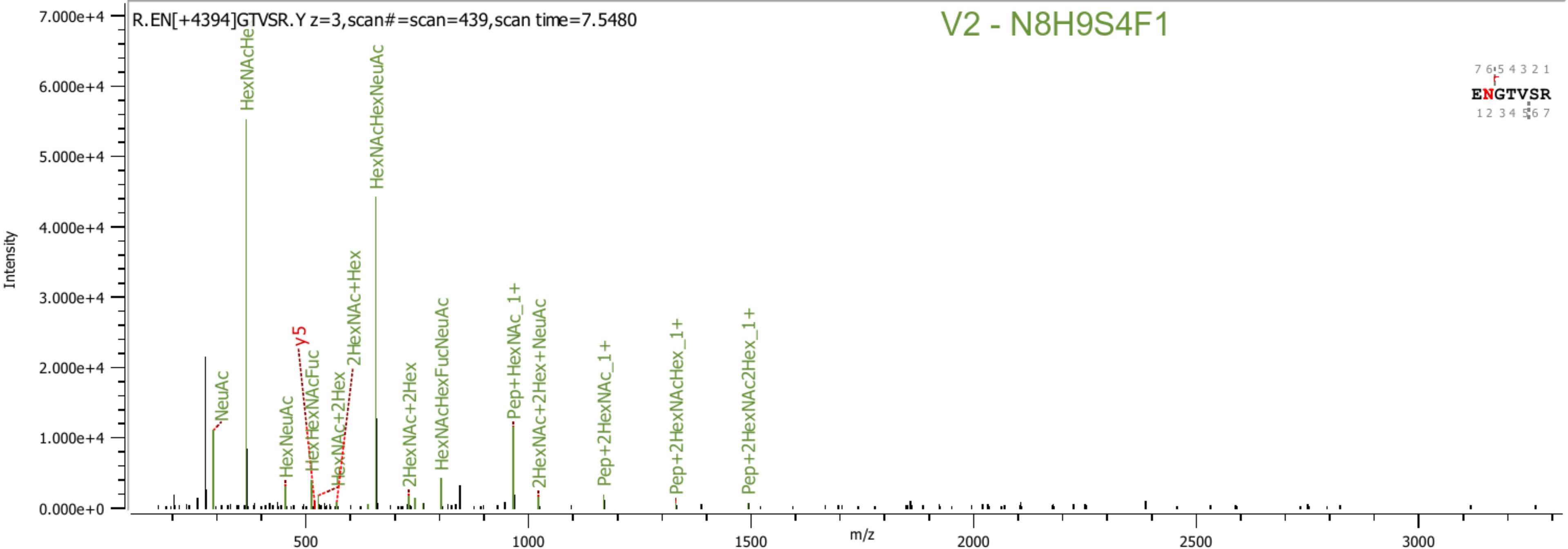

R.EN[+4394]GTVSR.Y z=3,scan#=scan=439,scan time=7.5480
V2 - N8H9S4F1
ENGTVSR
Intensity
m/z
NeuAc
HexNAcHe
HexNeuAc
y5
HexHexNAcFuc
HexNAc+2Hex
2HexNAc+Hex
HexNAcHexNeuAc
2HexNAc+2Hex
HexNAcHexFucNeuAc
Pep+HexNAc_1+
2HexNAc+2Hex+NeuAc
Pep+2HexNAc_1+
Pep+2HexNAcHex_1+
Pep+2HexNAc2Hex_1+
